# Supplementary material for: Chiral phosphoric acid-catalyzed enantioselective phosphinylation of 3,4-dihydroisoquinolines with diarylphosphine oxides
Source: Commun Chem. 2023 Feb 9;6:26. doi: 10.1038/s42004-023-00826-4 (PMC9911717; doi:10.1038/s42004-023-00826-4)
Supplement: Supplementary file 2 — Supplementary Information [file 42004_2023_826_MOESM2_ESM.pdf]

Chiral phosphoric acid-catalyzed enantioselective phosphinylation of 3,4-dihydroisoquinolines with diarylphosphine oxides

Yongbiao Guo,[a*] Ning Li, Junchen Li, Xiaojing Bi, Zhenhua Gao,[a*] Ya-Nan Duan,[b*] Junhua Xiao[a*]

[a]State Key Laboratory of NBC Protection for Civilian, Beijing 102205, China

[b]Chemistry and Chemical Engineering Guangdong Laboratory, Shantou 515031, China

[*]Corresponding author (van87120@126.com, gaozhenhua1223@163.com, duanyanan008_work@163.com, xiao.junhua@pku.edu.cn)

Table of Contents

1. General information 2

2. Reaction conditions optimization for the enantioselective phosphinylation of 3,4-dihydroisoquinoline. 3

3. Control and NMR tracking experiments 7

4. Supplementary Notes: 10

5. Characterization of products 10

6. DFT Calculations 30

7. Supplementary References 32

Supplementary Methods

1. General information

General information: Reagents and solvents were purchased from common commercial suppliers and were used without further purification. Column chromatography was generally performed on silica gel (200-300 mesh). Melting points were determined with a Büchi B-545 melting-point apparatus. 600MHz ^{1}H NMR and 151MHz ^{13}C NMR spectra were recorded on Varian VMS-600 spectrometers, respectively. The chemical shifts are reported in ppm (δ scale) relative to internal tetramethylsilane, and coupling constants are reported in hertz (Hz). High-resolution mass spectra (HRMS) were obtained on Agilent 6502 Q-TOF HPLC and mass spectrometry.

2. Reaction conditions optimization for the enantioselective phosphinylation of 3,4-dihydroisoquinoline.

Supplementary Table 1. Chiral Brønsted Acid Catalyst Evaluation[a]

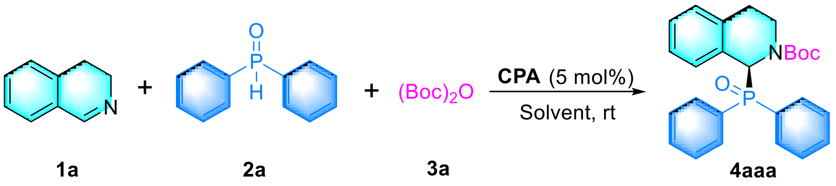

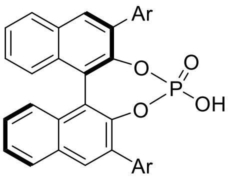

cat.1a, R = Ph, X = OH;
cat.1b, R = adamantane, X = OH;
cat.1c, R = 1-naphthyl, X = OH;
cat.1d, R = 2-naphthyl, X = OH;
cat.1e, R = 9-anthracenyl, X = OH;
cat.1f, R = 9-phenanthryl, X = OH;
cat.1g, R = 1-pyrenyl, X = OH;
cat.1h, R = $SiPh_3$, X = OH;
cat.1i, R = 4-$NO_2C_6H_4$, X = OH;
cat.1j, R = 4-$CF_3C_6H_4$, X = OH;
cat.1k, R = 4-PhC_6H_4, X = OH;
cat.1l, R = 4-*t*-BuC_6H_4, X = OH;
cat.1m,R = 4-$OMeC_6H_4$, X = OH;
cat.1n, R = 4-(2-naphthyl)C_6H_4, X = OH;
cat.1o, R = 2-OMe-5-*t*-BuC_6H_3, X = OH;
cat.1p, R = 2,6-$OMe_2C_6H_3$, X = OH;
cat.1q, R = 3,5-$(CF_3)_2C_6H_3$, X = OH;
cat.1r, R = 3,5-$Cl_2C_6H_3$, X = OH;
cat.1s, R = 3,5-$Me_2C_6H_3$, X = OH;
cat.1t, R = 3,5-*t*-$Bu_2C_6H_3$, X = OH;
cat.1u, R = 3,5-(3,5-*t*-$Bu_2C_6H_3)_2C_6H_3$, X = OH;
cat.1v, R = 3,5-*t*-Bu_2-4-$OMeC_6H_2$, X = OH;
cat.1w,R = 2,4,6-$Me_3C_6H_2$, X = OH;
cat.1x, R = 2,4,6-*i*-$Pr_3C_6H_2$, X = OH;
cat.1y, R = 2,4,6-$Ph_3C_6H_2$, X = OH;
cat.1z, R = 2,4,6-$Cy_3C_6H_2$, X = OH;
cat.1a', R = 2,4,6-$OMe_3C_6H_2$, X = OH;
cat.1b', R = 2,6-*i*-Pr_2-4-(9-anthracenyl)C_6H_2, X = OH;
cat.5a, R = 9-anthracenyl, X = NTf;
cat.5b, R = 9-phenanthryl, X = NTf;

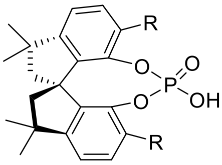

cat.3a, R = 9-anthracenyl
cat.3b, R = 9-phenanthryl
cat.3c, R = 2-naphthyl
cat.3d, R = 3,5-*t*-Bu_2-4-$OMeC_6H_2$
cat.3e,R = 2,4,6-$Me_3C_6H_2$
cat.3f, R = 2,4,6-*i*-$Pr_3C_6H_2$

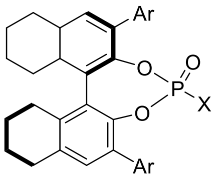

cat.2a, R = Ph, X = OH;
cat.2b, R = 1-naphthyl, X = OH;
cat.2c, R = 2-naphthyl, X = OH;
cat.2d, R = 9-anthracenyl, X = OH;
cat.2e, R = 9-phenanthryl, X = OH;
cat.2f, R = 1-pyrenyl, X = OH;
cat.2g, R = $SiPh_3$, X = OH;
cat.2h, R = 4-ClC_6H_4, X = OH;
cat.2i, R = 4-$NO_2C_6H_4$, X = OH;
cat.2j, R = 4-$CF_3C_6H_4$, X = OH;
cat.2k, R = 4-PhC_6H_4, X = OH;
cat.2l, R = 4-*t*-BuC_6H_4, X = OH;
cat.2m,R = 4-$OMeC_6H_4$, X = OH;
cat.2n, R = 4-(2-naphthyl)C_6H_4, X = OH;
cat.2o, R = 3,5-$(CF_3)_2C_6H_3$, X = OH;
cat.2p, R = 3,5-$Me_2C_6H_3$, X = OH;
cat.2q, R = 3,5-$Ph_2C_6H_3$, X = OH;
cat.2r,R = 2,4,6-$Me_3C_6H_2$, X = OH;
cat.2s, R = 2,4,6-*i*-$Pr_3C_6H_2$, X = OH;
cat.2t, R = 3,5-*t*-Bu_2-4-$OMeC_6H_2$, X = OH;
cat.6a, R = 9-anthracenyl, X = NTf;
cat.6b, R = 9-phenanthryl, X = NTf;

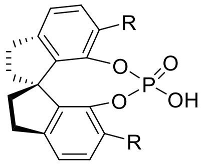

cat.4a, R = 9-anthracenyl
cat.4b, R = 9-phenanthryl
cat.4c, R = 1-pyrenyl
cat.4d, R = $SiPh_3$
cat.4e, R = 4-$CF_3C_6H_4$
cat.4f, R = 4-PhC_6H_4
cat.4g, R = 4-*t*-BuC_6H_4
cat.4h, R = 4-(2-naphthyl)C_6H_4
cat.4i, R = 3,5-$(CF_3)_2C_6H_3$
cat.4j, R = 3,5-$Cl_2C_6H_3$
cat.4k, R = 3,5-$Me_2C_6H_3$
cat.4l, R = 3,5-$Ph_2C_6H_3$
cat.4m,R = 3,5-*t*-Bu_2-4-$OMeC_6H_2$
cat.4n, R = 2,4,6-$Me_3C_6H_2$
cat.4o, R = 2,4,6-*i*-$Pr_3C_6H_2$

Entry	cat.	Yield (%)[b]	ee (%)[c]
1	-	60	0
2	**cat.1a**	99	7
3	**cat.1b**	86	1
4	**cat.1c**	46	4
5	**cat.1d**	89	17
6	**cat.1e**	99	16
7	**cat.1f**	65	22
8	**cat.1g**	83	10
9	**cat.1h**	94	5
10	**cat.1i**	92	5
11	**cat.1j**	85	9
12	**cat.1k**	89	1
13	**cat.1l**	96	2
14	**cat.1m**	87	2
15	**cat.1n**	95	1
16	**cat.1o**	99	2
17	**cat.1p**	96	5
18	**cat.1q**	98	1
19	**cat.1r**	79	10
20	**cat.1s**	94	10
21	**cat.1t**	88	3
22	**cat.1u**	90	4
23	**cat.1v**	95	12
24	**cat.1w**	93	10
25	**cat.1x**	87	6
26	**cat.1y**	93	1
27	**cat.1z**	99	1
28	**cat.1a’**	87	1
29	**cat.1b’**	90	1
30	**cat.2a**	94	6
31	**cat.2b**	95	31
32	**cat.2c**	99	17
33	**cat.2d (CPA 7)**	**90**	**44**
34	**cat.2e**	99	34
35	**cat.2f**	99	39
36	**cat.2g**	80	0
37	**cat.2h**	95	16
38	**cat.2i**	80	0
39	**cat.2j**	98	6
40	**cat.2k**	95	8
41	**cat.2l**	99	6
42	**cat.2m**	97	6

43	**cat.2n**	95	6
44	**cat.2o**	95	0
45	**cat.2p**	88	4
46	**cat.2q**	90	25
47	**cat.2r**	96	33
48	**cat.2s**	99	6
49	**cat.2t**	98	4
50	**cat.3a**	84	22
51	**cat.3b**	99	2
52	**cat.3c**	85	1
53	**cat.3d**	90	1
54	**cat.3e**	93	0
55	**cat.3f**	99	3
56	**cat.4a**	95	0
57	**cat.4b**	90	2
58	**cat.4c**	99	1
59	**cat.4d**	98	0
60	**cat.4e**	92	2
61	**cat.4f**	90	1
62	**cat.4g**	84	0
63	**cat.4h**	88	1
64	**cat.4i**	96	1
65	**cat.4j**	99	8
66	**cat.4k**	87	1
67	**cat.4l**	95	1
68	**cat.4m**	98	0
69	**cat.4n**	99	0
70	**cat.4o**	99	0
71	**cat.5a**	89	19
72	**cat.5b**	95	27
73	**cat.6a**	70	17
74	**cat.6b**	63	15

[a]Reaction conditions: **1a** (0.2 mmol), **3a** (0.6 mmol) and **cat.1-6** (5 mol%) at 50°C for 0.5 h. Solvent (2 mL) and **2a** was added, and the mixture was stirred at rt for 24 h. [b]Yield was determined by HPLC analysis. [c]Determined by HPLC (Chiralcel OD-RH).

Supplementary Table 2. Additional efforts at reaction optimization (solvent, temperature, additive, catalyst loading, concentration and activating reagents)[a]

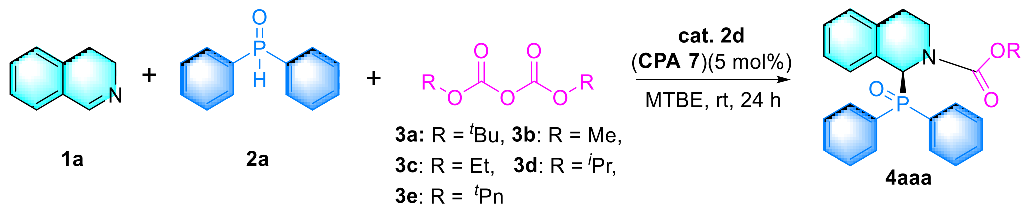

Entry	Solvent	additive	Temp. (°C)	Yield (%)[b]	ee (%)[c]
1	PhMe	-	25	90	44
2	PhH	-	25	81	63
3	PhCl	-	25	52	27
4	$PhNO_2$	-	25	78	5
5	PhOMe	-	25	77	24
6	PhOEt	-	25	80	36
7	CCl_4	-	25	99	2
8	CH_2Cl_2	-	25	92	16
9	EA	-	25	99	21
10	CH_3CN	-	25	99	2
11	Acetone	-	25	22	16
12	Et_2O	-	25	35	14
13	*n*-Pr_2O	-	25	95	56
14	*i*-Pr_2O	-	25	98	47
15	*n*-Bu_2O	-	25	96	67
16	MTBE	-	25	96	79
17	THF	-	25	95	23
18	2-MeTHF	-	25	82	46
19	1,4-Dioxane	-	25	57	12
20	MTBE	3Å MS	25	99	88
21	MTBE	4Å MS	25	99	91
22	MTBE	5Å MS	25	99	85
23	MTBE	13X MS	25	99	88
24	MTBE	4Å MS	0	70(99[d])	91
25[e]	MTBE	4Å MS	35	99	85
26[f]	MTBE	4Å MS	25	60	90
27[g]	MTBE	4Å MS	25	99	91
26[h]	MTBE	4Å MS	25	73	91
27[i]	MTBE	4Å MS	25	99	89
28	PhH	4Å MS	25	99	90
29[j]	MTBE	4Å MS	25	95	30
30[k]	MTBE	4Å MS	25	80	40
31[l]	MTBE	4Å MS	25	87	73
32[m]	MTBE	4Å MS	25	99	80

[a]Reaction conditions: **1a** (0.2 mmol), 3 (0.3 mmol) and **cat.2d** (5 mol%) at 50°C for 0.5 h. Solvent (2 mL) and **2a** was added, and the mixture was stirred at rt for 24 h. [b]Yield was determined by HPLC analysis. [c]Determined by HPLC (Chiralcel OD-RH). [d]48 h. [e]12h. [f]**cat.2d** (2.5 mol%). [g]**cat.2d** (10 mol%), 12h. [h]0.05M. [i]0.2M. [j]**3b** was used. [k]**3c** was used. [l]**3d** was used. [m]**3e** was used.

3. Control and NMR tracking experiments

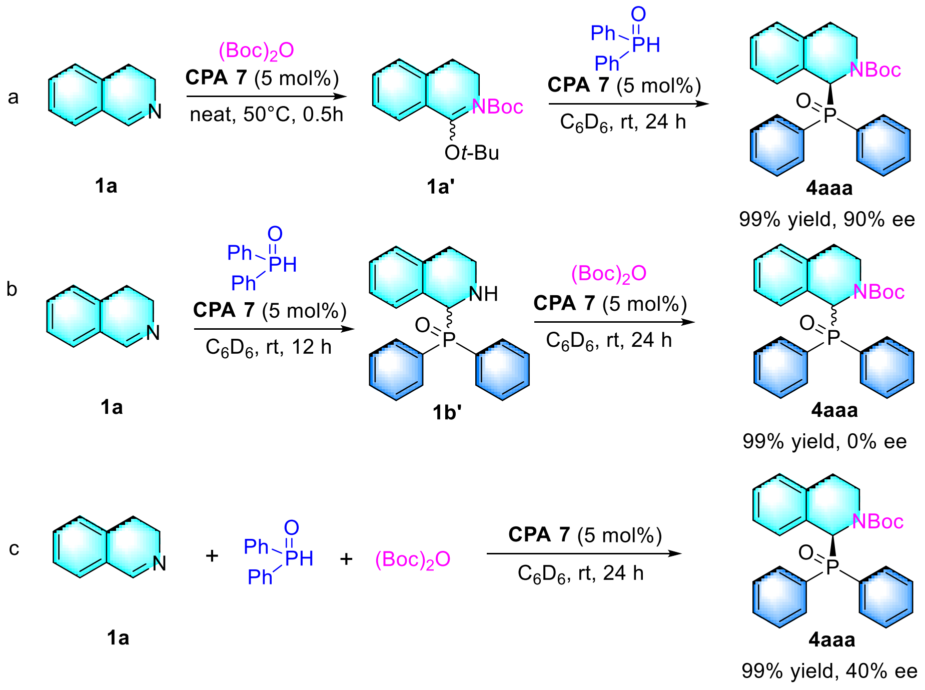

Supplementary Figure 1. Control experiments

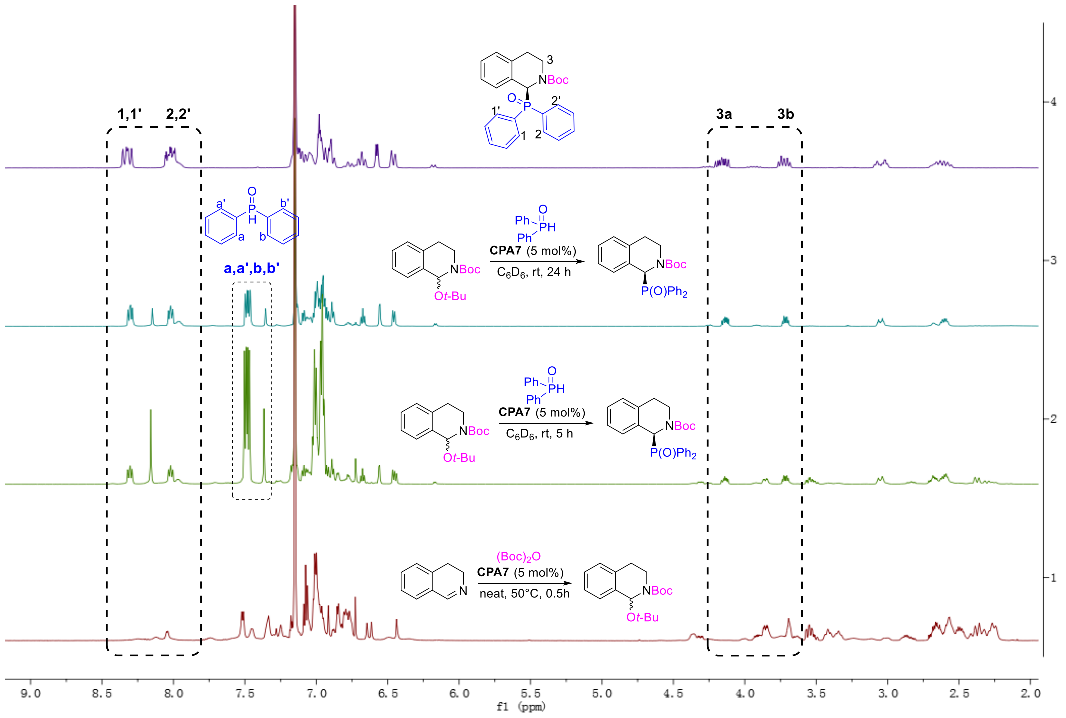

Supplementary Figure 2 ^{1}H NMR of mixture at different reaction processes and times

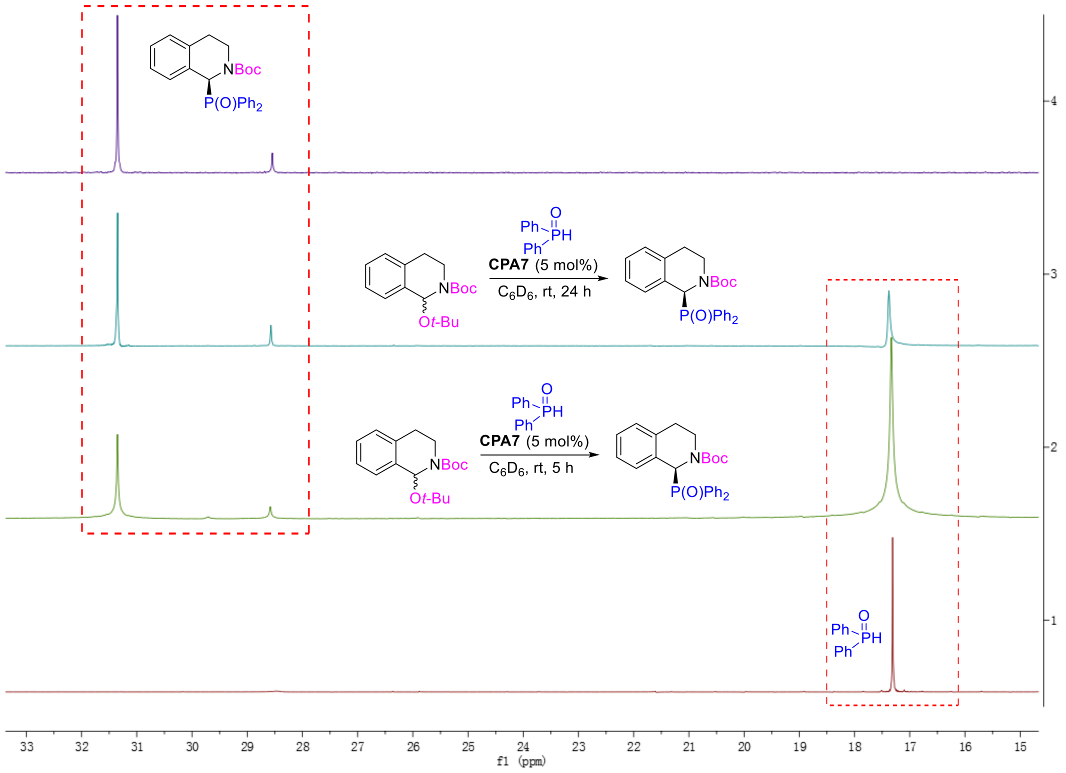

Supplementary Figure 3. ^{31}P NMR of mixture at different reaction processes and times

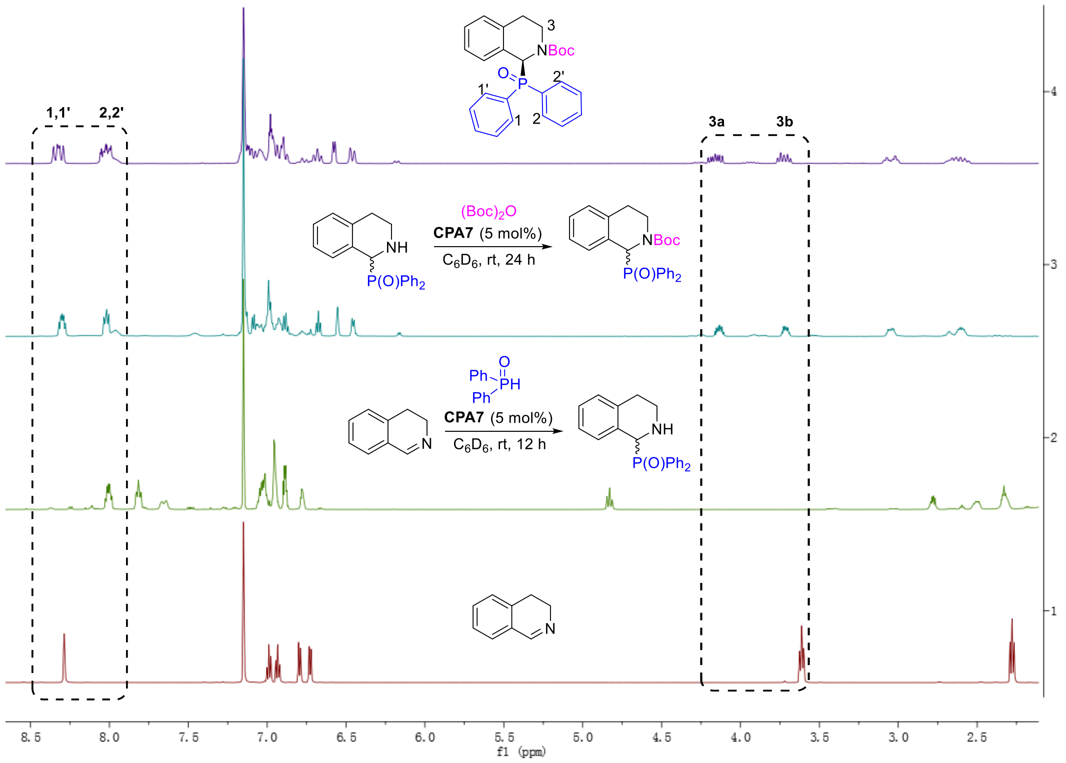

Supplementary Figure 4. ^{1}H NMR of mixture at different reaction processes and times

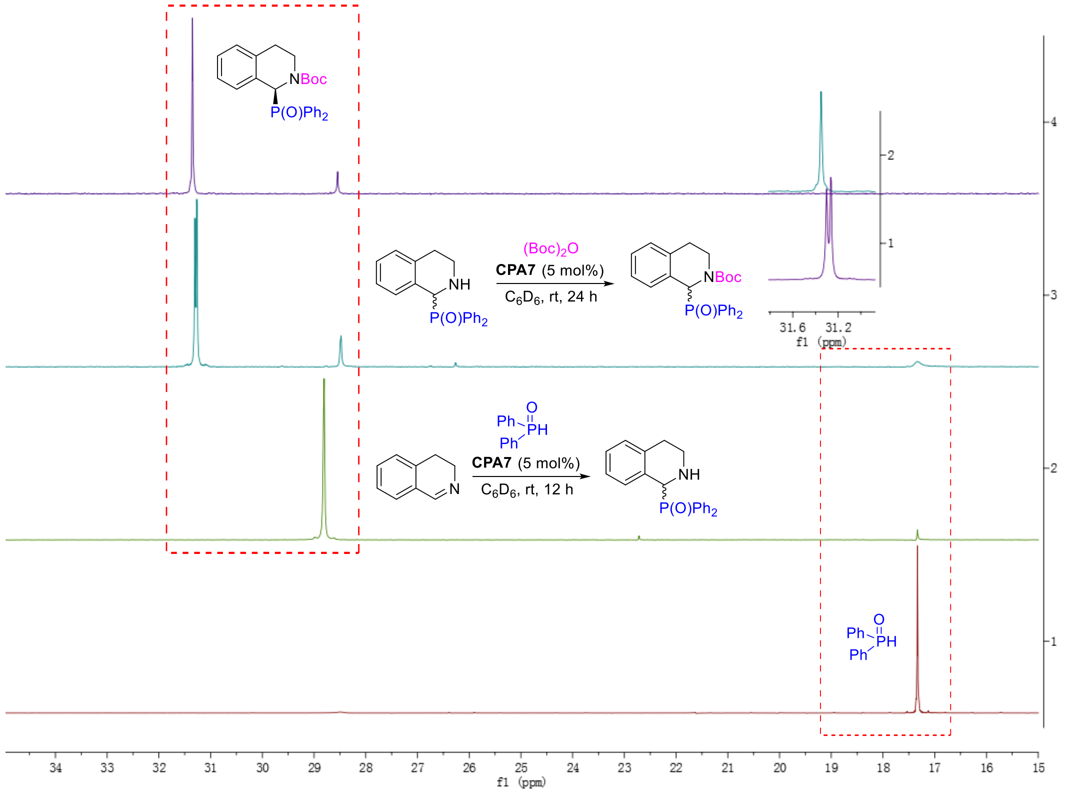

Supplementary Figure 5. ^{31}P NMR of mixture at different reaction processes and times

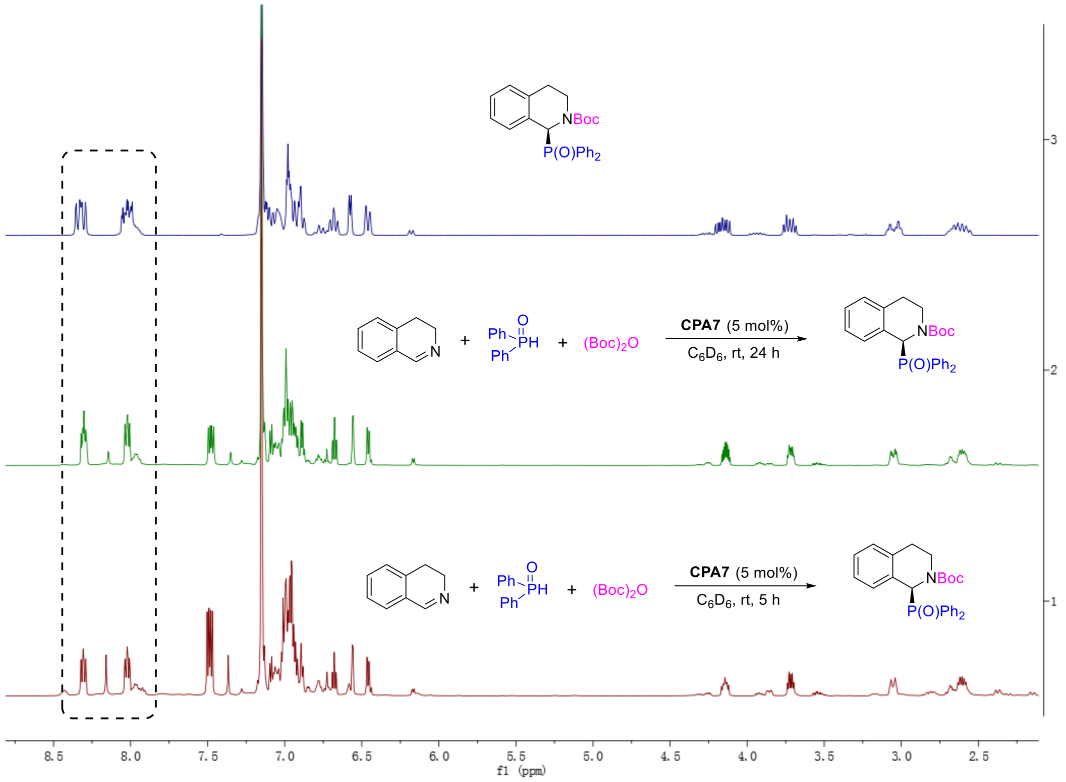

Supplementary Figure 6. ^{1}H NMR of mixture at different reaction processes and times

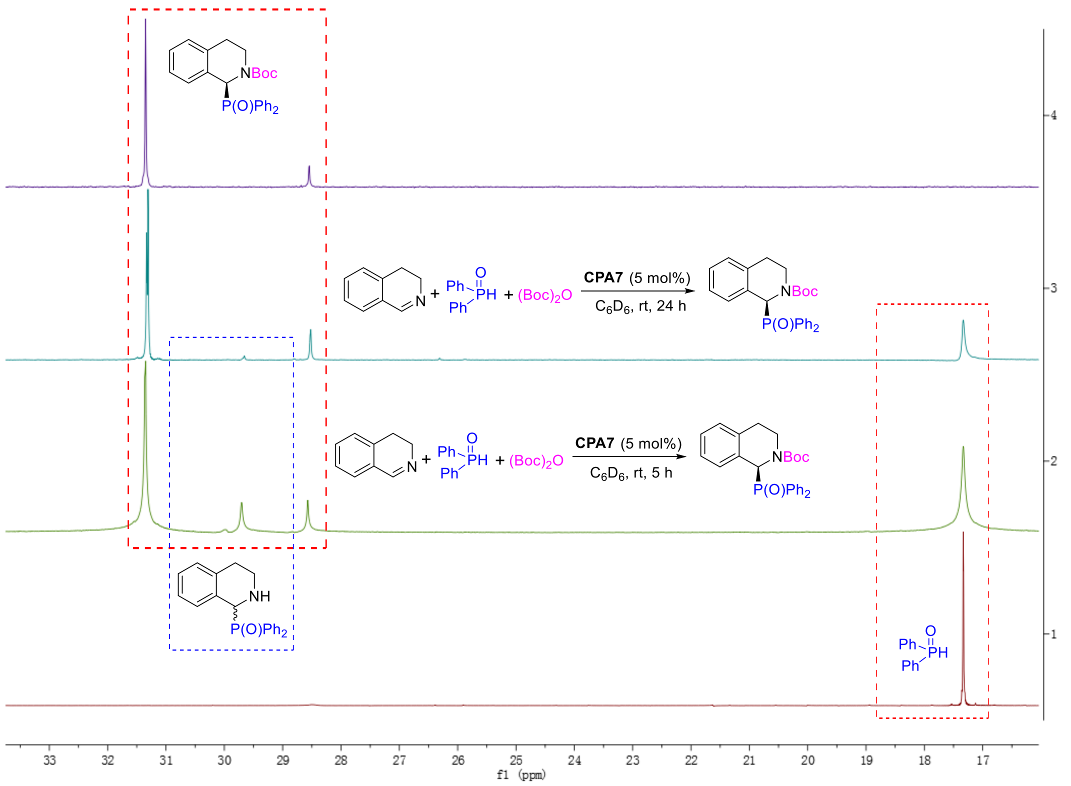

Supplementary Figure 7 ^{31}P NMR of mixture at different reaction processes and times

4. Supplementary Notes:

General Procedure for Synthesis of *tert*-butyl-1-(diarylphosphoryl)-3,4-dihydroisoquinoline-2(1*H*)-carboxylates.

A mixture of 3,4-dihydroisoquinoline **1** (0.2 mmol), catalyst **CPA7** (5 mol%) and $(Boc)_2O$ (0.3mmol) was stirred at 50°C for 0.5 h. Then 4Å MS (50 mg), MTBE or benzene (2 mL) and diarylphosphine oxide **2** (0.24mmol) was added, and the reaction was stirred at room temperature for 24 h. The reaction mixture was concentrated under reduced pressure. The residue was purified by flash column chromatography with PE/EA (2/1) to obtain tert-butyl-1-(diphenylphosphoryl)-3,4- dihydroisoquinoline-2(1H)-carboxylates **4**.

5. Characterization of products

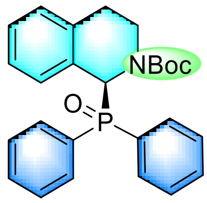

4aaa

99% yield, 91% ee

^{1}H NMR (600 MHz, C_6D_6): δ 8.37 - 8.24 (m, 2H), 8.02 (ddd, *J* = 10.5, 7.8, 1.6 Hz, 2H), 7.76 - 7.73 (m, 2H), 7.11 - 7.02 (m, 2H), 7.01 - 6.95 (m, 2H), 6.91 (dd, *J* = 21.5, 7.4 Hz, 2H), 6.68 (t, *J* = 7.3 Hz, 1H), 6.57 (d, *J* = 3.6 Hz, 1H), 6.46 (d, *J* = 7.7 Hz, 1H), 4.14 (ddd, *J* = 12.9, 8.9, 5.3 Hz, 1H), 3.77 - 3.67 (m, 1H), 3.05 (ddd, *J* = 15.9, 5.0, 3.4 Hz, 1H), 2.64 - 2.53 (m, 1H), 1.25 (s, 9H).

^{13}C NMR (151 MHz, C_6D_6): δ 155.26 (d, *J* = 3.3 Hz), 137.33 (d, *J* = 3.9 Hz), 133.85 (d, *J* = 18.4 Hz), 133.23 (d, *J* = 24.4 Hz), 132.49, 132.45, 132.43, 132.39, 132.03 (d, *J* = 2.7 Hz), 131.85

(d, J = 3.0 Hz), 131.22, 129.68 (d, J = 2.6 Hz), 129.06 (d, J = 11.2 Hz), 128.60, 128.47, 128.31, 128.01 (d, J = 3.3 Hz), 127.93 (d, J = 2.8 Hz), 126.10 (d, J = 2.6 Hz), 80.2, 57.3 (d, J = 73.9 Hz), 41.8, 28.8, 28.7 (3).

^{31}P NMR (243 MHz, C_6D_6): δ 31.5 (for major rotamer), 28.6 (for minor rotamer).

HRMS (m/z) calcd for $C_{26}H_{28}NO_3PNa$ $[M+Na]^+$ 456.1699, found 456.1704; $[\alpha]_D^{25}$ = + 6.1 (c = 0.20, $CHCl_3$); The *ee* was determined by HPLC analysis (Chiralpak OD-RH, CH_3CN/H_2O = 60/40, v/v, 1.0 mL/min, 280 nm, t_R (major) = 17.4 min, t_R (minor) = 19.1 min), 91% *ee*.

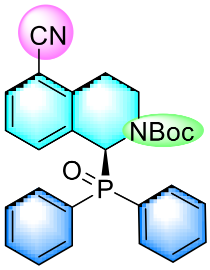

4baa
86% yield, 92% ee

^{1}H NMR (600 MHz, C_6D_6): δ 8.27 - 8.18 (m, 2H), 7.97 - 7.90 (m, 2H), 7.12 (m, 2H), 7.09 - 7.04 (m, 1H), 7.03 - 6.92 (m, 3H), 6.71 (s, 1H), 6.49 (d, J = 7.9 Hz, 1H), 6.38 (d, J = 4.7 Hz, 1H), 6.10 (d, J = 7.2 Hz, 1H), 3.98 (ddd, J = 13.2, 9.1, 5.3 Hz, 1H), 3.66 - 3.54 (m, 1H), 2.71 (d, J = 15.3 Hz, 1H), 2.32 - 2.23 (m, 1H), 1.23 (s, 9H).

^{13}C NMR (151 MHz, C_6D_6): δ 155.00 (d, J = 3.2 Hz), 138.29 (d, J = 3.7 Hz), 136.03, 133.24, 133.08 (d, J = 1.8 Hz), 132.95, 132.64, 132.55 (d, J = 2.8 Hz), 132.38, 132.33 (d, J = 9.5 Hz), 132.20 (d, J = 8.7 Hz), 129.25 (d, J = 11.3 Hz), 128.76 (d, J = 11.7 Hz). 129.25 (d, J = 2.3 Hz), 128.68, 128.47, 128.31, 119.01, 112.17 (d, J = 2.7 Hz), 80.69, 57.13 (d, J = 72.0 Hz), 41.00, 28.52 (3), 28.18.

^{31}P NMR (243 MHz, C_6D_6): δ 31.7 (for major rotamer), 28.9 (for minor rotamer).

HRMS (m/z) calcd for $C_{27}H_{27}N_2O_3PNa$ $[M+Na]^+$ 481.1652, found:481.1659; $[\alpha]_D^{25}$ = + 15.5 (c = 0.20, $CHCl_3$); The *ee* was determined by HPLC analysis (Chiralpak OJ-RH, CH_3CN/H_2O = 65/35, v/v, 1.0 mL/min, 280 nm, t_R (major) = 13.0 min, t_R (minor) = 15.0 min), 92% *ee*.

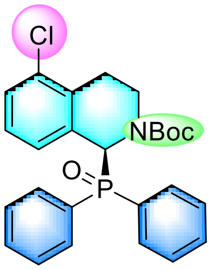

4caa
83% yield, 95% ee

^{1}H NMR (600 MHz, C_6D_6) δ: 8.41 - 8.20 (m, 2H), 8.00 (ddd, J = 10.6, 7.8, 1.5 Hz, 2H), 7.14 - 7.11 (m, 2H), 7.10 - 7.04 (m, 1H), 7.04 - 6.95 (m, 4H), 6.51 (d, J = 4.2 Hz, 1H), 6.39 (t, J = 7.8 Hz, 1H), 6.27 (d, J = 7.7 Hz, 1H), 4.11 (ddd, J = 13.4, 10.3, 5.1 Hz, 1H), 3.90 - 3.72 (m, 1H), 3.11 - 2.96 (m, 1H), 2.76 - 2.64 (m, 1H), 1.23 (s, 9H).

^{13}C NMR (151 MHz, C_6D_6): δ 154.97 (d, J = 3.7 Hz), 135.39 (d, J = 2.2 Hz), 135.05 (d, J = 3.8 Hz), 134.00, 133.38(d, J = 4.6 Hz), 133.37, 132.73, 132.34 (d, J = 7.2 Hz), 132.28 (d, J = 6.6 Hz),

132.15 (d, J = 2.6 Hz), 132.01 (d, J = 2.6 Hz), 129.17 (d, J = 11.2 Hz), 128.75 (d, J = 8.4 Hz), 128.68, 128.47, 128.31, 126.81 (d, J = 2.4 Hz), 126.42 (d, J = 2.9 Hz), 80.44, 56.78 (d, J = 73.1 Hz), 40.81, 28.54 (3), 26.76.

^{31}P NMR (243 MHz, C_6D_6): δ 31.6 (for major rotamer), 28.7 (for minor rotamer).

HRMS (m/z) calcd for $C_{26}H_{27}ClNO_3PNa$ $[M+Na]^+$ 490.1309, found 490.1316; $[\alpha]_D^{25}$ = + 36.5 (c = 0.20, $CHCl_3$); The *ee* was determined by HPLC analysis (Chiralpak OJ-RH, CH_3CN/H_2O = 55/45, v/v, 1.0 mL/min, 280 nm, t_R (major) = 8.1 min, t_R (minor) = 9.2 min), 95% *ee*.

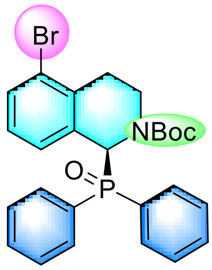

4daa

91% yield, 97% ee

^{1}H NMR (600 MHz, C_6D_6) δ 8.37 – 8.18 (m, 2H), 8.00 (ddd, J = 10.6, 7.8, 1.5 Hz, 2H), 7.18 (dt, J = 7.6, 2.2 Hz, 1H), 7.14 – 7.10 (m, 2H), 7.09 – 6.94 (m, 4H), 6.49 (d, J = 4.2 Hz, 1H), 6.30 (d, J = 5.2 Hz, 2H), 4.09 (ddd, J = 13.4, 10.2, 5.1 Hz, 1H), 3.83 – 3.69 (m, 1H), 3.12 – 2.96 (m, 1H), 2.81 – 2.64 (m, 1H), 1.23 (s, 9H).

^{13}C NMR (151 MHz, C_6D_6): δ 154.95 (d, J = 3.6 Hz), 136.63 (d, J = 3.8 Hz), 133.97, 133.60, 133.36 (d, J = 4.3 Hz), 132.71, 132.34 (d, J = 7.9 Hz), 132.29 (d, J = 7.3 Hz), 132.15 (d, J = 2.6 Hz), 132.06 (d, J = 2.6 Hz), 132.01 (d, J = 2.6 Hz), 129.17 (d, J = 11.3 Hz), 128.73 (d, J = 11.3 Hz), 128.68, 128.47, 128.31, 127.20 (d, J = 2.3 Hz), 127.07 (d, J = 2.9 Hz), 126.36 (d, J = 2.2 Hz), 80.45, 56.91 (d, J = 73.1 Hz), 41.05, 29.69, 28.54 (3).

^{31}P NMR (243 MHz, C_6D_6): δ 31.7 (for major rotamer), 28.8 (for minor rotamer).

HRMS (m/z) calcd for $C_{26}H_{27}ClNO_3PNa$ $[M+Na]^+$ 534.0804, found 534.0808; $[\alpha]_D^{25}$ = + 50.2 (c = 0.20, $CHCl_3$); The *ee* was determined by HPLC analysis (Chiralpak OJ-RH, CH_3CN/H_2O = 65/35, v/v, 1.0 mL/min, 280 nm, t_R (major) = 33.6 min, t_R (minor) = 36.6 min), 97% *ee*.

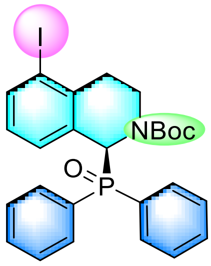

4eaa

83% yield, 95% ee

^{1}H NMR (600 MHz, C_6D_6): δ 8.36 – 8.21 (m, 2H), 8.07 – 7.95 (m, 2H), 7.47 (d, J = 7.9 Hz, 1H), 7.14 – 7.10 (m, 2H), 7.09 – 7.03 (m, 1H), 6.99 (tt, J = 8.7, 6.1 Hz, 3H), 6.45 (d, J = 4.0 Hz, 1H), 6.33 (d, J = 7.6 Hz, 1H), 6.14 (t, J = 7.7 Hz, 1H), 4.07 (ddd, J = 13.4, 10.0, 5.2 Hz, 1H), 3.74 (ddd, J = 10.4, 5.6, 3.7 Hz, 1H), 3.02 (d, J = 16.6 Hz, 1H), 2.70 (dt, J = 16.4, 7.2 Hz, 1H), 1.23 (s, 9H).

^{13}C NMR (151 MHz, C_6D_6): δ 154.92 (d, J = 3.6 Hz), 139.63 (d, J = 3.8 Hz), 138.82 (d, J = 2.6 Hz), 133.97, 133.37, 133.22, 132.73, 132.35 (d, J = 8.8 Hz), 132.30 (d, J = 7.8 Hz), 132.13 (d, J =

2.6 Hz), 131.99 (d, J = 2.6 Hz), 129.16 (d, J = 11.2 Hz), 128.72 (d, J = 12.0 Hz), 128.68, 128.47, 128.31, 128.00 (d, J = 2.9 Hz), 127.61 (d, J = 2.2 Hz), 103.36 (d, J = 2.2 Hz), 80.44, 57.12 (d, J = 73.0 Hz), 41.58, 35.17, 28.54 (3).

^{31}P NMR (243 MHz, C_6D_6): δ 31.6 (for major rotamer), 28.6 (for minor rotamer).

HRMS (m/z) calcd for $C_{26}H_{27}INO_3PNa$ $[M+Na]^+$ 582.0665, found 582.0669; $[\alpha]_D^{25}$ = + 53.4 (c = 0.20, $CHCl_3$); The *ee* was determined by HPLC analysis (Chiralpak AS-RH, CH_3CN/H_2O = 50/50, v/v, 1.0 mL/min, 280 nm, t_R (major) = 9.8 min, t_R (minor) = 11.2 min), 95% *ee*.

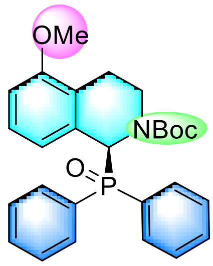

4faa
92% yield, 89% ee

^{1}H NMR (600 MHz, C_6D_6): δ 8.37 – 8.29 (m, 2H), 8.11 – 8.04 (m, 2H), 7.15 – 7.11 (m, 1H), 7.10 – 7.03 (m, 2H), 7.01 (dt, J = 7.6, 3.5 Hz, 3H), 6.68 (t, J = 8.0 Hz, 1H), 6.64 (d, J = 4.2 Hz, 1H), 6.29 (d, J = 8.3 Hz, 1H), 6.19 (d, J = 7.8 Hz, 1H), 4.28 – 4.19 (m, 1H), 4.00 – 3.89 (m, 1H), 3.23 (s, 3H), 3.15 – 3.07 (m, 1H), 2.89 – 2.81 (m, 1H), 1.23 (s, 9H).

^{13}C NMR (151 MHz, C_6D_6): δ 158.02 (d, J = 2.1 Hz), 155.21 (d, J = 3.9 Hz), 134.51, 133.92, 133.28, 132.40 (d, J = 2.6 Hz), 132.34 (d, J = 3.1 Hz), 132.14, 131.98 (d, J = 2.7 Hz), 131.83 (d, J = 2.6 Hz), 129.10 (d, J = 11.2 Hz), 128.68 (d, J = 11.4 Hz), 128.68, 128.47, 128.31, 126.48 (d, J = 2.4 Hz), 125.89 (d, J = 3.9 Hz), 120.29 (d, J = 2.8 Hz), 109.24 (d, J = 2.6 Hz), 80.12, 56.76 (d, J = 73.8 Hz), 55.12, 41.06, 28.58 (3), 23.01.

^{31}P NMR (243 MHz, C_6D_6): δ 31.5 (for major rotamer), 28.7 (for minor rotamer).

HRMS (m/z) calcd for $C_{27}H_{30}NO_4PNa$ $[M+Na]^+$ 486.1805, found 486.1810; $[\alpha]_D^{25}$ = + 34.8 (c = 0.20, $CHCl_3$); The *ee* was determined by HPLC analysis (Chiralpak AS-RH, CH_3CN/H_2O = 50/50, v/v, 1.0 mL/min, 280 nm, t_R (major) = 5.4 min, t_R (minor) = 6.6 min), 89% *ee*.
C27H30NO4P+Na,Clac:486.1805, Found:486.1810

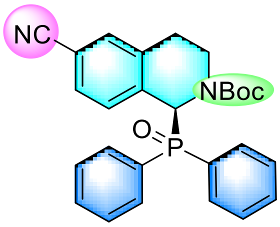

4gaa
90% yield, 89% ee

^{1}H NMR (600 MHz, Benzene-d_6) δ 8.21 (ddt, J = 11.3, 6.1, 1.4 Hz, 2H), 7.98 (ddt, J = 9.6, 6.3, 1.7 Hz, 2H), 7.11 (ddd, J = 9.1, 5.3, 2.3 Hz, 2H), 7.09 – 7.04 (m, 1H), 7.03 – 6.95 (m, 3H), 6.84 (dt, J = 7.7, 1.5 Hz, 1H), 6.39 (d, J = 4.4 Hz, 1H), 6.35 (d, J = 7.8 Hz, 1H), 6.24 (t, J = 7.7 Hz, 1H), 3.90 (ddd, J = 13.2, 9.8, 5.1 Hz, 1H), 3.60 (dddd, J = 13.3, 6.5, 3.9, 1.2 Hz, 1H), 3.19 – 3.05 (m, 1H), 2.72 (m, 1H), 1.22 (s, 9H).

^{13}C NMR (151 MHz, C_6D_6): δ 154.87 (d, J = 3.1 Hz), 140.64 (d, J = 3.8 Hz), 133.66, 132.99(d,

J = 18.9 Hz), 132.68, 132.32, 132.21 (d, J =1.8 Hz), 132.26, 132.19, 132.14(d, J = 1.2 Hz), 131.90 (d, J = 2.3 Hz), 131.45 (d, J = 2.9 Hz), 129.25 (d, J = 11.3 Hz), 128.76 (d, J = 11.7 Hz), 128.68, 128.47, 128.31, 126.13 (d, J = 2.1 Hz), 117.76, 114.23 (d, J = 1.9 Hz), 80.66, 56.69 (d, J = 72.5 Hz), 40.48, 28.51 (3), 27.55.

^{31}P NMR (243 MHz, C_6D_6): δ 31.5 (for major rotamer), 28.6 (for minor rotamer).

HRMS (m/z) calcd for $C_{27}H_{27}NO_3PNa$ $[M+Na]^+$ 481.1652, found 481.1658; $[\alpha]_D^{25}$ = + 26.6 (c = 0.20, $CHCl_3$); The *ee* was determined by HPLC analysis (Chiralpak OD-RH, CH_3CN/H_2O = 68/32, v/v, 1.0 mL/min, 280 nm, t_R (major) = 65.5 min, t_R (minor) = 70.6 min), 89% *ee*.

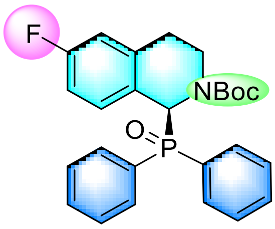

4haa
93% yield, 88% ee

^{1}H NMR (600 MHz, C_6D_6): δ 8.47 – 8.19 (m, 2H), 8.10 – 7.77 (m, 2H), 7.15 – 7.10 (m, 1H), 7.10 – 7.01 (m, 2H), 7.01 – 6.92 (m, 3H), 6.57 (dd, J = 9.2, 2.3 Hz, 1H), 6.46 (d, J = 2.9 Hz, 1H), 6.32 (td, J = 8.5, 2.5 Hz, 1H), 6.27 – 6.20 (m, 1H), 4.07 (ddd, J = 13.1, 8.9, 5.3 Hz, 1H), 3.71 – 3.57 (m, 1H), 2.90 (dt, J = 16.0, 4.2 Hz, 1H), 2.47 – 2.38 (m, 1H), 1.25 (s, 9H).

^{13}C NMR (151 MHz, C_6D_6): δ 162.74 (dd, J_{C-F} = 245.7, J_{C-P} = 2.9 Hz), 155.18 (d, J = 3.5 Hz), 139.78 (dd, J_{C-F} = 7.8, J_{C-P} = 3.7 Hz), 133.61 (d, J_{C-F} = 29.1 Hz), 133.11, 132.87, 132.42 (d, J = 9.3 Hz), 132.33 (d, J = 8.6 Hz), 132.12 (d, J = 2.6 Hz), 131.95 (d, J = 2.7 Hz), 129.45 (dd, J_{C-F} = 8.2, J_{C-P} = 3.2 Hz), 129.11 (d, J = 11.1 Hz), 128.70 (d, J = 11.1 Hz), 128.68, 128.47, 128.31, 126.86 (d, J = 2.7 Hz), 116.31 (d, J = 21.3 Hz), 113.39 – 112.70 (m), 80.36, 56.66 (d, J = 74.4 Hz), 41.22, 28.72, 28.57 (3).

^{31}P NMR (243 MHz, C_6D_6): δ 31.2 (d, J_{P-F} = 4.3 Hz), 28.4 (d, J_{P-F} = 3.9 Hz).

HRMS (m/z) calcd for $C_{26}H_{27}FNO_3PNa$ $[M+Na]^+$ 474.1605, found 474.1613; $[\alpha]_D^{25}$ = + 18.4(c = 0.20, $CHCl_3$); The *ee* was determined by HPLC analysis (Chiralpak OJ-RH, CH_3CN/H_2O = 50/50 v/v, 1.0 mL/min, 280 nm, t_R (major) = 4.0 min, t_R (minor) = 6.1 min), 88% *ee*.

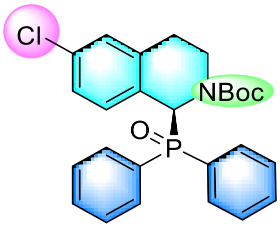

4iaa
91% yield, 93% ee

^{1}H NMR (600 MHz, C_6D_6) δ 8.31 – 8.20 (m, 2H), 7.96 (ddd, J = 10.6, 7.8, 1.5 Hz, 2H), 7.14 – 7.10 (m, 1H), 7.10 – 7.00 (m, 2H), 7.00 – 6.94 (m, 3H), 6.88 (s, 1H), 6.60 (dd, J = 8.2, 1.9 Hz, 1H), 6.42 (d, J = 3.4 Hz, 1H), 6.16 (dd, J = 8.2, 1.3 Hz, 1H), 4.02 (ddd, J = 13.1, 9.1, 5.3 Hz, 1H), 3.68 – 3.60 (m, 1H), 2.88 – 2.80 (m, 1H), 2.44 – 2.36 (m, 1H), 1.24 (s, 9H).

^{13}C NMR (151 MHz, C_6D_6) δ 155.14 (d, J = 3.3 Hz), 139.25 (d, J = 3.8 Hz), 133.79 (d, J = 3.3 Hz), 133.11, 132.90, 132.38 (d, J = 9.7 Hz), 132.38 – 132.27 (m), 132.28 (d, J = 8.0 Hz), 132.11

(d, J = 2.6 Hz), 129.80 (d, J = 1.8 Hz), 129.39, 129.20 (d, J = 11.3 Hz), 129.12 (d, J = 3.2 Hz), 128.75 (d, J = 11.7 Hz), 128.68, 128.47, 128.31, 126.28 (d, J = 2.4 Hz), 104.67 (d, J = 2.8 Hz), 80.51, 56.71 (d, J = 73.8 Hz), 41.18, 28.54 (3), 28.45.

^{31}P NMR (243 MHz, C_6D_6): δ 32.4 (for major rotamer), 29.4 (for minor rotamer).

HRMS (m/z) calcd for $C_{26}H_{27}ClNO_3PNa$ $[M+Na]^+$ 490.1309, found 490.1313; $[\alpha]_D^{25}$ = + 20.5 (c = 0.20, $CHCl_3$); The *ee* was determined by HPLC analysis (Chiralpak OJ-RH, CH_3CN/H_2O = 50/50 v/v, 1.0 mL/min, 280 nm, t_R (major) = 5.1 min, t_R (minor) = 8.1 min), 93% *ee*.

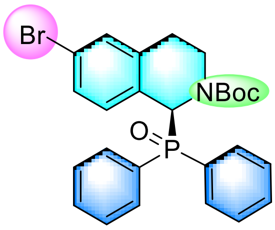

4jaa
93% yield, 93% ee

^{1}H NMR (600 MHz, C_6D_6): δ 8.32 – 8.20 (m, 2H), 8.02 – 7.80 (m, 2H), 7.14 – 7.11 (m, 1H), 7.08 (td, J = 7.4, 1.2 Hz, 1H), 7.02 (s, 2H), 7.00 – 6.92 (m, 3H), 6.75 (dd, J = 8.2, 1.6 Hz, 1H), 6.41 (d, J = 3.8 Hz, 1H), 6.09 (dd, J = 8.2, 1.3 Hz, 1H), 4.06 (ddd, J = 13.0, 9.1, 5.3 Hz, 1H), 3.68 – 3.58 (m, 1H), 2.89 – 2.76 (m, 1H), 2.43 – 2.31 (m, 1H), 1.24 (s, 9H).

^{13}C NMR (151 MHz, C_6D_6): δ 155.15 (d, J = 3.5 Hz), 139.65 (d, J = 3.8 Hz), 133.48 (d, J = 26.5 Hz), 132.97, 132.75, 132.68 (d, J = 1.9 Hz), 132.39 (d, J = 9.3 Hz), 132.30 (d, J = 8.7 Hz), 132.16 (d, J = 2.6 Hz), 131.99 (d, J = 2.6 Hz), 130.19, 129.43 (d, J = 3.2 Hz), 129.13 (d, J = 2.3 Hz), 129.12 (d, J = 11.2 Hz), 128.71 (d, J = 8.8 Hz), 128.68, 128.47 , 128.31, 121.94 (d, J = 3.4 Hz), 80.42, 56.76 (d, J = 73.7 Hz), 41.19, 28.56 (3), 28.40.

^{31}P NMR (243 MHz, C_6D_6): δ 31.1 (for major rotamer), 28.3 (for minor rotamer).

HRMS (m/z) calcd for $C_{26}H_{27}BrNO_3PNa$ $[M+Na]^+$ 534.0804, found 534.0806; $[\alpha]_D^{25}$ = + 24.5 (c = 0.20, $CHCl_3$); The *ee* was determined by HPLC analysis (Chiralpak OJ-RH, CH_3CN/H_2O = 55/45 v/v, 1.0 mL/min, 280 nm, t_R (major) = 8.5 min, t_R (minor) = 12.6 min), 93% *ee*.

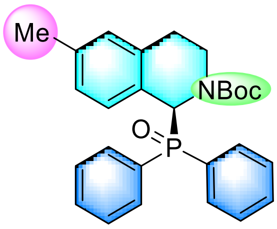

4kaa
99% yield, 79% ee

^{1}H NMR (600 MHz, C_6D_6) δ 8.38 – 8.27 (m, 2H), 8.03 (ddd, J = 10.5, 7.7, 1.6 Hz, 2H), 7.20 – 7.13 (m, 1H), 7.11 – 7.03 (m, 2H), 7.03 – 6.95 (m, 3H), 6.70 (s, 1H), 6.57 (d, J = 3.2 Hz, 1H), 6.50 (d, J = 7.7 Hz, 1H), 6.37 (dd, J = 7.8, 1.3 Hz, 1H), 4.21 (ddd, J = 12.9, 9.0, 5.3 Hz, 1H), 3.83 – 3.71 (m, 1H), 3.06 (ddd, J = 15.9, 4.9, 3.3 Hz, 1H), 2.68 – 2.57 (m, 1H), 2.00 (s, 3H), 1.26 (s, 9H).

^{13}C NMR (151 MHz, C_6D_6) δ 155.32 (d, J = 3.7 Hz), 137.34 (d, J = 2.8 Hz), 137.13 (d, J = 3.8 Hz), 134.13, 133.98, 133.53, 133.35, 132.48 (d, J = 3.8 Hz), 132.42 (d, J = 3.2 Hz), 131.95 (d, J = 2.6 Hz), 131.80 (d, J = 2.6 Hz), 130.41 (d, J = 2.0 Hz), 129.03 (d, J = 11.1 Hz), 128.67, 128.47, 128.31, 128.12, 127.88 (d, J = 3.3 Hz), 126.93 (d, J = 2.3 Hz), 80.14, 57.01 (d, J = 74.5 Hz), 41.80,

28.81, 28.61 (3), 21.37.

^{31}P NMR (243 MHz, C_6D_6): δ 31.1 (for major rotamer), 28.4 (for minor rotamer).

HRMS (m/z) calcd for $C_{27}H_{30}NO_3PNa$ $[M+Na]^+$ 470.1856, found 470.1862; $[\alpha]_D^{25}$ = + 12.9 (c = 0.20, $CHCl_3$); The *ee* was determined by HPLC analysis (Chiralpak OJ-RH, CH_3CN/H_2O = 50/50 v/v, 1.0 mL/min, 280 nm, t_R (major) = 4.1 min, t_R (minor) = 5.4 min), 79% *ee*.

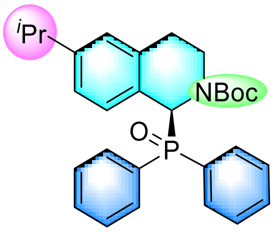

4laa
86% yield, 42% ee

^{1}H NMR (600 MHz, C_6D_6) δ 8.38 – 8.27 (m, 2H), 8.04 (ddd, *J* = 10.5, 7.8, 1.5 Hz, 2H), 7.19 – 7.12 (m, 1H), 7.12 – 7.03 (m, 2H), 7.02 – 6.96 (m, 3H), 6.87 (s, 1H), 6.59 (dd, *J* = 9.6, 2.1 Hz, 2H), 6.45 (dd, *J* = 7.9, 1.4 Hz, 1H), 4.24 – 4.15 (m, 1H), 3.83 – 3.71 (m, 1H), 3.08 (ddd, *J* = 15.9, 4.9, 3.2 Hz, 1H), 2.71 – 2.63 (m, 1H), 2.58 (ddt, *J* = 20.3, 13.6, 6.9 Hz, 1H), 1.26 (s, 9H), 1.11 – 1.04 (m, 6H).

^{13}C NMR (151 MHz, C_6D_6) δ 155.30 (d, *J* = 3.5 Hz), 148.37 (d, *J* = 2.8 Hz), 137.12 (d, *J* = 3.8 Hz), 134.08, 133.96, 133.49, 133.33, 132.48 (d, *J* = 4.2 Hz), 132.42 (d, *J* = 4.9 Hz), 131.97 (d, *J* = 2.6 Hz), 131.80 (d, *J* = 2.7 Hz), 129.05 (d, *J* = 11.1 Hz), 128.67, 128.65 (d, *J* = 11.1 Hz), 128.47, 128.31, 128.01 (d, *J* = 3.2 Hz), 127.78 (d, *J* = 1.8 Hz), 124.38 (d, *J* = 2.3 Hz), 80.15, 57.00 (d, *J* = 74.5 Hz), 41.82, 34.41, 28.98, 28.62 (3), 24.39, 24.32.

^{31}P NMR (243 MHz, C_6D_6): δ 31.3 (for major rotamer), 28.6 (for minor rotamer).

HRMS (m/z) calcd for $C_{29}H_{34}NO_3PNa$ $[M+Na]^+$ 498.2169, found 498.2175; $[\alpha]_D^{25}$ = + 12.3 (c = 0.20, $CHCl_3$); The *ee* was determined by HPLC analysis (Chiralpak OJ-RH, CH_3CN/H_2O = 55/45 v/v, 1.0 mL/min, 280 nm, t_R (major) = 8.3 min, t_R (minor) = 9.6 min), 42% *ee*.

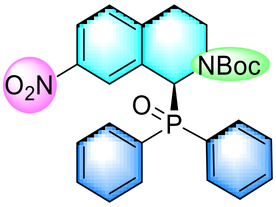

4maa
52% yield, 89% ee

^{1}H NMR (600 MHz, C_6D_6) δ 8.30 – 8.20 (m, 2H), 7.89 – 7.80 (m, 2H), 7.65 (d, *J* = 8.3 Hz, 1H), 7.14 – 7.11 (m, 1H), 7.11 – 7.02 (m, 4H), 6.99 (td, *J* = 7.5, 2.8 Hz, 2H), 6.48 (d, *J* = 8.4 Hz, 1H), 6.42 (d, *J* = 4.0 Hz, 1H), 4.19 (ddd, *J* = 13.2, 9.5, 5.2 Hz, 1H), 3.78 – 3.60 (m, 1H), 2.79 (d, *J* = 16.3 Hz, 1H), 2.46 – 2.36 (m, 1H), 1.25 (s, 9H).

^{13}C NMR (151 MHz, C_6D_6) δ 167.84, 154.95 (d, *J* = 3.5 Hz), 146.32, 144.35 (d, *J* = 3.5 Hz), 132.85 (d, *J* = 2.6 Hz), 132.42 (d, *J* = 9.4 Hz), 132.33, 132.22 (d, *J* = 2.6 Hz), 132.04 (d, *J* = 8.6 Hz), 131.11, 130.22, 129.49 (d, *J* = 11.4 Hz), 128.79 (d, *J* = 11.6 Hz), 128.68, 128.47, 128.31, 122.97 (d, *J* = 2.9 Hz), 122.41 (d, *J* = 2.3 Hz), 80.73, 56.67 (d, *J* = 73.0 Hz), 40.83, 28.75, 28.50 (3).

^{31}P NMR (243 MHz, C_6D_6): δ 31.8 (for major rotamer), 29.7 (for minor rotamer).

HRMS (m/z) calcd for $C_{26}H_{27}N_2O_5PNa$ $[M+Na]^+$ 501.1550, found 501.1556; $[\alpha]_D^{25}$ = + 12.7 (c = 0.20, $CHCl_3$); The *ee* was determined by HPLC analysis (Chiralpak OJ-RH, CH_3CN/H_2O = 70/30 v/v, 1.0 mL/min, 280 nm, t_R (major) = 41.3 min, t_R (minor) = 44.4 min), 89% *ee*.

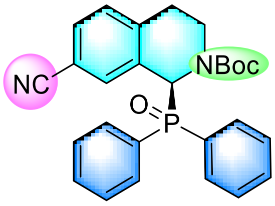

4naa
86% yield, 90% ee

^{1}H NMR (600 MHz, C_6D_6) δ 7.88 (dd, *J* = 11.4, 7.6 Hz, 2H), 7.81 (dd, *J* = 10.8, 7.7 Hz, 2H), 7.53 (q, *J* = 10.0, 8.7 Hz, 1H), 7.47 (td, *J* = 7.7, 2.7 Hz, 2H), 7.41 (d, *J* = 7.3 Hz, 1H), 7.36 (td, *J* = 7.6, 2.7 Hz, 2H), 7.31 (d, *J* = 8.0 Hz, 1H), 7.19 – 7.07 (m, 1H), 6.41 (s, 1H), 6.06 (d, *J* = 3.8 Hz, 1H), 3.84 – 3.66 (m, 2H), 3.11 – 3.04 (m, 1H), 2.79 (dt, *J* = 16.2, 7.5 Hz, 1H), 1.17 (s, 9H).

^{13}C NMR (151 MHz, Chloroform-*d*) δ 153.32, 141.00, 131.78 (d, *J* = 2.8 Hz), 131.03 (d, *J* = 2.7 Hz), 130.59, 130.55, 130.53, 130.51, 130.46, 129.87 (d, *J* = 3.0 Hz), 129.60 (d, *J* = 5.0 Hz), 129.56, 129.01, 128.08, 128.01, 127.38, 127.30, 117.39, 108.47 (d, *J* = 2.4 Hz), 79.91, 54.92 (d, *J* = 72.7 Hz), 39.20, 27.43, 27.12(2), 27.02.

^{31}P NMR (243 MHz, C_6D_6): δ 32.5 (for major rotamer), 29.9 (for minor rotamer).

HRMS (m/z) calcd for $C_{27}H_{27}N_2O_3PNa$ $[M+Na]^+$ 481.1652, found 481.1656; $[\alpha]_D^{25}$ = + 37.1 (c = 0.20, $CHCl_3$); The *ee* was determined by HPLC analysis (Chiralpak OJ-RH, CH_3CN/H_2O = 72/28 v/v, 1.0 mL/min, 280 nm, t_R (major) = 34.2 min, t_R (minor) = 36.9 min), 90% *ee*.

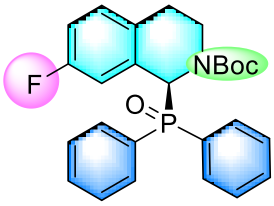

4oaa
92% yield, 94% ee

^{1}H NMR (600 MHz, C_6D_6) δ 8.36 – 8.21 (m, 2H), 7.94 (ddt, *J* = 10.8, 6.7, 1.6 Hz, 2H), 7.14 – 7.12 (m, 1H), 7.08 (dq, *J* = 8.4, 2.3, 1.9 Hz, 1H), 7.05 – 7.00 (m, 1H), 6.99 – 6.89 (m, 3H), 6.64 – 6.60 (m, 2H), 6.46 (d, *J* = 4.2 Hz, 1H), 6.13 – 6.08 (m, 1H), 4.17 (ddd, *J* = 12.9, 9.5, 5.1 Hz, 1H), 3.74 (dddd, *J* = 13.0, 6.0, 4.6, 1.1 Hz, 1H), 2.89 – 2.76 (m, 1H), 2.61 – 2.40 (m, 1H), 1.25 (s, 9H).

^{13}C NMR (151 MHz, C_6D_6) δ 160.48 (d, *J* = 2.8 Hz), 158.87 (d, *J* = 2.7 Hz), 153.60 (d, *J* = 3.5 Hz), 131.84 (d, *J* = 4.2 Hz), 131.38 (d, *J* = 7.3 Hz), 131.26 (d, *J* = 3.2 Hz), 131.20 (d, *J* = 5.6 Hz), 130.90, 130.84, 130.76 (d, *J* = 2.6 Hz), 130.70, 130.65, 130.44 (d, *J* = 3.2 Hz), 129.47 (d, *J* = 8.4 Hz), 127.65(d, *J* = 11.2 Hz), 127.18, 127.12 (d, *J* = 3.9 Hz), 126.92, 126.77 – 126.75, 113.26 (ddd, *J* = 22.9, 10.5, 3.0 Hz), 78.84, 55.42 (d, *J* = 73.8 Hz), 40.02, 27.02(3), 26.51.

^{31}P NMR (243 MHz, C_6D_6): δ 31.3 (for major rotamer), 28.6 (for minor rotamer).

HRMS (m/z) calcd for $C_{26}H_{27}FNO_3PNa$ $[M+Na]^+$ 474.1605, found 474.1613; $[\alpha]_D^{25}$ = + 48.0 (c = 0.20, $CHCl_3$); The *ee* was determined by HPLC analysis (Chiralpak OJ-RH, CH_3CN/H_2O = 60/40 v/v, 1.0 mL/min, 280 nm, t_R (major) = 8.0 min, t_R (minor) = 9.7 min), 94% *ee*.

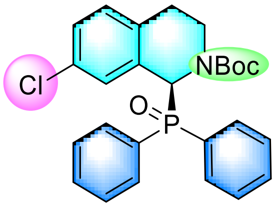

4paa
76% yield, 95% ee

^{1}H NMR (600 MHz, C_6D_6) δ 8.29 (ddt, *J* = 11.1, 6.2, 1.3 Hz, 2H), 7.90 (ddt, *J* = 9.7, 6.9, 1.4 Hz, 2H), 7.13 (dt, *J* = 6.4, 1.5 Hz, 1H), 7.11 – 7.06 (m, 1H), 7.06 – 7.03 (m, 1H), 7.03 – 6.98 (m, 1H), 6.98 – 6.94 (m, 2H), 6.91 (dt, *J* = 8.1, 2.0 Hz, 1H), 6.56 (d, *J* = 8.1 Hz, 1H), 6.41 (d, *J* = 4.1 Hz, 1H), 6.30 (d, *J* = 2.1 Hz, 1H), 4.20 (ddd, *J* = 13.0, 9.5, 5.1 Hz, 1H), 3.73 (dddd, *J* = 13.0, 6.0, 4.6, 1.1 Hz, 1H), 2.82 (dtd, *J* = 16.5, 5.0, 2.1 Hz, 1H), 2.46 (dddd, *J* = 16.0, 9.2, 6.3, 2.3 Hz, 1H), 1.25 (s, 9H).

^{13}C NMR (151 MHz, C_6D_6) δ 153.61 (d, *J* = 3.8 Hz), 134.16 (d, *J* = 3.8 Hz), 131.66, 131.30, 131.04 (d, *J* = 6.6 Hz), 130.93, 130.87, 130.79 (d, *J* = 2.6 Hz), 130.73, 130.67, 130.49 (d, *J* = 3.2 Hz), 130.05 (d, *J* = 2.8 Hz), 129.38, 127.65 (d, *J* = 11.1 Hz), 127.17 (d, *J* = 11.1 Hz), 126.93, 126.76, 126.59 (d, *J* = 3.1 Hz), 126.38 (d, *J* = 2.8 Hz), 78.89, 55.18 (d, *J* = 73.6 Hz), 39.84, 27.01(3), 26.59.

^{31}P NMR (243 MHz, C_6D_6): δ 31.5 (for major rotamer), 28.9 (for minor rotamer).

HRMS (m/z) calcd for $C_{26}H_{27}ClNO_3PNa$ $[M+Na]^+$ 490.1316, found:490.1313; $[\alpha]_D^{25}$ = +22.2 (c = 0.20, $CHCl_3$); The *ee* was determined by HPLC analysis (Chiralpak OJ-RH, CH_3CN/H_2O = 65/35 v/v, 1.0 mL/min, 280 nm, t_R (major) = 22.2 min, t_R (minor) = 24.7 min), 89% *ee*.

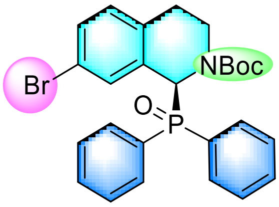

4qaa
56%yield, 76% ee

^{1}H NMR (600 MHz, C_6D_6) δ 8.29 (ddt, *J* = 11.1, 6.3, 1.3 Hz, 2H), 7.93 – 7.79 (m, 2H), 7.14 – 7.12 (m, 1H), 7.11 – 7.07 (m, 1H), 7.05 (dtd, *J* = 6.9, 5.2, 1.6 Hz, 2H), 7.03 – 7.00 (m, 1H), 7.00 – 6.94 (m, 2H), 6.49 (d, *J* = 8.1 Hz, 1H), 6.43 (d, *J* = 2.1 Hz, 1H), 6.39 (d, *J* = 4.2 Hz, 1H), 4.21 (ddd, *J* = 12.9, 9.5, 5.2 Hz, 1H), 3.79 – 3.62 (m, 1H), 2.80 (ddt, *J* = 14.5, 5.3, 2.6 Hz, 1H), 2.43 (dddd, *J* = 16.1, 9.2, 6.3, 2.3 Hz, 1H), 1.25 (s, 9H).

^{13}C NMR (151 MHz, C_6D_6) δ 153.61 (d, *J* = 3.6 Hz), 134.65 (d, *J* = 3.8 Hz), 131.61 (d, *J* = 4.4 Hz), 130.94, 130.88, 130.80 (d, *J* = 2.8 Hz), 130.73, 130.67, 130.50 (d, *J* = 2.8 Hz), 129.68, 129.53 (d, *J* = 3.2 Hz), 129.27 (d, *J* = 2.8 Hz), 127.66 (d, *J* = 11.4 Hz), 127.17 (d, *J* = 11.9 Hz), 127.13, 126.92, 126.76, 118.01 (d, *J* = 2.9 Hz), 78.90, 55.06 (d, *J* = 73.7 Hz), 39.77 , 27.00(3), 26.63 .

^{31}P NMR (243 MHz, C_6D_6): δ 31.6 (for major rotamer), 28.9 (for minor rotamer).

HRMS (m/z) calcd for $C_{26}H_{27}BrNO_3PNa$ $[M+Na]^+$ 534.0804, found 534.0804; $[\alpha]_D^{25}$ = + 17.0 (c = 0.20, $CHCl_3$); The *ee* was determined by HPLC analysis (Chiralpak OD-RH-3um, CH_3CN/H_2O

= 65/35 v/v, 0.6 mL/min, 280 nm, t_R (major) = 32.0 min, t_R (minor) = 34.5 min), 76% *ee*.

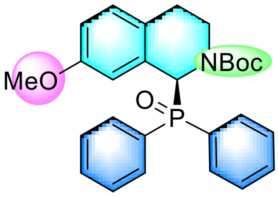

4raa

82% yield, 95% ee

^{1}H NMR (600 MHz, C_6D_6) δ 8.41 – 8.30 (m, 2H), 7.98 (ddt, *J* = 10.2, 4.8, 2.3 Hz, 2H), 7.17 (d, *J* = 2.8 Hz, 1H), 7.11 – 7.07 (m, 1H), 7.05 – 6.99 (m, 1H), 6.95 – 6.87 (m, 3H), 6.82 (d, *J* = 8.4 Hz, 1H), 6.75 – 6.71 (m, 1H), 6.56 (d, *J* = 3.9 Hz, 1H), 5.98 (s, 1H), 4.35 – 4.21 (m, 1H), 3.90 – 3.82 (m, 1H), 2.99 (s, 3H), 2.83 (d, *J* = 15.7 Hz, 1H), 2.69 – 2.59 (m, 1H), 1.26 (s, 9H).

^{13}C NMR (151 MHz, Benzene-*d*$_6$) δ 158.11 (d, *J* = 2.4 Hz), 155.33 (d, *J* = 4.0 Hz), 134.37, 133.79 (d, *J* = 4.8 Hz), 133.17, 132.48, 132.43 (d, *J* = 2.7 Hz), 132.38, 131.89 (d, *J* = 2.6 Hz), 131.82 (d, *J* = 2.6 Hz), 131.71, 130.78 (d, *J* = 1.7 Hz), 129.05 (d, *J* = 11.1 Hz), 128.70 (d, *J* = 6.4 Hz), 128.65, 128.47, 128.31, 115.37 (d, *J* = 2.6 Hz), 112.24 (d, *J* = 2.9 Hz), 80.22, 57.09 (d, *J* = 74.3 Hz), 54.71, 41.84, 28.59(3), 27.97.

^{31}P NMR (243 MHz, C_6D_6): δ 31.0 (for major rotamer), 28.3 (for minor rotamer).

HRMS (m/z) calcd for $C_{27}H_{30}NO_4PNa$ $[M+Na]^+$ 486.1810, found 486.1805; $[\alpha]_D^{25}$ = + 22.6 (c = 0.20, $CHCl_3$); The *ee* was determined by HPLC analysis (Chiralpak OJ-RH, CH_3CN/H_2O = 65/35 v/v, 1.0 mL/min, 280 nm, t_R (major) = 12.3 min, t_R (minor) = 13.4 min), 95% *ee*.

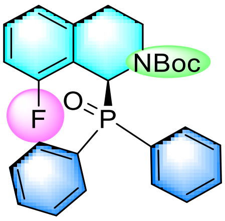

4saa

53% yield, 3% ee

^{1}H NMR (600 MHz, Benzene-*d*$_6$) δ 8.29 (ddt, *J* = 11.2, 6.1, 1.4 Hz, 2H), 8.21 (ddt, *J* = 10.9, 7.2, 2.0 Hz, 2H), 7.12 (ddd, *J* = 9.1, 5.3, 2.3 Hz, 2H), 7.07 (dd, *J* = 7.3, 1.6 Hz, 1H), 7.06 – 6.99 (m, 3H), 6.91 (d, *J* = 3.7 Hz, 1H), 6.70 (tdd, *J* = 7.6, 5.5, 1.6 Hz, 1H), 6.60 (d, *J* = 7.5 Hz, 1H), 6.42 (t, *J* = 8.7 Hz, 1H), 3.76 (dtd, *J* = 12.2, 6.1, 1.0 Hz, 1H), 3.50 (dt, *J* = 14.9, 7.0 Hz, 1H), 3.40 (ddd, *J* = 12.2, 8.1, 5.9 Hz, 1H), 2.46 (dt, *J* = 15.5, 6.1 Hz, 1H), 1.24 (s, 9H).

^{13}C NMR (151 MHz, C_6D_6) δ 157.98 (dd, *J* = 246.8, 4.1 Hz), 153.71, 138.56, 131.96, 131.32, 131.13, 131.07, 130.77, 130.71, 130.54 (d, *J* = 3.2 Hz), 130.28 (d, *J* = 2.9 Hz), 127.58 (dd, *J* = 8.7, 2.8 Hz), 127.27 (d, *J* = 11.5 Hz), 127.15, 126.94, 126.77, 122.91 (t, *J* = 2.7 Hz), 118.49 (d, *J* = 17.0 Hz), 111.77 (d, *J* = 22.0 Hz), 78.83, 51.09 (d, *J* = 73.6 Hz), 40.45, 27.07(4).

^{31}P NMR (243 MHz, C_6D_6): δ 31.8 (for major rotamer), 29.9 (for minor rotamer).

HRMS (m/z) calcd for $C_{26}H_{27}FNO_3PNa$ $[M+Na]^+$ 474.1605, found 474.1611; $[\alpha]_D^{25}$ = 0 (c = 0.20, $CHCl_3$); The *ee* was determined by HPLC analysis (Chiralpak AS-RH, CH_3CN/H_2O = 50/50 v/v, 1.0 mL/min, 280 nm, t_R (major) = 4.1 min, t_R (minor) = 9.7 min), 3% *ee*.

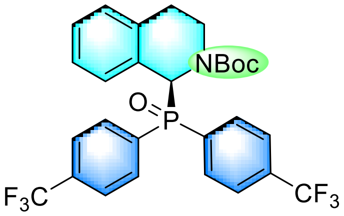

4aba, 92% yield, 79% *ee*

^{1}H NMR (600 MHz, C_6D_6) δ 8.15 (dd, *J* = 10.4, 8.4 Hz, 2H), 7.95 – 7.82 (m, 2H), 7.39 (d, *J* = 6.6 Hz, 2H), 7.20 (d, *J* = 6.6 Hz, 2H), 6.93 (t, *J* = 7.4 Hz, 1H), 6.88 (d, *J* = 7.4 Hz, 1H), 6.65 (t, *J* = 7.4 Hz, 1H), 6.40 (d, *J* = 2.1 Hz, 1H), 6.33 (d, *J* = 7.7 Hz, 1H), 3.94 (ddd, *J* = 13.0, 7.9, 5.4 Hz, 1H), 3.56 (dt, *J* = 12.4, 6.1 Hz, 1H), 3.17 – 3.06 (m, 1H), 2.57 – 2.47 (m, 1H), 1.17 (s, 9H).

^{13}C NMR (151 MHz, C_6D_6) δ 155.22 (d, *J* = 2.9 Hz), 137.51 (d, *J* = 3.9 Hz), 137.18 (d, *J* = 11.0 Hz), 136.59 (d, *J* = 4.1 Hz), 134.23, 134.22 (d, *J* = 2.8 Hz), 134.03 (d, *J* = 3.5 Hz), 134.01 (d, *J* = 3.2 Hz), 133.82 (d, *J* = 2.8 Hz), 133.01 (d, *J* = 10.8 Hz), 132.82 (d, *J* = 11.2 Hz), 130.12, 129.73 (d, *J* = 2.0 Hz), 128.68, 128.47, 128.31, 127.69 (d, *J* = 3.6 Hz), 126.40 (d, *J* = 2.3 Hz), 126.17 – 125.91 (m), 125.47 - 125.37 (m), 80.80, 57.35 (d, *J* = 75.4 Hz), 41.90, 28.73, 28.37 (3).

^{31}P NMR (243 MHz, C_6D_6): δ 29.5 (for major rotamer), 26.7 (for minor rotamer).

HRMS (m/z) calcd for $C_{28}H_{26}F_6NO_3PNa$ $[M+Na]^+$ 592.1447, found 592.1454; $[\alpha]_D^{25}$ = + 7.5 (c = 0.20, $CHCl_3$); The *ee* was determined by HPLC analysis (Chiralpak OD-RH, CH_3CN/H_2O = 45/55 v/v, 1.0 mL/min, 280 nm, t_R (major) = 11.0 min, t_R (minor) = 13.0 min), 79% *ee*.

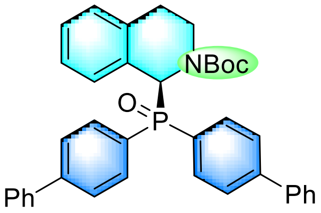

4aca, 88% yield, 82% ee

^{1}H NMR (600 MHz, C_6D_6) δ 9.53 (d, *J* = 8.6 Hz, 1H), 9.41 (d, *J* = 8.6 Hz, 1H), 8.72 (dd, *J* = 14.5, 7.1 Hz, 1H), 8.10 (dd, *J* = 14.2, 6.9 Hz, 1H), 7.61 (d, *J* = 8.1 Hz, 1H), 7.52 – 7.44 (m, 3H), 7.29 (td, *J* = 8.0, 2.3 Hz, 1H), 7.19 (t, *J* = 7.4 Hz, 1H), 7.14 (d, *J* = 9.5 Hz, 2H), 7.08 (t, *J* = 7.4 Hz, 2H), 7.02 – 6.97 (m, 2H), 6.95 (d, *J* = 3.9 Hz, 1H), 6.91 (dd, *J* = 12.5, 7.0 Hz, 2H), 6.79 (t, *J* = 7.3 Hz, 2H), 6.25 (t, *J* = 7.4 Hz, 1H), 5.95 (d, *J* = 7.6 Hz, 1H), 4.74 – 4.64 (m, 1H), 3.80 – 3.72 (m, 1H), 3.40 (d, *J* = 15.7 Hz, 1H), 2.77 – 2.67 (m, 1H), 1.24 (s, 9H).

^{13}C NMR (151 MHz, C_6D_6) δ 156.15 (d, *J* = 4.9 Hz), 137.91 (d, *J* = 3.7 Hz), 135.80 (d, *J* = 8.0 Hz), 135.56 (d, *J* = 7.5 Hz), 135.05 (d, *J* = 8.7 Hz), 134.60 (d, *J* = 7.7 Hz), 134.33 (d, *J* = 8.9 Hz), 133.53 (d, *J* = 2.8 Hz), 133.48 (d, *J* = 2.7 Hz), 132.81 (d, *J* = 11.7 Hz), 131.07, 130.46, 130.16, 129.85, 129.59, 129.38 (d, *J* = 1.5 Hz), 129.27, 128.74 (d, *J* = 4.1 Hz), 128.68, 128.47, 128.31, 127.88 (d, *J* = 2.7 Hz), 127.83, 127.44, 126.69 (d, *J* = 9.8 Hz), 125.57, 125.31, 125.22, 124.86, 124.77, 80.41, 58.05 (d, *J* = 76.8 Hz), 42.07, 28.74, 28.47 (3).

^{31}P NMR (243 MHz, C_6D_6): δ 39.5 (for major rotamer), 25.6 (for minor rotamer).

HRMS (m/z) calcd for $C_{38}H_{36}NO_3PNa$ $[M+Na]^+$ 608.2325, found 608.2328; $[\alpha]_D^{25}$ = + 9.9 (c = 0.20, $CHCl_3$); The *ee* was determined by HPLC analysis (Chiralpak OD-RH, CH_3CN/H_2O = 35/65 v/v, 1.0 mL/min, 280 nm, t_R (major) = 16.8 min, t_R (minor) = 20.9 min), 82% *ee*.

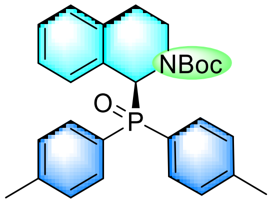

4ada, 85% yield, 88% ee

^{1}H NMR (600 MHz, C_6D_6) δ 8.26 (dd, *J* = 10.8, 8.1 Hz, 2H), 7.99 (dd, *J* = 10.4, 8.1 Hz, 2H), 7.01 (dd, *J* = 8.0, 2.2 Hz, 2H), 6.96 - 6.90(m, 3H), 6.84 (dd, *J* = 8.0, 2.1 Hz, 2H), 6.72 (t, *J* = 7.3 Hz, 1H), 6.59 (t, *J* = 5.6 Hz, 1H), 4.20 (ddd, *J* = 12.9, 9.0, 5.3 Hz, 1H), 3.83 – 3.72 (m, 1H), 3.07 (ddd, *J* = 15.9, 4.8, 3.3 Hz, 1H), 2.68 – 2.57 (m, 1H), 1.99 (s, 3H), 1.90 (s, 3H), 1.27 (s, 9H).

^{13}C NMR (151 MHz, C_6D_6) δ 155.28 (d, *J* = 3.4 Hz), 142.34 (d, *J* = 2.6 Hz), 142.01 (d, *J* = 2.7 Hz), 137.30 (d, *J* = 3.8 Hz), 132.54 (d, *J* = 1.3 Hz), 132.48, 131.54, 129.90 (d, *J* = 11.5 Hz), 129.70 (d, *J* = 1.9 Hz), 129.44 (d, *J* = 11.9 Hz), 128.68, 128.47, 128.31, 128.09 (d, *J* = 3.3 Hz), 127.84 (d, *J* = 2.5 Hz), 127.92 (d, *J* = 5.8 Hz), 126.09 (d, *J* = 2.2 Hz), 80.08, 57.35 (d, *J* = 73.8 Hz), 41.73, 28.83, 28.59 (3), 21.63 (2).

^{31}P NMR (243 MHz, C_6D_6): δ 31.9 (for major rotamer), 29.0 (for minor rotamer).

HRMS (m/z) calcd for $C_{28}H_{32}NO_3PNa$ $[M+Na]^+$ 484.2012, found 484.2016; $[\alpha]_D^{25}$ = + 12.6 (c = 0.20, $CHCl_3$); The *ee* was determined by HPLC analysis (Chiralpak OD-RH, CH_3CN/H_2O = 55/45 v/v, 1.0 mL/min, 280 nm, t_R (major) = 17.2 min, t_R (minor) = 19.5 min), 88% *ee*.

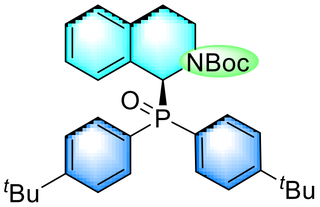

4a**ea**, 88% yield, 91% ee

^{1}H NMR (600 MHz, C_6D_6) δ 8.32 (dd, *J* = 10.7, 8.4 Hz, 2H), 8.10 (dd, *J* = 10.3, 8.4 Hz, 2H), 7.30 (dd, *J* = 8.4, 2.4 Hz, 2H), 7.17 – 7.15 (m, 2H), 6.97 – 6.88 (m, 2H), 6.68 (t, *J* = 7.3 Hz, 1H), 6.64 (d, *J* = 3.6 Hz, 1H), 6.58 (d, *J* = 7.7 Hz, 1H), 4.20 (ddd, *J* = 12.9, 9.0, 5.2 Hz, 1H), 3.85 – 3.68 (m, 1H), 3.17 – 2.99 (m, 1H), 2.63 (dd, *J* = 15.4, 7.4 Hz, 1H), 1.27 (s, 9H), 1.13 (s, 9H), 1.07 (s, 9H).

^{13}C NMR (151 MHz, C_6D_6) δ 155.30 (d, *J* = 2.7 Hz), 155.22 (d, *J* = 3.1 Hz), 154.79 (d, *J* = 2.7 Hz), 137.28 (d, *J* = 3.7 Hz), 132.52 (d, *J* = 9.8 Hz), 132.45 (d, *J* = 9.3 Hz), 131.58, 131.15, 130.97, 130.54, 130.32, 129.65 (d, *J* = 1.8 Hz), 128.68, 128.47, 128.31, 128.13 (d, *J* = 3.3 Hz), 127.81 (d, *J* = 2.5 Hz), 126.19 (d, *J* = 11.6 Hz), 125.71 (d, *J* = 11.7 Hz), 80.01, 57.42 (d, *J* = 73.5 Hz), 41.80, 35.14, 35.12, 31.47 (3), 31.41 (3), 28.91, 28.71 (3).

^{31}P NMR (243 MHz, C_6D_6): δ 31.9 (for major rotamer), 28.8 (for minor rotamer).

HRMS (m/z) calcd for $C_{34}H_{44}NO_3PNa$ $[M+Na]^+$ 568.2951, found 568.2955; $[\alpha]_D^{25}$ = + 13.5 (c = 0.20, $CHCl_3$); The *ee* was determined by HPLC analysis (Chiralpak OD-RH, CH_3CN/H_2O = 45/55 v/v, 1.0 mL/min, 280 nm, t_R (major) = 19.4 min, t_R (minor) = 21.7 min), 91% *ee*.

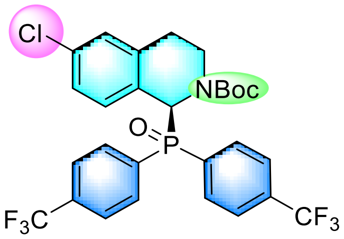

4iba, 98% yield, 86% *ee*

^{1}H NMR (600 MHz, C_6D_6): δ 8.12 (dd, *J* = 10.5, 8.4 Hz, 2H), 7.93 – 7.75 (m, 2H), 7.47 – 7.35 (m, 2H), 7.20 (d, *J* = 6.6 Hz, 2H), 6.86 (s, 1H), 6.58 (dd, *J* = 8.2, 1.9 Hz, 1H), 6.27 (d, *J* = 2.3 Hz, 1H), 6.03 (dd, *J* = 8.2, 1.5 Hz, 1H), 3.93 – 3.75 (m, 1H), 3.49 (dt, *J* = 12.4, 6.0 Hz, 1H), 2.88 (dt, *J* = 14.7, 4.9 Hz, 1H), 2.43 – 2.22 (m, 1H), 1.16 (s, 9H).

^{13}C NMR (151 MHz, C_6D_6): δ 155.10 (d, *J* = 3.0 Hz), 139.43 (d, *J* = 3.9 Hz), 136.79, 136.20 (d, *J* = 7.3 Hz), 134.39 (d, *J* = 2.8 Hz), 134.32 (d, *J* = 3.5 Hz), 134.18 (t, *J* = 2.9 Hz), 133.98 (d, *J* = 2.7 Hz), 132.99 (d, *J* = 9.6 Hz), 132.76 (d, *J* = 9.0 Hz), 129.87 (d, *J* = 1.9 Hz), 128.79 (d, *J* = 3.5 Hz), 128.68, 128.48, 128.31, 127.94, 126.54 (d, *J* = 2.3 Hz), 126.25 – 125.97 (m), 125.66 – 125.38 (m), 125.37, 123.67 (d, *J* = 31.7 Hz), 81.05, 56.77 (d, *J* = 75.3 Hz), 41.35, 28.41, 28.34 (3).

^{31}P NMR (243 MHz, C_6D_6): δ 29.4 (for major rotamer), 26.6 (for minor rotamer).

HRMS (m/z) calcd for $C_{28}H_{25}ClF_6NO_3PNa$ $[M+Na]^+$ 626.1062, found 626.1060; $[\alpha]_D^{25}$ = + 3.7 (c = 0.20, $CHCl_3$); The *ee* was determined by HPLC analysis (Chiralpak OJ-RH, CH_3CN/H_2O = 45/55 v/v, 1.0 mL/min, 280 nm, t_R (major) = 6.6 min, t_R (minor) = 8.0 min), 86% *ee*.

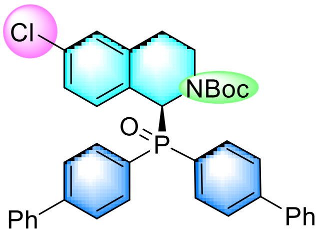

4ica, 93% yield, 93% *ee*

^{1}H NMR (600 MHz, C_6D_6): δ 8.42 (dd, *J* = 10.7, 8.3 Hz, 2H), 8.12 (dd, *J* = 10.3, 8.3 Hz, 2H), 7.54 (dd, *J* = 8.2, 2.4 Hz, 2H), 7.42 – 7.38 (m, 2H), 7.37 (dd, *J* = 8.2, 2.2 Hz, 2H), 7.35 – 7.32 (m, 2H), 7.18 (dd, *J* = 15.4, 7.4 Hz, 3H), 7.11 (dd, *J* = 15.7, 7.4 Hz, 3H), 6.91 (s, 1H), 6.65 (dd, *J* = 8.2, 1.9 Hz, 1H), 6.55 (d, *J* = 3.4 Hz, 1H), 6.36 (d, *J* = 8.2 Hz, 1H), 4.17 (ddd, *J* = 13.0, 9.0, 5.3 Hz, 1H), 3.77 – 3.67 (m, 1H), 2.97 – 2.86 (m, 1H), 2.49 – 2.39 (m, 1H), 1.24 (s, 9H).

^{13}C NMR (151 MHz, C_6D_6) δ 155.22 (d, *J* = 3.3 Hz), 145.21 (d, *J* = 2.6 Hz), 145.11 (d, *J* = 2.6 Hz), 140.93, 140.46, 139.37 (d, *J* = 3.6 Hz), 133.81, 133.08 (d, *J* = 9.5 Hz), 132.96 (d, *J* = 8.9 Hz), 132.22 (d, *J* = 6.4 Hz), 131.63, 131.55, 129.82 (d, *J* = 1.5 Hz), 129.75, 129.52, 129.50 (d, *J* = 5.8 Hz), 129.26 (d, *J* = 2.9 Hz), 128.80, 128.68, 128.65, 128.47, 128.31, 127.93 (d, *J* = 14.6 Hz), 127.88, 127.54 (d, *J* = 11.8 Hz), 126.37, 80.51, 56.93 (d, *J* = 74.0 Hz), 41.31, 28.57, 28.55 (3).

^{31}P NMR (243 MHz, C_6D_6): δ 31.5 (for major rotamer), 28.5 (for minor rotamer).

HRMS (m/z) calcd for $C_{38}H_{52}ClNO_3PNa$ $[M+Na]^+$ 642.1941, found 642.1936; $[\alpha]_D^{25}$ = + 22.5 (c = 0.20, $CHCl_3$); The *ee* was determined by HPLC analysis (Chiralpak OD-RH, CH_3CN/H_2O = 25/75 v/v, 1.0 mL/min, 280 nm, t_R (major) = 11.6 min, t_R (minor) = 17.6 min), 93% *ee*.

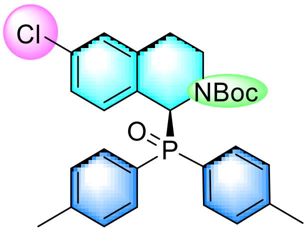

4ida, 83% yield, 90% *ee*

^{1}H NMR (600 MHz, C_6D_6): δ 8.23 (dd, *J* = 10.8, 8.1 Hz, 2H), 8.03 – 7.79 (m, 2H), 7.01 (dd, *J* = 8.0, 2.1 Hz, 2H), 6.88 (s, 1H), 6.83 (dd, *J* = 7.9, 2.0 Hz, 2H), 6.64 (dd, *J* = 8.1, 1.8 Hz, 1H), 6.46 (d, *J* = 3.9 Hz, 1H), 6.33 – 6.26 (m, 1H), 4.22 – 4.02 (m, 1H), 3.80 – 3.61 (m, 1H), 2.85 (d, *J* = 15.3 Hz, 1H), 2.54 – 2.30 (m, 1H), 1.98 (s, 3H), 1.91 (s, 3H), 1.27 (s, 9H).

^{13}C NMR (151 MHz, C_6D_6): δ 155.17 (d, *J* = 3.5 Hz), 142.53 (d, *J* = 2.7 Hz), 142.20 (d, *J* = 2.7 Hz), 139.30 (d, *J* = 3.7 Hz), 133.61 (d, *J* = 3.2 Hz), 132.82 (d, *J* = 8.4 Hz), 132.45 (d, *J* = 7.5 Hz), 132.39 (d, *J* = 6.9 Hz), 130.70, 130.57, 130.08, 129.96 (d, *J* = 11.7 Hz), 129.76 (d, *J* = 1.9 Hz), 129.50 (d, *J* = 11.9 Hz), 129.26 (d, *J* = 3.1 Hz), 128.68, 128.47, 128.31, 126.21 (d, *J* = 2.1 Hz), 80.30, 56.80 (d, *J* = 73.7 Hz), 41.19, 28.56 (3), 28.51, 21.63 (2).

^{31}P NMR (243 MHz, C_6D_6): δ 31.6 (for major rotamer), 28.8 (for minor rotamer).

HRMS (m/z) calcd for $C_{28}H_{31}ClNO_3PNa$ $[M+Na]^+$ 518.1628, found 518.1628; $[\alpha]_D^{25}$ = + 19.2 (c = 0.20, $CHCl_3$); The *ee* was determined by HPLC analysis (Chiralpak OD-RH, CH_3CN/H_2O = 40/60 v/v, 1.0 mL/min, 280 nm, t_R (major) = 7.3 min, t_R (minor) = 8.5 min), 90% *ee*.

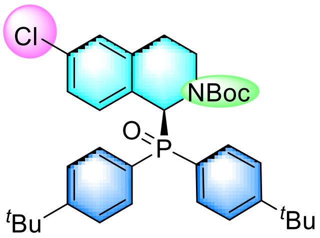

4iea, 85% yield, 95% *ee*

^{1}H NMR (600 MHz, C_6D_6): δ 8.30 (dd, *J* = 10.8, 8.4 Hz, 2H), 8.03 (dd, *J* = 10.3, 8.4 Hz, 2H), 7.30 (dd, *J* = 8.4, 2.4 Hz, 2H), 7.14 (dd, *J* = 8.4, 2.4 Hz, 2H), 6.88 (s, 1H), 6.61 (dd, *J* = 8.2, 1.9 Hz, 1H), 6.51 (d, *J* = 3.7 Hz, 1H), 6.32 – 6.25 (m, 1H), 4.13 (ddd, *J* = 13.0, 9.2, 5.2 Hz, 1H), 3.75 – 3.63 (m, 1H), 2.85 (d, *J* = 15.3 Hz, 1H), 2.47 – 2.34 (m, 1H), 1.27 (s, 9H), 1.12 (s, 9H), 1.07 (s, 9H).

^{13}C NMR (151 MHz, C_6D_6) δ 155.55 (d, *J* = 2.6 Hz), 155.11 (d, *J* = 3.2 Hz), 155.01 (d, *J* = 2.7 Hz), 133.59 (d, *J* = 3.1 Hz), 132.46 (d, *J* = 9.5 Hz), 132.36 (d, *J* = 9.0 Hz), 130.79, 130.56, 130.18, 130.04, 129.91, 129.72, 129.30 (d, *J* = 3.0 Hz), 128.68, 128.47, 128.31, 126.27 (d, *J* = 11.3 Hz), 126.23, 125.79 (d, *J* = 11.7 Hz), 80.24, 56.86 (d, *J* = 73.4 Hz), 41.25, 35.16, 35.14, 31.45 (3), 31.40 (3), 28.67 (3), 28.59.

^{31}P NMR (243 MHz, C_6D_6): δ 31.7 (for major rotamer), 28.7 (for minor rotamer).

HRMS (m/z) calcd for $C_{34}H_{43}ClNO_3PNa$ $[M+Na]^+$ 602.2567, found 602.2563; $[\alpha]_D^{25}$ = + 24.3 (c = 0.20, $CHCl_3$); The *ee* was determined by HPLC analysis (Chiralpak OD-RH, CH_3CN/H_2O = 45/55 v/v, 1.0 mL/min, 280 nm, t_R (major) = 9.8 min, t_R (minor) = 10.9 min), 95% *ee*.

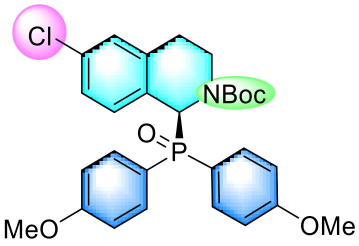

4ifa, 80% yield, 83% *ee*

^{1}H NMR (600 MHz, C_6D_6): δ 8.23 (dd, *J* = 10.4, 8.8 Hz, 2H), 7.95 – 7.89 (m, 2H), 6.90 (s, 1H), 6.80 (dd, *J* = 8.6, 1.9 Hz, 2H), 6.68 (dd, *J* = 8.2, 1.6 Hz, 1H), 6.62 (dd, *J* = 8.6, 1.8 Hz, 2H), 6.40 (d, *J* = 3.9 Hz, 1H), 6.31 (d, *J* = 7.9 Hz, 1H), 4.14 (ddd, *J* = 14.1, 9.2, 5.3 Hz, 1H), 3.78 – 3.68 (m, 1H), 3.19 (s, 3H), 3.11 (s, 3H), 2.87 (d, *J* = 15.8 Hz, 1H), 2.50 – 2.40 (m, 1H), 1.27 (s, 9H).

^{13}C NMR (151 MHz, C_6D_6) δ 163.10, 155.18 (d, *J* = 3.3 Hz), 139.29 (d, *J* = 3.6 Hz), 134.24 (d, *J* = 5.0 Hz), 134.17 (d, *J* = 4.4 Hz), 133.56 (d, *J* = 3.1 Hz), 130.09, 129.73, 129.34 (d, *J* = 2.9 Hz), 128.68, 128.47, 128.31, 126.19 (d, *J* = 1.9 Hz), 124.82 (s), 124.16 (d, *J* = 5.8 Hz), 114.88 (d, *J* = 12.2 Hz), 114.47 (d, *J* = 12.5 Hz), 80.34, 56.89 (d, *J* = 74.1 Hz), 55.09 (2), 41.16, 28.60 (3), 28.54.

^{31}P NMR (243 MHz, C_6D_6): δ 31.7 (for major rotamer), 28.6 (for minor rotamer).

HRMS (m/z) calcd for $C_{28}H_{31}ClNO_5PNa$ $[M+Na]^+$ 550.1526, found 550.1521; $[\alpha]_D^{25}$ = + 23.9 (c = 0.20, $CHCl_3$); The *ee* was determined by HPLC analysis (Chiralpak AS-RH, CH_3CN/H_2O = 55/45 v/v, 1.0 mL/min, 280 nm, t_R (major) = 11.7 min, t_R (minor) = 13.9 min), 83% *ee*.

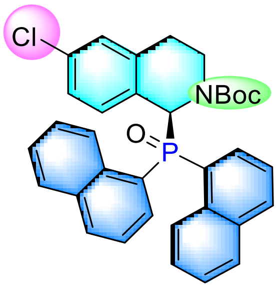

4iga, 80% yield, 91% *ee*

^{1}H NMR (600 MHz, C_6D_6) δ 9.48 (d, *J* = 8.6 Hz, 1H), 9.34 (d, *J* = 8.6 Hz, 1H), 8.68 (dd, *J* = 14.5, 7.0 Hz, 1H), 8.05 (dd, *J* = 14.0, 6.5 Hz, 1H), 7.61 (d, *J* = 8.2 Hz, 1H), 7.47 (t, *J* = 8.5 Hz, 2H), 7.44 (d, *J* = 8.2 Hz, 1H), 7.29 (td, *J* = 8.0, 2.5 Hz, 1H), 7.19 (t, *J* = 7.4 Hz, 1H), 7.13 (d, *J* = 7.6 Hz, 1H), 7.08 (dd, *J* = 11.9, 7.2 Hz, 2H), 6.88 (s, 2H), 6.81 (d, *J* = 4.1 Hz, 1H), 6.20 – 6.09 (m, 1H), 5.64 (d, *J* = 7.4 Hz, 1H), 4.67 – 4.53 (m, 1H), 3.72 – 3.61 (m, 1H), 3.19 (d, *J* = 15.9 Hz, 1H), 2.54 – 2.45 (m, 1H), 1.25 (s, 9H).

^{13}C NMR (151 MHz, C_6D_6) δ 156.03 (d, *J* = 4.9 Hz), 139.87 (d, *J* = 3.8 Hz), 135.64 (d, *J* = 7.3 Hz), 135.49 (d, *J* = 7.6 Hz), 135.04 (d, *J* = 8.8 Hz), 134.56 (d, *J* = 11.9 Hz), 134.29 (d, *J* = 8.9 Hz), 133.71 (d, *J* = 3.3 Hz), 133.66 (d, *J* = 2.8 Hz), 133.62 (d, *J* = 2.7 Hz), 132.74 (d, *J* = 11.6 Hz), 129.78 (d, *J* = 3.2 Hz), 129.55, 129.41 (d, *J* = 1.5 Hz), 129.36, 129.31, 128.68, 128.47, 128.31, 128.02 (d, *J* = 2.2 Hz), 127.95, 127.52, 126.85, 126.74, 125.62 (d, *J* = 1.9 Hz), 125.27 (d, *J* = 13.1 Hz), 124.78 (d, *J* = 13.8 Hz), 80.65, 57.52 (d, *J* = 76.6 Hz), 41.53, 28.71, 28.45 (3).

^{31}P NMR (243 MHz, C_6D_6): δ 39.2 (for major rotamer), 35.3 (for minor rotamer).

HRMS (m/z) calcd for $C_{34}H_{31}ClNO_3PNa$ $[M+Na]^+$ 590.1628, found 590.1623; $[\alpha]_D^{25}$ = + 12.9 (c = 0.20, $CHCl_3$); The *ee* was determined by HPLC analysis (Chiralpak OJ-RH, CH_3CN/H_2O = 50/50

v/v, 1.0 mL/min, 280 nm, t_R (major) = 16.0 min, t_R (minor) = 17.8 min), 91% *ee*.

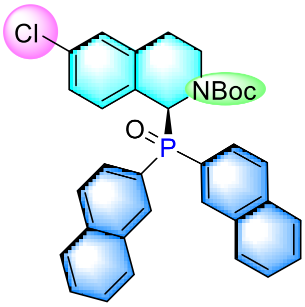

4iha, 98% yield, 96% *ee*

^{1}H NMR (600 MHz, C_6D_6) δ 9.04 (d, *J* = 12.9 Hz, 1H), 8.76 (d, *J* = 12.7 Hz, 1H), 8.50 – 8.40 (m, 1H), 8.08 (td, *J* = 8.5, 1.2 Hz, 1H), 7.73 (d, *J* = 8.0 Hz, 1H), 7.69 (dd, *J* = 8.4, 2.6 Hz, 1H), 7.53 (dd, *J* = 8.4, 2.8 Hz, 1H), 7.51 (d, *J* = 8.1 Hz, 1H), 7.46 (d, *J* = 8.2 Hz, 1H), 7.38 (d, *J* = 8.2 Hz, 1H), 7.20 (dd, *J* = 11.0, 4.0 Hz, 1H), 7.17 (d, *J* = 3.9 Hz, 2H), 7.07 (t, *J* = 7.2 Hz, 1H), 6.88 (s, 1H), 6.74 (d, *J* = 3.5 Hz, 1H), 6.41 (dd, *J* = 8.2, 1.7 Hz, 1H), 6.22 (d, *J* = 8.2 Hz, 1H), 4.22 (ddd, *J* = 13.0, 9.3, 5.2 Hz, 1H), 3.77 – 3.66 (m, 1H), 2.87 (d, *J* = 16.0 Hz, 1H), 2.54 – 2.35 (m, 1H), 1.14 (s, 9H).

^{13}C NMR (151 MHz, C_6D_6) δ 155.32 (d, *J* = 3.6 Hz), 139.24 (d, *J* = 3.7 Hz), 135.60, 135.38 (d, *J* = 2.1 Hz), 135.12 (d, *J* = 7.3 Hz), 134.30 (d, *J* = 8.7 Hz), 133.77 (d, *J* = 3.2 Hz), 133.50 (d, *J* = 3.8 Hz), 133.42 (d, *J* = 3.3 Hz), 130.85 (d, *J* = 2.5 Hz), 130.23 (d, *J* = 3.3 Hz), 129.83, 129.73, 129.62, 129.25 (d, *J* = 11.6 Hz), 129.20 (d, *J* = 3.4 Hz), 128.68, 128.63, 128.47, 128.31, 127.59 (d, *J* = 10.0 Hz), 127.48, 127.12, 126.76 (d, *J* = 10.3 Hz), 126.33 (d, *J* = 2.2 Hz), 80.53, 56.65 (d, *J* = 74.0 Hz), 41.27, 28.50, 28.41 (3).

^{31}P NMR (243 MHz, C_6D_6): δ 31.9 (for major rotamer), 28.8 (for minor rotamer).

HRMS (m/z) calcd for $C_{34}H_{31}ClNO_3PNa$ $[M+Na]^+$ 590.1628, found 590.1624; $[\alpha]_D^{25}$ = + 23.0 (c = 0.20, $CHCl_3$); The *ee* was determined by HPLC analysis (Chiralpak OJ-RH, CH_3CN/H_2O = 45/55 v/v, 1.0 mL/min, 280 nm, t_R (major) = 9.2 min, t_R (minor) = 12.1 min), 96% *ee*.

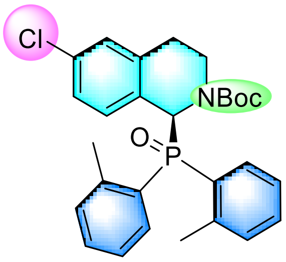

4iia, 88% yield, 90% *ee*

^{1}H NMR (600 MHz, C_6D_6) δ 8.58 (dd, *J* = 11.5, 7.8 Hz, 1H), 7.52 (dd, *J* = 13.1, 7.7 Hz, 1H), 7.24 (t, *J* = 6.5 Hz, 1H), 7.09 (t, *J* = 7.5 Hz, 1H), 6.92 (t, *J* = 7.5 Hz, 1H), 6.88 (dd, *J* = 7.3, 3.8 Hz, 1H), 6.86 (s, 1H), 6.75 – 6.68 (m, 2H), 6.55 (d, *J* = 7.2 Hz, 1H), 6.50 (d, *J* = 5.6 Hz, 1H), 5.81 (d, *J* = 8.0 Hz, 1H), 4.78 (ddd, *J* = 13.6, 10.8, 5.3 Hz, 1H), 4.01 – 3.84 (m, 1H), 2.73 – 2.59 (m, 1H), 2.57 – 2.52 (m, 1H), 2.51 (s, 3H), 2.26 (s, 3H), 1.37 (s, 9H).

^{13}C NMR (151 MHz, C_6D_6) δ 156.23 (d, *J* = 6.7 Hz), 144.35 (d, *J* = 7.5 Hz), 139.83 (d, *J* = 3.7 Hz), 133.68, 133.51 (d, *J* = 12.0 Hz), 132.99 (d, *J* = 10.2 Hz), 132.55 (d, *J* = 11.3 Hz), 132.35 (d, *J* = 10.5 Hz), 132.23 (d, *J* = 2.4 Hz), 132.17, 129.97, 129.93 (d, *J* = 2.7 Hz), 129.41, 128.68, 128.47, 128.31, 126.21 (d, *J* = 12.1 Hz), 125.61 (d, *J* = 11.8 Hz), 125.57, 80.87, 55.57 (d, *J* = 76.4 Hz),

40.89, 28.60 (3), 27.98, 22.07 (d, *J* = 3.9 Hz), 21.65 (d, *J* = 2.2 Hz).

^{31}P NMR (243 MHz, C_6D_6): δ 38.4 (for major rotamer), 24.8 (for minor rotamer).

HRMS (m/z) calcd for $C_{28}H_{31}ClNO_3PNa$ $[M+Na]^+$ 518.1628, found 518.1625; $[\alpha]_D^{25}$ = + 124.8 (c = 0.20, $CHCl_3$); The *ee* was determined by HPLC analysis (Chiralpak OD-RH-3um, CH_3CN/H_2O = 50/50 v/v, 0.6 mL/min, 280 nm, t_R (major) = 38.1 min, t_R (minor) = 40.0 min), 90% *ee*.

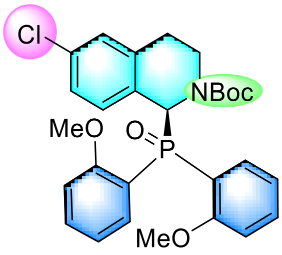

4ija, 78% yield, 86% ee

Major rotamer : ^{1}H NMR (600 MHz, $CDCl_3$) δ 7.76 (dd, *J* = 13.5, 7.6 Hz, 1H), 7.56 (t, *J* = 7.5 Hz, 1H), 7.48 (q, *J* = 7.5 Hz, 1H), 7.44 – 7.38 (m, 1H), 7.16 – 7.13 (m, 1H), 7.11 – 7.06 (m, 1H), 7.01 (dd, *J* = 8.4, 5.3 Hz, 1H), 6.99 – 6.95 (m, 1H), 6.79 – 6.75 (m, 1H), 6.74 – 6.70 (m, 1H), 6.59 (dd, *J* = 9.0, 1.8 Hz, 1H), 6.34 (d, *J* = 8.3 Hz, 1H), 4.36 – 4.29 (m, 1H), 4.14 – 4.06 (m, 1H), 3.90 (s, 3H), 3.56 (s, 3H), 3.03 (t, *J* = 14.9 Hz, 2H), 2.98 (d, *J* = 4.6 Hz, 3H), 1.09 (s, 9H).

^{31}P NMR (243 MHz, C_6D_6): δ 40.8.

^{13}C NMR (151 MHz, $CDCl_3$) 161.10, 153.96, 138.16, 135.18, 134.86 (d, *J* = 7.0 Hz), 134.35, 134.33, 133.98, 132.26, 130.53, 128.77, 128.31, 127.75, 124.87 (d, *J* = 3.2 Hz), 121.03 (d, *J* = 2.9 Hz), 120.79 (d, *J* = 11.7 Hz), 111.16 (d, *J* = 6.6 Hz), 110.54 (d, *J* = 6.6 Hz), 79.90, 56.39 (d, *J* = 73.3 Hz), 55.63, 55.00, 40.66, 28.06, 27.90 (3).

Minor rotamer : ^{1}H NMR (600 MHz, $CDCl_3$) δ 7.98 – 7.90 (m, 1H), 7.62 – 7.57 (m, 1H), 7.48 (q, *J* = 7.5 Hz, 2H), 7.13 – 7.11 (m, 1H), 7.06 – 7.02 (m, 1H), 6.99 – 6.95 (m, 1H), 6.92 (dd, J = 8.3, 5.3 Hz, 1H), 6.82 (dd, *J* = 8.4, 5.3 Hz, 1H), 6.78 (dd, *J* = 8.4, 4.7 Hz, 1H), 6.63 (d, *J* = 8.0 Hz, 1H), 6.56 – 6.51 (m, 1H), 4.23 (ddd, J = 15.1, 10.6, 4.9 Hz, 1H), 4.02 – 3.96 (m, 1H), 3.82 (s, 3H), 3.63 (s, 3H), 2.94 (dd, *J* = 10.3, 6.3 Hz, 1H), 2.85 (q, *J* = 9.0, 8.0 Hz, 2H), 1.35 (s, 9H).

^{13}C NMR (151 MHz, $CDCl_3$) δ 160.36, 154.92, 138.13, 135.13, 134.97 (d, *J* = 7.1 Hz), 134.40, 134.08, 133.74, 132.26, 130.26, 128.77, 128.31, 127.73, 125.17, 120.96 (d, J = 2.8 Hz), 120.54 (d, *J* = 11.9 Hz), 111.16 (d, *J* = 6.6 Hz), 110.54 (d, *J* = 6.6 Hz), 80.06, 55.68, 55.24, 54.68 (d, *J* = 77.9 Hz), 39.14, 28.25(3), 27.99 .

^{31}P NMR (243 MHz, C_6D_6): δ 37.0

HRMS (m/z) calcd for $C_{28}H_{31}ClNO_5PNa$ $[M+Na]^+$ 550.1526, found 550.1524; $[\alpha]_D^{25}$ = + 83.6 (c = 0.20, $CHCl_3$); The *ee* was determined by HPLC analysis (Chiralpak OD-RH, CH_3CN/H_2O = 50/50 v/v, 1.0 mL/min, 280 nm, t_R (major) = 9.0 min, t_R (minor) = 10.1 min), 86% *ee*.

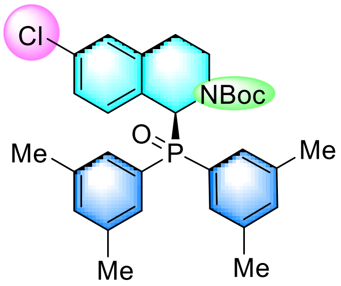

3ika, 85% yield, 94% *ee*

^{1}H NMR (600 MHz, C_6D_6) δ 8.09 (d, *J* = 11.3 Hz, 2H), 7.77 (d, *J* = 10.9 Hz, 2H), 6.88 (s, 1H), 6.81 (s, 1H), 6.70 (s, 1H), 6.64 (dd, *J* = 8.2, 1.9 Hz, 1H), 6.60 (d, *J* = 4.1 Hz, 1H), 6.39 – 6.30 (m, 1H), 4.32 – 4.19 (m, 1H), 3.77 (ddd, *J* = 10.8, 5.6, 4.4 Hz, 1H), 2.79 (d, *J* = 16.5 Hz, 1H), 2.54 – 2.41 (m, 1H), 2.13 (s, 6H), 1.91 (s, 6H), 1.28 (s, 9H).

^{13}C NMR (151 MHz, C_6D_6) δ 155.31 (d, *J* = 3.9 Hz), 139.29 (d, *J* = 3.7 Hz), 138.98 (d, *J* = 11.8 Hz), 138.39 (d, *J* = 12.2 Hz), 134.12 (d, *J* = 2.7 Hz), 133.95 (d, *J* = 2.4 Hz), 133.65 (d, *J* = 3.0 Hz), 133.45, 133.35, 132.81, 130.05 (d, *J* = 6.1 Hz), 130.00 (d, *J* = 5.6 Hz), 129.93, 129.88, 129.37 (d, *J* = 2.9 Hz), 128.68, 128.47, 128.31, 126.10 (d, *J* = 2.1 Hz), 80.34, 56.46 (d, *J* = 73.5 Hz), 41.17, 28.54 (3), 28.44, 21.62 (2), 21.39 (2).

^{31}P NMR (243 MHz, C_6D_6): δ 32.3 (for major rotamer), 29.2 (for minor rotamer).

HRMS (m/z) calcd for $C_{30}H_{35}ClNO_3PNa$ $[M+Na]^+$ 546.1941, found 456.1938; $[\alpha]_D^{25}$ = + 22.2 (c = 0.20, $CHCl_3$); The *ee* was determined by HPLC analysis (Chiralpak OX-RH, CH_3CN/H_2O = 35/65 v/v, 1.0 mL/min, 280 nm, t_R (major) = 12.6 min, t_R (minor) = 14.3 min), 94% *ee*.

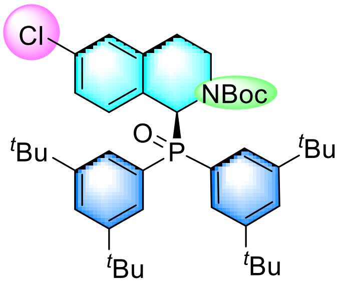

4ila, 60% yield, 96% *ee*

Major rotamer : ^{1}H NMR (600 MHz, C_6D_6) δ 8.43 (dd, *J* = 11.7, 1.7 Hz, 2H), 8.11 (dd, *J* = 11.4, 1.8 Hz, 2H), 7.68 (s, 1H), 7.58 (s, 1H), 6.89 (s, 1H), 6.65 – 6.56 (m, 2H), 6.17 (d, *J* = 8.2 Hz, 1H), 4.43 (ddd, *J* = 13.1, 9.7, 5.2 Hz, 1H), 3.83 – 3.74 (m, 1H), 2.85 (d, *J* = 16.4 Hz, 1H), 2.53 – 2.43 (m, 1H), 1.33 (s, 18H), 1.21 (s, 9H), 1.16 (s, 18H).

^{13}C NMR (151 MHz, C_6D_6) δ 155.49 (d, *J* = 4.6 Hz), 152.02 (d, *J* = 10.9 Hz), 151.39 (d, *J* = 11.3 Hz), 139.38 (d, *J* = 3.6 Hz), 133.53 (d, *J* = 3.0 Hz), 133.37, 132.77, 132.62, 131.98, 130.10, 129.86 (d, *J* = 1.4 Hz), 129.50 (d, *J* = 2.7 Hz), 128.68, 128.47, 128.31, 126.92 (d, *J* = 9.5 Hz), 126.68 (d, *J* = 2.5 Hz), 126.30 (d, *J* = 2.4 Hz), 125.97 (d, *J* = 1.9 Hz), 80.25, 56.87 (d, *J* = 73.8 Hz), 41.40, 35.63 (2), 35.46 (2), 31.91 (6), 31.68 (6), 28.62 (3), 28.37. ^{31}P NMR (243 MHz, C_6D_6): δ 32.3.

^{13}C NMR (151 MHz, Benzene-d_6) δ 80.18, 59.85 (d, *J* = 70.0 Hz), 39.81, 35.57 , 35.52 , 31.78 , 31.76 , 28.44, 28.37 .

Minor rotamer : ^{1}H NMR (600 MHz, C_6D_6) δ 8.08 (dd, J = 10.9, 1.6 Hz, 2H), 7.89 (dd, J = 11.5, 1.5 Hz, 2H), 7.71 (s, 1H), 7.58 (s, 1H), 6.76 (m, 3H), 6.08 (d, J = 8.5 Hz, 1H), 4.34 – 4.26 (m, 1H), 4.00 – 3.91 (m, 1H), 2.57 (d, J = 15.9 Hz, 1H), 2.52 – 2.45 (m, 1H), 1.26 (s, 18H), 1.21 (s, 18H), 1.18 (s, 9H).

^{13}C NMR (151 MHz, C_6D_6) δ154.07, 151.95 (d, J = 9.6 Hz), 151.46 (d, J = 11.3 Hz), 138.93 (d, J = 3.1 Hz), 133.71 (d, J = 3.1 Hz), 133.37, 132.73, 132.62, 131.98, 130.24, 129.90, 129.09 (d, J = 2.6 Hz), 128.68, 128.47, 128.31, 126.92 (d, J = 9.5 Hz), 126.59 (d, J = 2.1 Hz), 126.35 (d, J = 2.0 Hz), 125.97 (d, J = 1.9 Hz), 80.18, 59.85 (d, J = 70.0 Hz), 39.81, 35.57 (2), 35.52 (2), 31.78 (6), 31.76 (6), 28.44 (3), 28.37.

^{31}P NMR (243 MHz, C_6D_6): δ 29.2.

HRMS (m/z) calcd for $C_{42}H_{59}ClNO_3PNa$ $[M+Na]^+$ 714.3819, found 714.3820; $[\alpha]_D^{25}$ = +12.8 (c = 0.20, $CHCl_3$); The *ee* was determined by HPLC analysis (Chiralpak OX-RH, CH_3CN/H_2O = 20/80 v/v, 1.0 mL/min, 280 nm, t_R (major) = 8.0 min, t_R (minor) = 9.9 min), 96% *ee*.

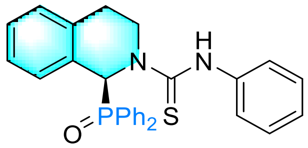

6aaa, 85% yield, 97% ee

^{1}H NMR (600 MHz, $CDCl_3$) δ 9.62 (s, 1H), 7.86 (dd, J = 11.1, 7.7 Hz, 2H), 7.73 – 7.64 (m, 2H), 7.61 (tdd, J = 7.3, 4.4, 1.4 Hz, 2H), 7.49 (dtd, J = 13.0, 7.7, 3.0 Hz, 4H), 7.31 (t, J = 7.7 Hz, 2H), 7.29 – 7.24 (m, 2H), 7.22 (tt, J = 7.5, 1.5 Hz, 1H), 7.15 (tt, J = 7.7, 1.7 Hz, 2H), 6.94 (t, J = 7.5 Hz, 2H), 6.39 (d, J = 7.7 Hz, 1H), 4.11 (dt, J = 12.9, 6.4 Hz, 1H), 3.77 – 3.63 (m, 1H), 2.93 – 2.76 (m, 1H), 2.59 (s, 1H).

^{13}C NMR (151 MHz, $CDCl_3$) δ 185.52, 140.33, 136.21, 132.86 (d, J = 2.8 Hz), 132.64 (d, J = 2.8 Hz), 132.36 (d, J = 8.8 Hz), 131.92 (d, J = 9.3 Hz), 130.33 (d, J = 95.3 Hz), 129.07, 128.74 (d, J = 8.2 Hz), 128.69 (9), 128.62, 128.24 (d, J = 2.9 Hz), 127.55 (d, J = 3.5 Hz), 126.13 (d, J = 2.2 Hz), 124.94, 124.02. 64.91 (d, J = 70.9 Hz), 45.94, 27.92.

^{31}P NMR (243 MHz, $CDCl_3$): δ 34.1.

HRMS (m/z) calcd for $C_{28}H_{25}N_2OPSNa$ $[M+Na]^+$ 491.1323, found 491.1329; $[\alpha]_D^{25}$ = +103.5 (c = 0.20, $CHCl_3$); The *ee* was determined by HPLC analysis (Chiralpak OD-RH, CH_3CN/H_2O = 40/60 v/v, 1.0 mL/min, 280 nm, t_R (major) = 4.7 min, t_R (minor) = 5.9 min), 97% *ee*.

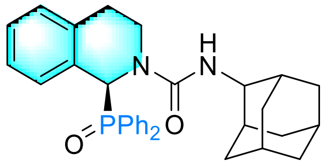

6aab, 88% yield, 99% ee

^{1}H NMR (600 MHz, $CDCl_3$) δ 8.02 – 7.90 (m, 2H), 7.89 – 7.81 (m, 2H), 7.57 – 7.52 (m, 2H), 7.52 – 7.42 (m, 4H), 7.18 – 7.11 (m, 2H), 6.90 (dt, J = 8.3, 4.2 Hz, 1H), 6.45 (t, J = 5.4 Hz, 2H), 3.78 – 3.72 (m, 1H), 3.32 (q, J = 8.6, 7.9 Hz, 2H), 2.90 – 2.71 (m, 1H), 2.03 (s, 3H), 1.81 (d, J = 14.7 Hz, 6H), 1.64 (s, 6H).

^{13}C NMR (151 MHz, Chloroform-*d*) δ 156.06, 136.55 (d, J = 3.8 Hz), 132.01 (d, J = 2.7 Hz), 131.93 , 131.88 , 131.63 (d, J = 2.7 Hz), 131.57 , 131.51 , 131.12 , 130.75 , 130.73 (d, J = 90.7 Hz), 128.52 ,

128.45 , 128.32 (d, *J* = 2.2 Hz), 128.25 , 128.18 , 127.67 (d, *J* = 2.8 Hz), 127.52 (d, *J* = 3.9 Hz), 125.87 (d, *J* = 2.2 Hz), 58.42, 51.34, 42.15 (2), 41.89, 36.39 (2), 29.52 (4), 28.01, 18.40.

^{31}P NMR (243 MHz, $CDCl_3$): δ 34.1.

HRMS (m/z) calcd for $C_{32}H_{35}N_2O_2PNa$ $[M+Na]^+$ 533.2334, found 533.2335; $[\alpha]_D^{25}$ = +63.6 (c = 0.20, $CHCl_3$); The *ee* was determined by HPLC analysis (Chiralpak OD-RH, CH_3CN/H_2O = 50/50 v/v, 1.0 mL/min, 280 nm, t_R (major) = 13.9 min, t_R (minor) = 15.6 min), 99% *ee*.

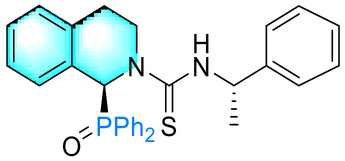

6aac, 93% yield, 98% ee

^{1}H NMR (600 MHz, $CDCl_3$) δ 8.02 – 7.96 (m, 2H), 7.96 – 7.91 (m, 2H), 7.60 (td, *J* = 7.4, 1.5 Hz, 1H), 7.57 – 7.49 (m, 4H), 7.45 (td, *J* = 7.7, 2.9 Hz, 2H), 7.35 – 7.29 (m, 4H), 7.24 (tt, *J* = 6.2, 2.2 Hz, 1H), 7.17 (tt, *J* = 7.4, 1.5 Hz, 1H), 7.13 (d, *J* = 7.5 Hz, 1H), 6.97 – 6.92 (m, 1H), 6.53 (d, *J* = 7.7 Hz, 1H), 5.58 (p, *J* = 7.1 Hz, 1H), 3.85 (dt, *J* = 11.8, 5.9 Hz, 1H), 3.70 – 3.63 (m, 1H), 3.26 (s, 1H), 2.84 (dt, *J* = 15.7, 5.7 Hz, 1H), 1.42 (d, *J* = 6.8 Hz, 3H).

^{13}C NMR (151 MHz, $CDCl_3$) δ 182.34, 142.86, 135.87, 132.44, 132.25, 132.19, 131.99, 131.72, 131.66, 128.73, 128.65, 128.29 (d, *J* = 3.0 Hz), 128.23, 128.15, 128.05 (d, *J* = 3.3 Hz), 127.50 (d, *J* = 4.3 Hz), 127.34, 126.44, 126.26 (d, *J* = 2.8 Hz), 63.62 (d, *J* = 71.1 Hz), 54.92, 44.38, 27.88, 21.47.

^{31}P NMR (243 MHz, $CDCl_3$): δ 34.2.

HRMS (m/z) calcd for $C_{30}H_{29}N_2OPSNa$ $[M+Na]^+$ 519.1630, found 519.1632; $[\alpha]_D^{25}$ = +103.5 (c = 0.20, $CHCl_3$); The *ee* was determined by HPLC analysis (Chiralpak OJ-RH, CH_3CN/H_2O = 50/50 v/v, 1.0 mL/min, 280 nm, t_R (major) = 6.6 min, t_R (minor) = 7.9 min), 98% *ee*.

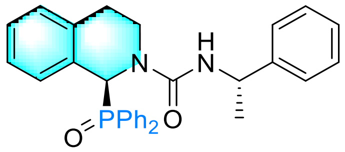

6aad, 96% yield, 98% ee

^{1}H NMR (600 MHz, $CDCl_3$) δ 7.89 – 7.81 (m, 2H), 7.78 – 7.70 (m, 2H), 7.48 – 7.39 (m, 2H), 7.36 (td, *J* = 7.7, 2.6 Hz, 4H), 7.20 – 7.12 (m, 4H), 7.12 – 7.07 (m, 1H), 7.06 – 6.99 (m, 2H), 6.83 – 6.74 (m, 1H), 6.35 (d, *J* = 6.8 Hz, 2H), 4.70 (s, 1H), 3.65 (dt, *J* = 10.9, 5.5 Hz, 1H), 3.25 (td, *J* = 10.3, 9.7, 5.6 Hz, 1H), 3.16 (s, 1H), 2.69 (dt, *J* = 15.4, 5.4 Hz, 1H), 1.19 (d, *J* = 6.8 Hz, 3H).

^{13}C NMR (151 MHz, $CDCl_3$) δ 156.62, 143.90, 136.21 (d, *J* = 3.7 Hz), 132.10 (d, *J* = 2.6 Hz), 131.99, 131.93, 131.72 (d, *J* = 2.7 Hz), 131.64, 131.58, 131.10, 130.52, 130.44 (d, *J* = 91.2 Hz), 128.55 (2), 128.47, 128.28 (d, *J* = 2.3 Hz), 128.21, 128.13, 127.75 (d, *J* = 2.7 Hz), 127.48 (d, *J* = 3.9 Hz), 127.10, 126.03 (2), 125.97 (d, *J* = 2.1 Hz), 58.36 , 50.28 , 41.57, 28.01, 22.44, 18.39 .

^{31}P NMR (243 MHz, $CDCl_3$): δ 34.0.

HRMS (m/z) calcd for $C_{30}H_{29}N_2O_2PNa$ $[M+Na]^+$ 503.1859, found 503.1865; $[\alpha]_D^{25}$ = +83.5 (c = 0.20, $CHCl_3$); The *ee* was determined by HPLC analysis (Chiralpak AS-RH, CH_3CN/H_2O = 50/50 v/v, 1.0 mL/min, 280 nm, t_R (major) = 4.1 min, t_R (minor) = 4.8 min), 98% *ee*.

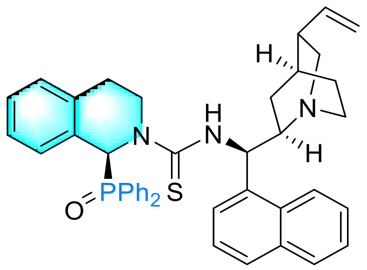

6aae, 86% yield, 99% ee

^{1}H NMR (600 MHz, $CDCl_3$) δ 8.68 (d, J = 4.5 Hz, 1H), 8.17 – 7.98 (m, 4H), 7.84 – 7.74 (m, 3H), 7.67 (d, J = 41.0 Hz, 2H), 7.56 – 7.50 (m, 1H), 7.46 (td, J = 7.7, 2.8 Hz, 2H), 7.38 (dd, J = 9.2, 2.6 Hz, 1H), 7.29 (s, 1H), 7.22 – 7.14 (m, 2H), 7.11 – 6.89 (m, 3H), 6.48 (d, J = 7.6 Hz, 1H), 5.89 (ddd, J = 16.9, 10.6, 5.9 Hz, 1H), 5.14 (t, J = 12.7 Hz, 2H), 4.11 (s, 1H), 3.96 (s, 2H), 3.82 (s, 1H), 3.47 (s, 1H), 2.95 (t, J = 28.4 Hz, 6H), 2.32 (s, 1H), 1.68 (s, 1H), 1.57 (s, 1H), 1.51 – 1.37 (m, 2H), 1.32 – 1.18 (m, 1H), 0.99 – 0.81 (m, 1H).

^{13}C NMR (151 MHz, $CDCl_3$) δ 179.71, 155.74, 145.77, 137.93, 133.59, 130.22, 129.98, 129.65, 129.42, 128.83 (6), 126.80, 126.72 (6), 125.91 (3), 125.31, 124.42 (d, J = 2.2 Hz), 120.26, 116.55, 113.30, 100.05, 60.99 (d, J = 70.9 Hz), 60.16, 53.98, 53.58, 47.18, 44.83, 41.60, 36.71, 26.22, 25.16, 24.50.

^{31}P NMR (243 MHz, $CDCl_3$): δ 34.9.

HRMS (m/z) calcd for $C_{42}H_{42}N_3OPSNa$ $[M+Na]^+$ 690.2684, found 690.2688; $[\alpha]_D^{25}$ = +153.5 (c = 0.20, $CHCl_3$); The *ee* was determined by HPLC analysis (Chiralpak AS-RH, CH_3CN/H_2O = 50/50 v/v, 1.0 mL/min, 280 nm, t_R (major) = 4.8 min, t_R (minor) = 5.3 min), 99% *ee*.

6. DFT Calculations

DFT calculations were performed using the Gaussian 16 package.[1] All the structures were optimized at the B3LYP/6-31G* level,2-5 which has been found to produce reliable results for the phosphoric acid catalyzed reactions.[6-7] Vibrational analyses were performed to ensure intermediates to have no imaginary frequencies and the transition state structures to have only one imaginary frequency. Transition state structures were confirmed to connect appropriate reactants or products by intrinsic reaction coordinate (IRC) calculations.[8] Solvent effects were considered using diisopropylether with the SMD[9] model at the M06-2X/6-311++G** level.[5, 10-13]. We also performed single-point energy calculations using various methods including M062X-D3, B3LYP, B3LYP-D3 and B3LYP-D3(BJ) with basis set 6-311++G** for the transition states **TS5(S)** and **TS5(R)** presented in **Figure 3** in the main text.

Supplementary Table 3. The energies of calculated structures.

	E(gas)	G(gas)	E(sol)	G(sol)
**CPA7**	-2493.7487	-2493.0974	-2493.5260	-2492.8747
**1a**	-403.1082	-402.9808	-403.0521	-402.9247
**$(BOC)_2O$**	-768.0602	-767.8272	-767.9921	-767.7591
**TS1**	-3664.9223	-3663.8675	-3664.5868	-3663.5320
**1a-1**	-1171.2032	-1170.8167	-1171.0885	-1170.7020
**TS2**	-3664.9511	-3663.8968	-3664.6145	-3663.5603
**1a-2**	-3664.9540	-3663.8991	-3664.6205	-3663.5656
**TS3**	-3664.9250	-3663.8659	-3664.5874	-3663.5283
**CO_2**	-188.5776	-188.5867	-188.5755	-188.5846

**1a'**	-982.6266	-982.2504	-982.5128	-982.1367
**Ph$_2$P(O)H**	-880.4756	-880.3190	-880.4059	-880.2492
**TS4**	-4356.8524	-4355.6245	-4356.4628	-4355.2349
**1a'-1**	-4123.1926	-4122.0932	-4122.8304	-4121.7310
**TS5(*S*)**	-4123.1743	-4122.0767	-4122.8162	-4121.7186
**TS5(*R*)**	-4123.1731	-4122.0738	-4122.8134	-4121.7140
**^{t}BuOH**	-233.6667	-233.5596	-233.6385	-233.5314

Supplementary Table 4. Comparison of results from single-point energy calculations using different DFT methods for the relative energy difference of **TS5(*S*)** and **TS5(*R*)**.

	B3LYP[a)]	B3LYP	B3LYP-D3	B3LYP-D3(BJ)	M062X[b)]	M062X-D3	Experimental
ΔΔ*G**[c)]	1.9	2.6	3.0	2.1	2.9	3.2	1.8
ee	91.6%	97.5%	98.7%	94.1%	98.4%	99.1%	91%

a) data from gas phase calculation.

b) data given in the main text.

c) ΔΔG* = ΔG***TS5(*R*) -** ΔG***TS5(*S*)**, Relative free energies are given in kcal/mol.

Remarks on **Supplementary Table 4**:

The results shown in **Supplementary Table 4** indicate that different DFT methods give approximately consistent results and correctly reproduce the enantiomeric preference observed experimentally.

Supplementary Table 5. Comparison of relative energy difference of **TS5(*S*)** and **TS5(*R*)** optimized by B3LYP and B3LYP-D3. Relative free energies are given in kcal/mol.

	B3LYP	B3LYP-D3
ΔΔ*G**	1.9	1.7
ee (%)	91.6	89.5

7. Supplementary References

1. Gaussian 16, Frisch, M. J.; Trucks, G. W.; Schlegel, H. B.; Scuseria, G. E.; Robb, M. A.; Cheeseman, J. R.; Scalmani, G.; Barone, V.; Petersson, G. A.; Nakatsuji, H.; Li, X.; Caricato, M.; Marenich, A. V.; Bloino, J.; Janesko, B. G.; Gomperts, R.; Mennucci, B.; Hratchian, H. P.; Ortiz, J. V.; Izmaylov, A. F.; Sonnenberg, J. L.; Williams-Young, D.; Ding, F.; Lipparini, F.; Egidi, F.; Goings, J.; Peng, B.; Petrone, A.; Henderson, T.; Ranasinghe, D.; Zakrzewski, V. G.; Gao, J.; Rega, N.; Zheng, G.; Liang, W.; Hada, M.; Ehara, M.; Toyota, K.; Fukuda, R.; Hasegawa, J.; Ishida, M.; Nakajima, T.; Honda, Y.; Kitao, O.; Nakai, H.; Vreven, T.; Throssell, K.; Montgomery, J. A., Jr.; Peralta, J. E.; Ogliaro, F.; Bearpark, M. J.; Heyd, J. J.; Brothers, E. N.; Kudin, K. N.; Staroverov, V. N.; Keith, T. A.; Kobayashi, R.; Normand, J.; Raghavachari, K.; Rendell, A. P.; Burant, J. C.; Iyengar, S. S.; Tomasi, J.; Cossi, M.; Millam, J. M.; Klene, M.; Adamo, C.; Cammi, R.; Ochterski, J. W.; Martin, R. L.; Morokuma, K.; Farkas, O.; Foresman, J. B.; Fox, D. J. Gaussian, Inc., Wallingford CT, 2016.

2. Becke, A. D. Density-functional exchange-energy approximation with correct asymptotic-behavior. *Phys. Rev. A.* **38**, 3098-3100 (1988).

3. Lee, C.; Yang, W.; Parr, R. G. Development of the Colle-Salvetti correlation-energy formula into a functional of the electron density. *Phys. Rev. B.* **37**, 785-789 (1988).

4. Miehlich, B.; Savin, A.; Stoll, H.; Preuss, H. Results obtained with the correlation-energy density functionals of Becke and Lee, Yang and Parr. *Chem. Phys. Lett.* **157**, 200-206 (1989).

5. Petersson, G. A.; Bennett, A.; Tensfeldt, T. G.; Al-Laham, M. A.; Shirley, W. A.; Mantzaris, J. A complete basis set model chemistry. I. The total energies of closed-shell atoms and hydrides of the first-row atoms. *J. Chem. Phys*. **89**, 2193-2218 (1988).

6. Yan, J.; Zhang, Z.; Chen, M.; Lin, Z. Sun, J. A Study of the Reactivity of (Aza-) Quinone Methides in Selective C6-Alkylations of Indoles. *ChemCatChem* **12**, 5053-5057 (2020).

7. Zhang, J.; Yu, P.; Li, S.Y.; Sun, H.; Xiang, S.H.; Wang, J.J.; Houk, K.N.; Tan, B.; Asymmetric phosphoric acid–catalyzed four-component Ugi reaction. *Science* **361**, eaas8707 (2018).

8. Fukui, K. The path of chemical reactions-the IRC approach. Acc. Chem. Res. 1981, 14, 363–368.

9. Marenich, A. V.; Cramer, C. J.; Truhlar, D. G. Universal solvation model based on solute electron density and on a continuum model of the solvent defined by the bulk dielectric constant and atomic surface tensions. *J. Phys. Chem. B* **113**, 6378– 6396 (2009).

10. Zhao, Y.; Truhlar, D. G. The M06 suite of density functionals for main group thermochemistry, thermochemical kinetics, noncovalent interactions, excited states, and transition elements: two new functionals and systematic testing of four M06-class functionals and 12 other functionals. *Theor. Chem. Acc*. **120**, 215-241 (2008).
11. Raghavachari, K.; Binkley, J. S.; Seeger, R.; Pople, J. A. Self-Consistent Molecular Orbital Methods. 20. Basis set for correlated wave-functions. *J. Chem. Phys*. **72**, 650-654 (1980).
12. Clark, T.; Chandrasekhar, J.; Spitznagel, G. W.; Schleyer, P. v. R. Efficient diffuse function-augmented basis-sets for anion calculations. 3. The 3-21+G basis set for 1st-row elements, Li-F. J. *Comp. Chem*. **4**, 294-301 (1983).
13. Frisch, M. J.; Pople, J. A.; Binkley, J. S. Self-Consistent Molecular Orbital Methods. 25. Supplementary Functions for Gaussian Basis Sets. *J. Chem. Phys*. **80**, 3265-3269 (1984).
