# Supplementary material for: Chiral phosphoric acid-catalyzed enantioselective phosphinylation of 3,4-dihydroisoquinolines with diarylphosphine oxides
Source: Commun Chem. 2023 Feb 9;6:26. doi: 10.1038/s42004-023-00826-4 (PMC9911717; doi:10.1038/s42004-023-00826-4)
Supplement: Supplementary file 3 — Description of Additional Supplementary Files [file 42004_2023_826_MOESM3_ESM.pdf]

Description of Additional Supplementary Files

File name: Supplementary Data 1

Description: NMR spectra

File name: Supplementary Data 2

Description: HPLC chromatogram

File name: Supplementary Data 3

Description: Cartesian coordination of the key structures
