# Supplementary material for: Chiral phosphoric acid-catalyzed enantioselective phosphinylation of 3,4-dihydroisoquinolines with diarylphosphine oxides
Source: Commun Chem. 2023 Feb 9;6:26. doi: 10.1038/s42004-023-00826-4 (PMC9911717; doi:10.1038/s42004-023-00826-4)
Supplement: Supplementary file 4 — Supplementary data 1 [file 42004_2023_826_MOESM4_ESM.pdf]

Supplementary Data 1.

The spectra of NMR

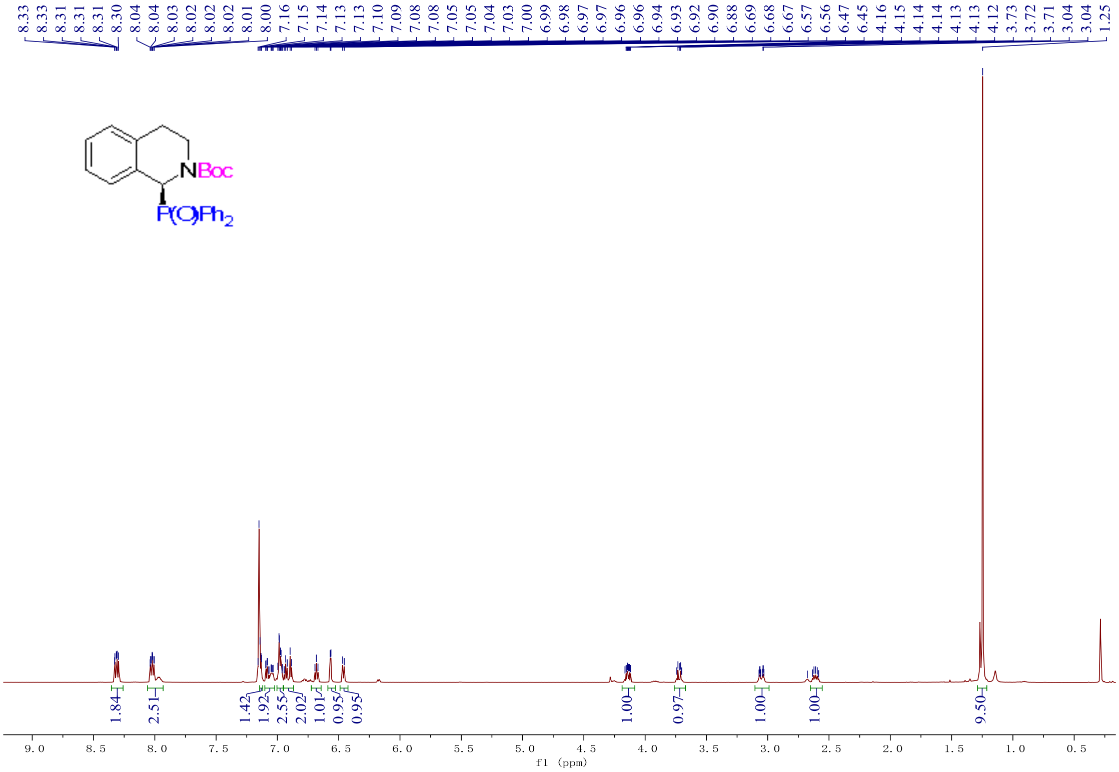

^{1}H NMR of compound **4aaa**

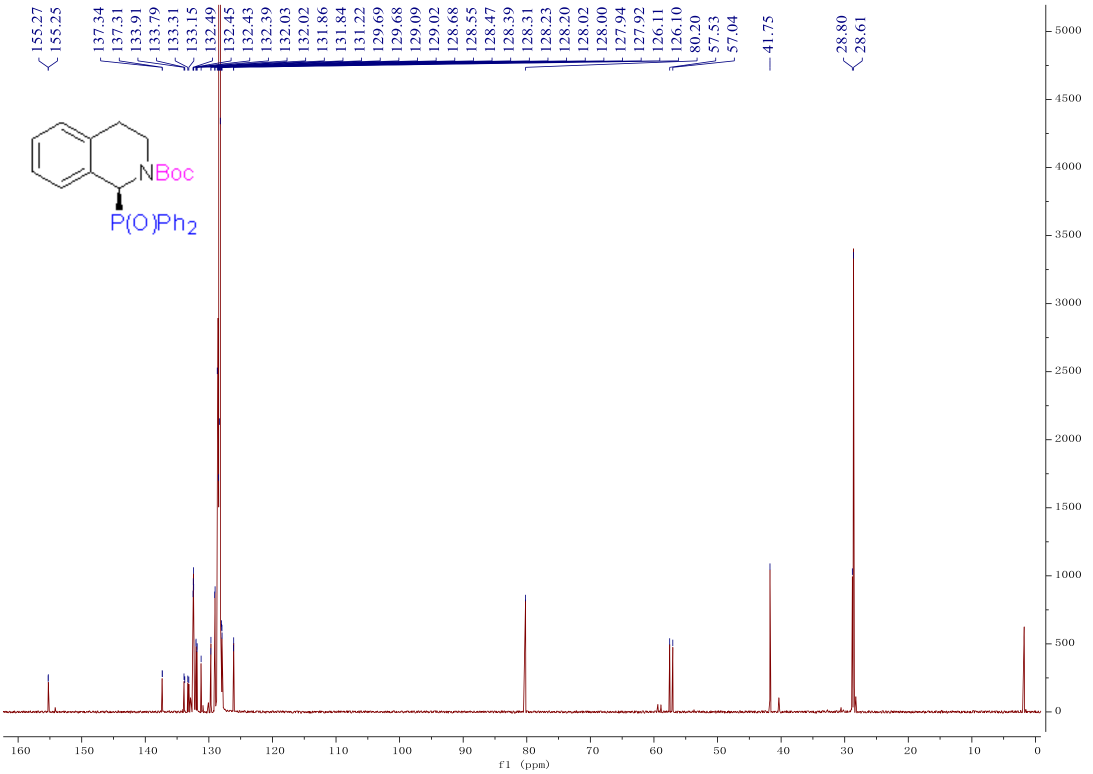

^{13}C NMR of compound **4aaa**

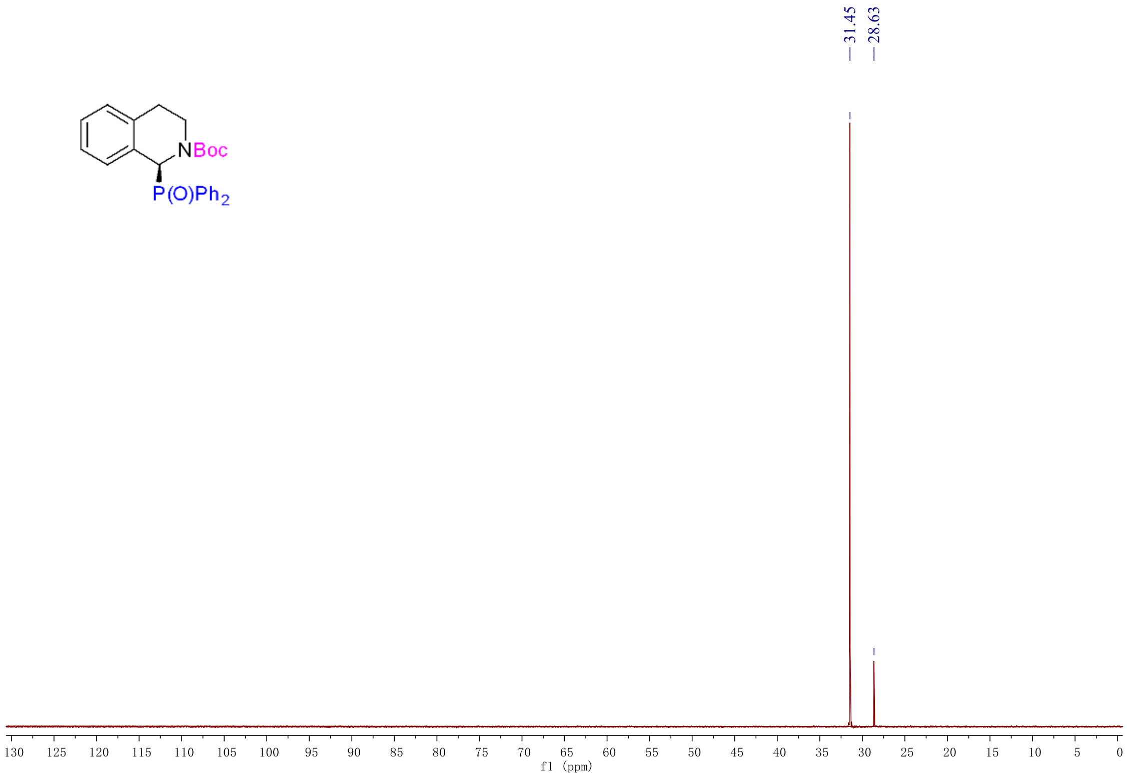

^{31}P NMR of compound **4aaa**

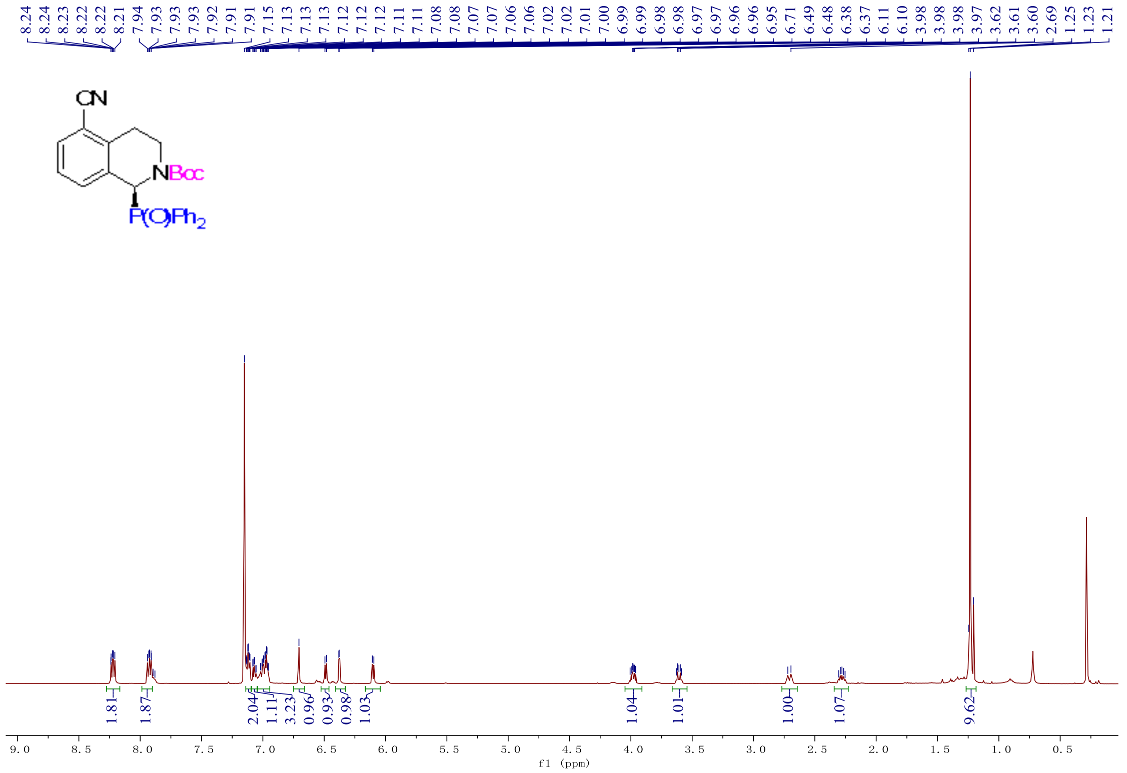

^{1}H NMR of compound **4baa**

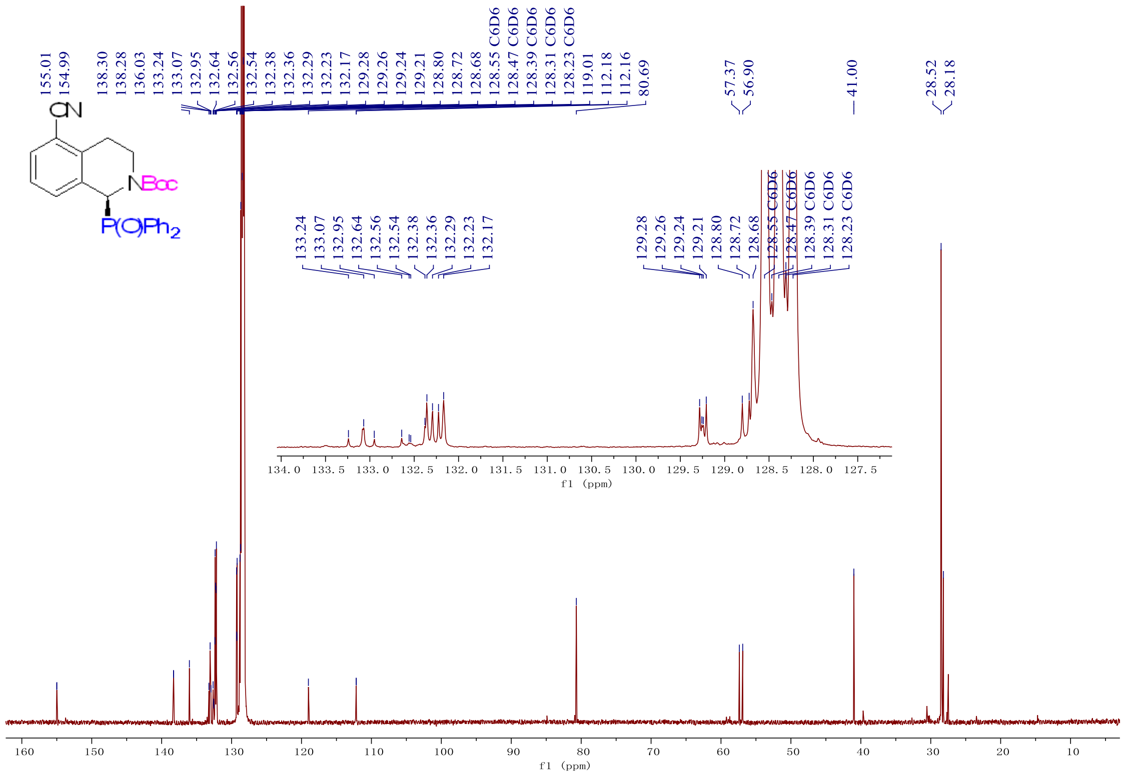

^{13}C NMR of compound **4baa**

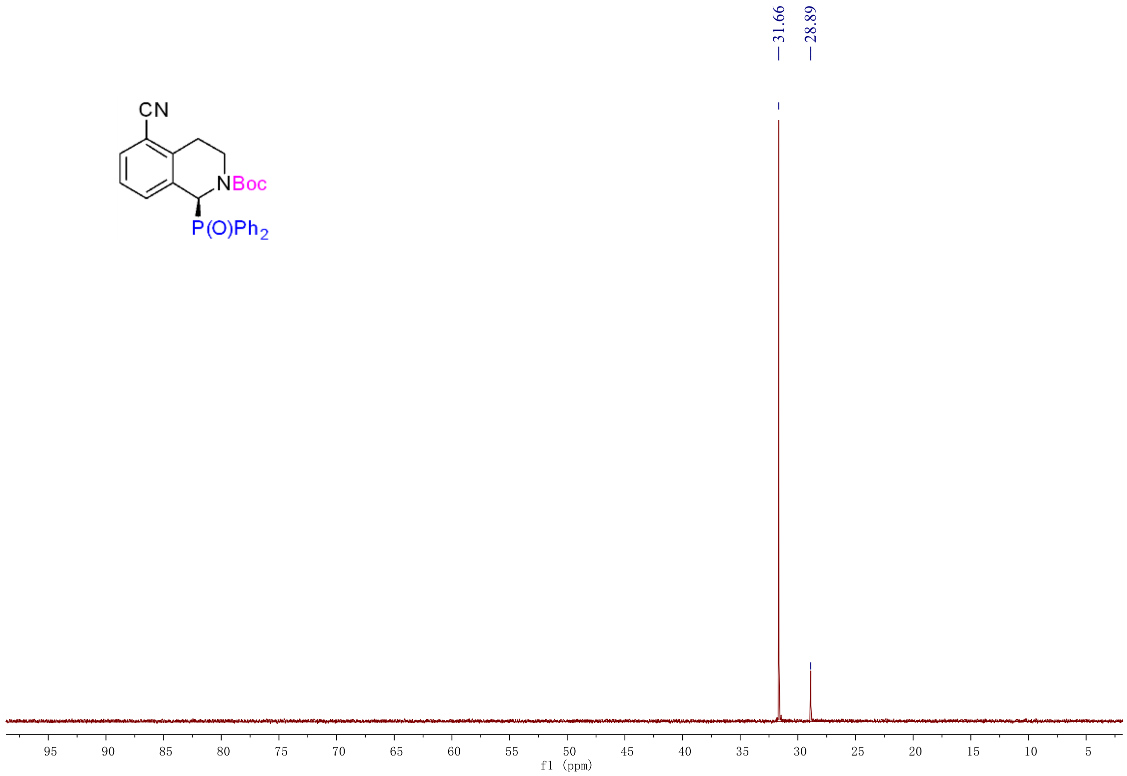

^{31}P NMR of compound **4baa**

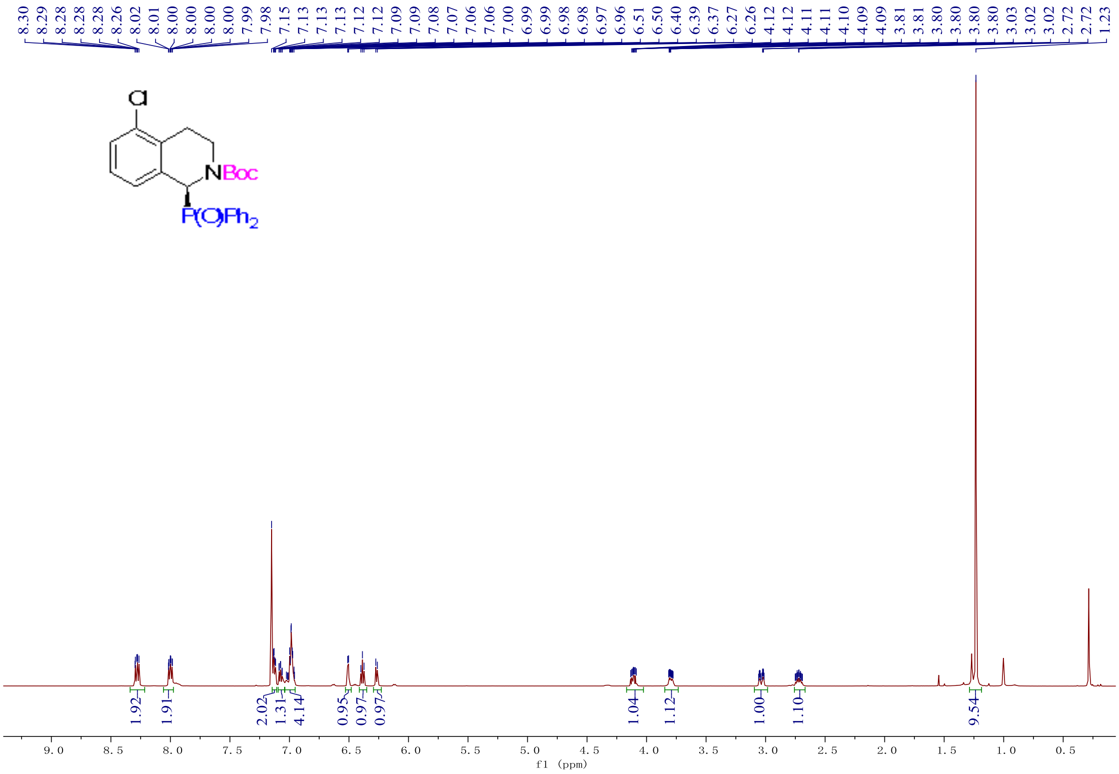

^{1}H NMR of compound **4caa**

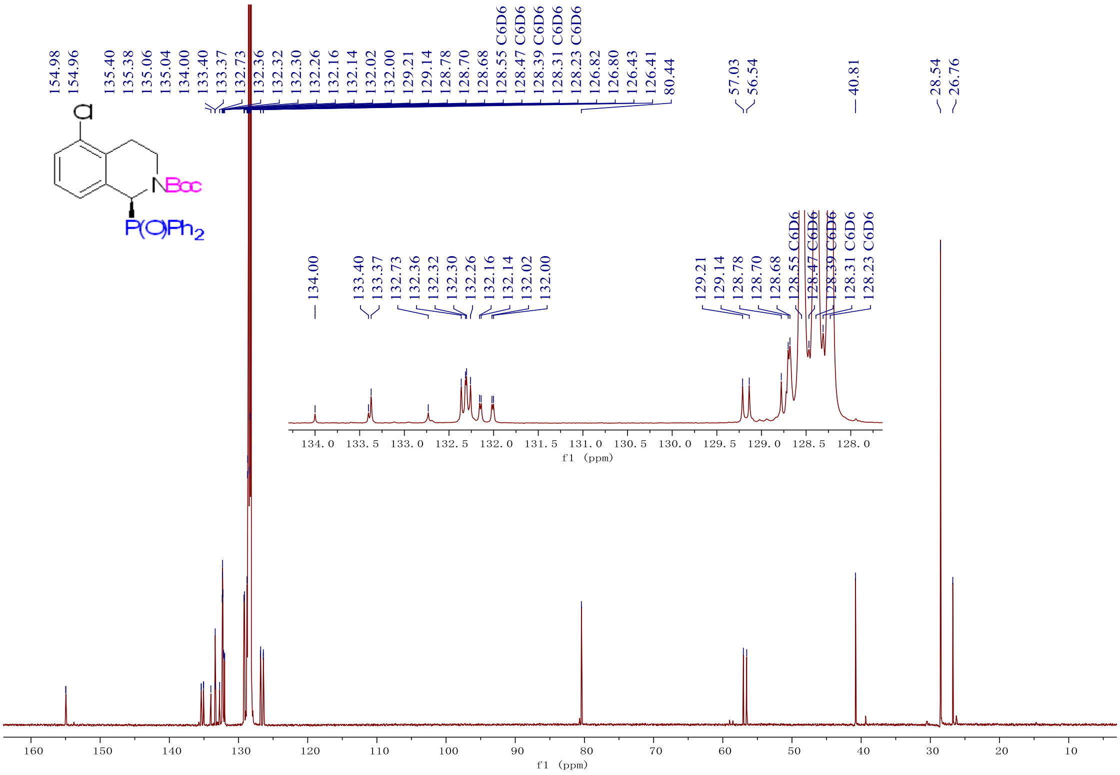

^{13}C NMR of compound **4caa**

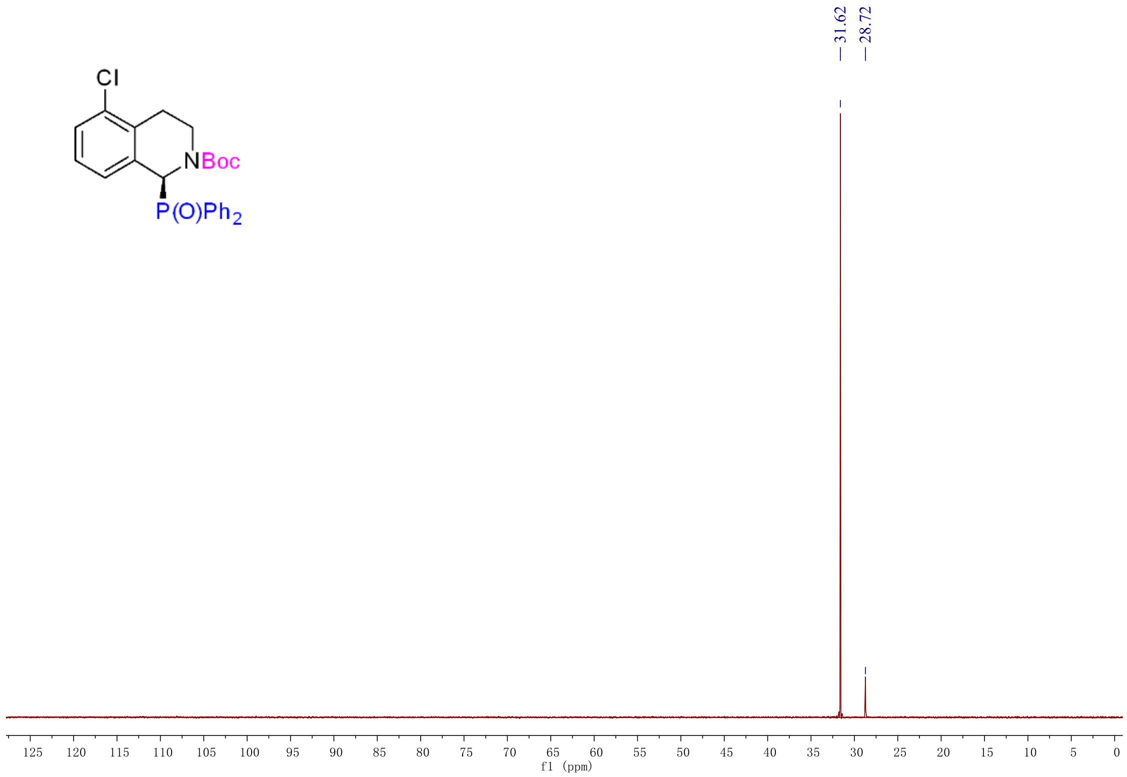

^{31}P NMR of compound **4caa**

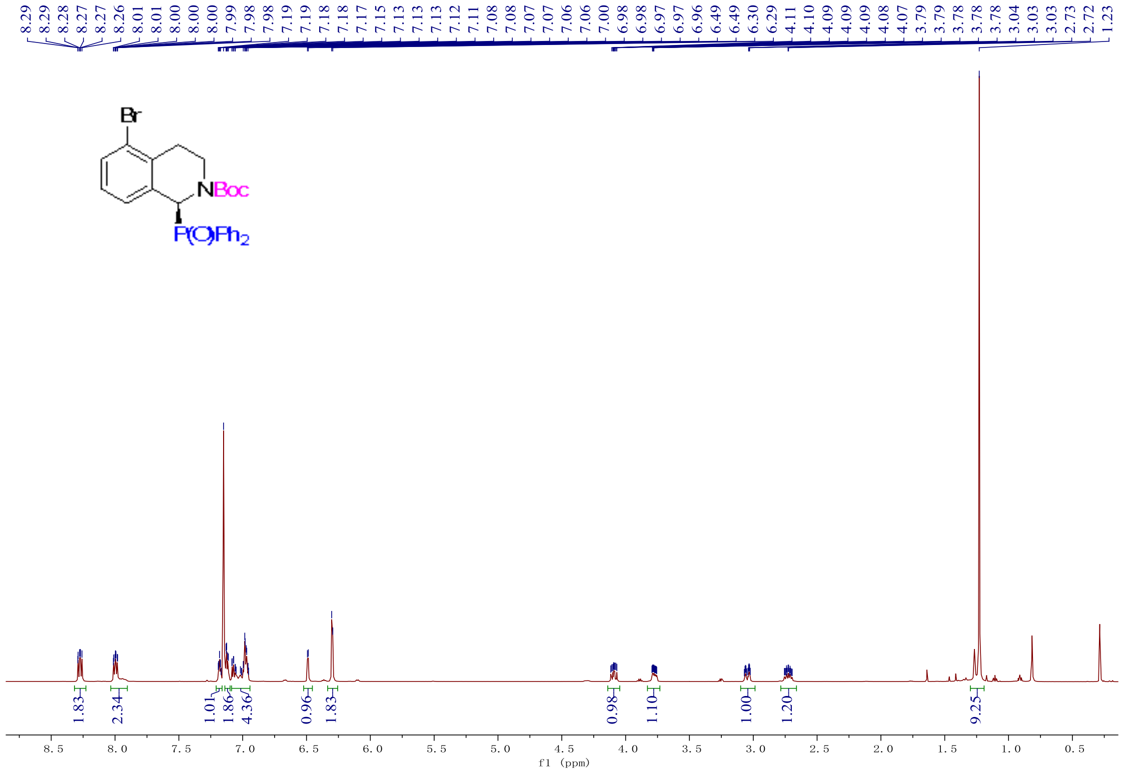

^{1}H NMR of compound **4daa**

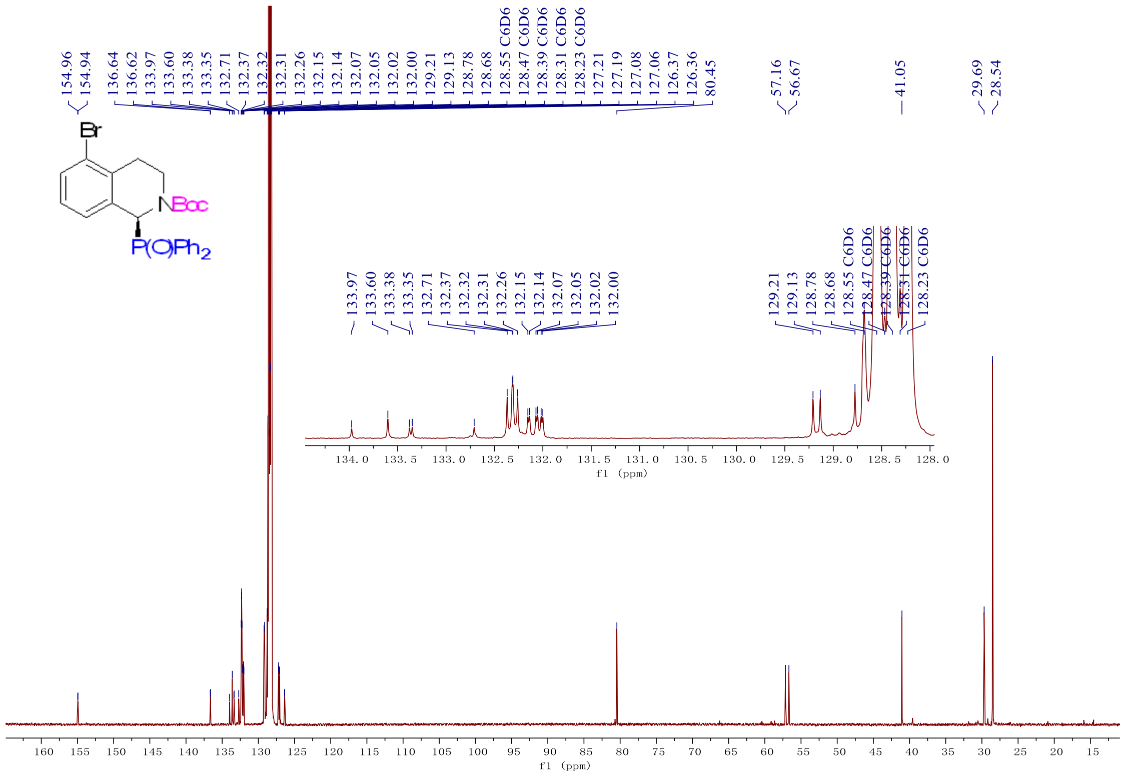

^{13}C NMR of compound **4daa**

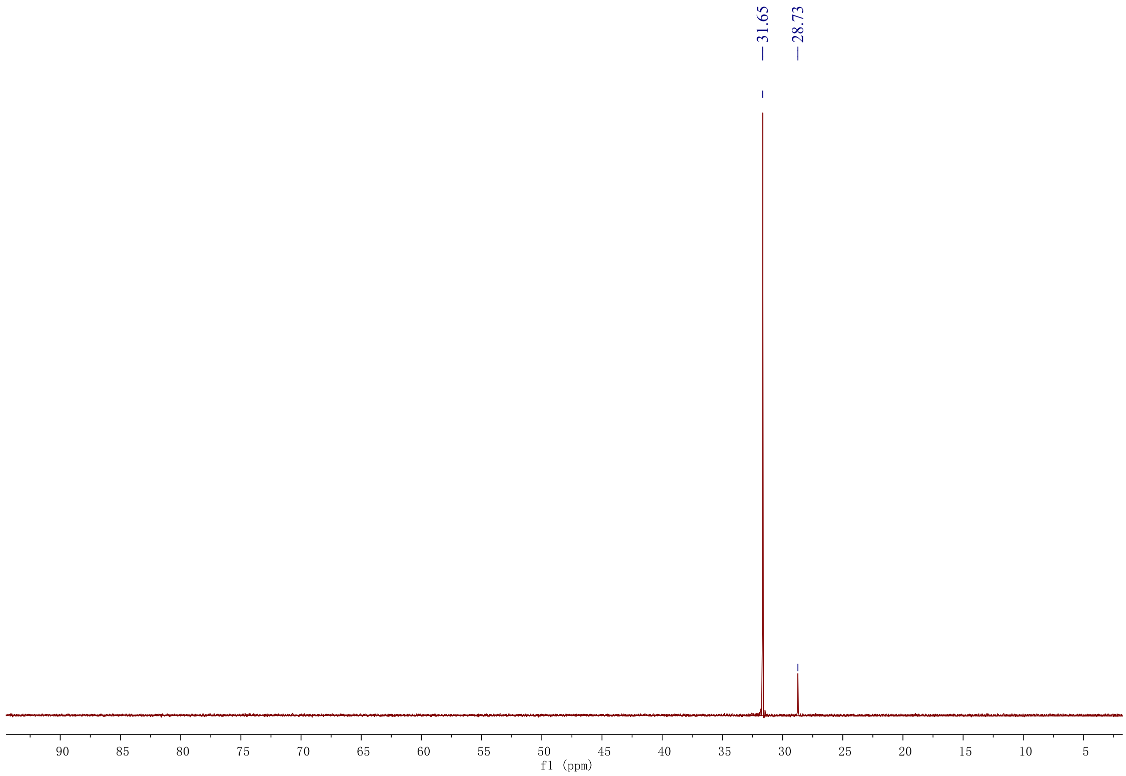

^{31}P NMR of compound **4daa**

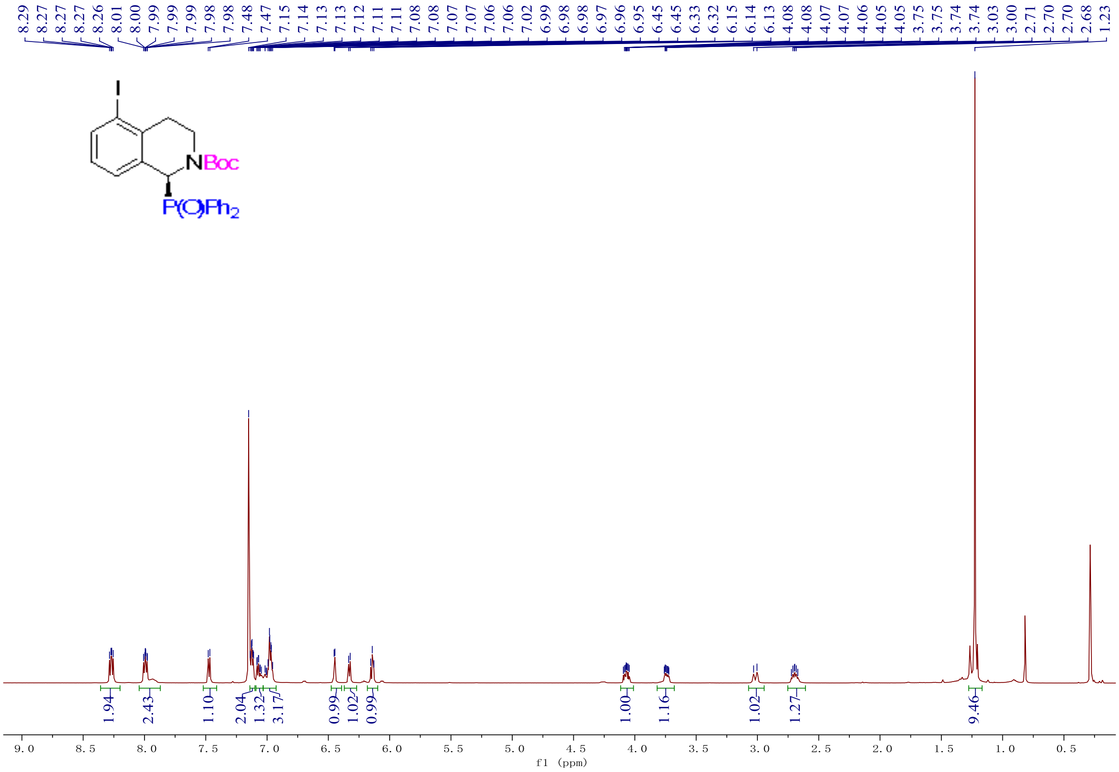

^{1}H NMR of compound **4eaa**

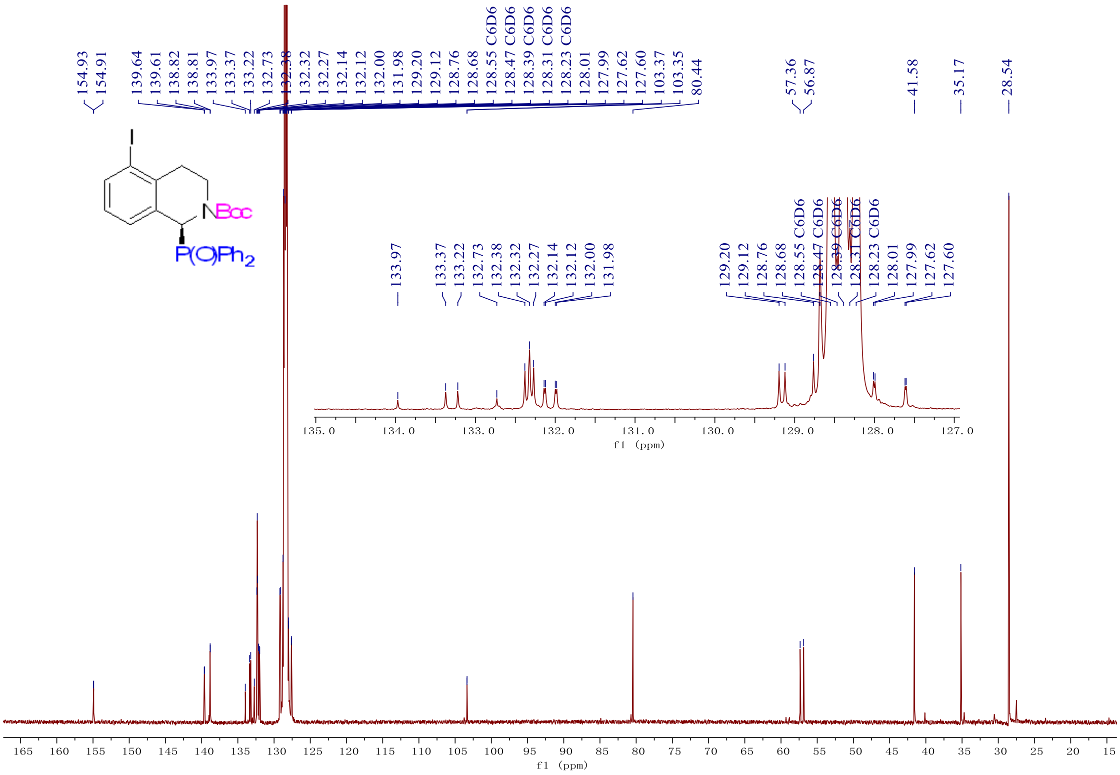

^{13}C NMR of compound **4eaa**

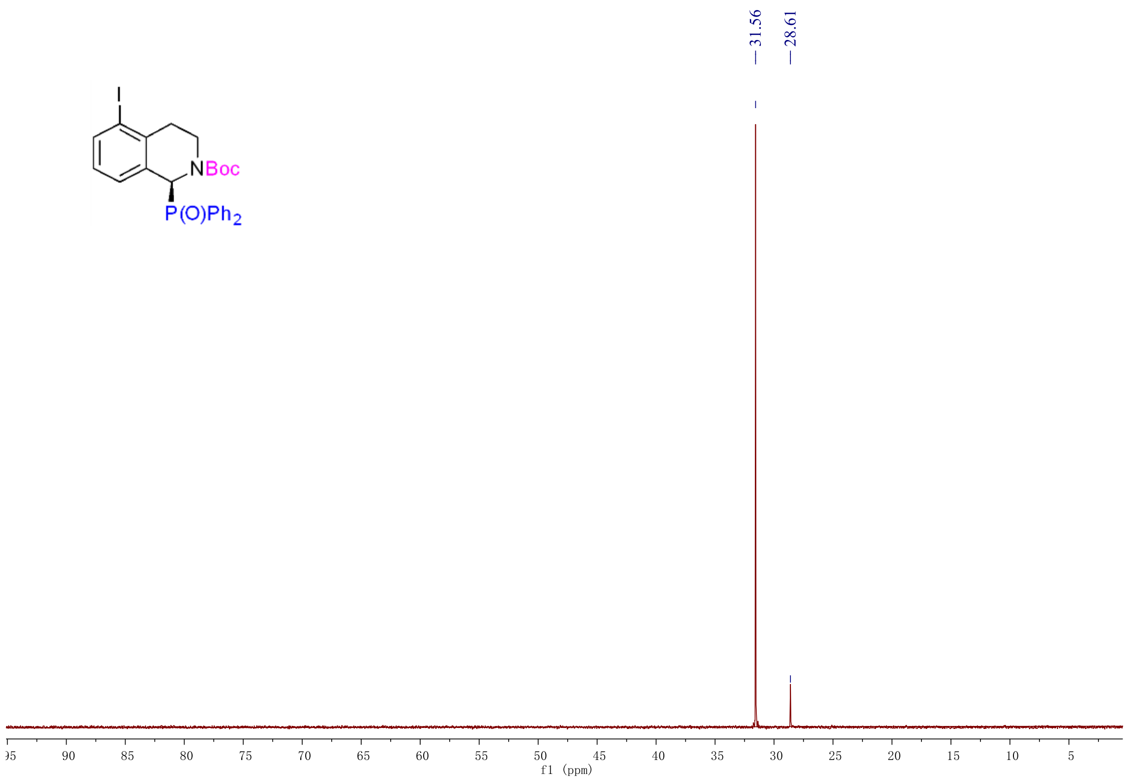

^{31}P NMR of compound **4eaa**

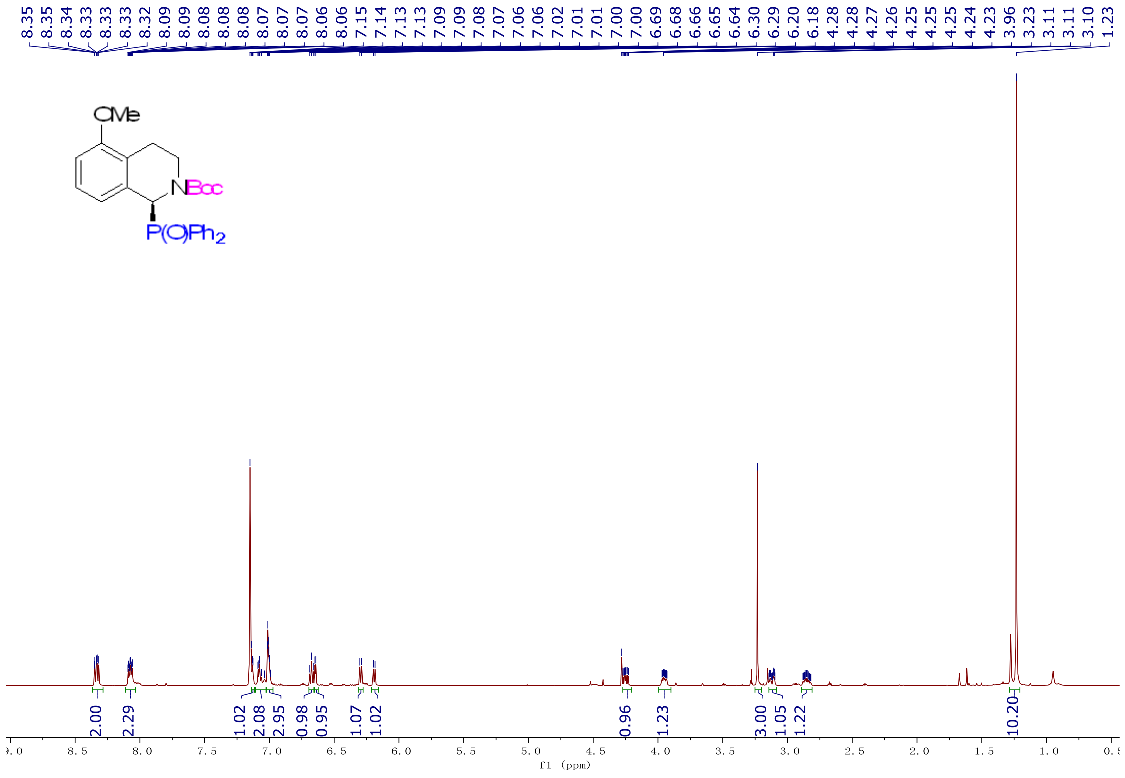

^{1}H NMR of compound **4faa**

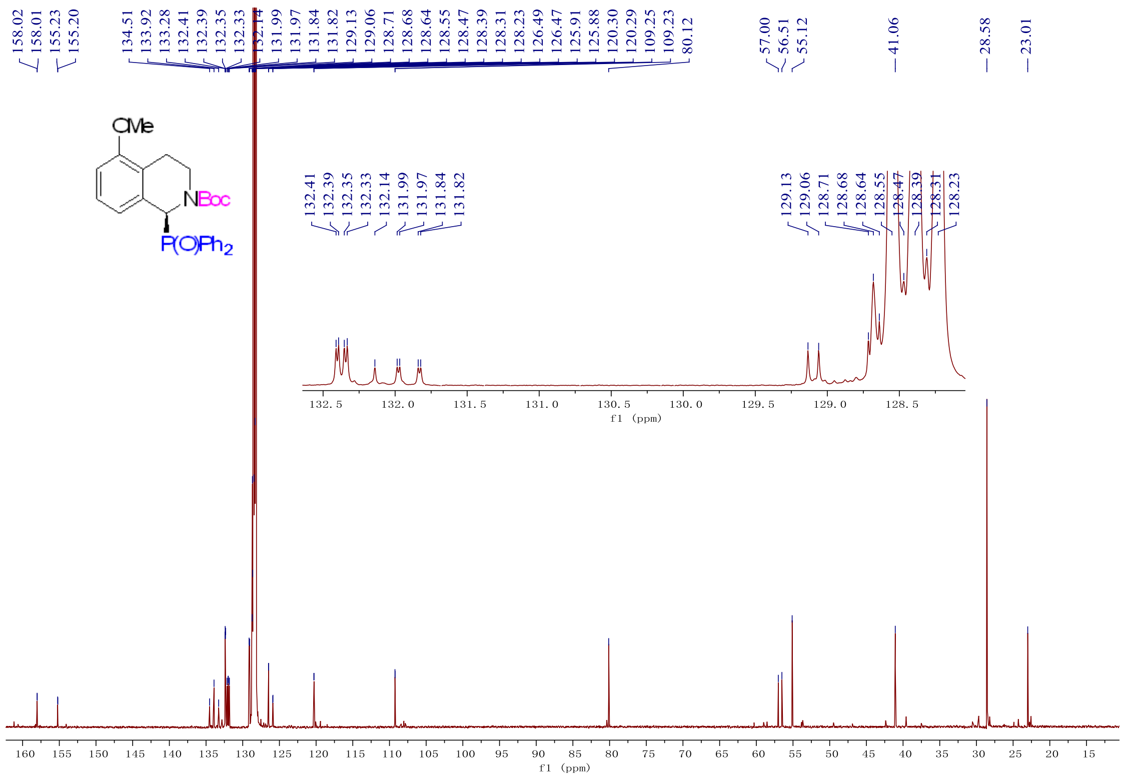

^{13}C NMR of compound **4faa**

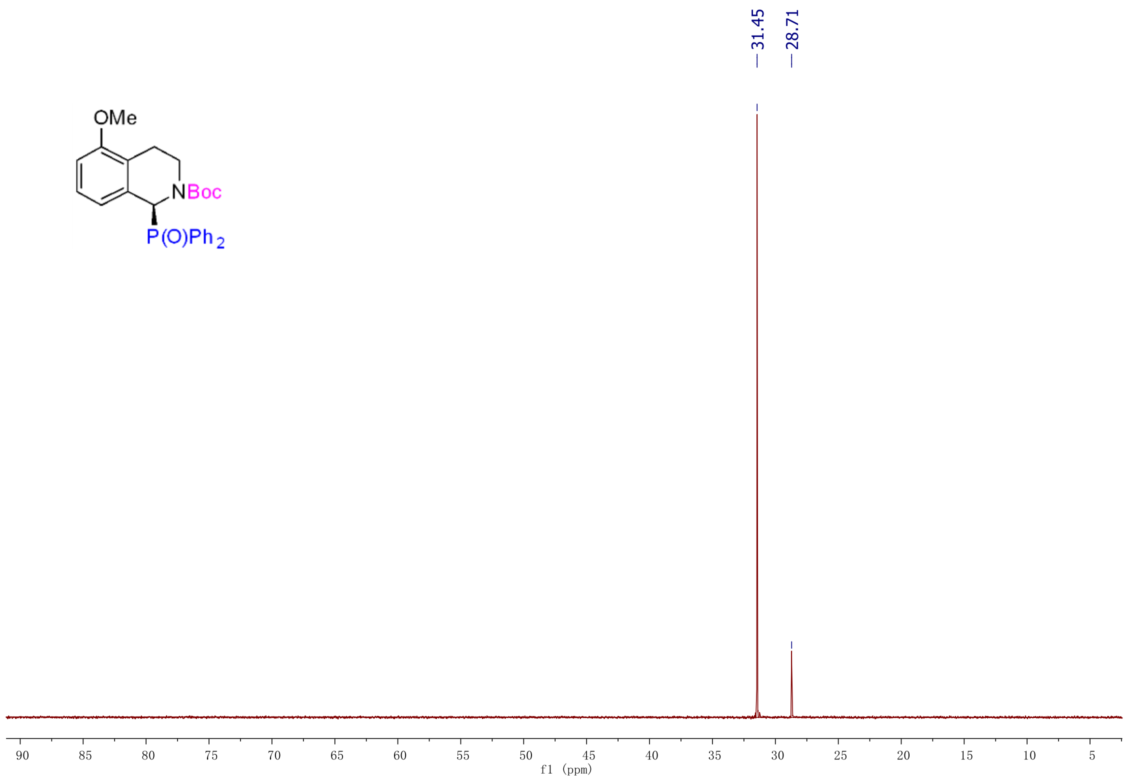

^{31}P NMR of compound **4faa**

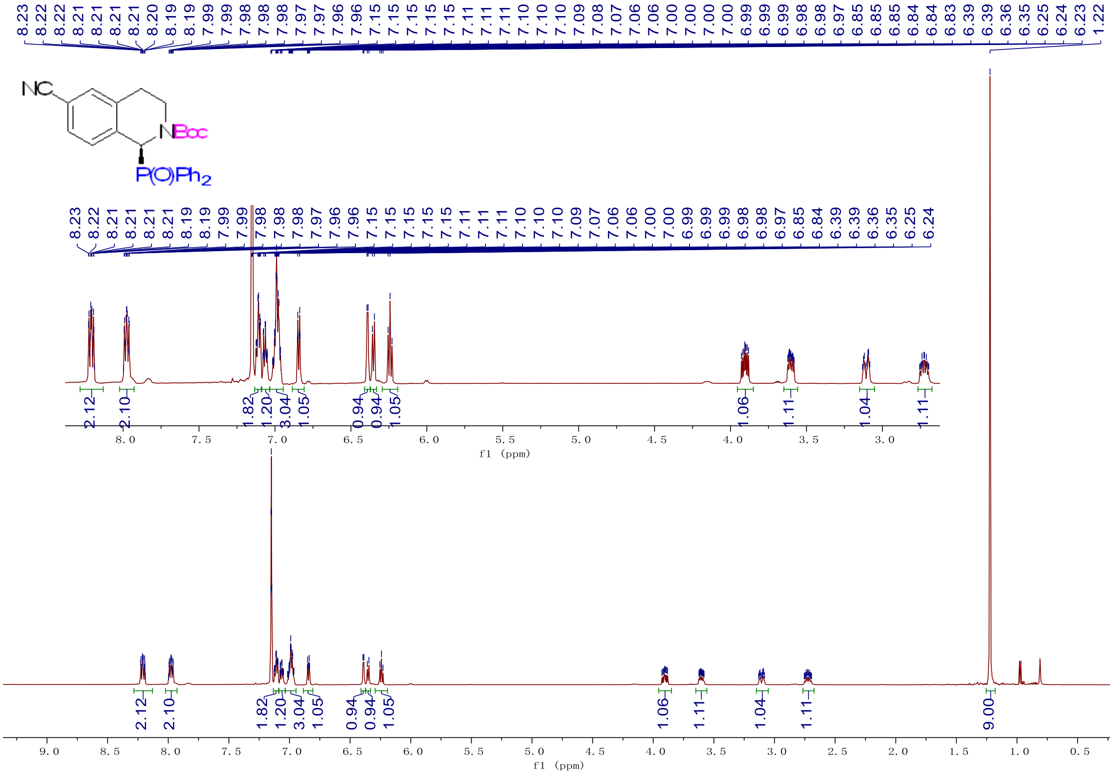

^{1}H NMR of compound **4gaa**

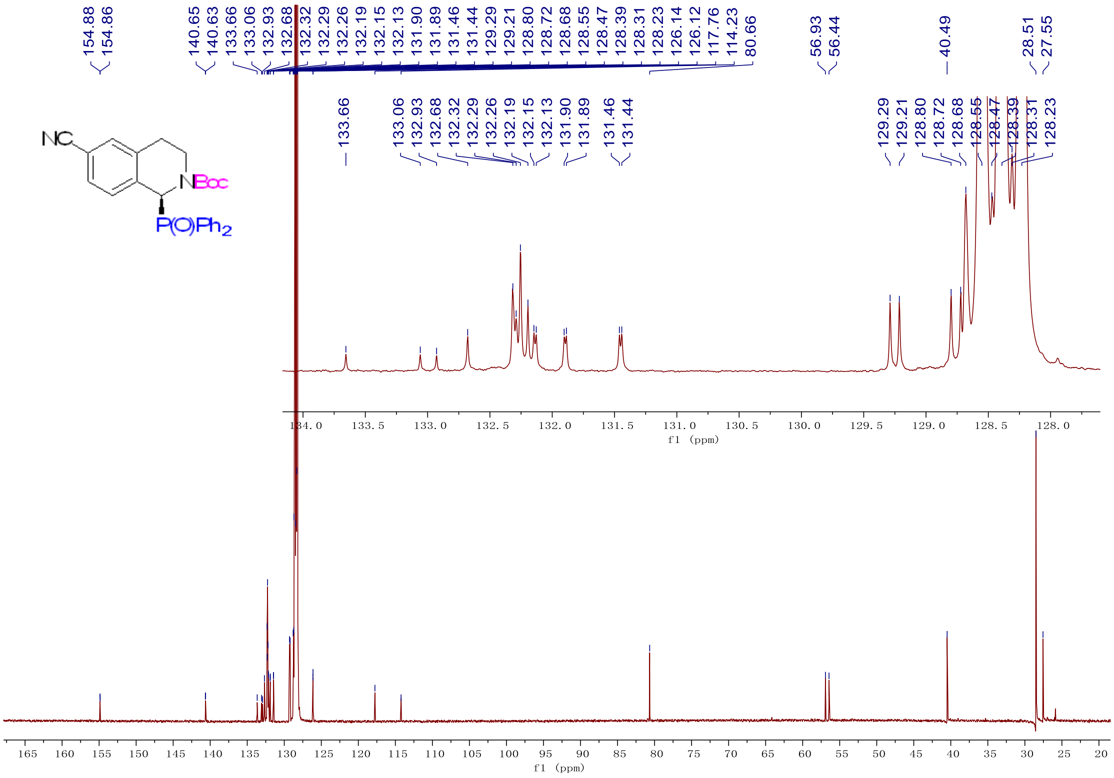

^{13}C NMR of compound **4gaa**

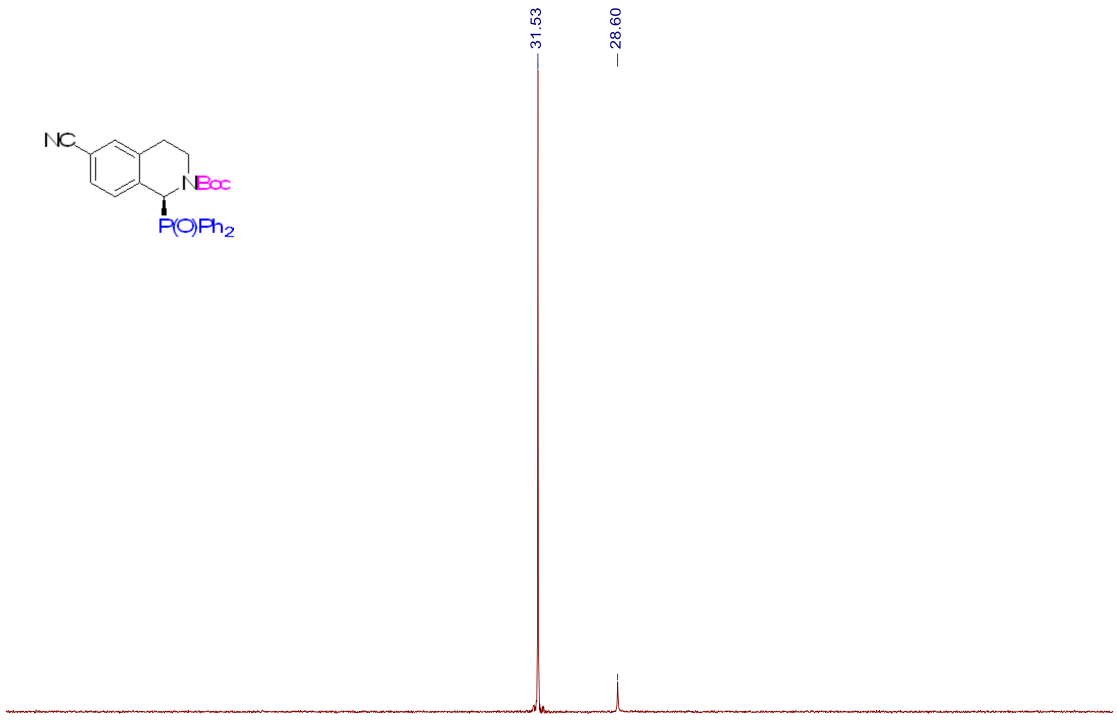

^{31}P NMR of compound **4gaa**

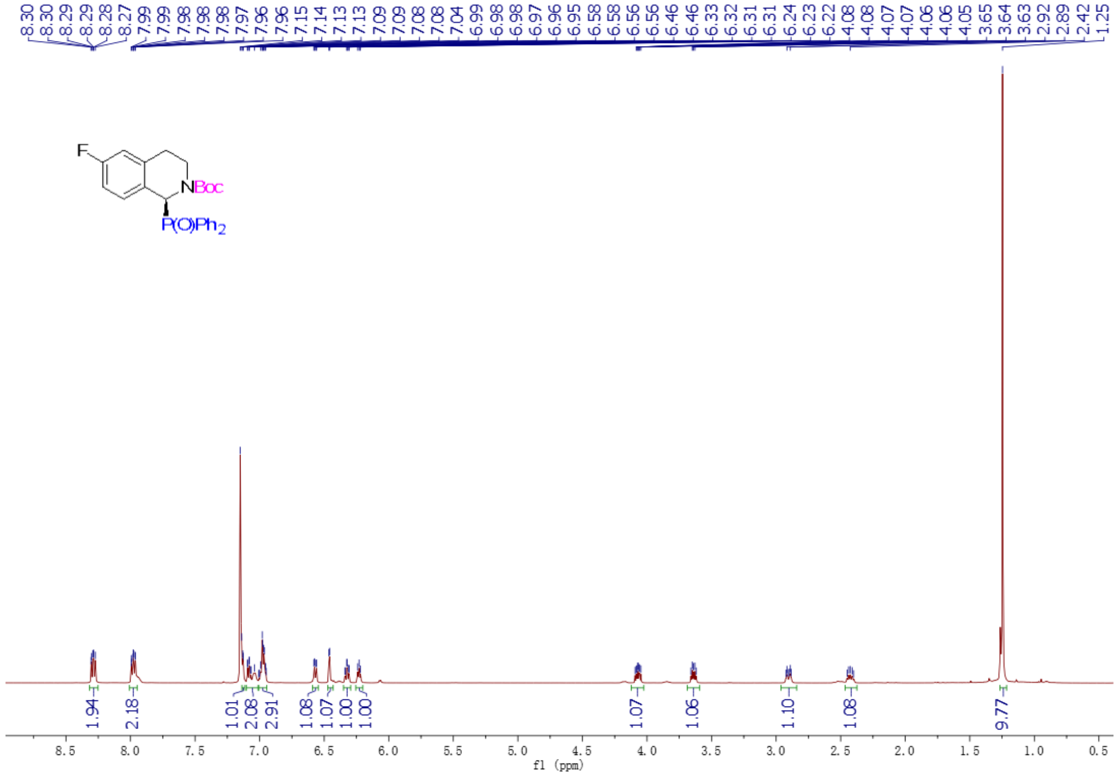

^{1}H NMR of compound **4haa**

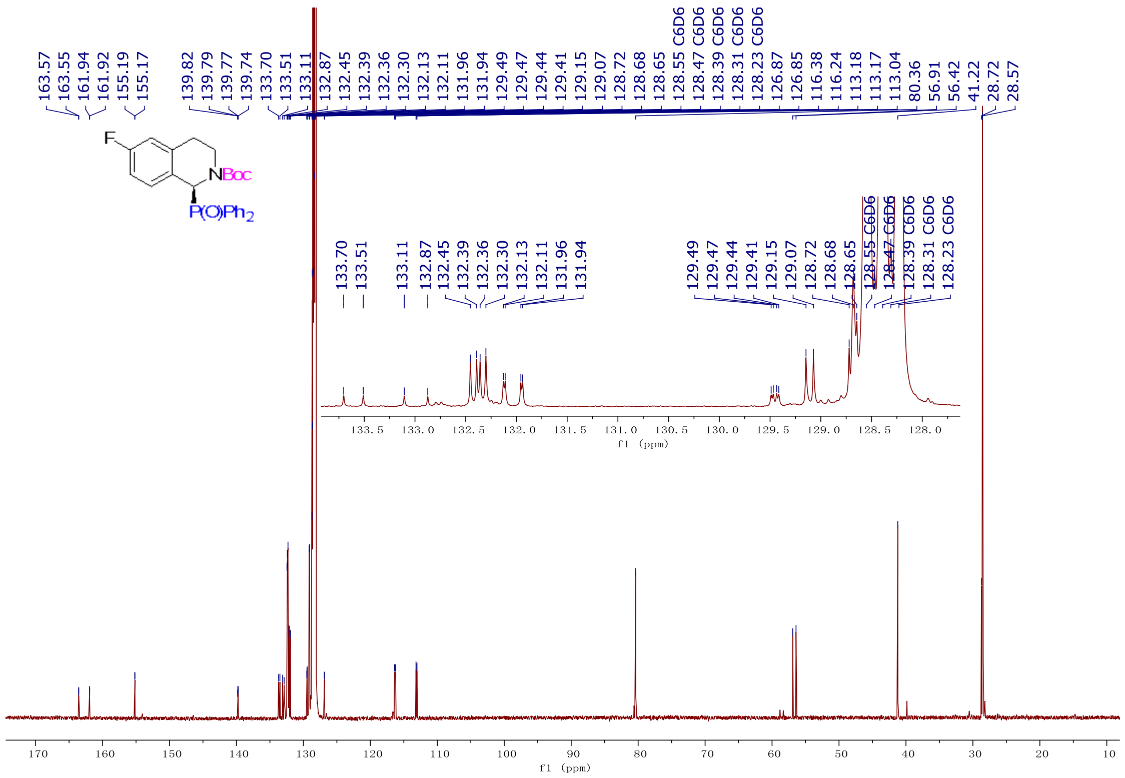

^{13}C NMR of compound **4haa**

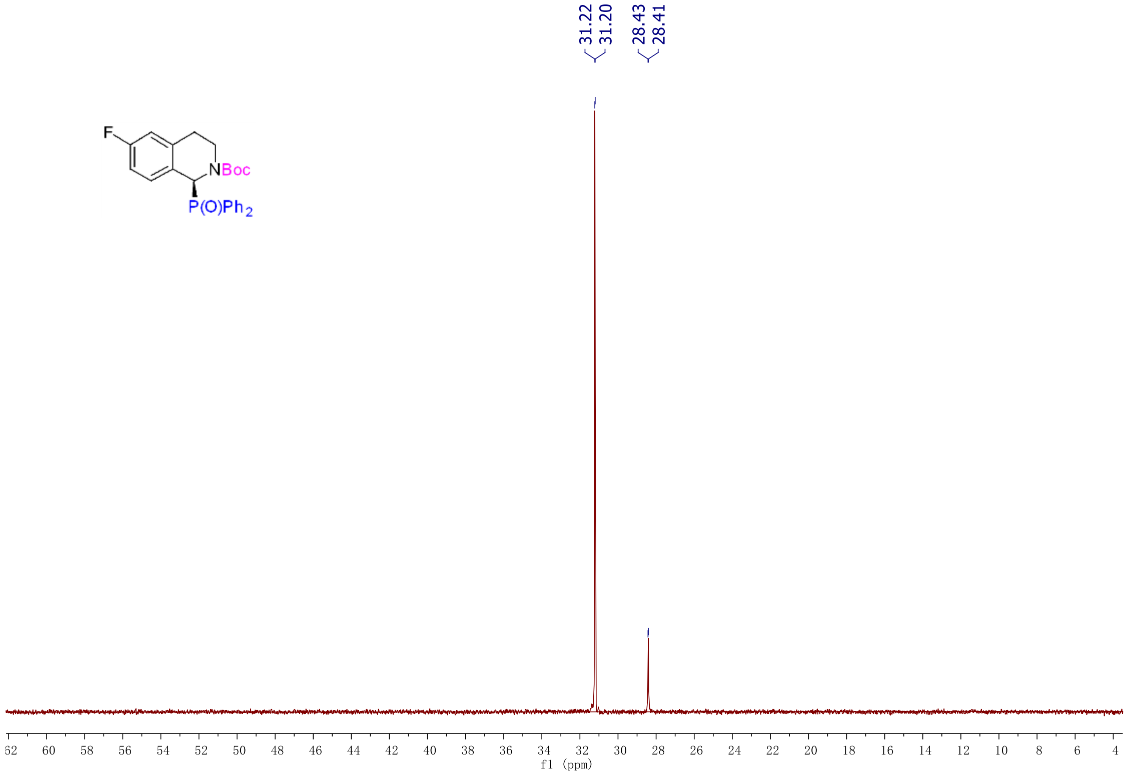

^{31}P NMR of compound **4haa**

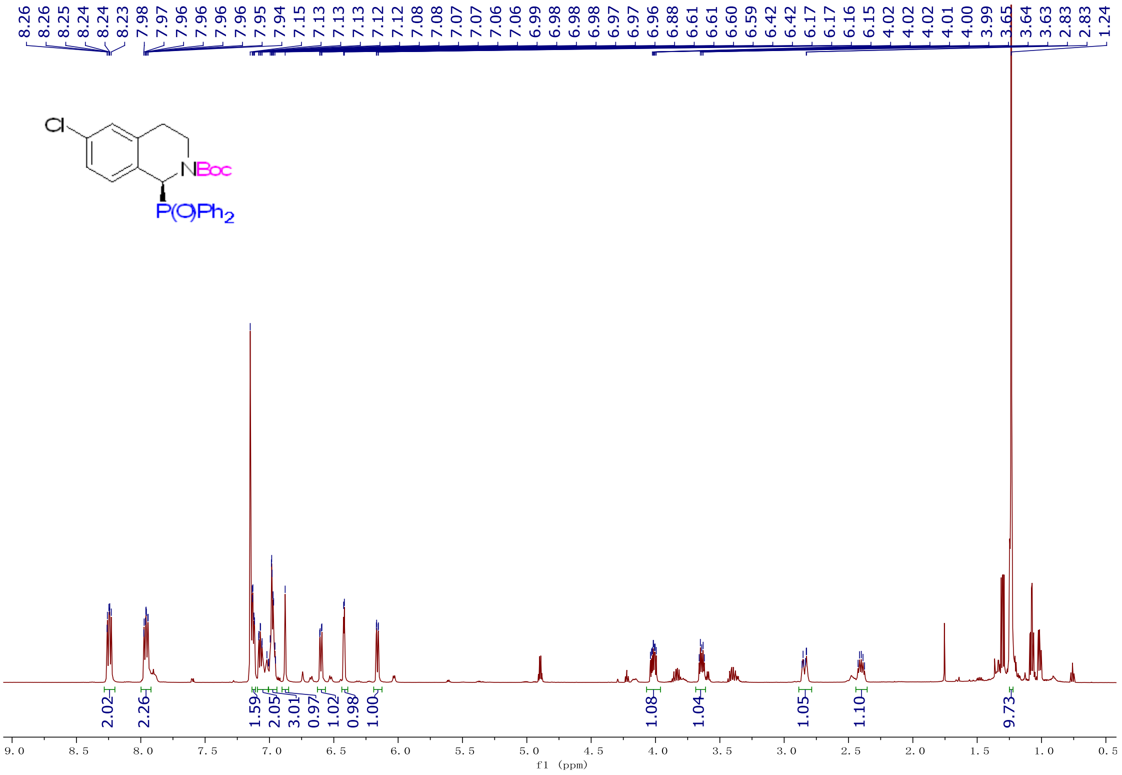

^{1}H NMR of compound **4iaa**

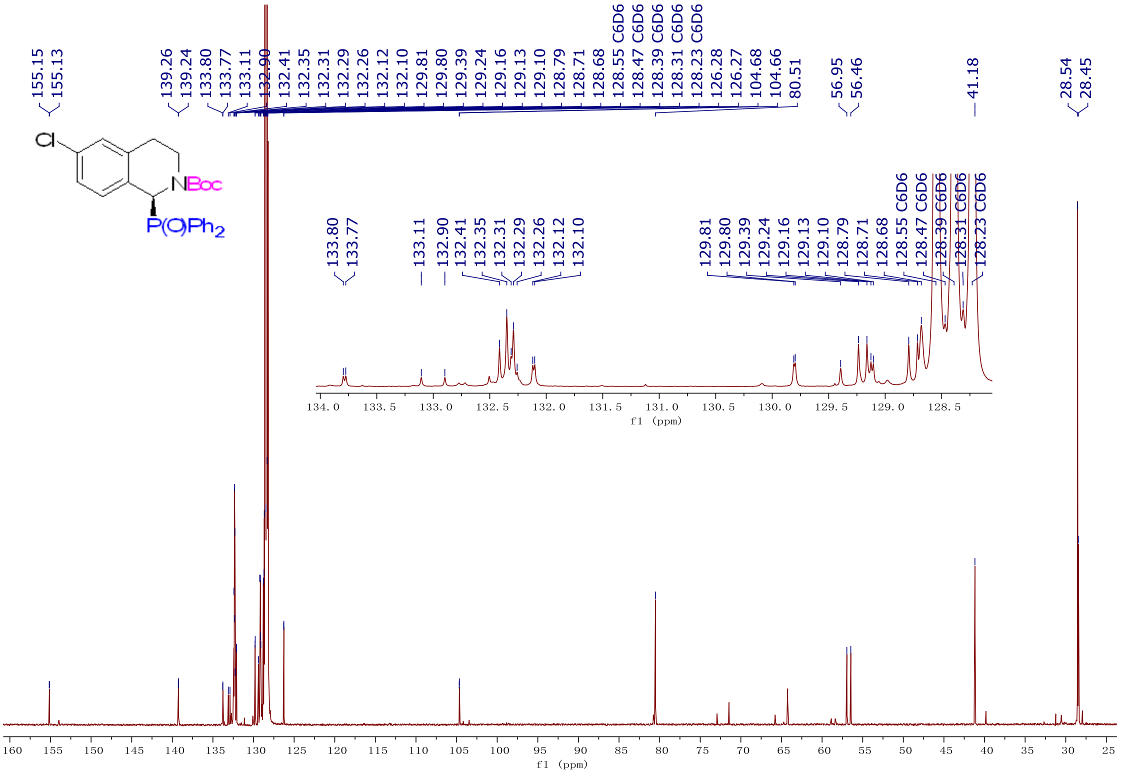

^{13}C NMR of compound **4iaa**

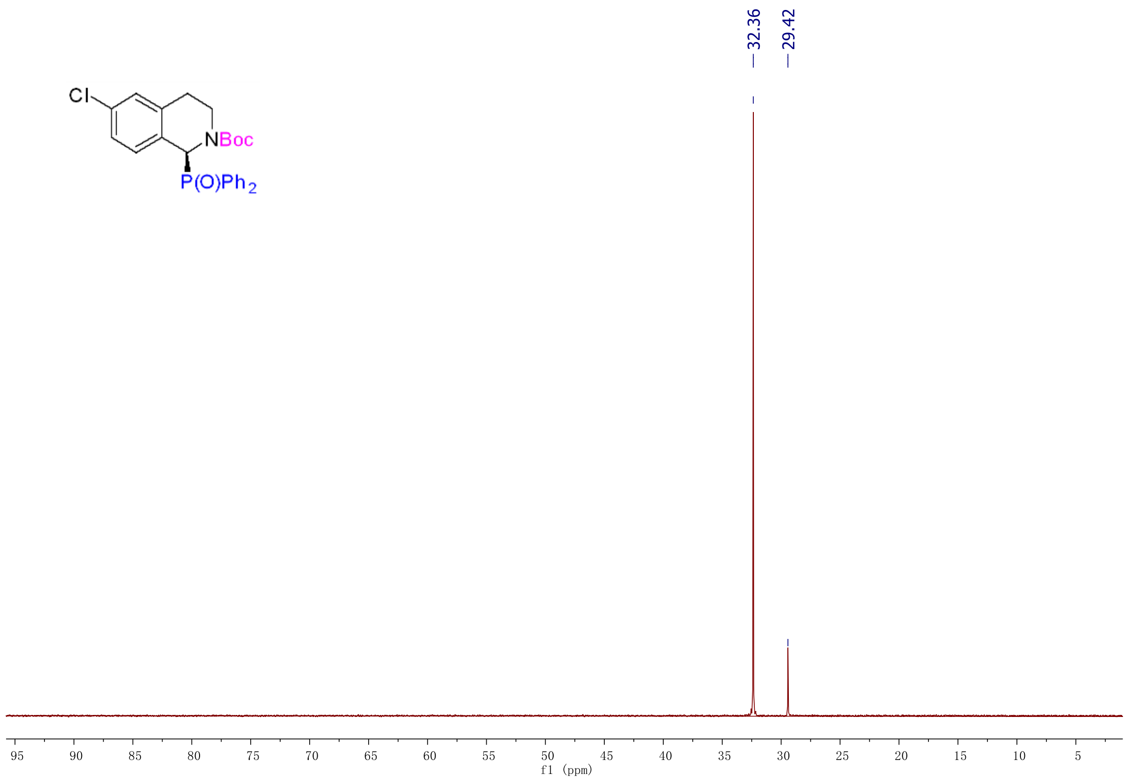

^{31}P NMR of compound **4iaa**

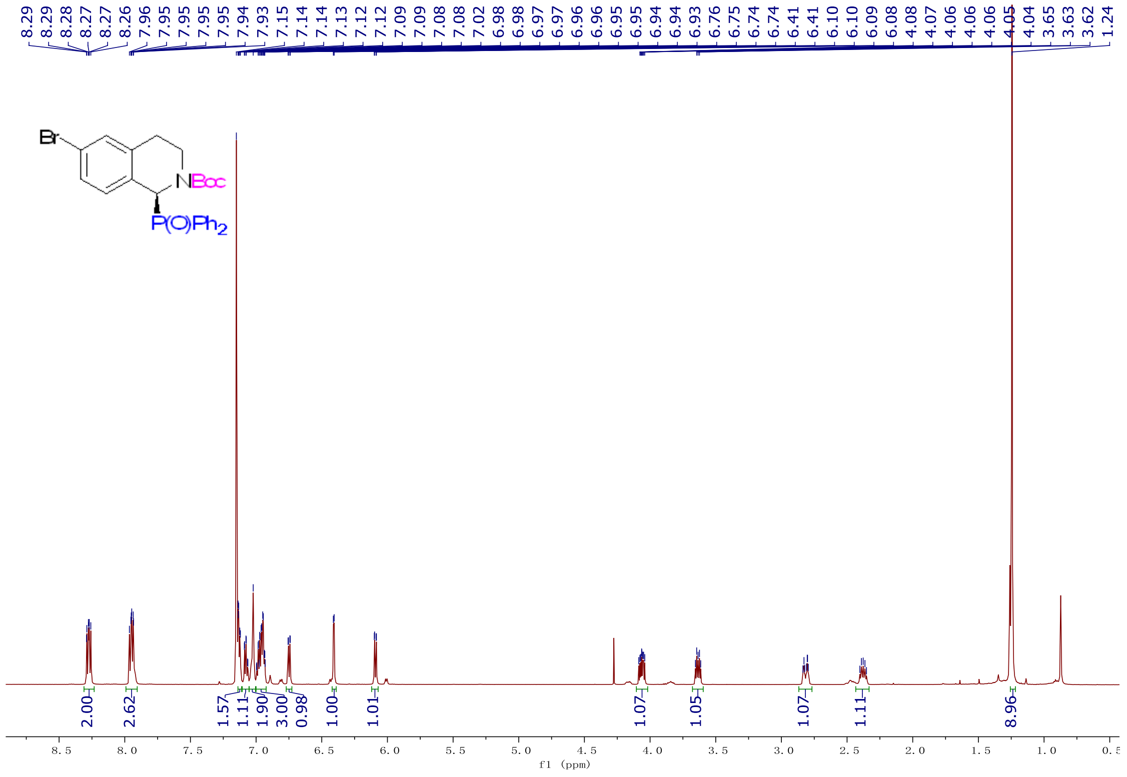

^{1}H NMR of compound **4jaa**

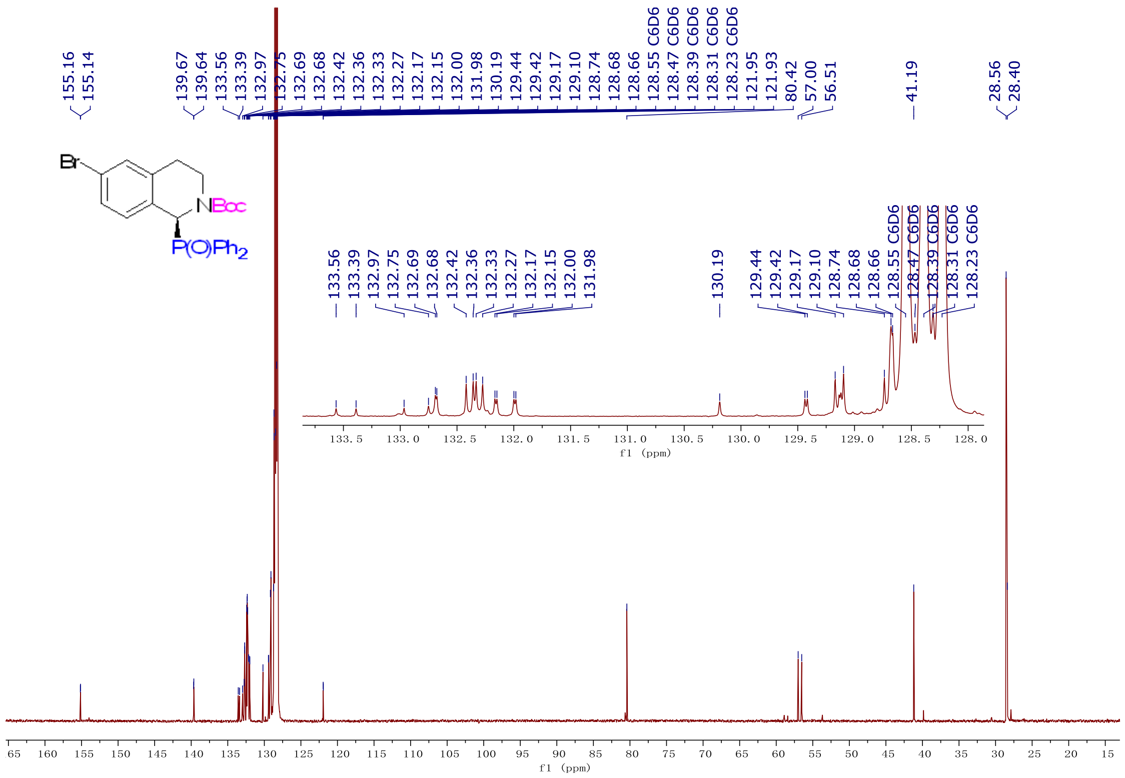

^{13}C NMR of compound **4jaa**

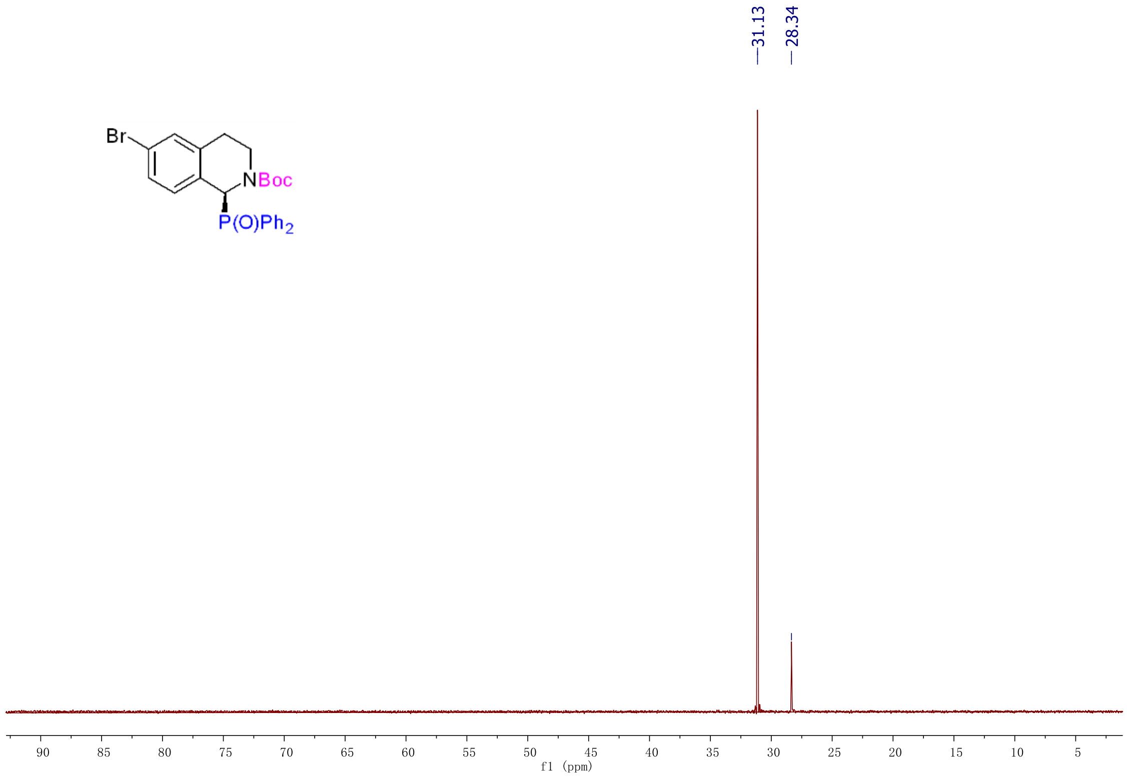

^{31}P NMR of compound **4jaa**

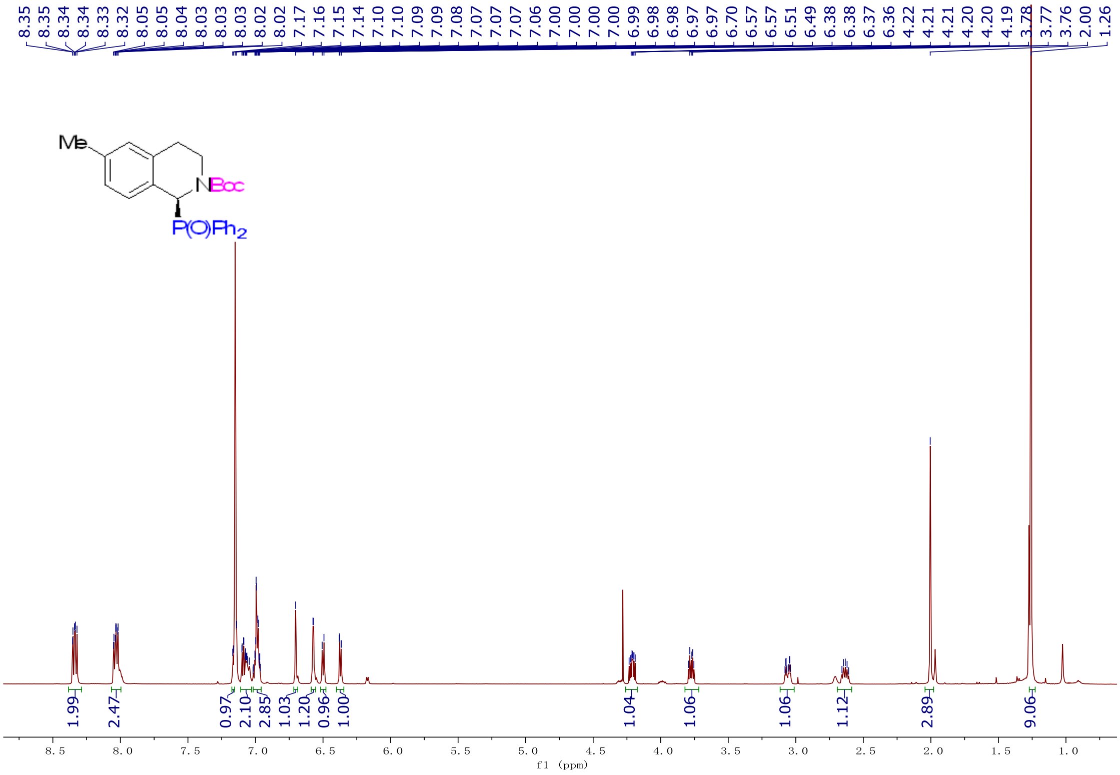

^{1}H NMR of compound **4kaa**

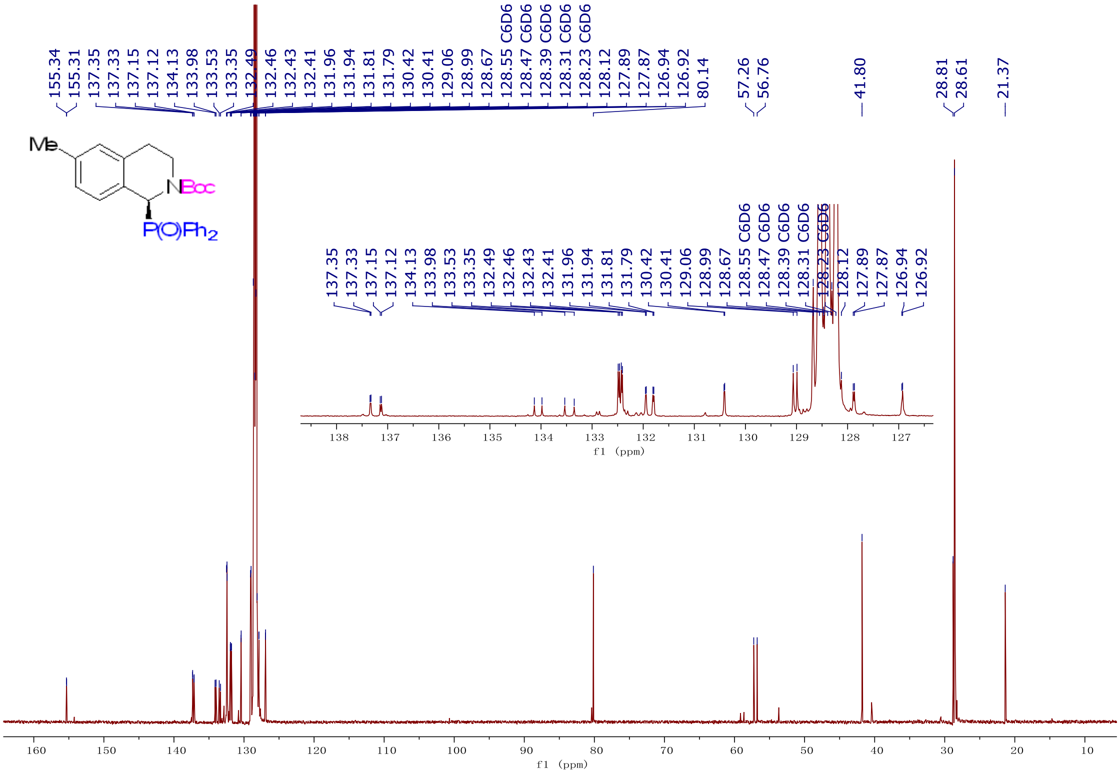

^{13}C NMR of compound **4kaa**

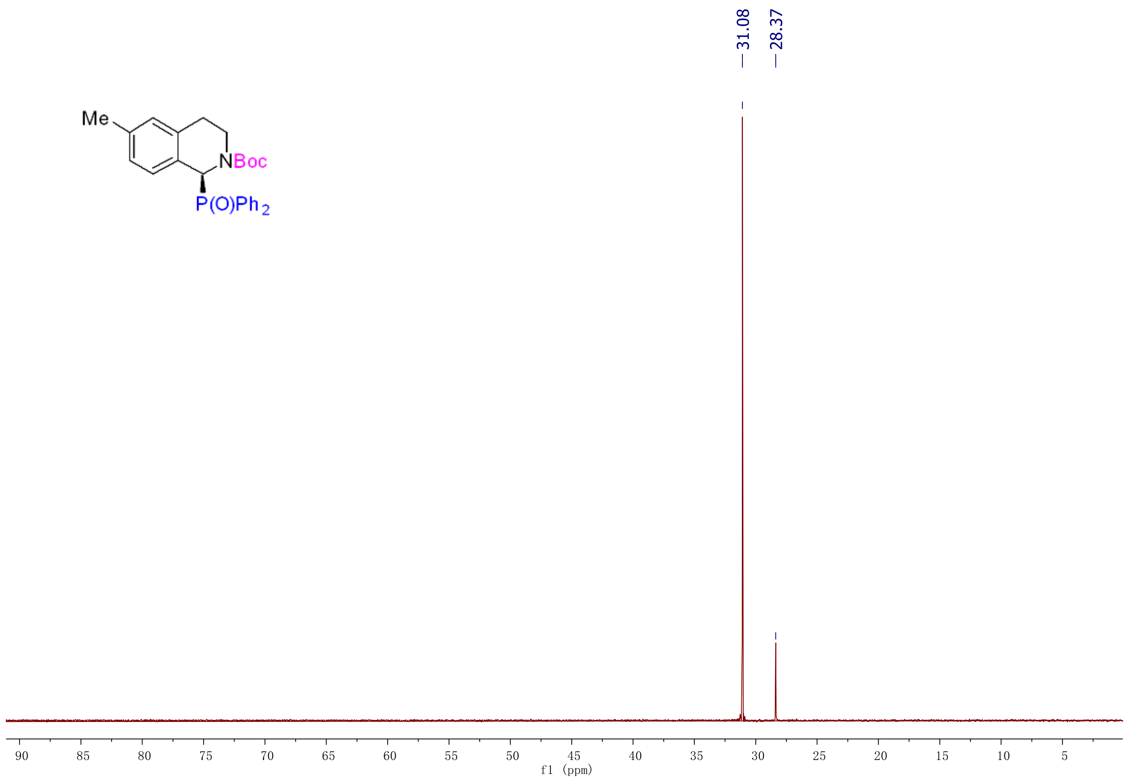

^{31}P NMR of compound **4kaa**

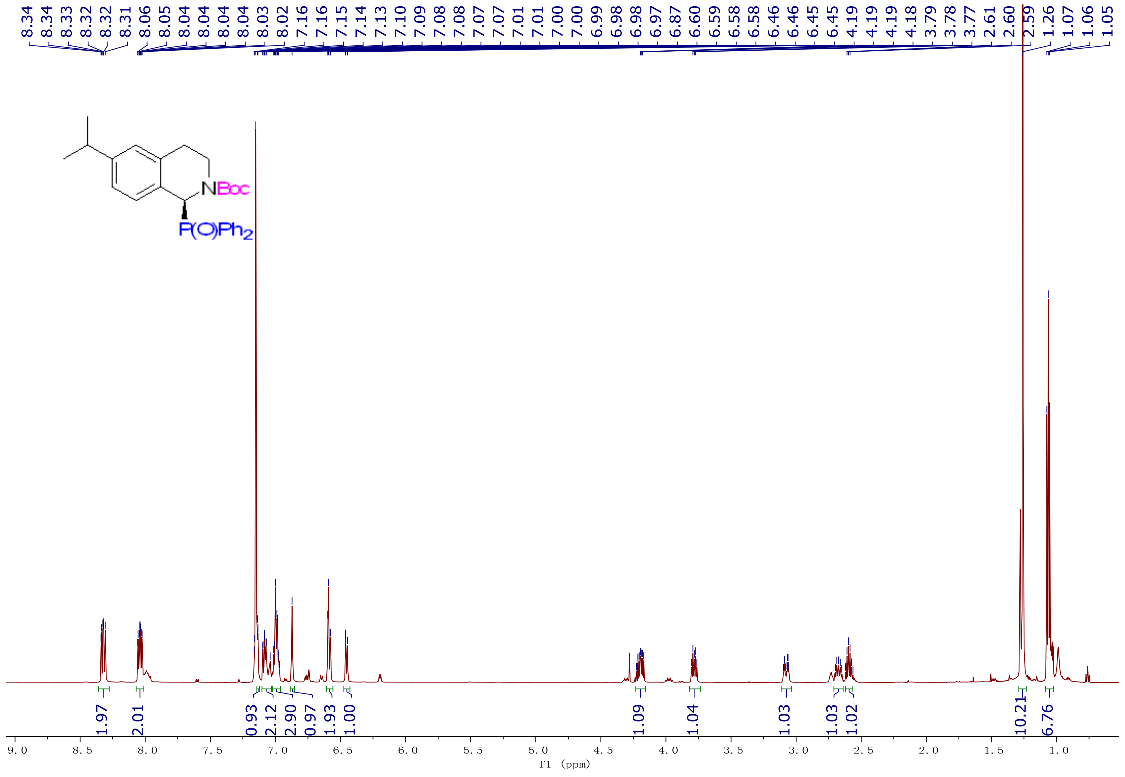

^{1}H NMR of compound **4laa**

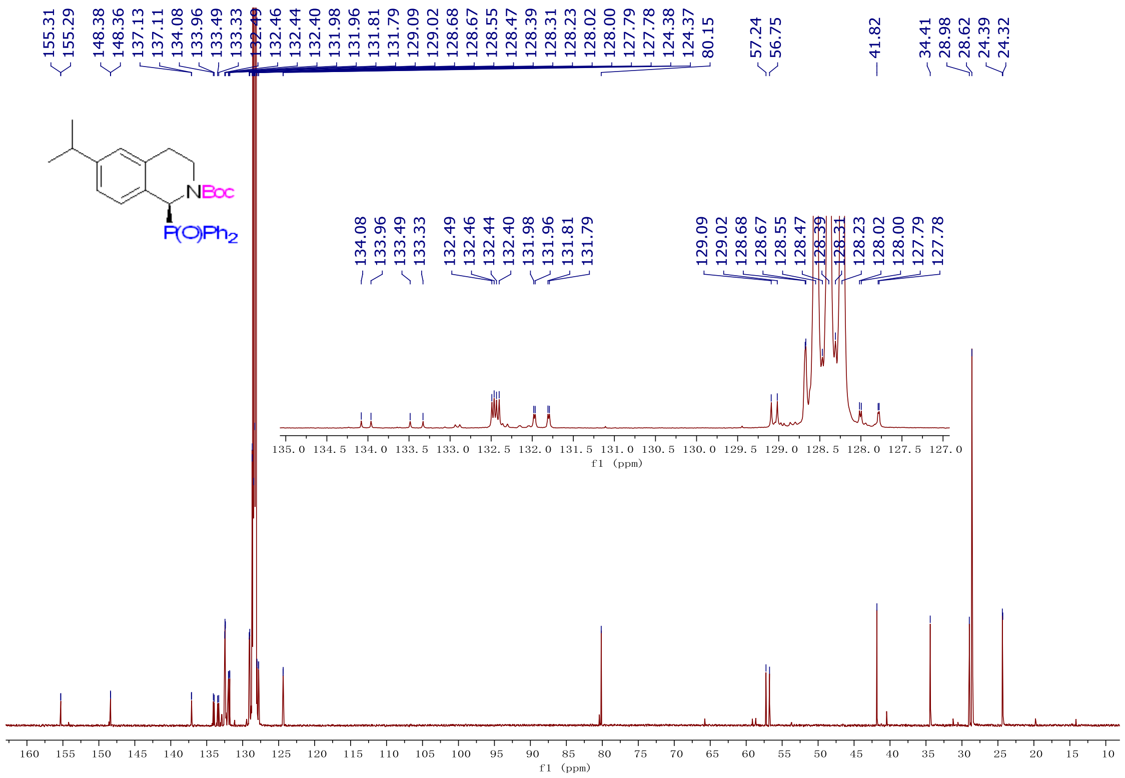

^{13}C NMR of compound **4laa**

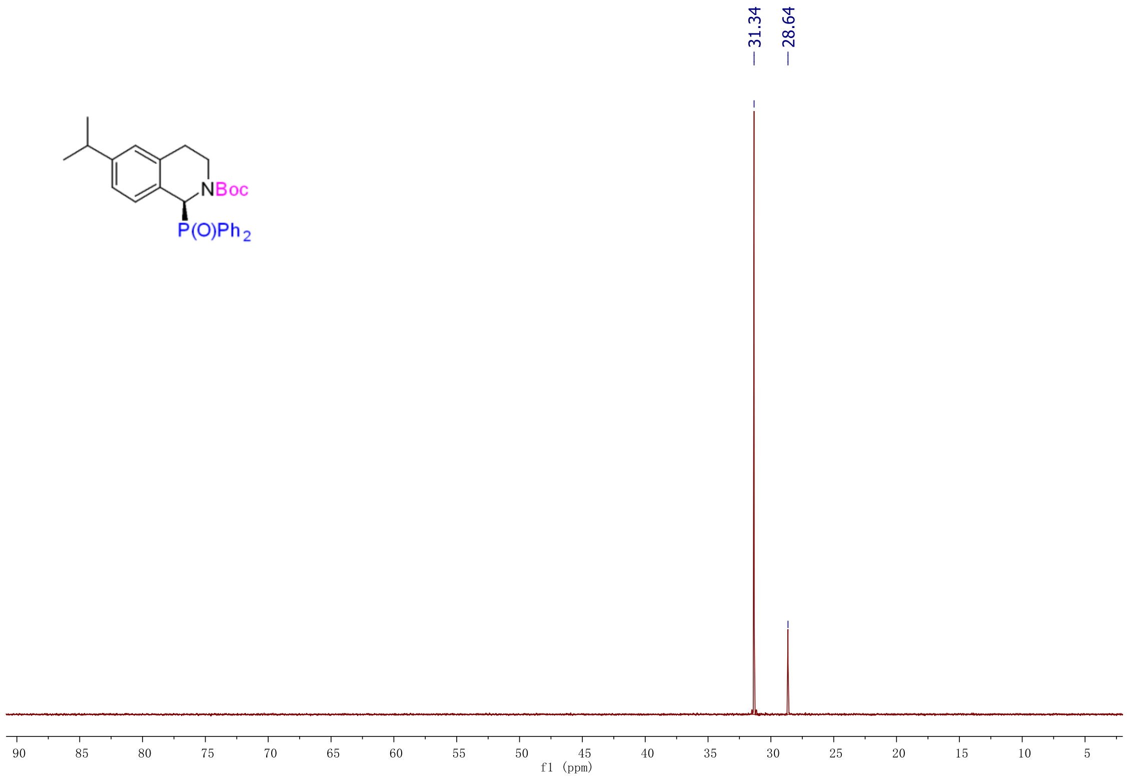

^{31}P NMR of compound **4laa**

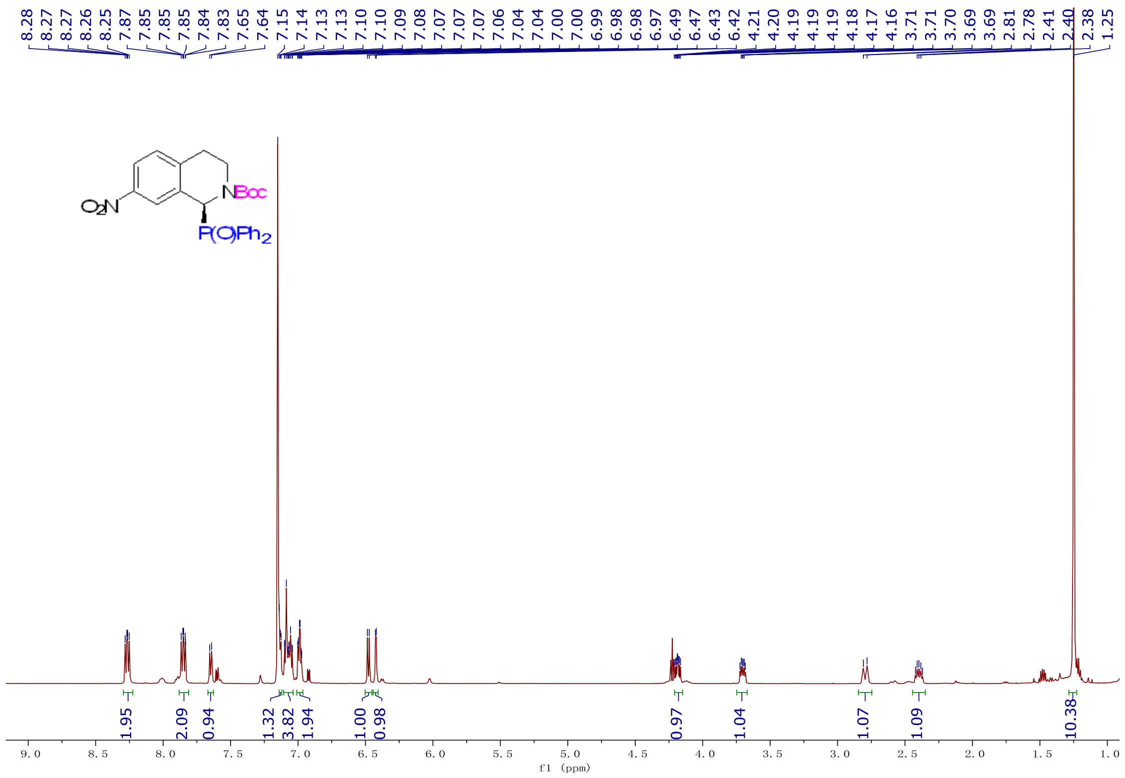

^{1}H NMR of compound **4maa**

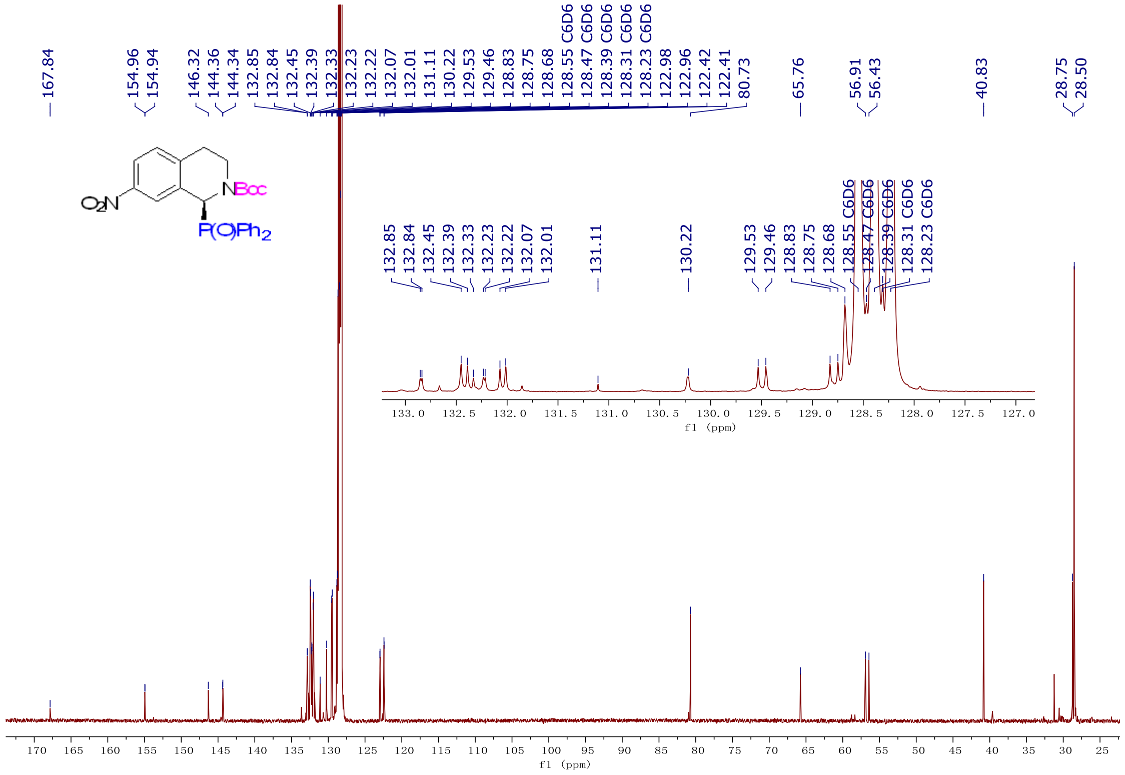

^{13}C NMR of compound **4maa**

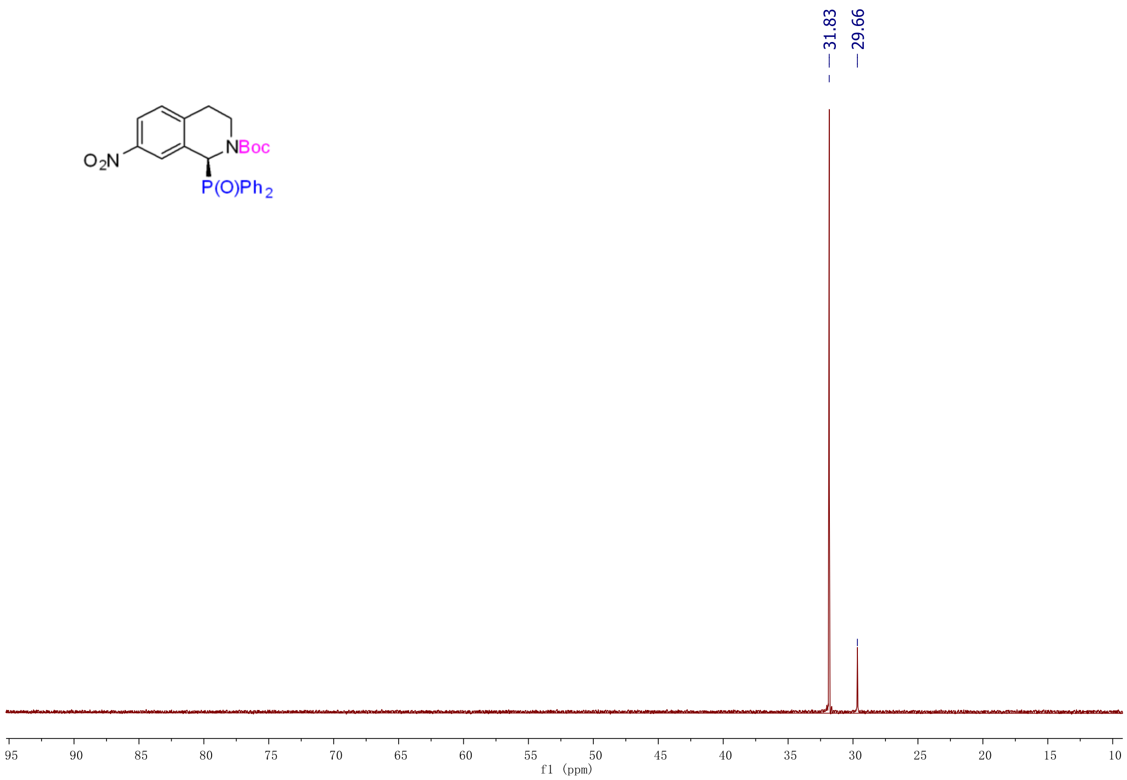

^{31}P NMR of compound **4maa**

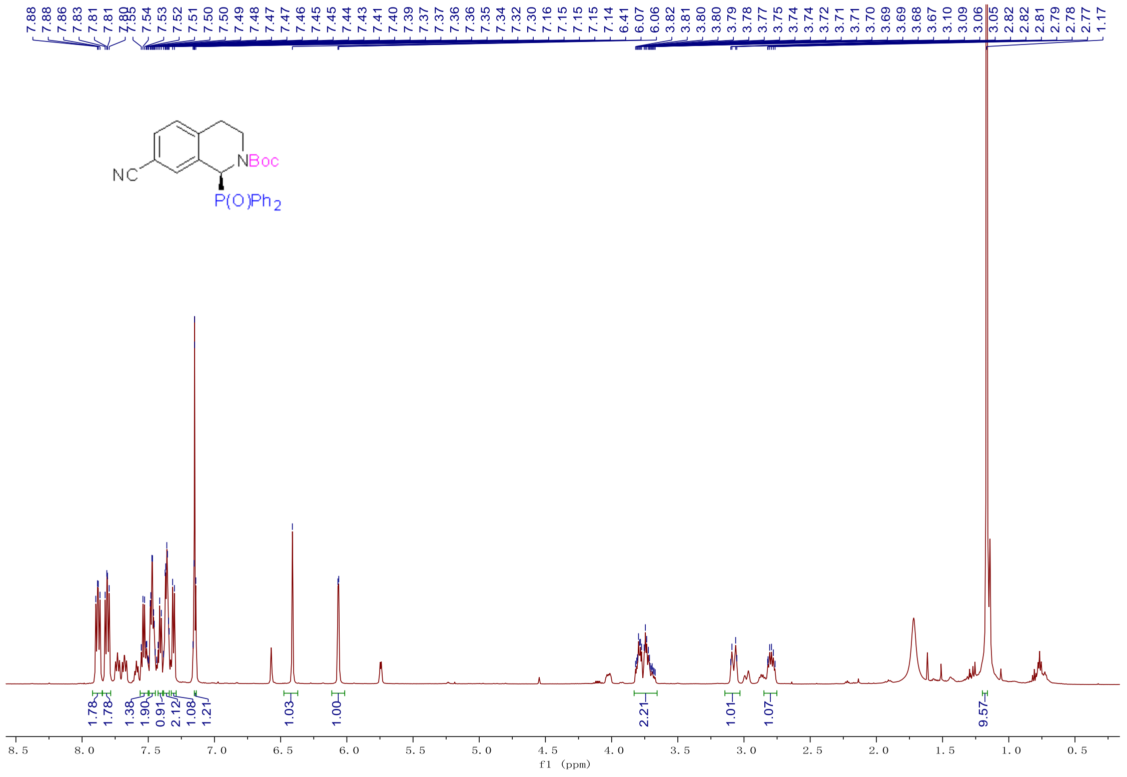

^{1}H NMR of compound **4naa**

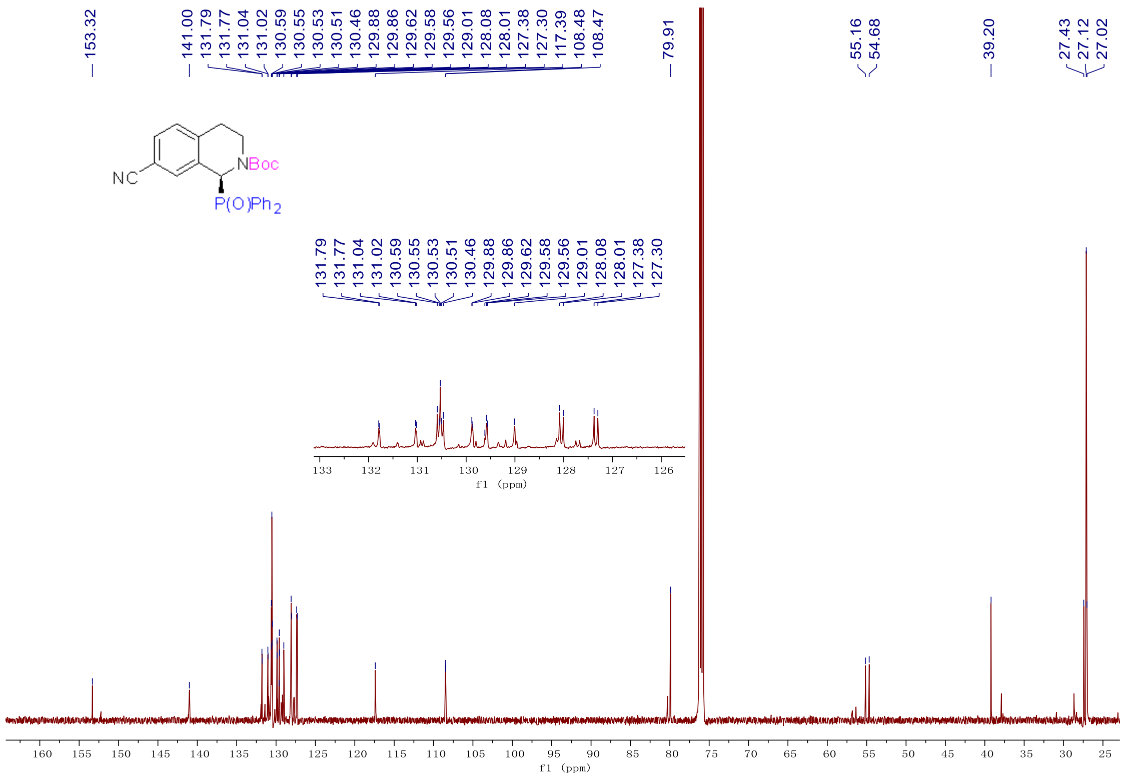

^{13}C NMR of compound **4naa**

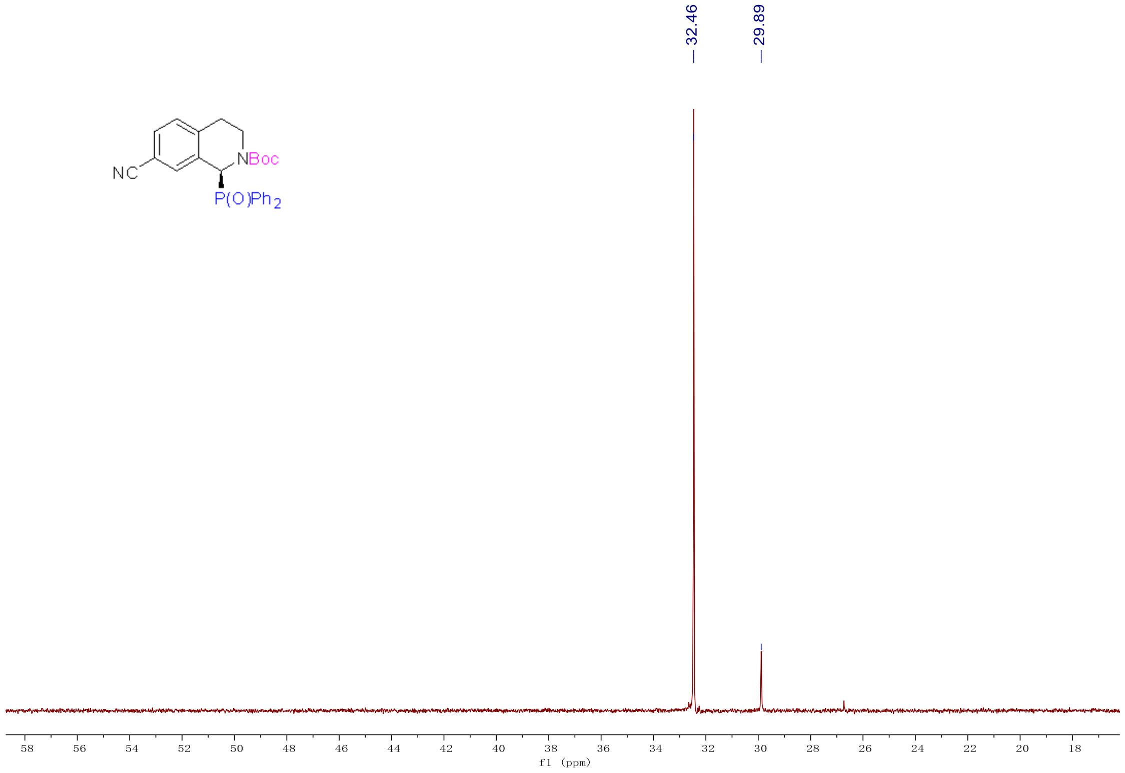

^{31}P NMR of compound **4naa**

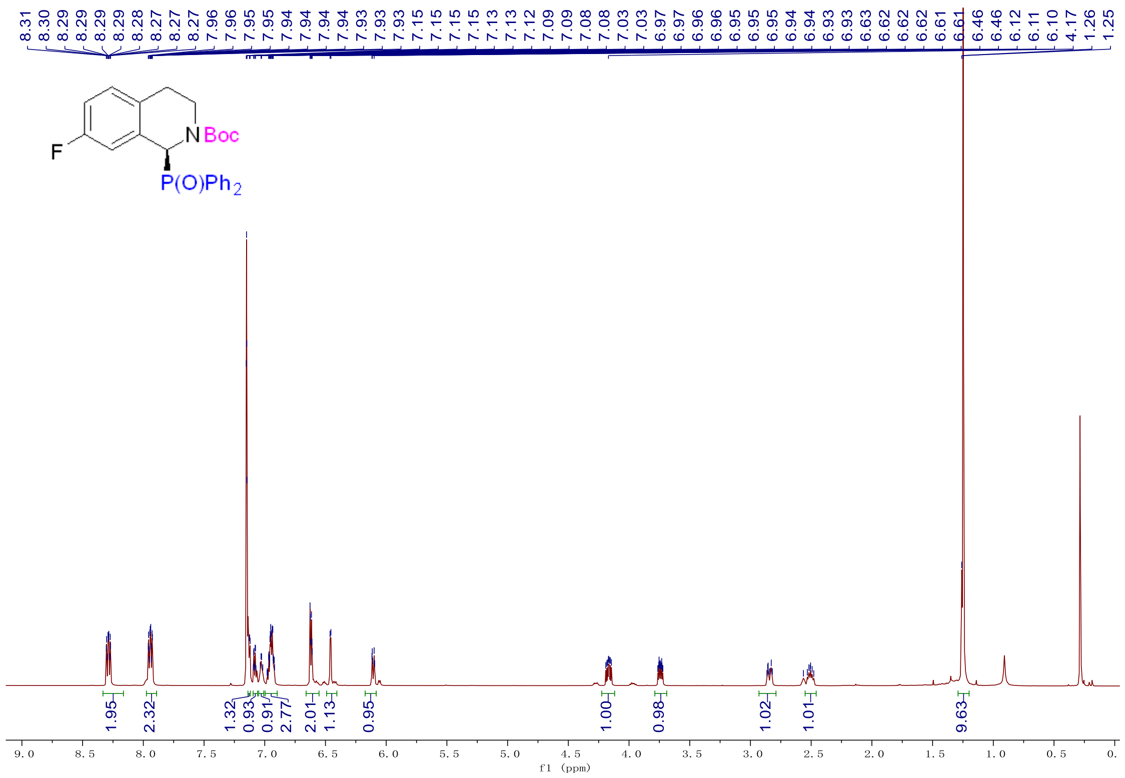

^{1}H NMR of compound **4oaa**

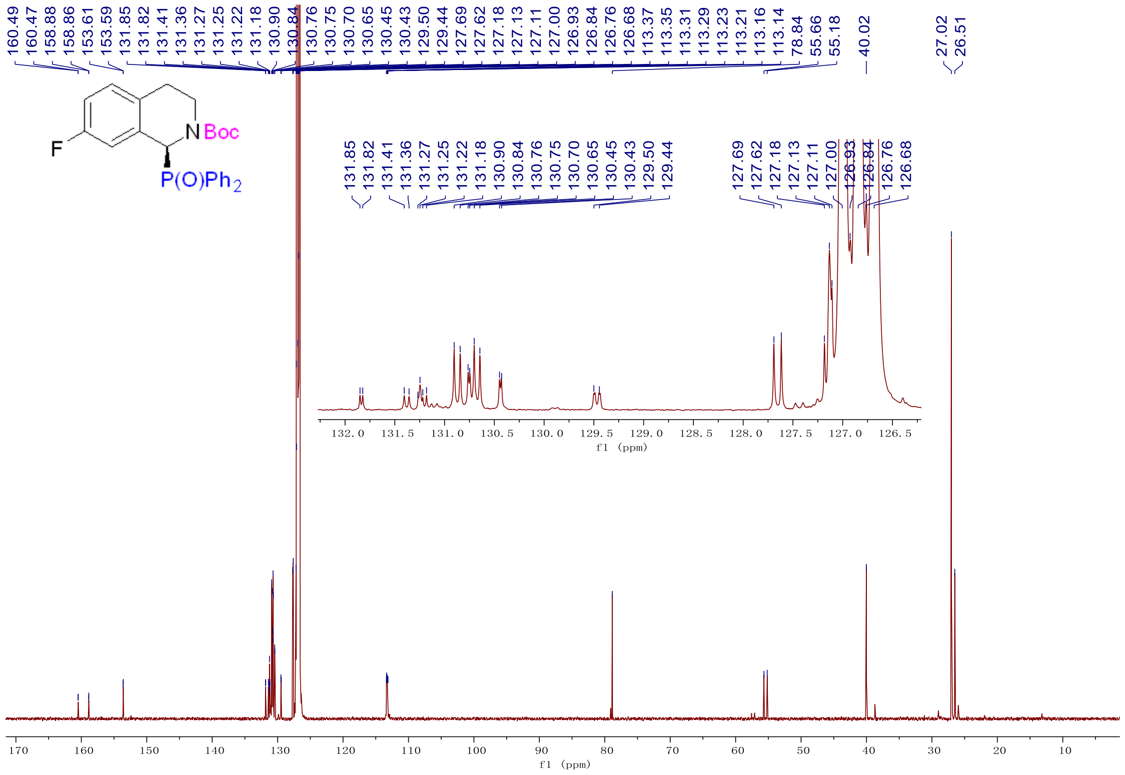

^{13}C NMR of compound **4oaa**

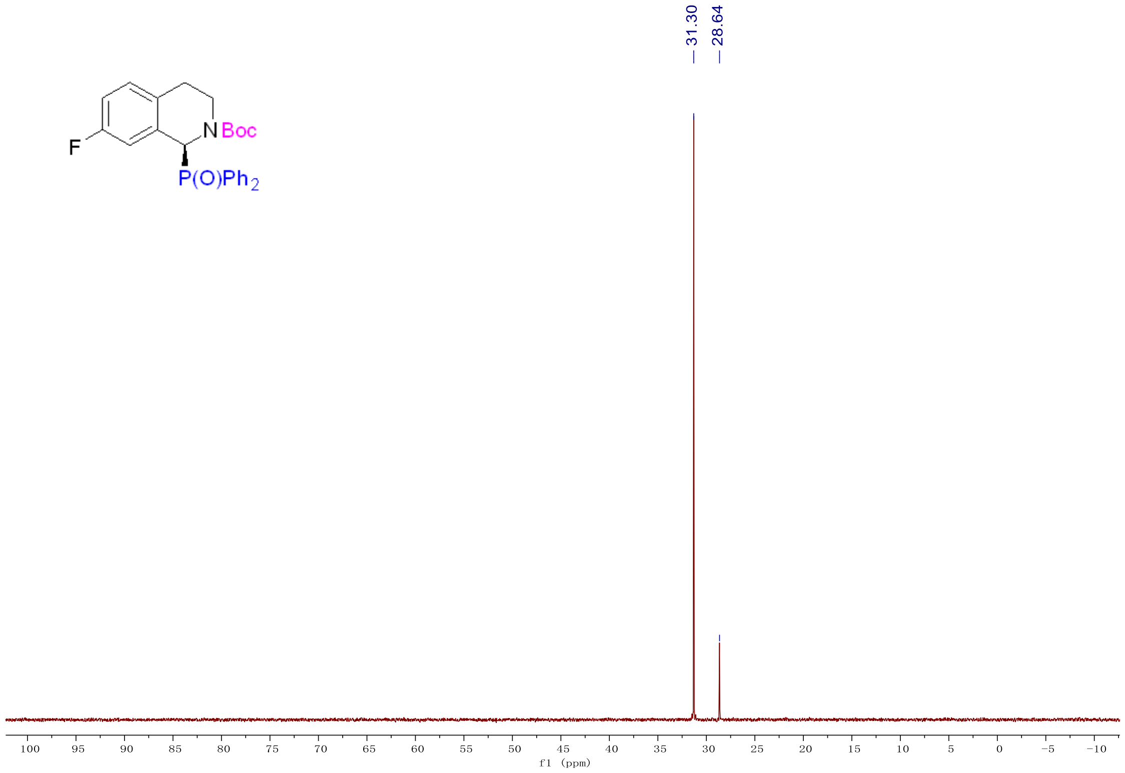

^{31}P NMR of compound **4oaa**

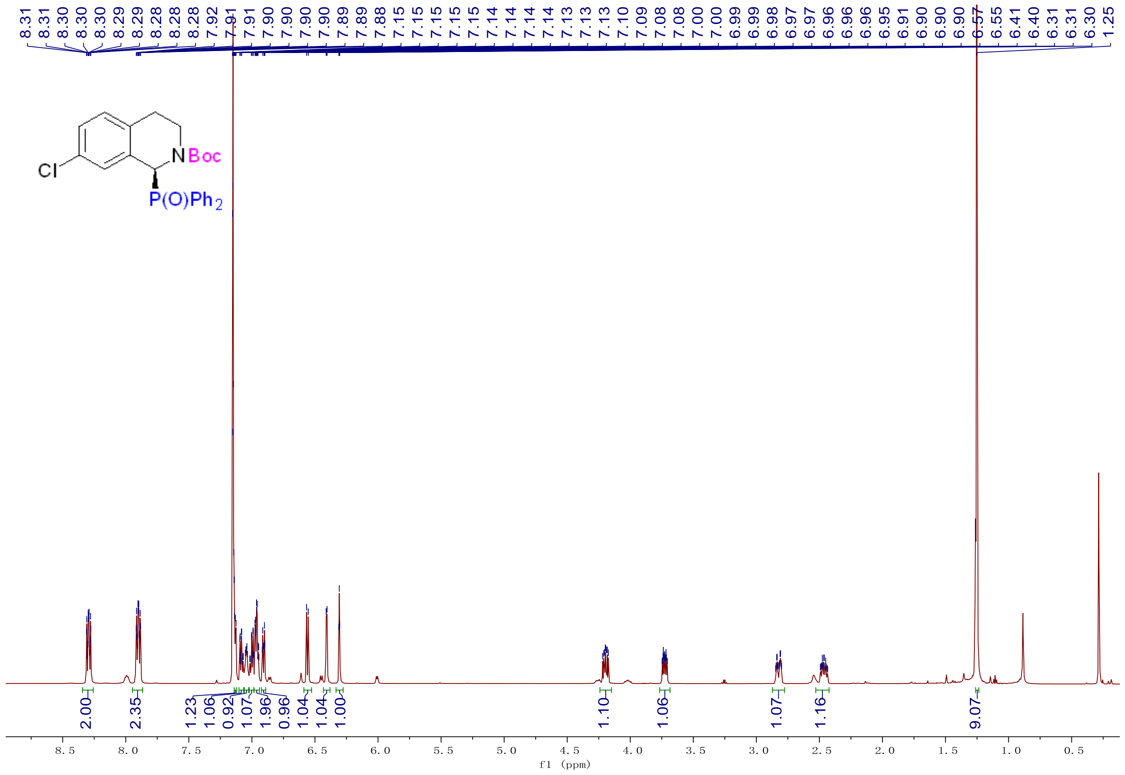

^{1}H NMR of compound **4paa**

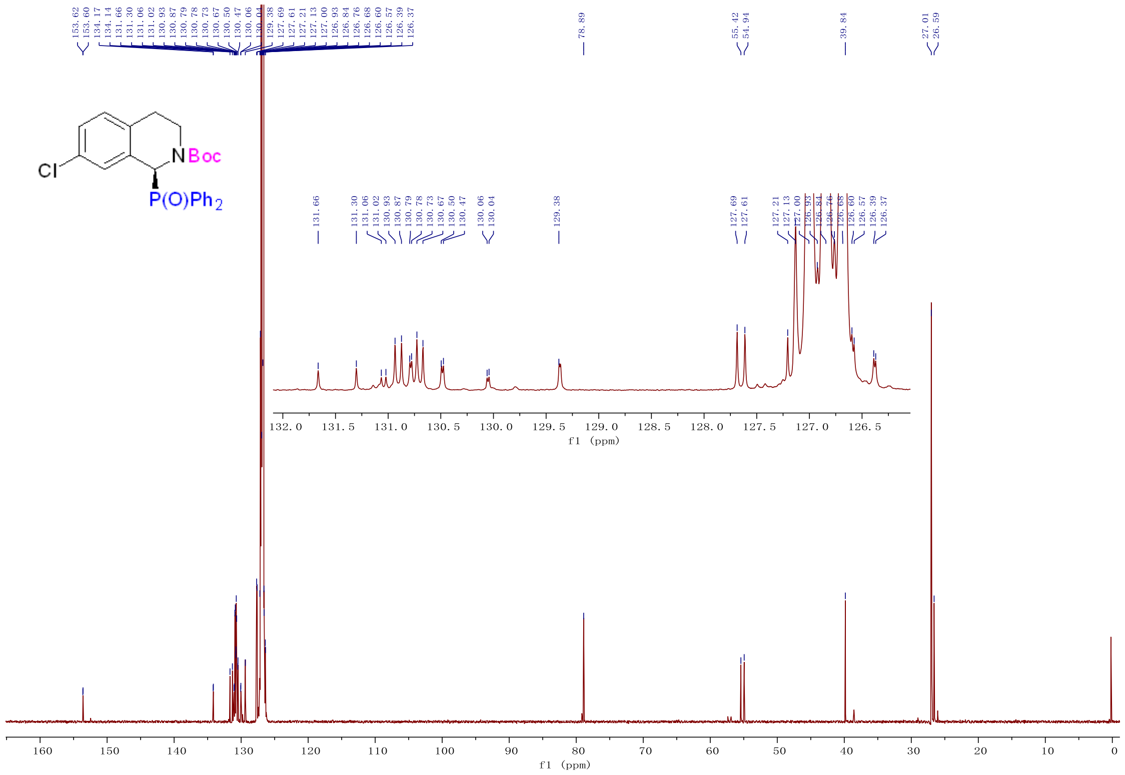

^{13}C NMR of compound **4paa**

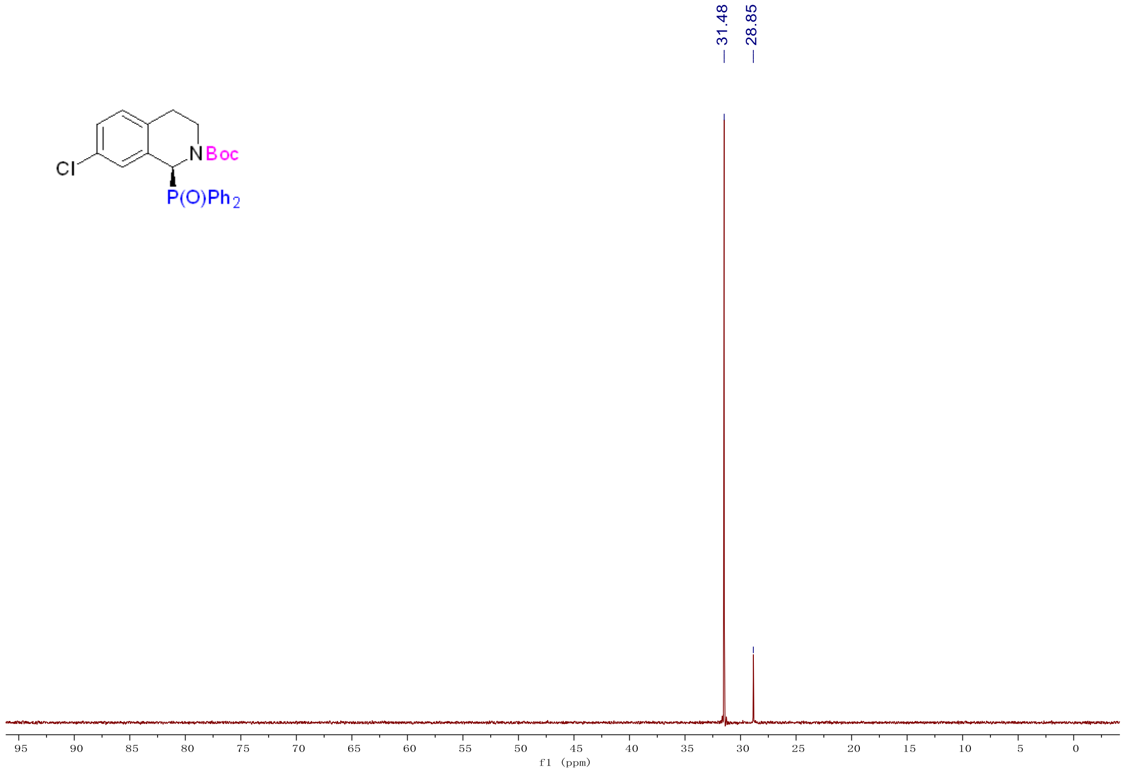

^{31}P NMR of compound **4paa**

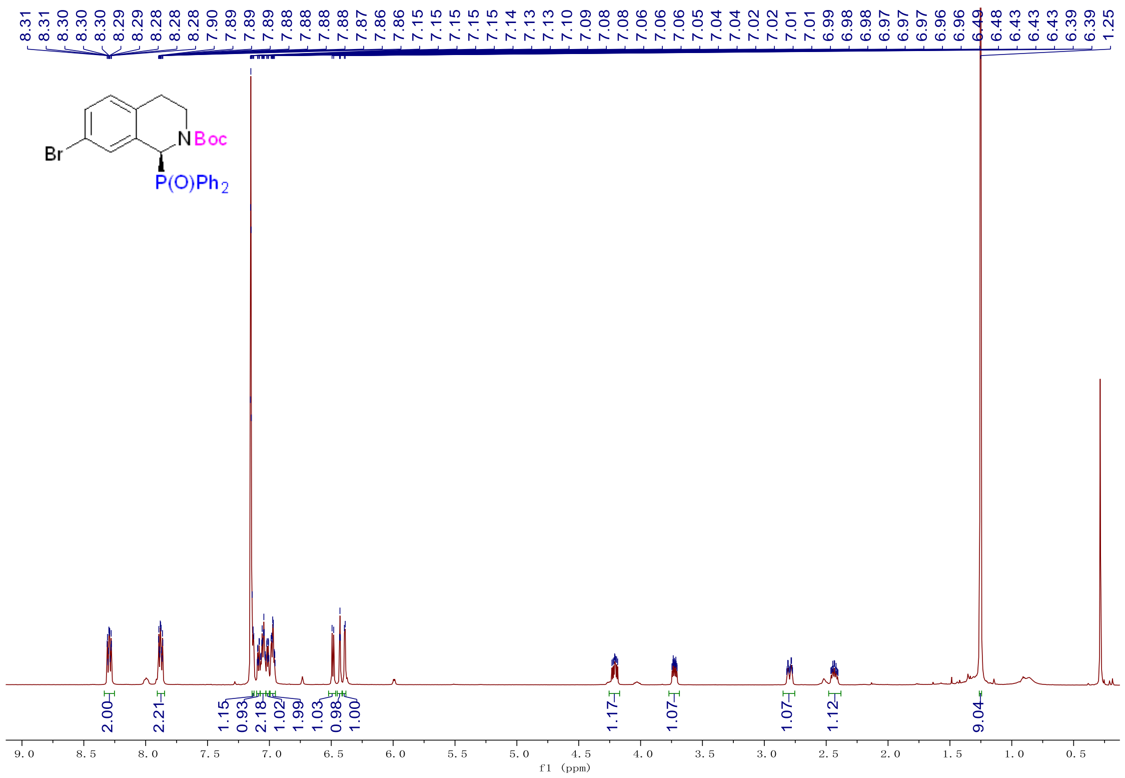

^{1}H NMR of compound **4qaa**

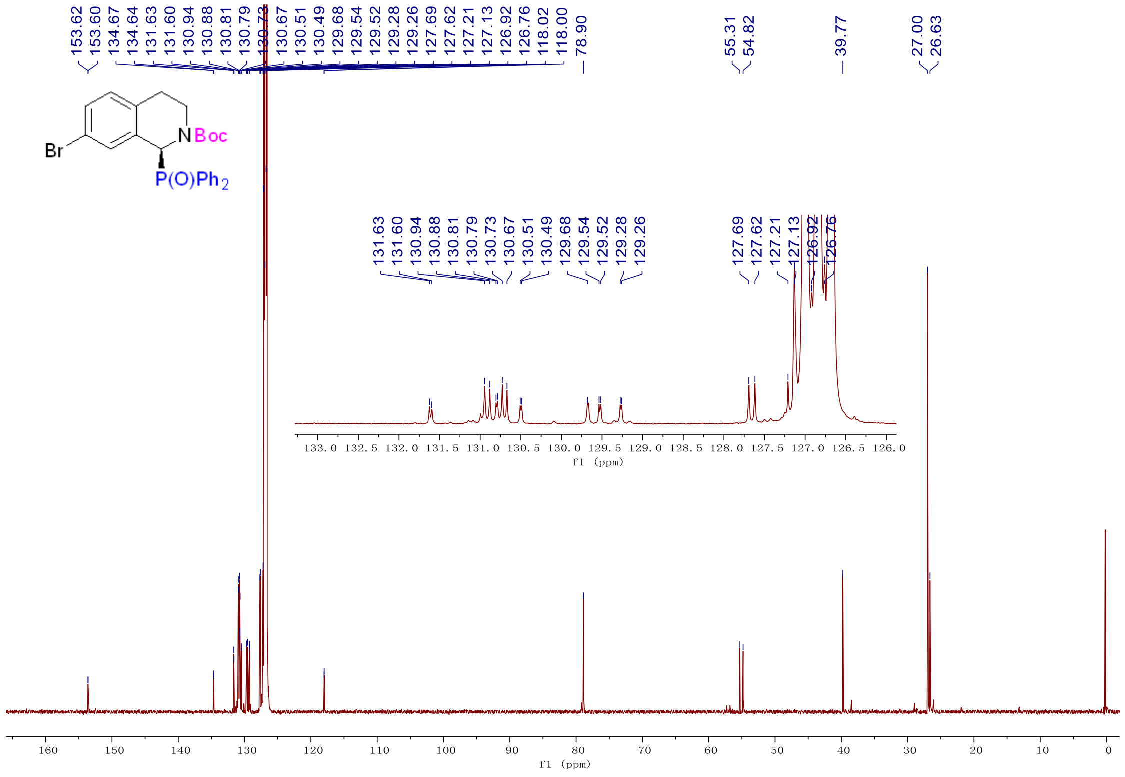

^{13}C NMR of compound **4qaa**

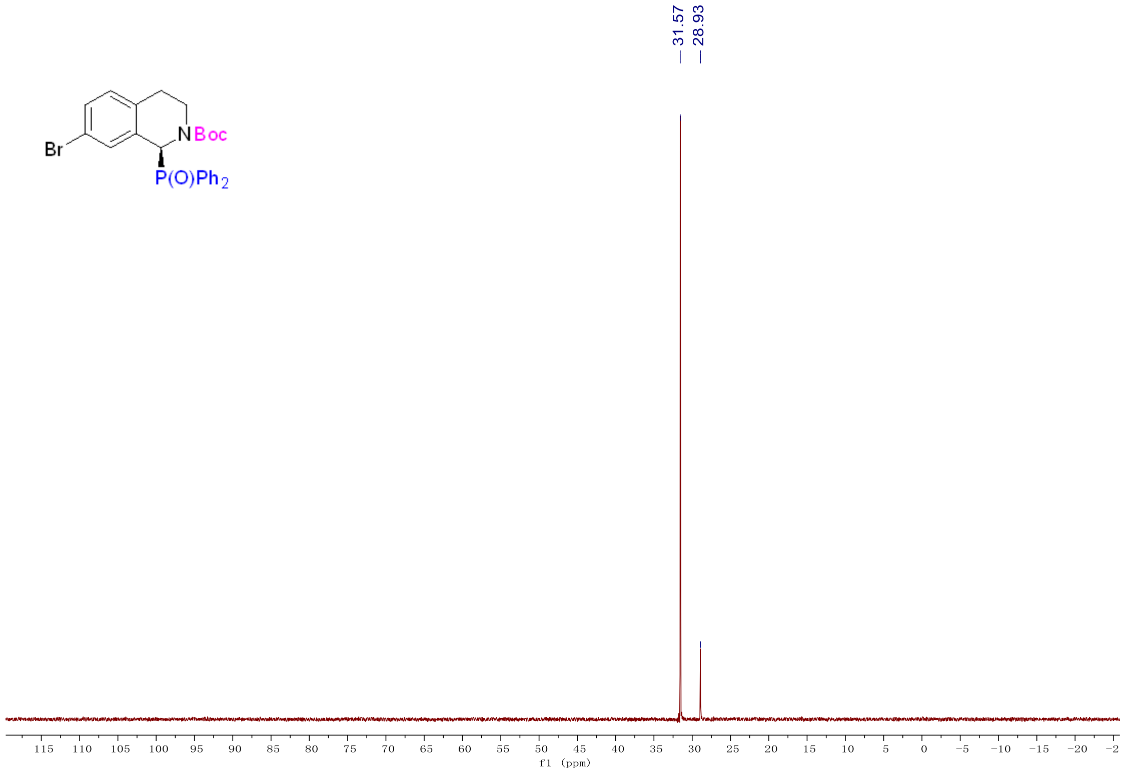

^{31}P NMR of compound **4qaa**

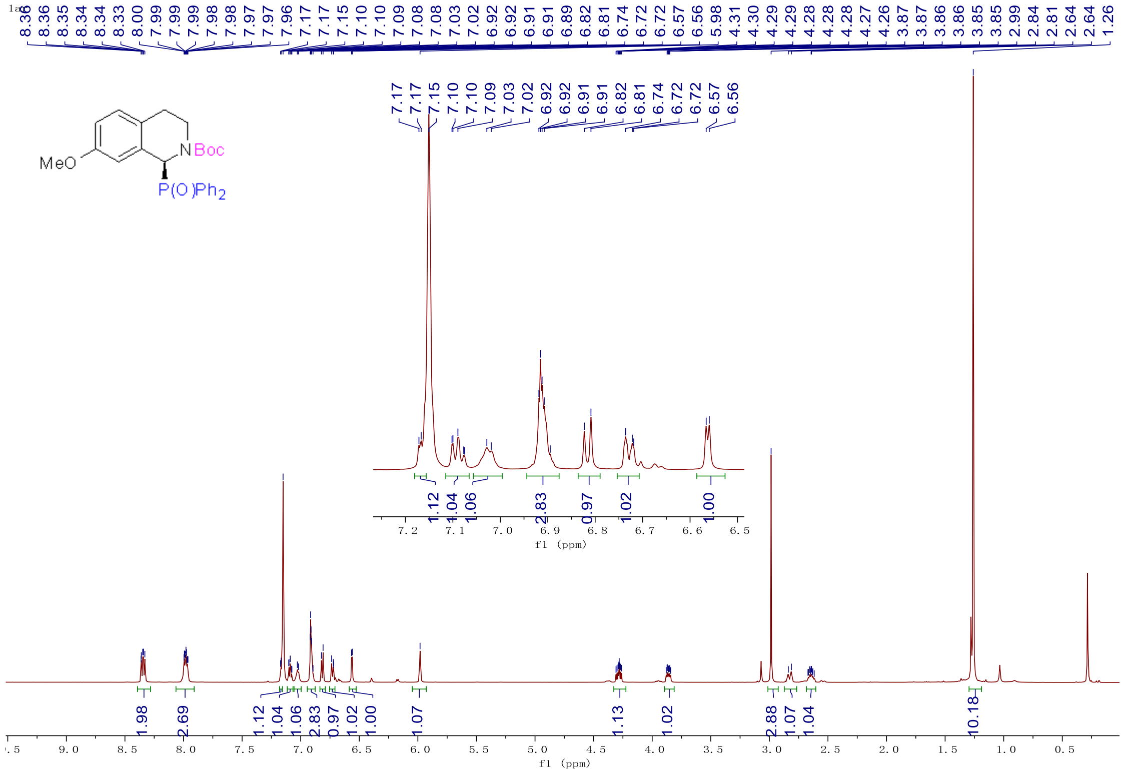

^{1}H NMR of compound **4raa**

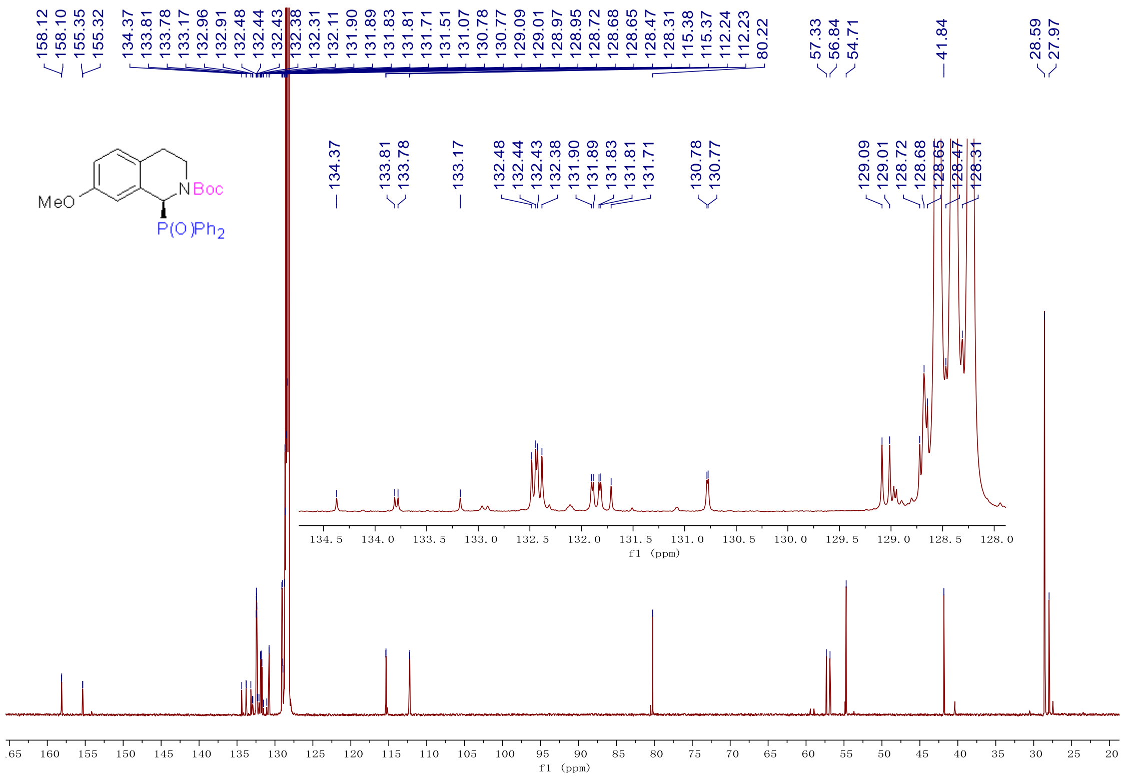

^{13}C NMR of compound **4raa**

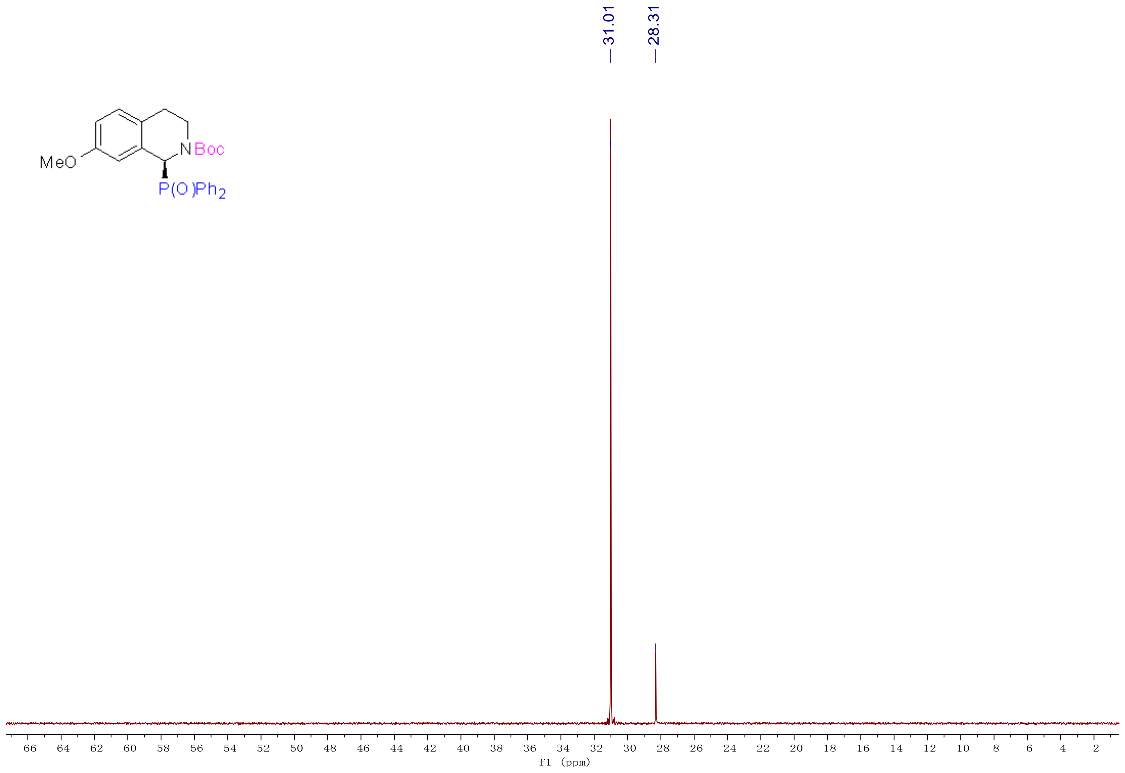

^{31}P NMR of compound **4raa**

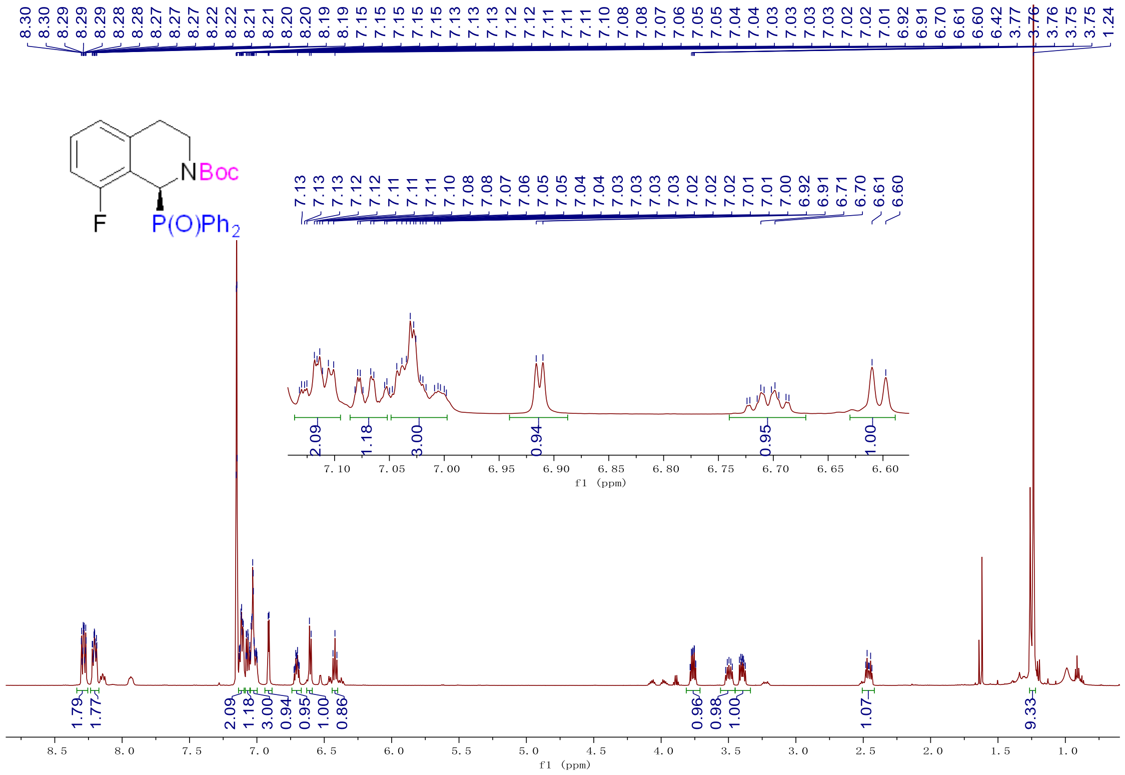

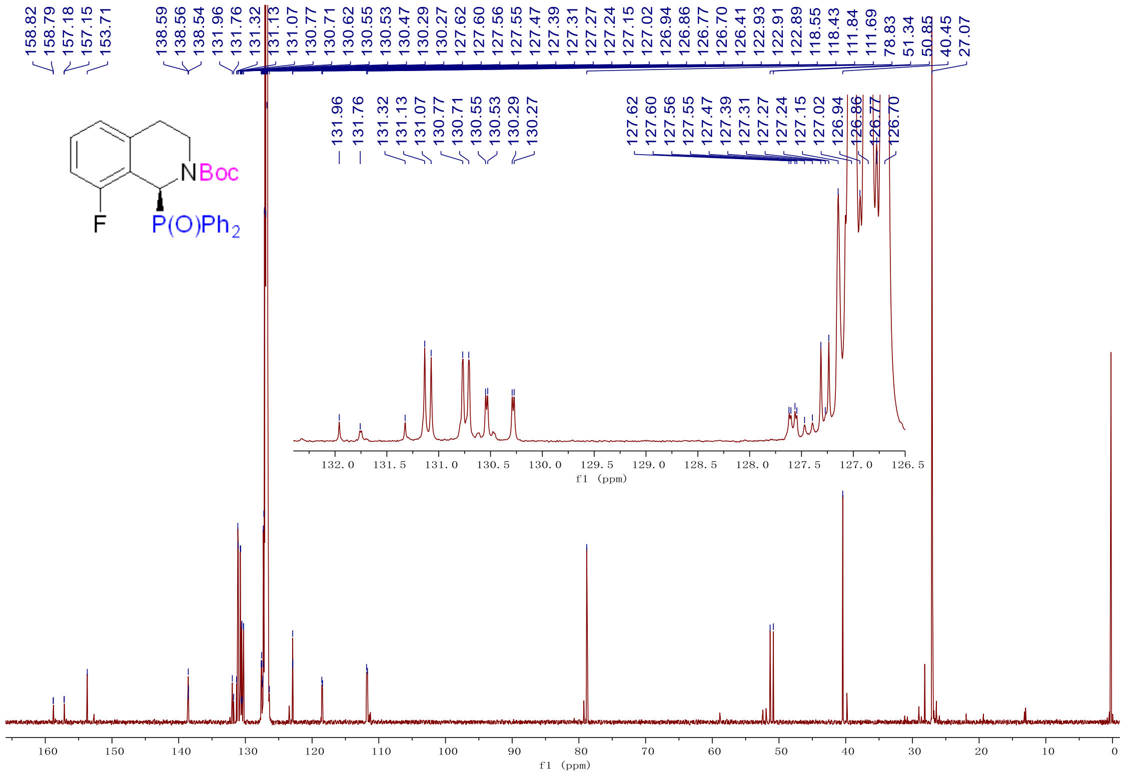

^{13}C NMR of compound **4saa**

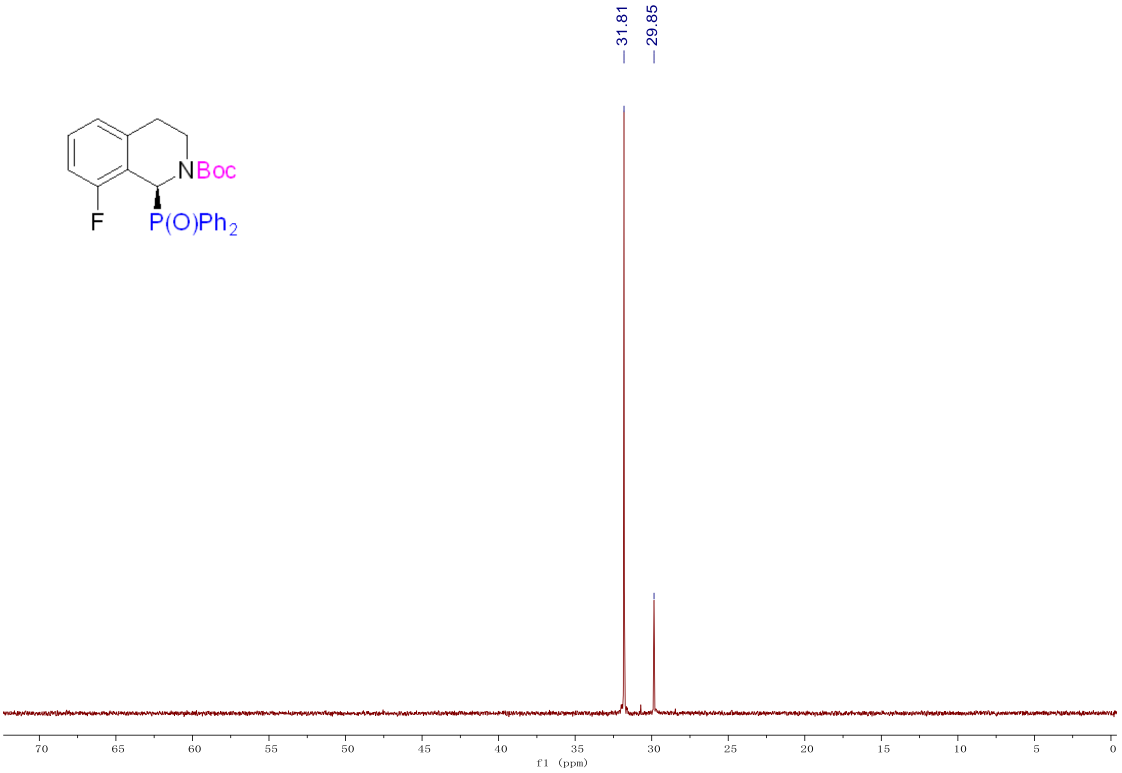

^{31}P NMR of compound **4saa**

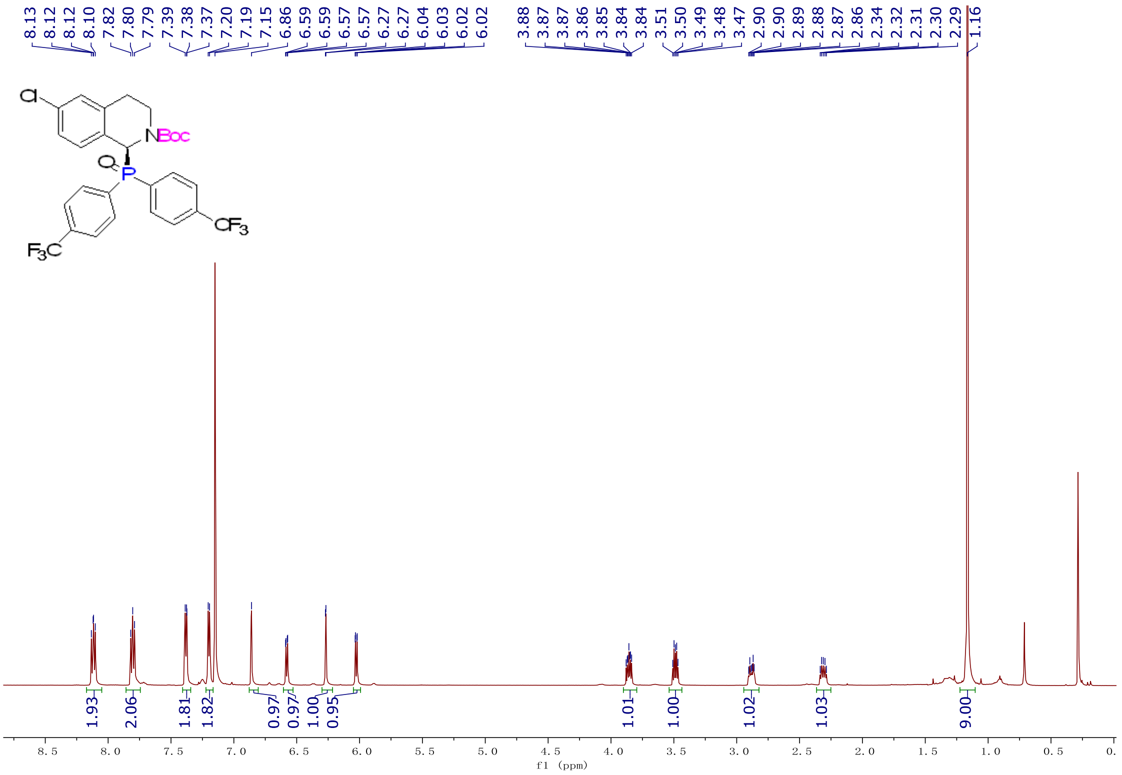

^{1}H NMR of compound **4iba**

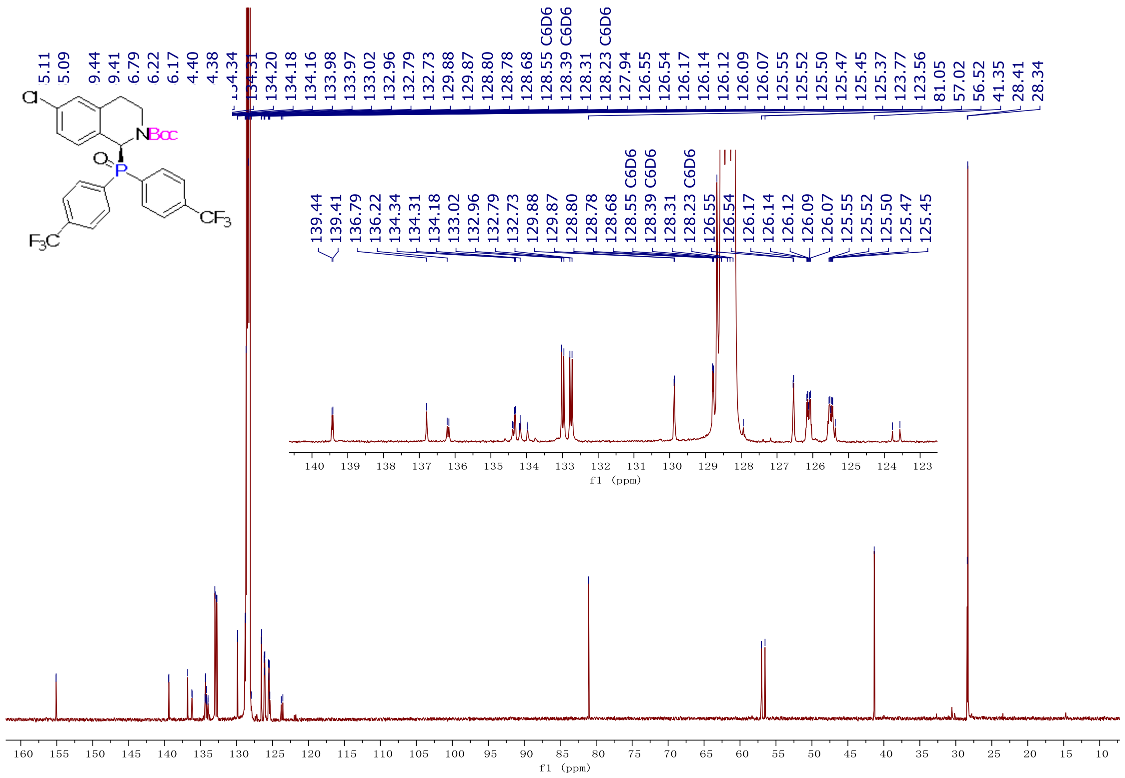

^{13}C NMR of compound **4iba**

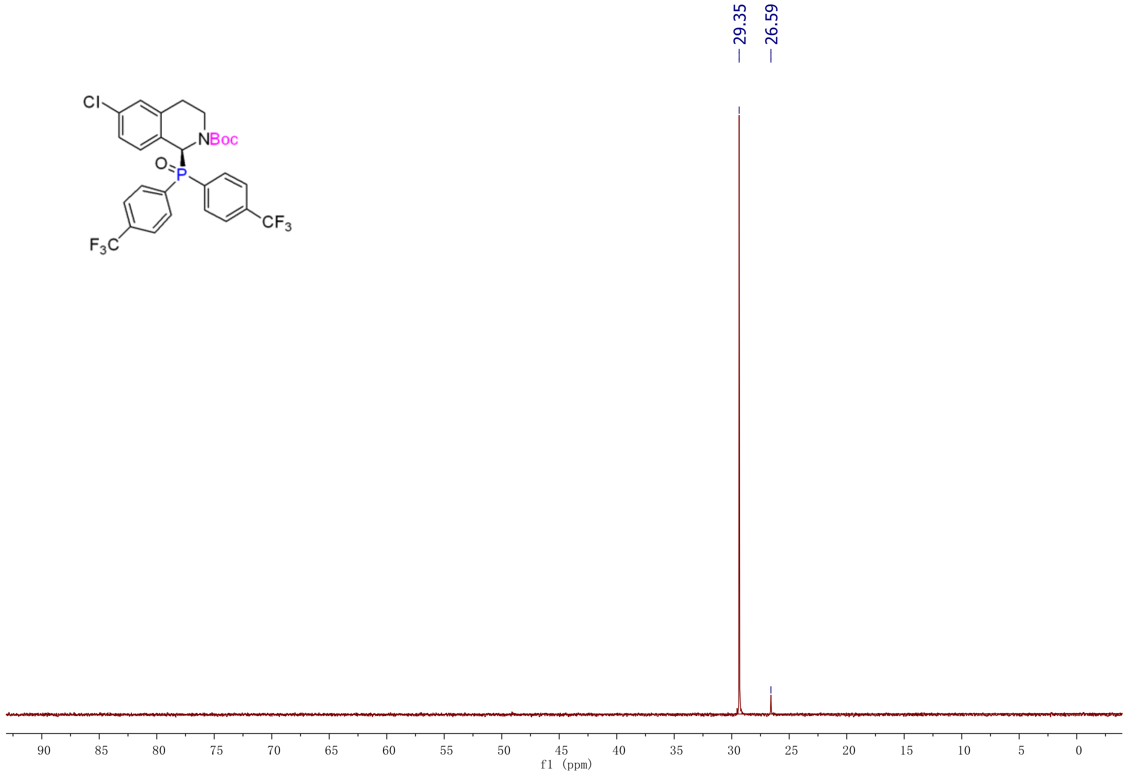

^{31}P NMR of compound **4iba**

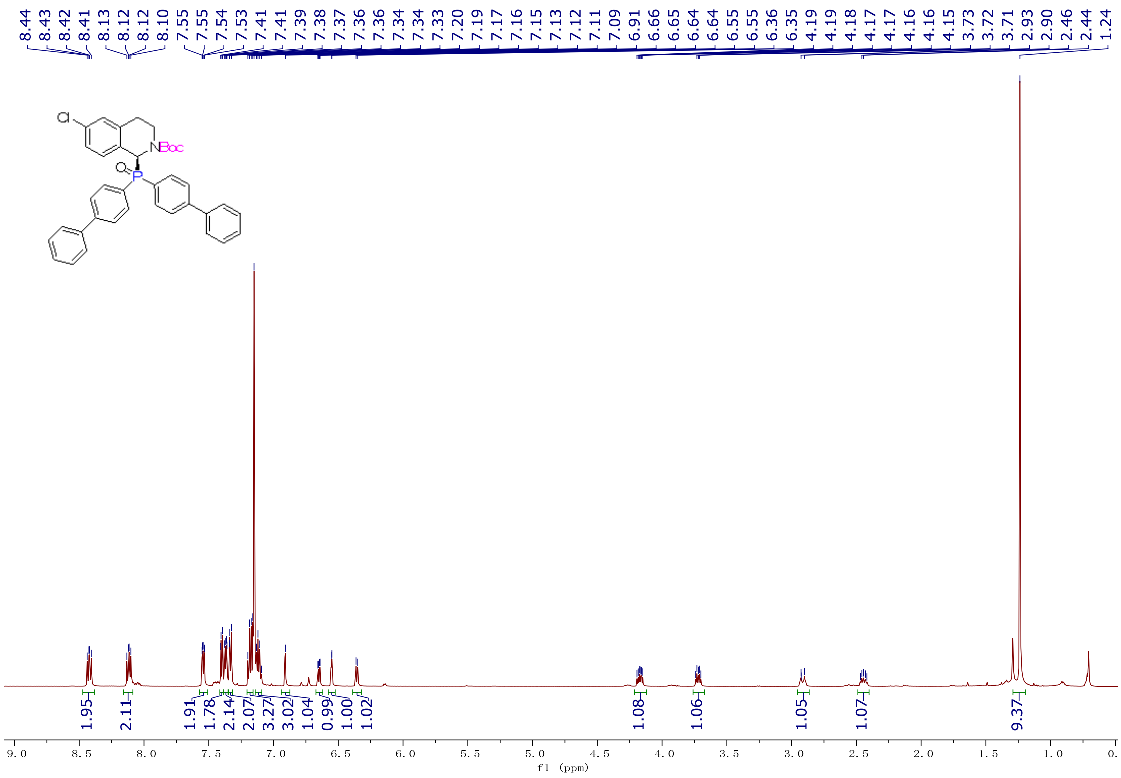

^{1}H NMR of compound **4ica**

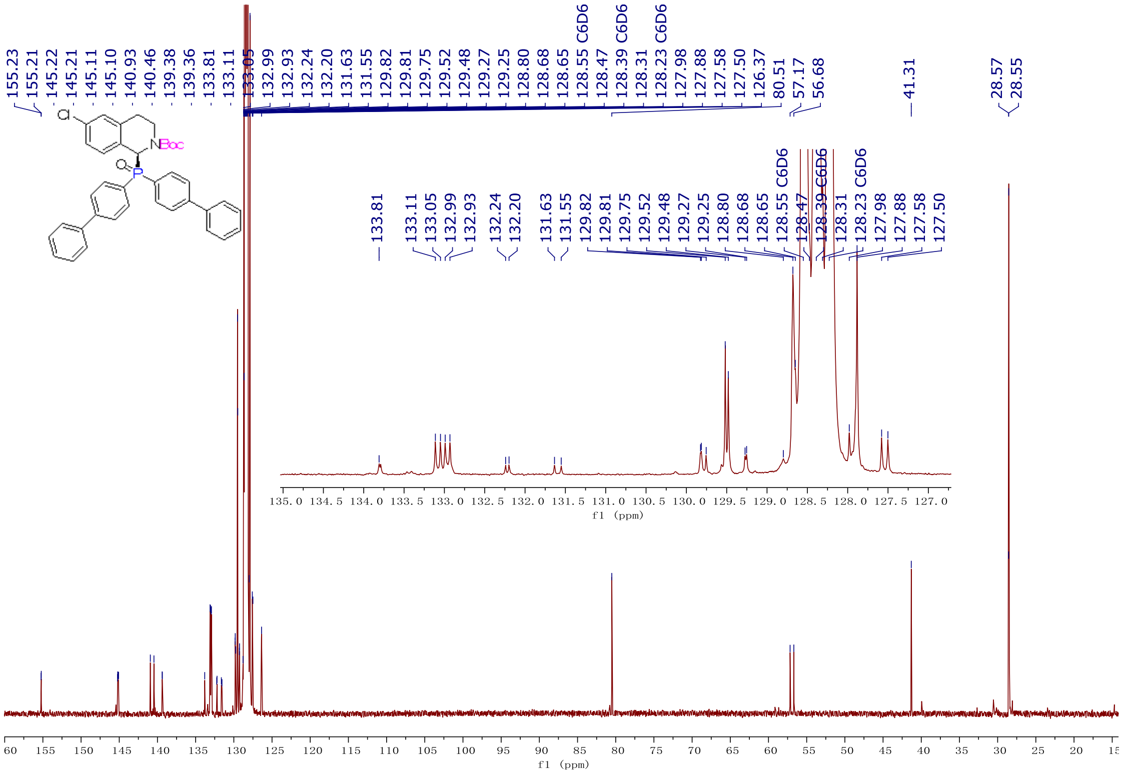

^{13}C NMR of compound **4ica**

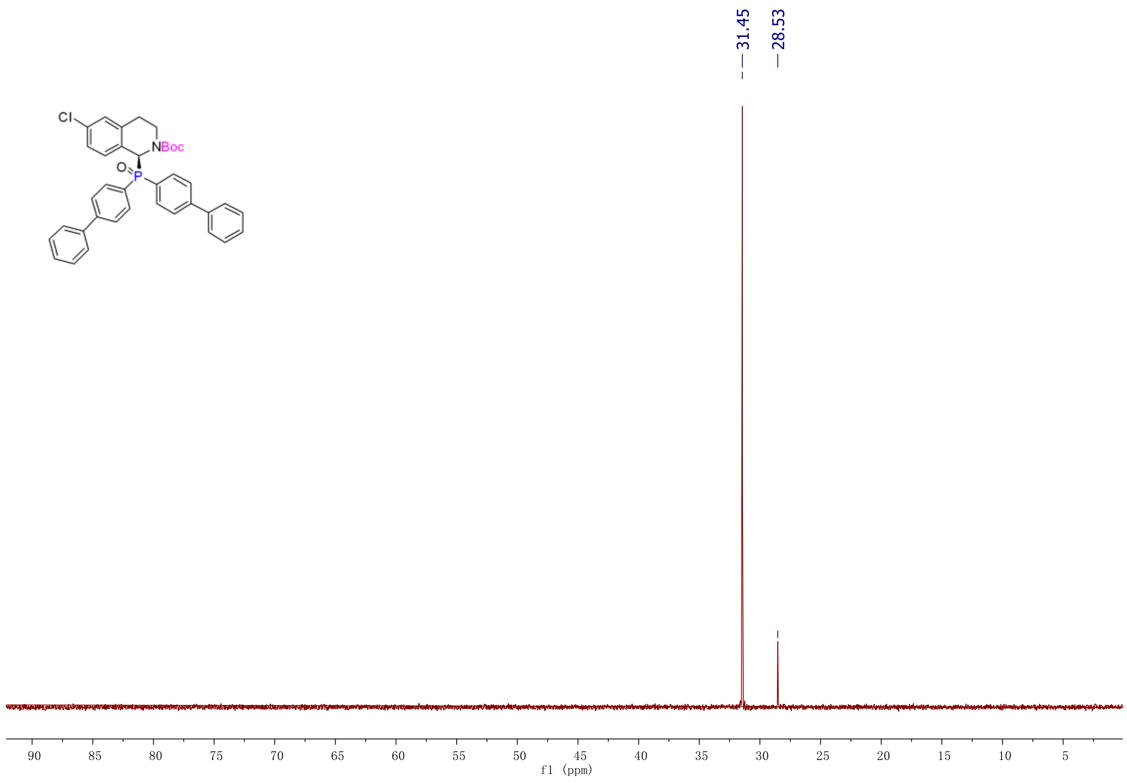

^{31}P NMR of compound **4ica**

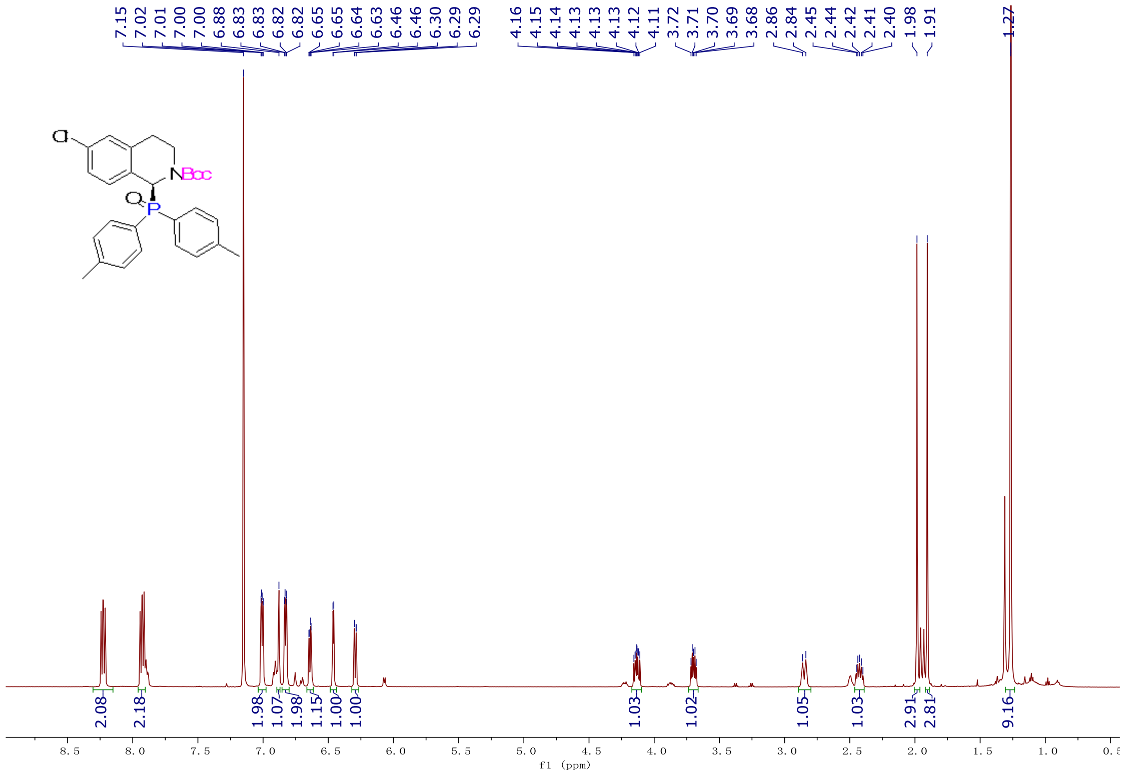

^{1}H NMR of compound **4ida**

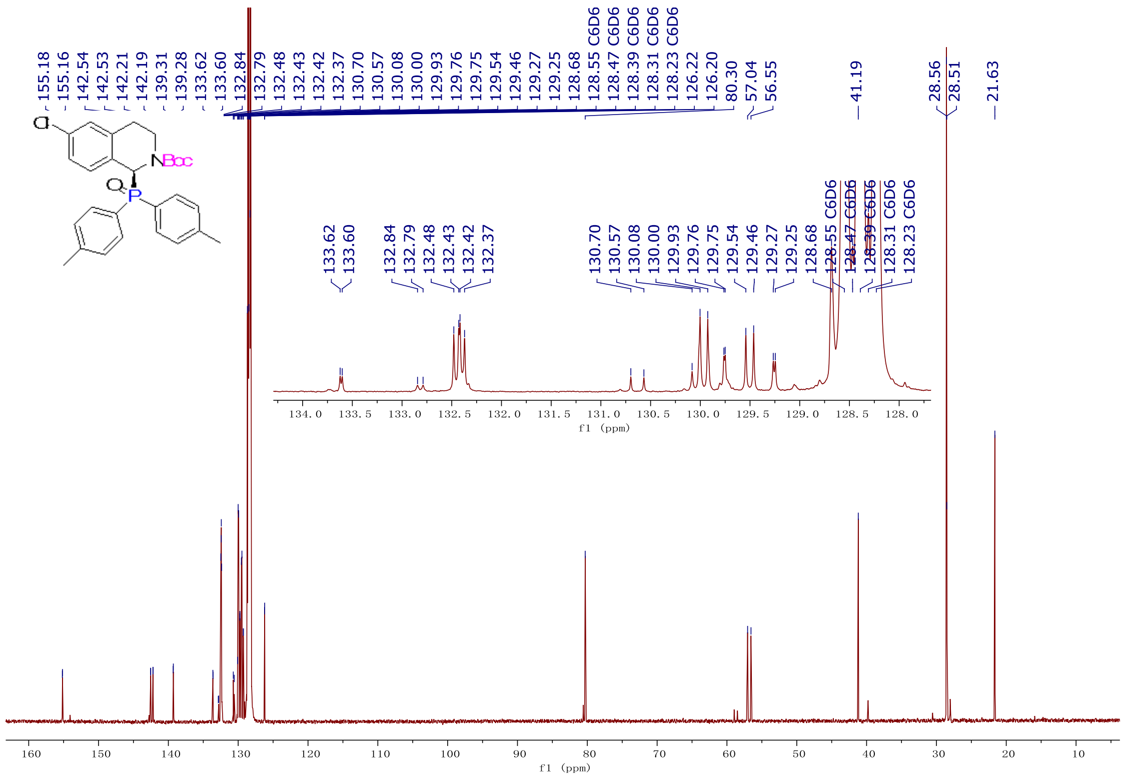

^{13}C NMR of compound **4ida**

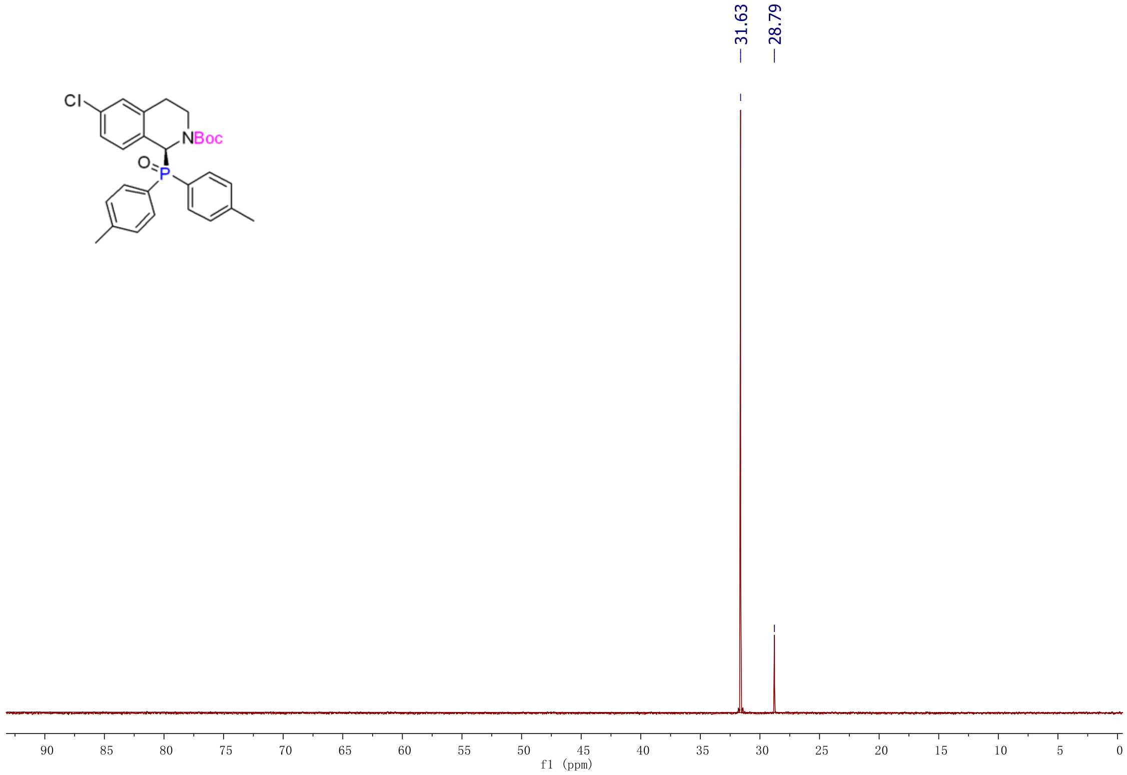

^{31}P NMR of compound **4ida**

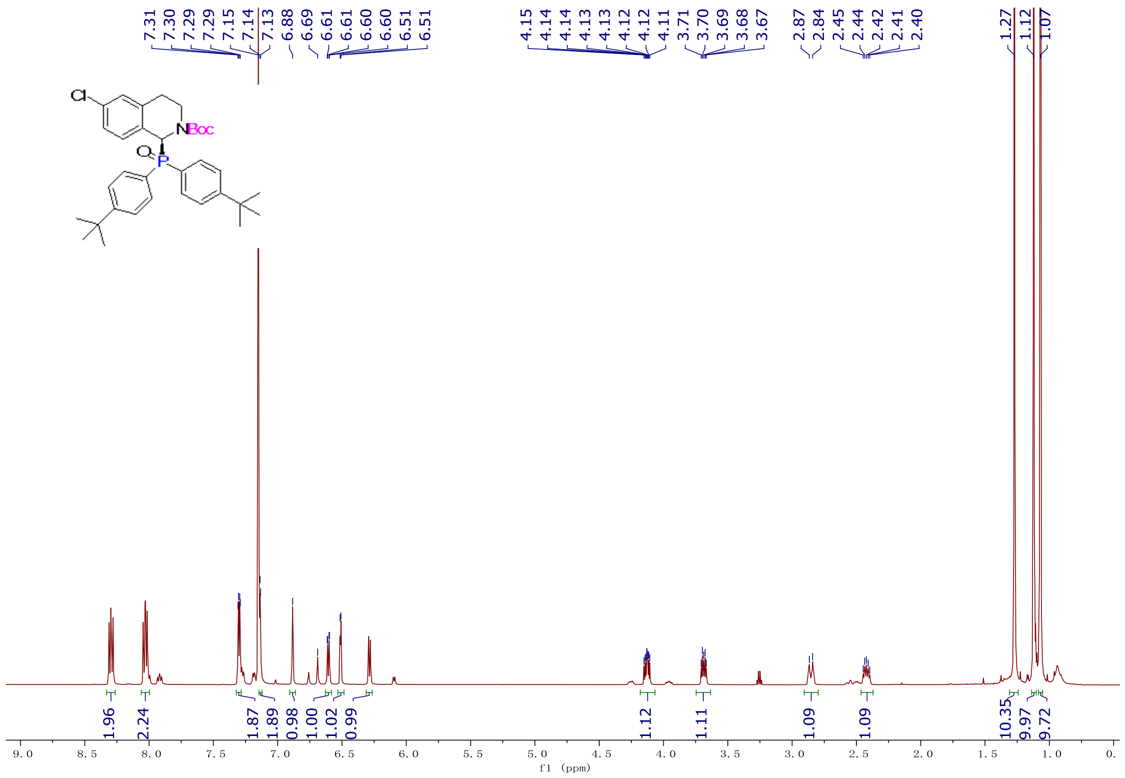

^{1}H NMR of compound **4iea**

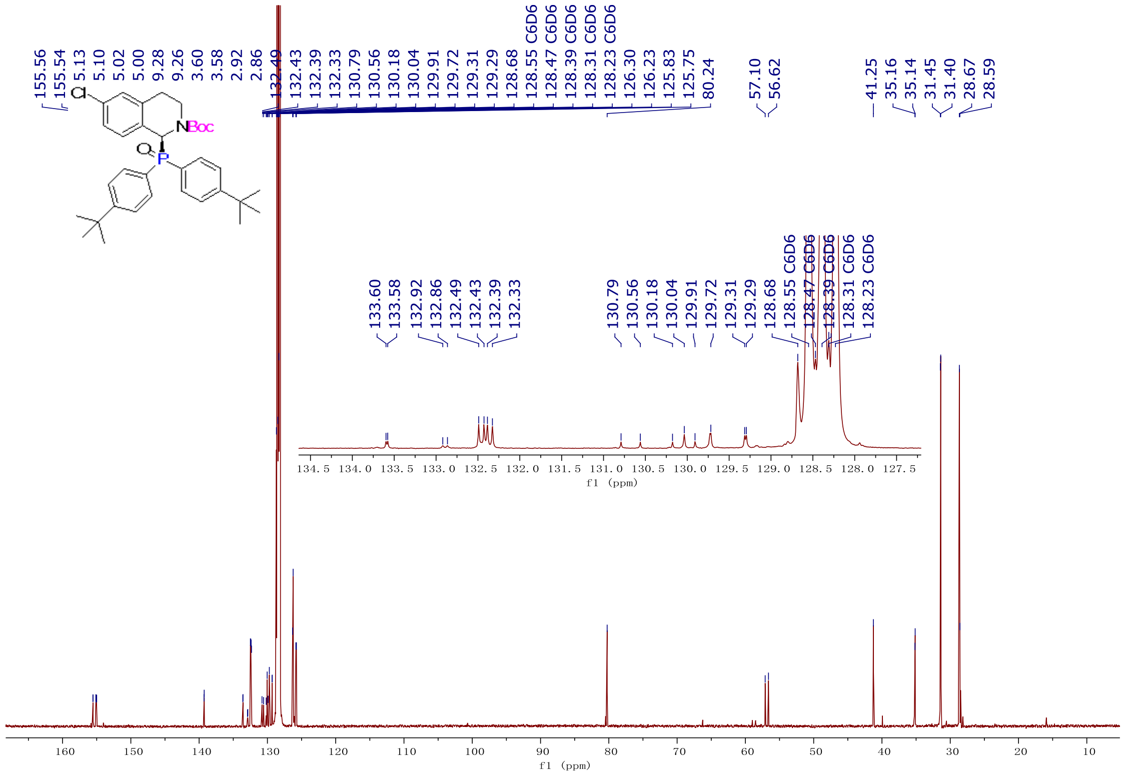

^{13}C NMR of compound **4iea**

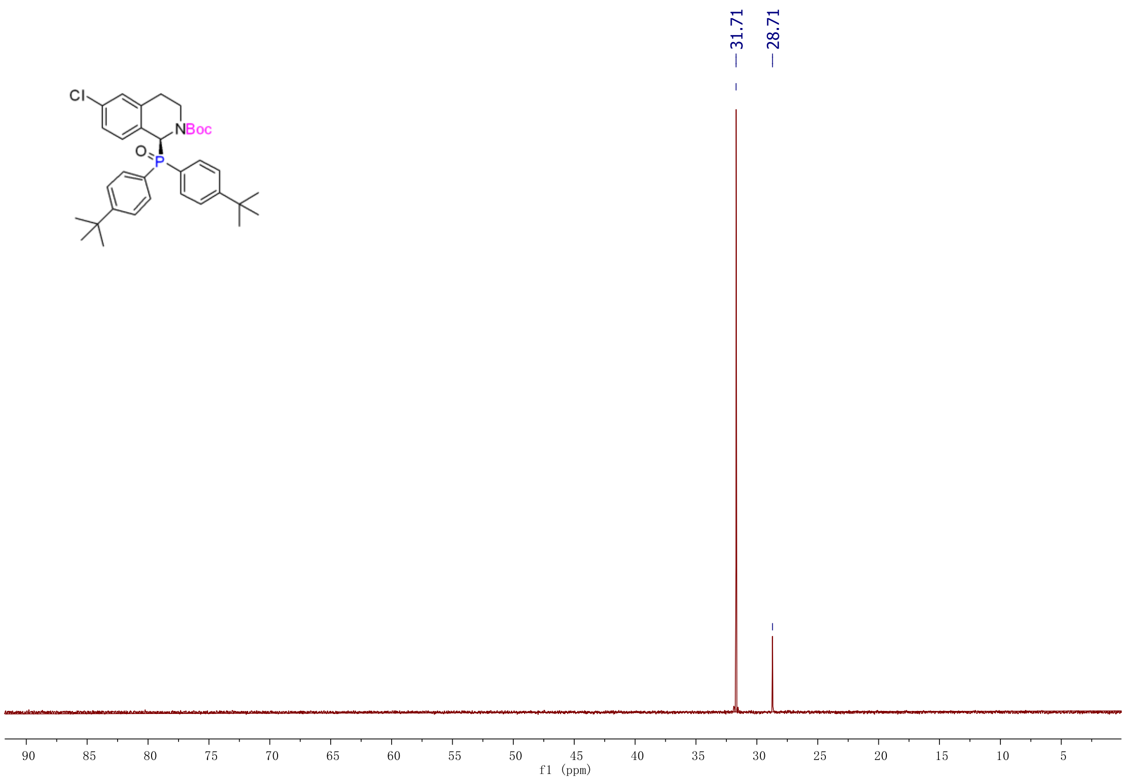

^{31}P NMR of compound **4iea**

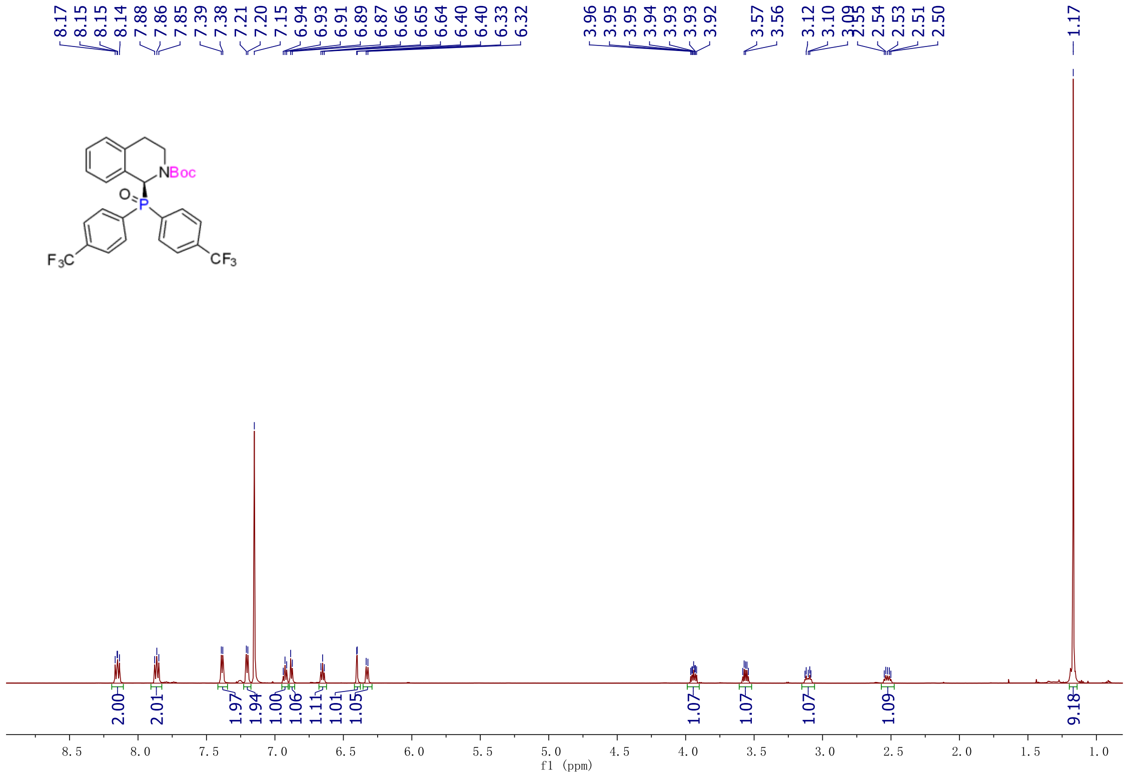

^{1}H NMR of compound **4aba**

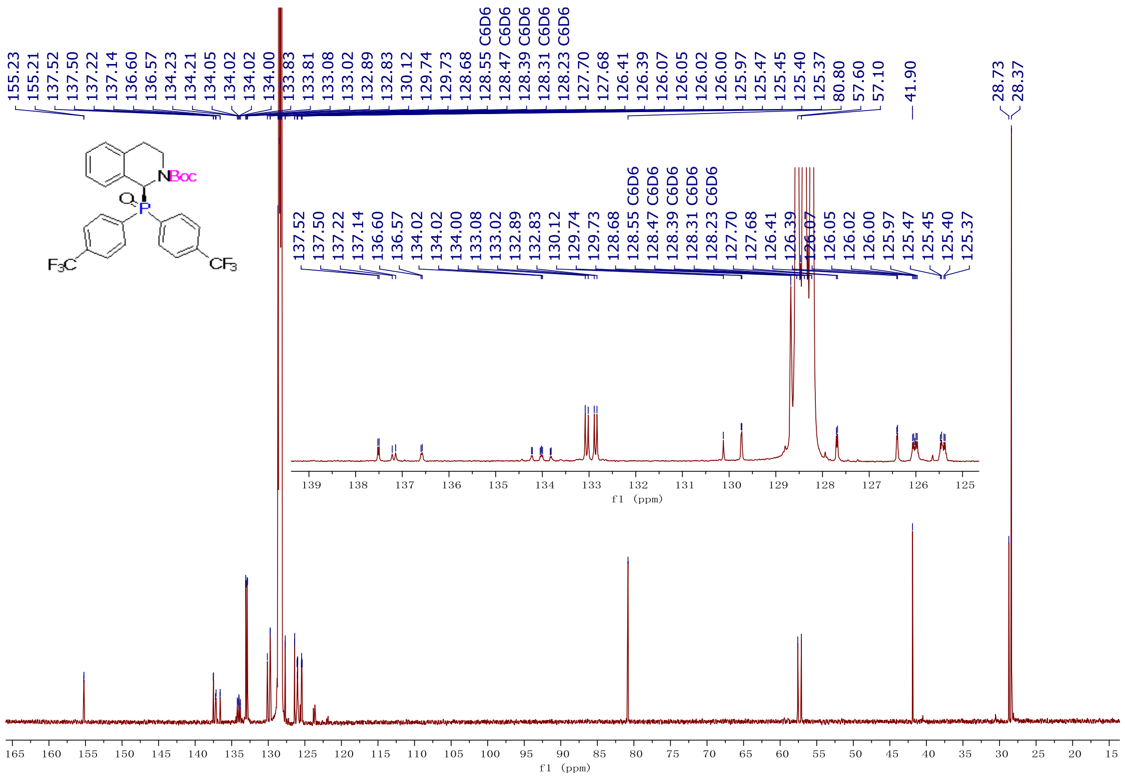

^{13}C NMR of compound **4aba**

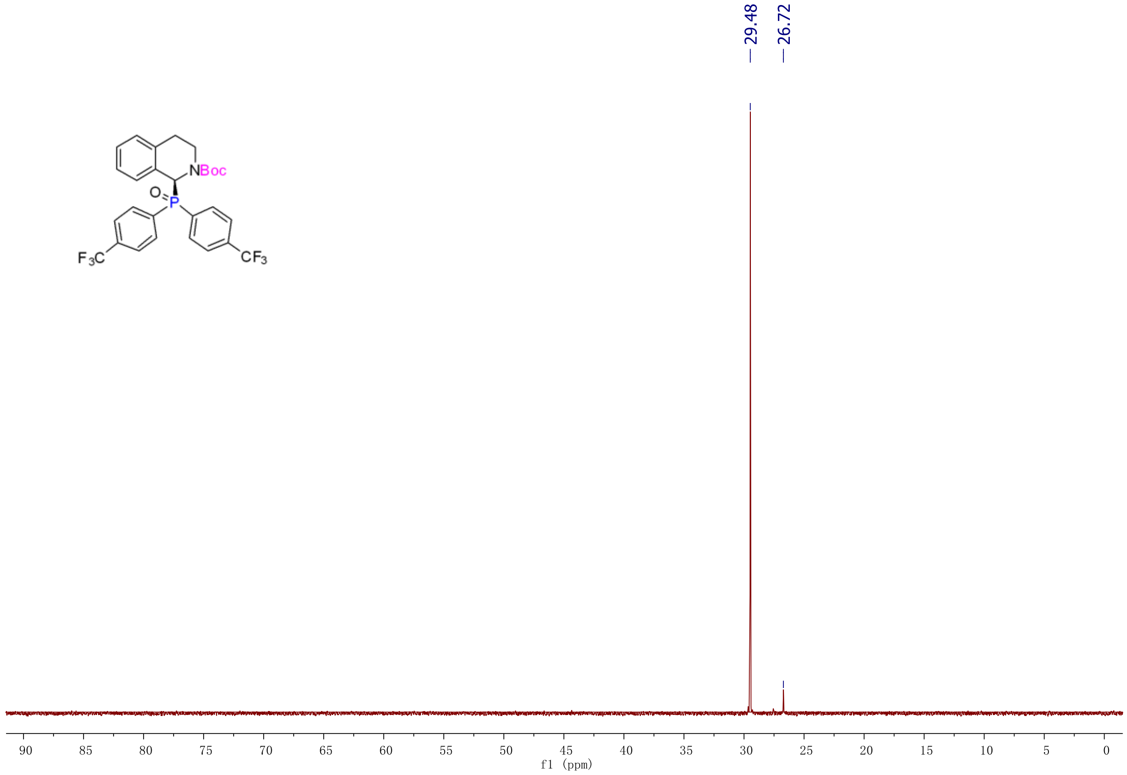

^{31}P NMR of compound **4aba**

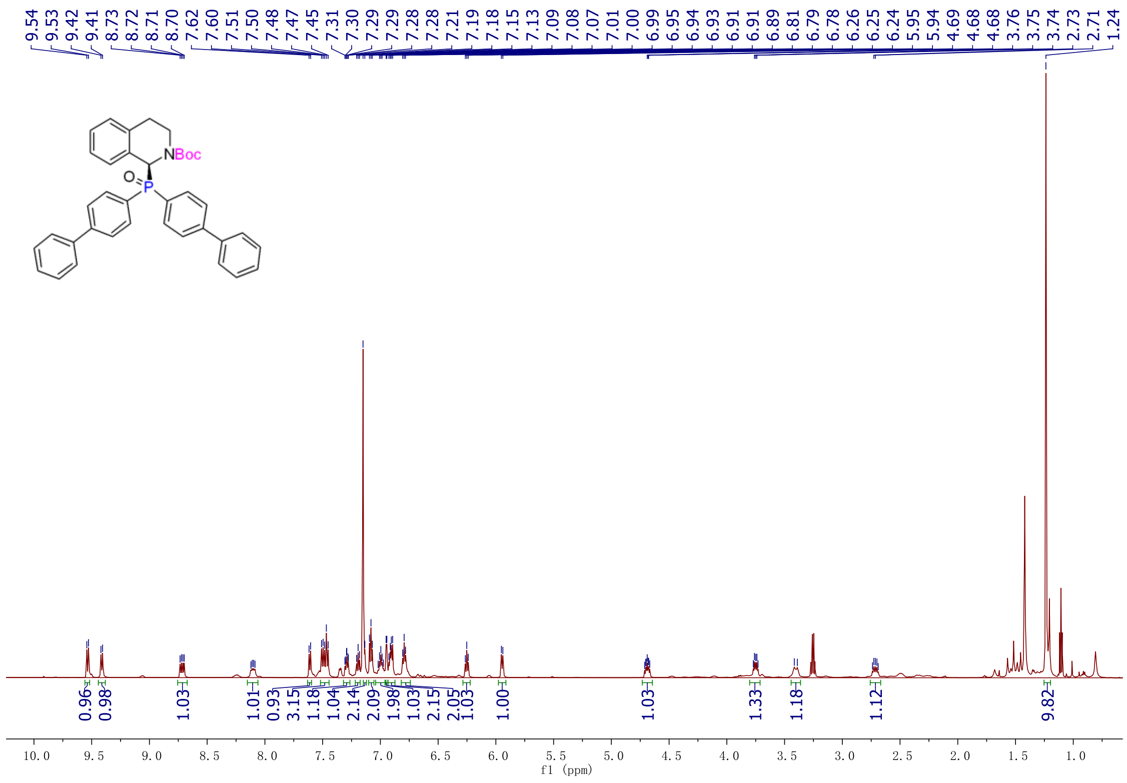

^{1}H NMR of compound **4aca**

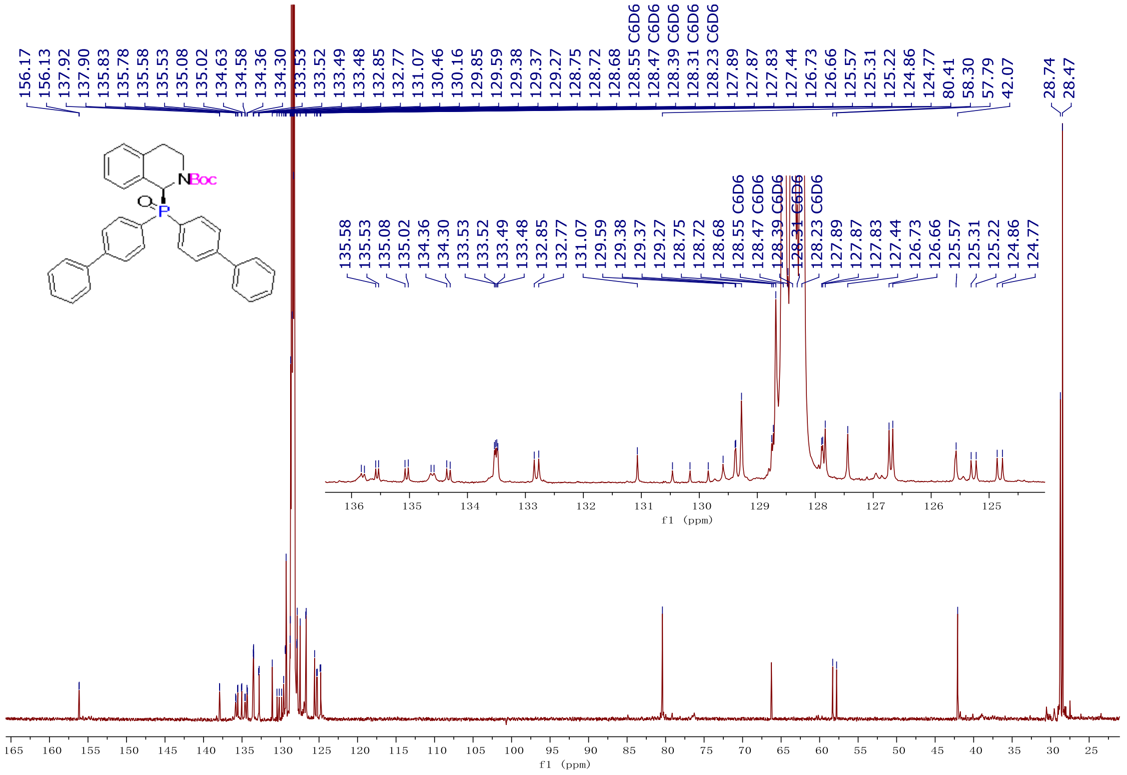

^{13}C NMR of compound **4aca**

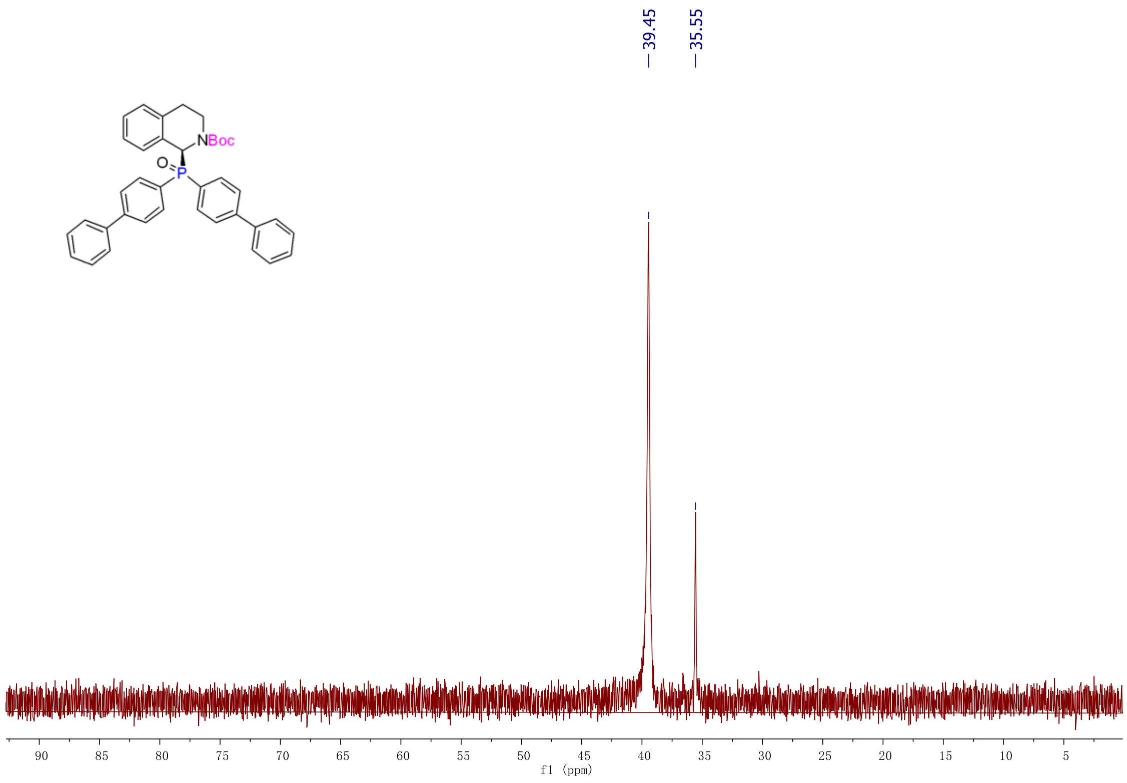

^{31}P NMR of compound **4aca**

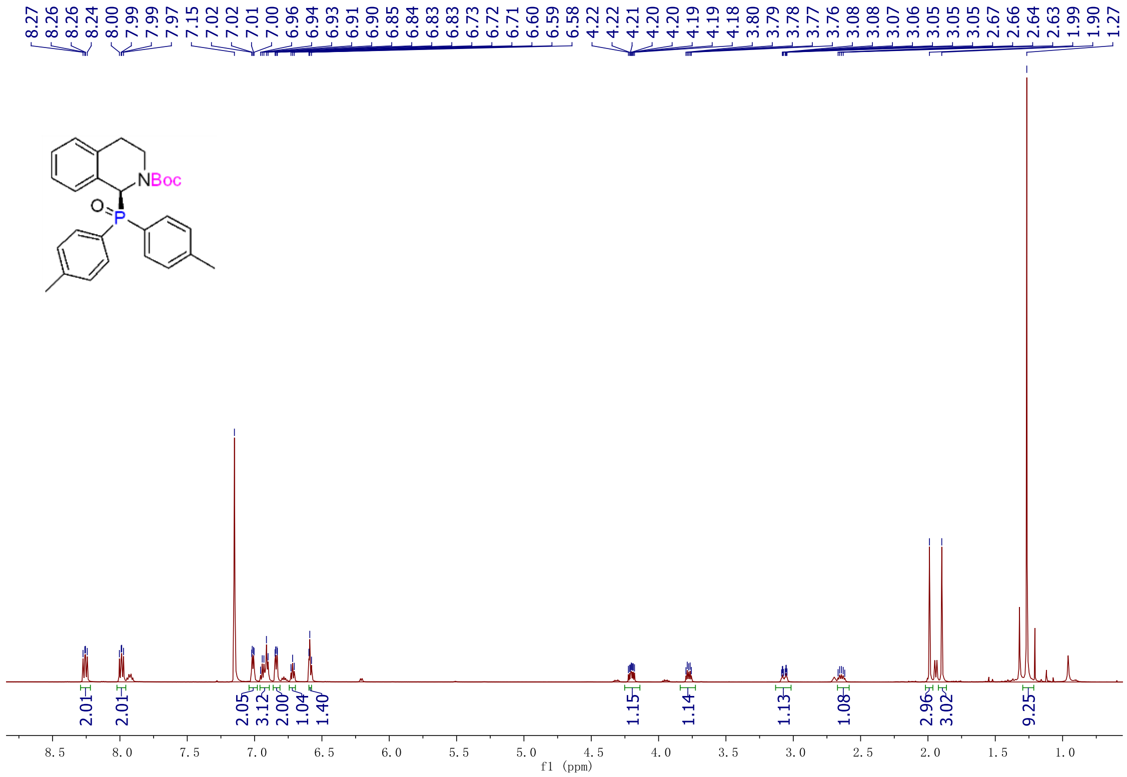

^{1}H NMR of compound **4ada**

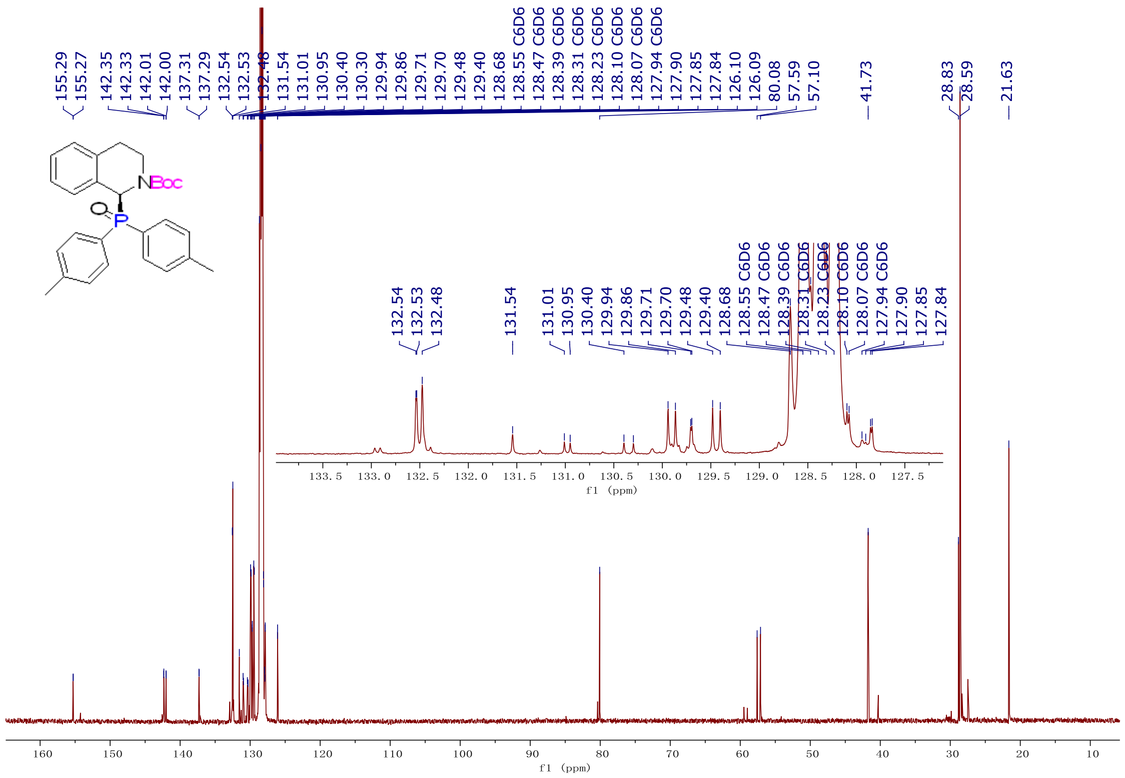

^{13}C NMR of compound **4ada**

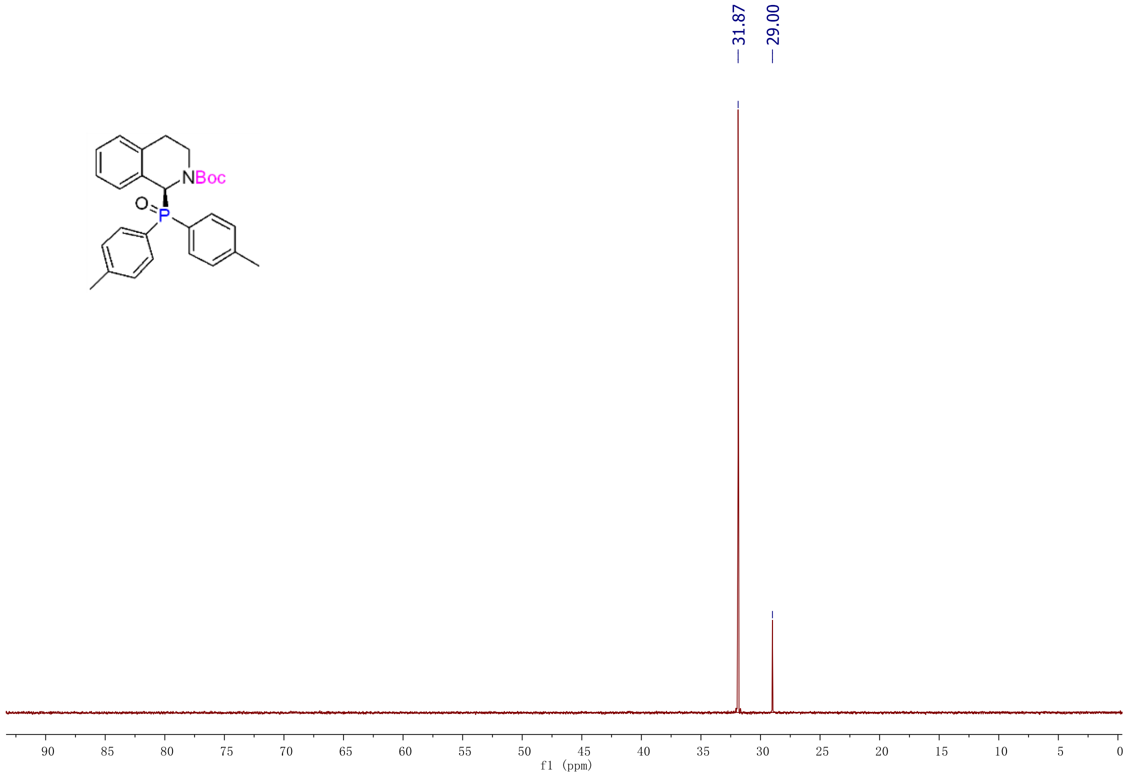

^{31}P NMR of compound **4ada**

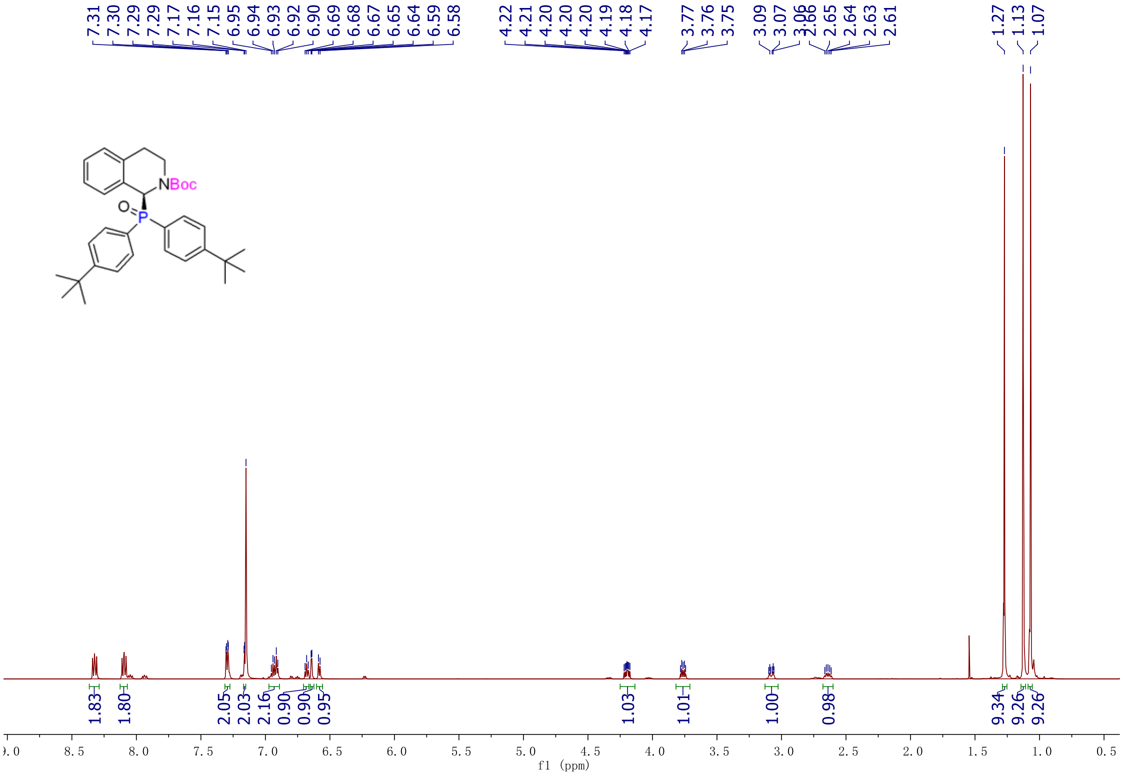

^{1}H NMR of compound **4aea**

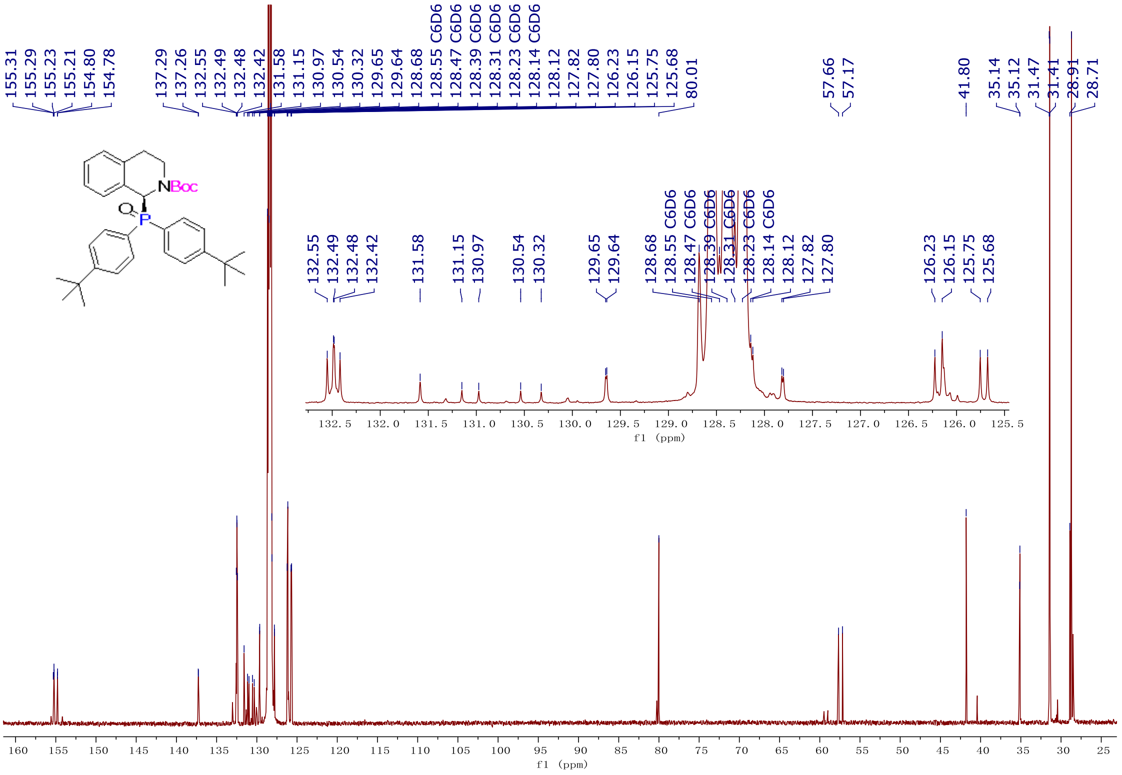

^{13}C NMR of compound **4aea**

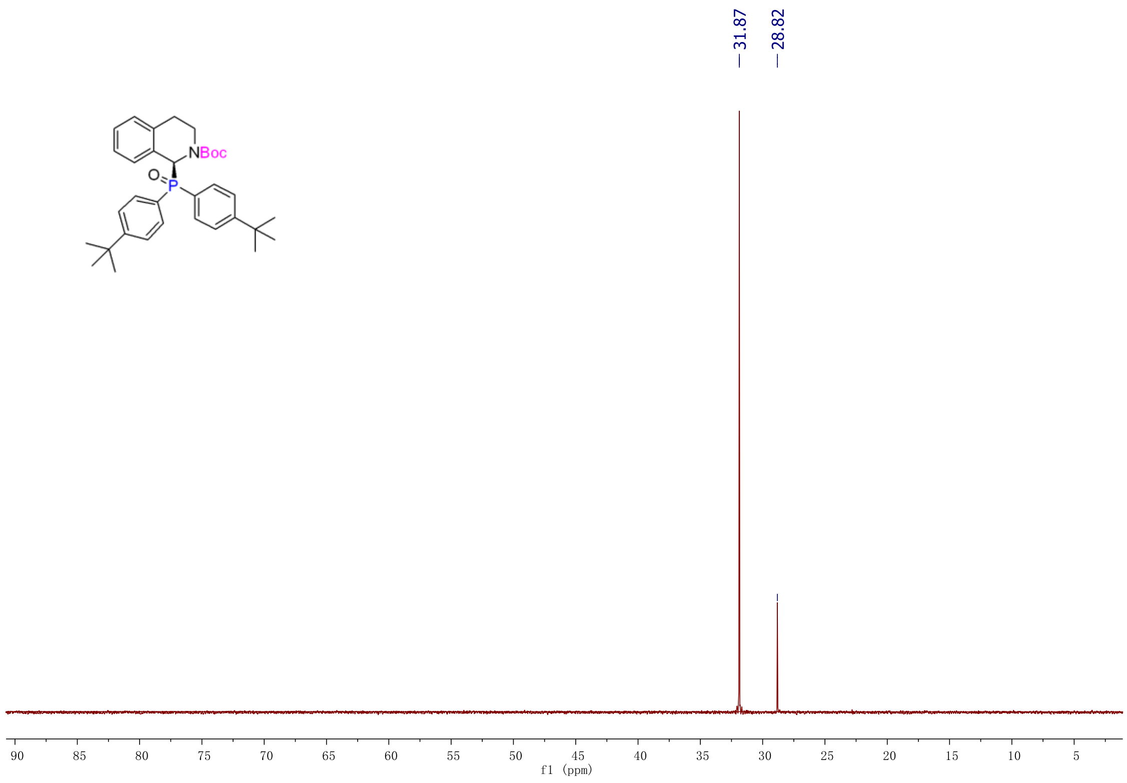

^{31}P NMR of compound **4aea**

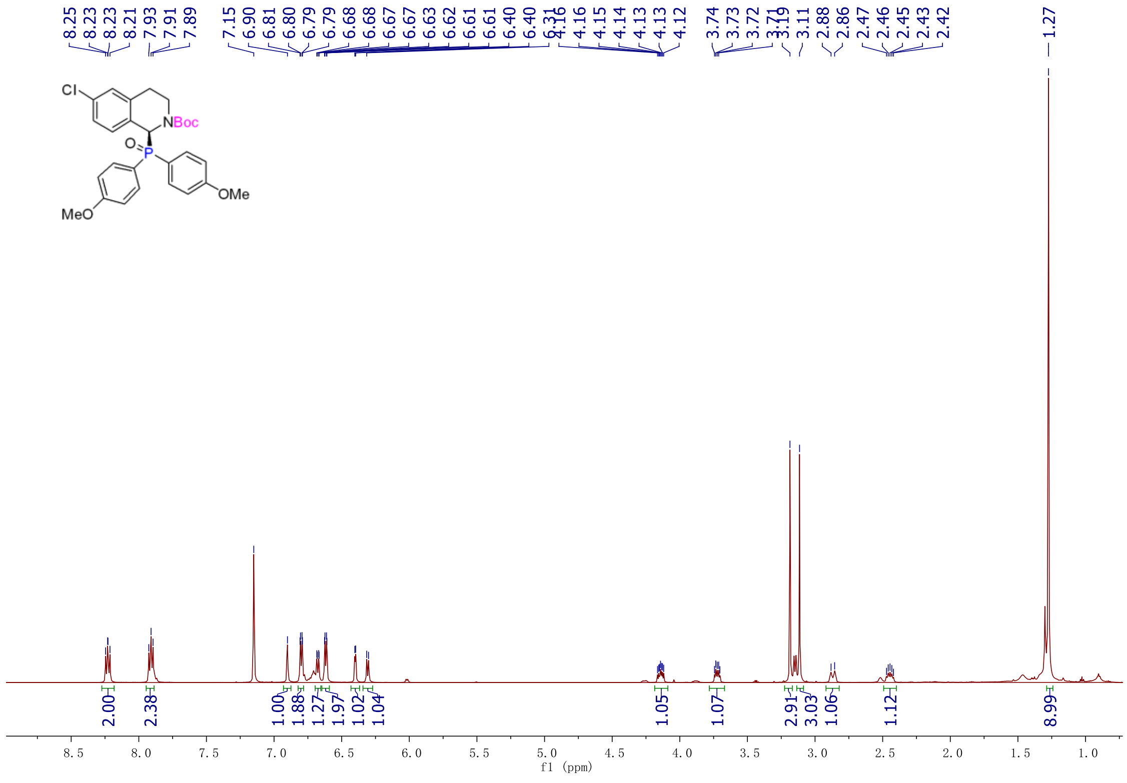

^{1}H NMR of compound **4ifa**

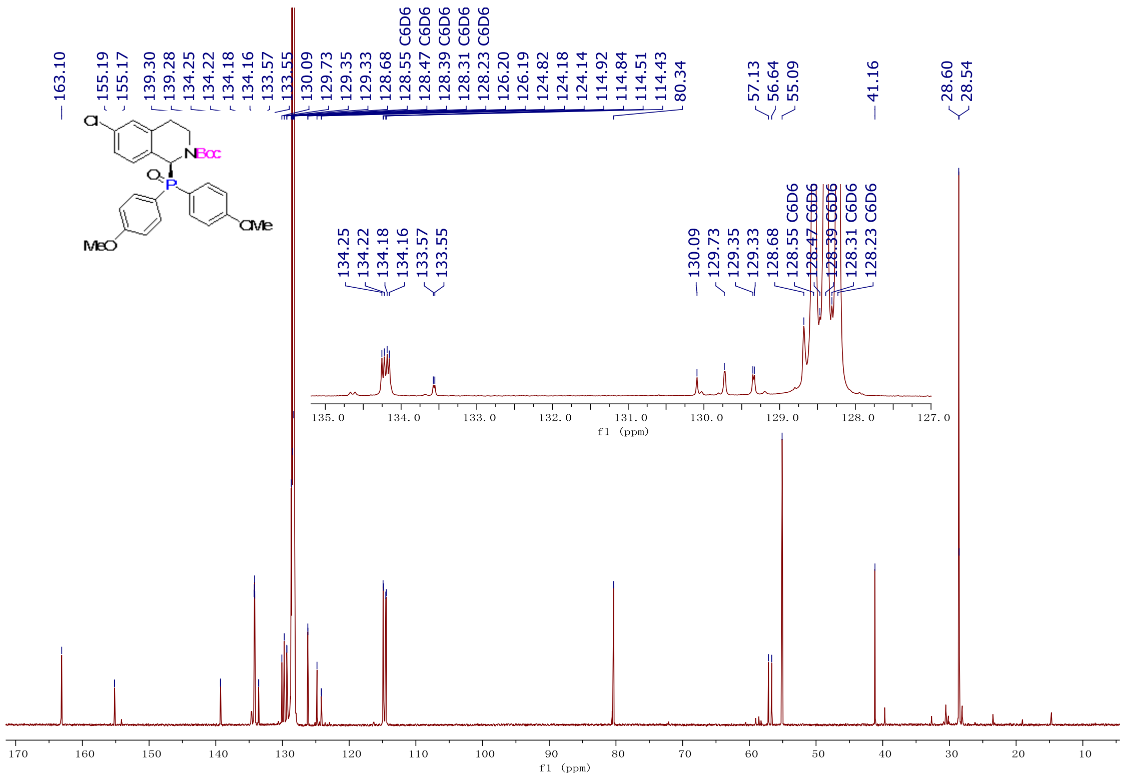

^{13}C NMR of compound **4ifa**

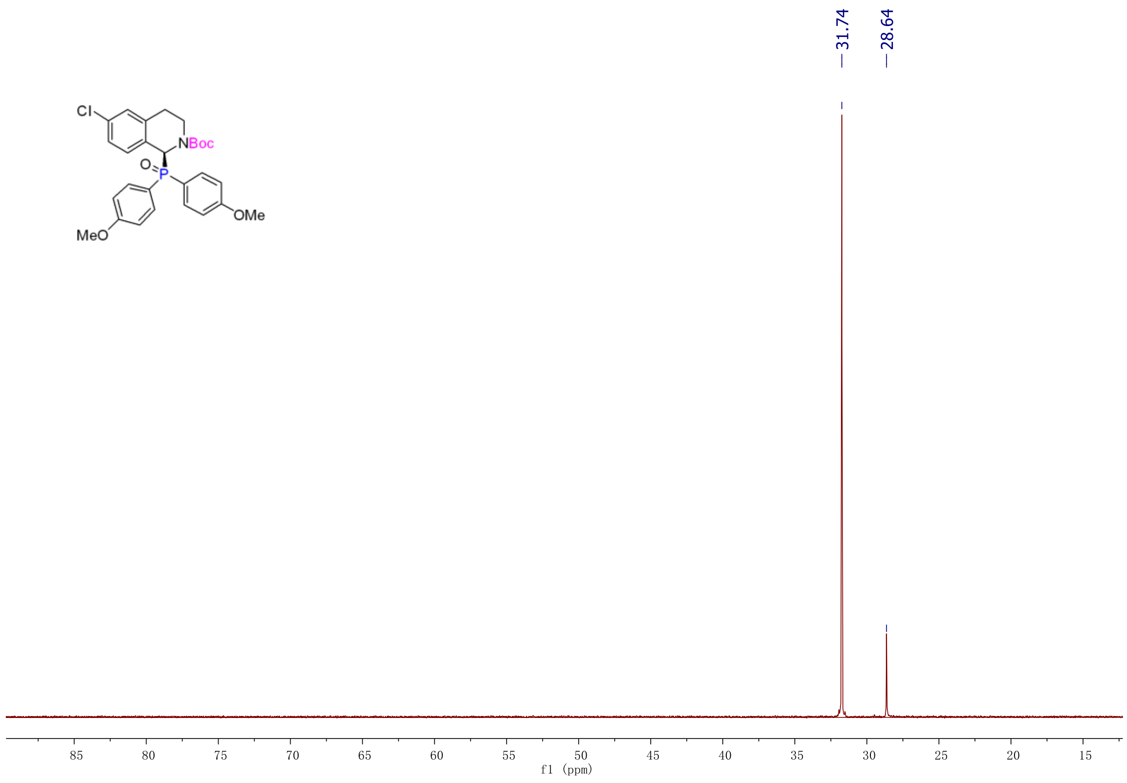

^{31}P NMR of compound **4ifa**

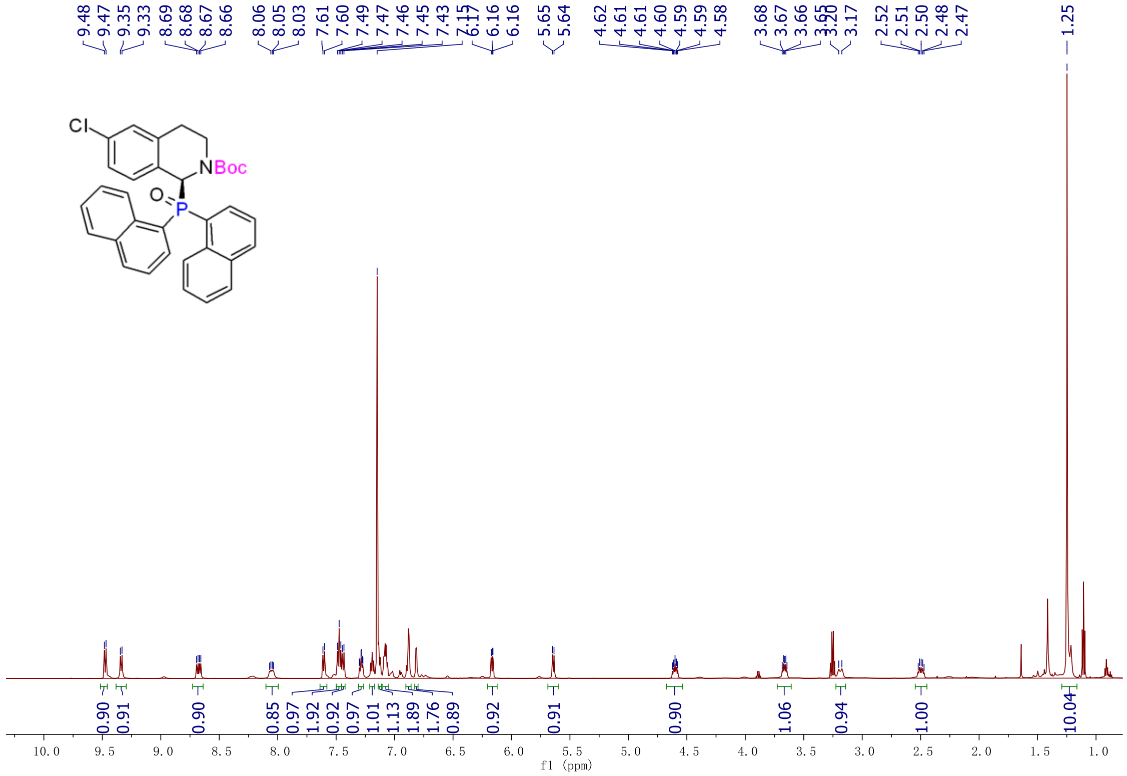

^{1}H NMR of compound **4iga**

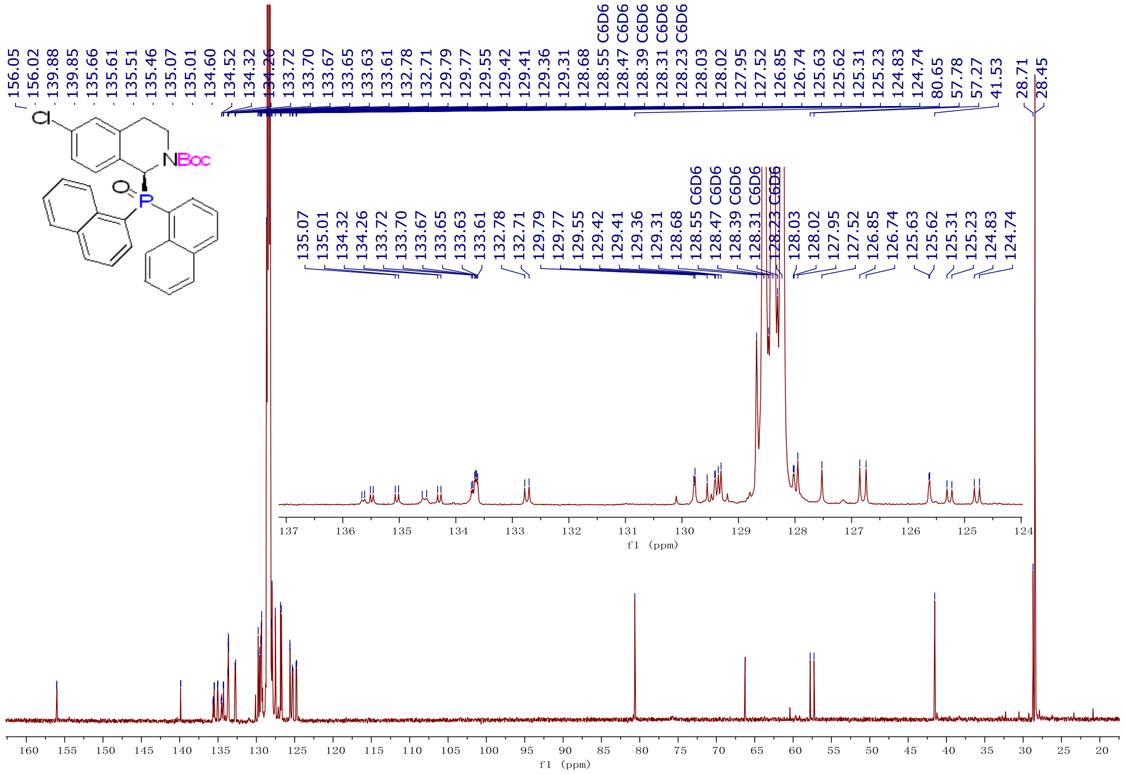

^{13}C NMR of compound **4iga**

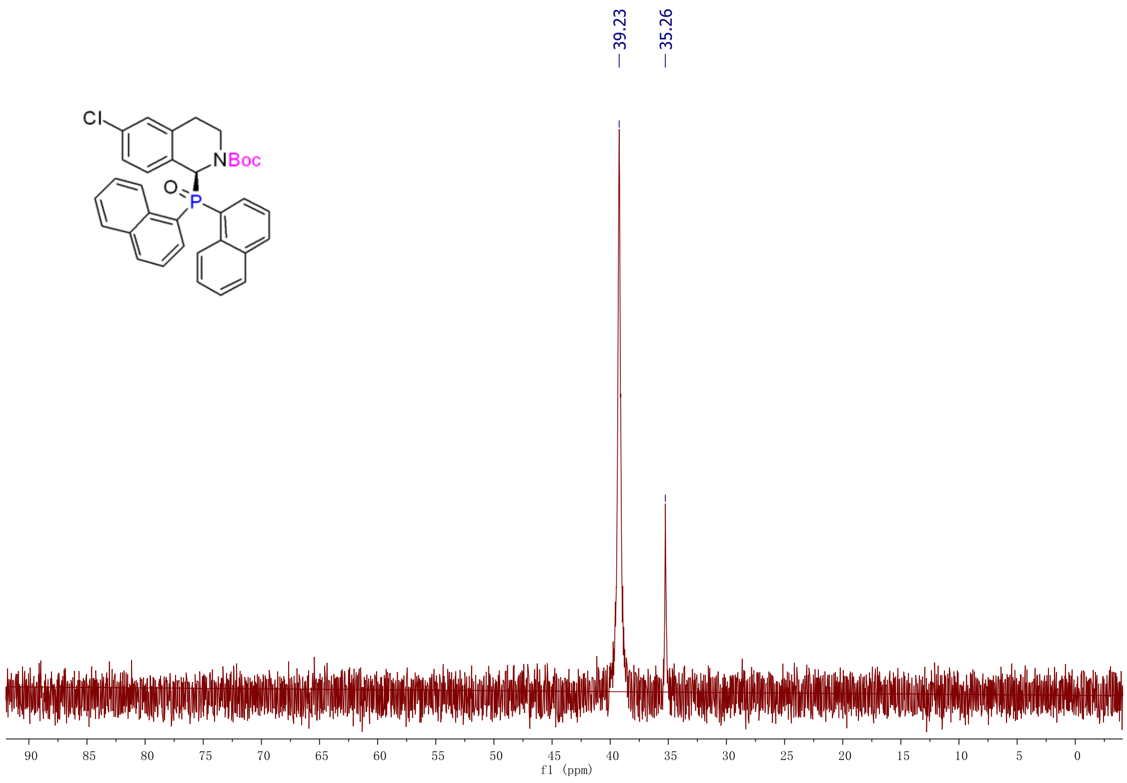

^{31}P NMR of compound **4iga**

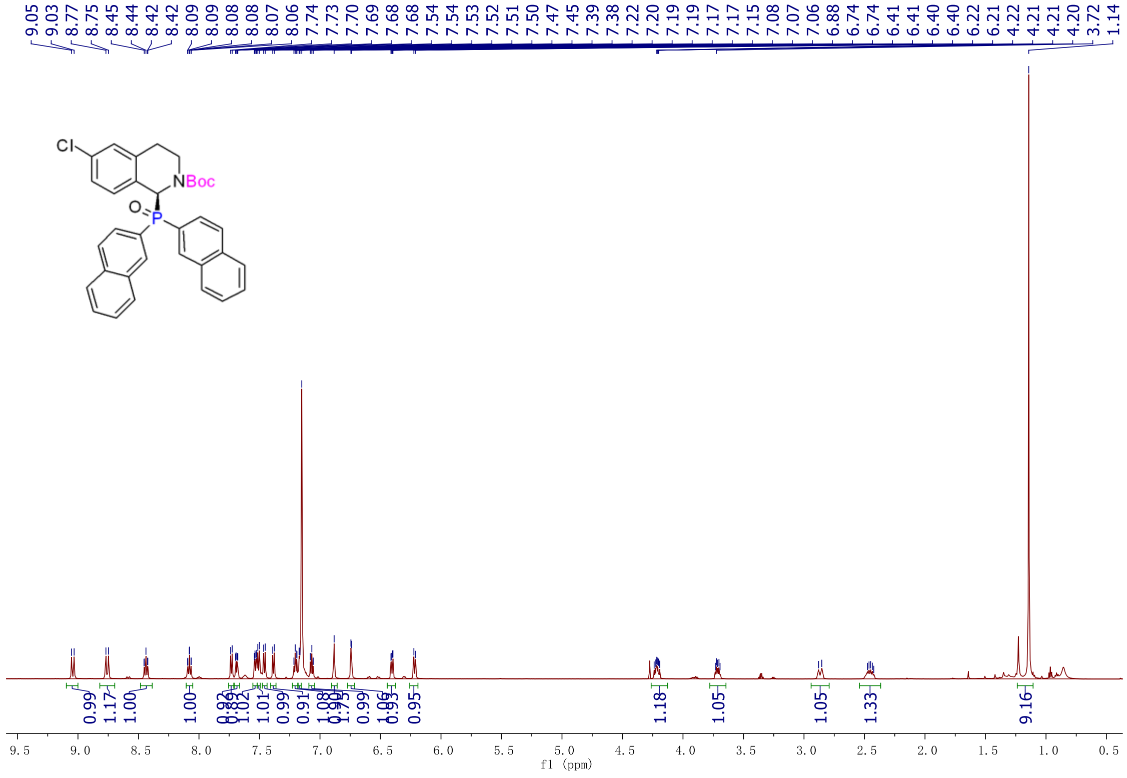

^{1}H NMR of compound **4iha**

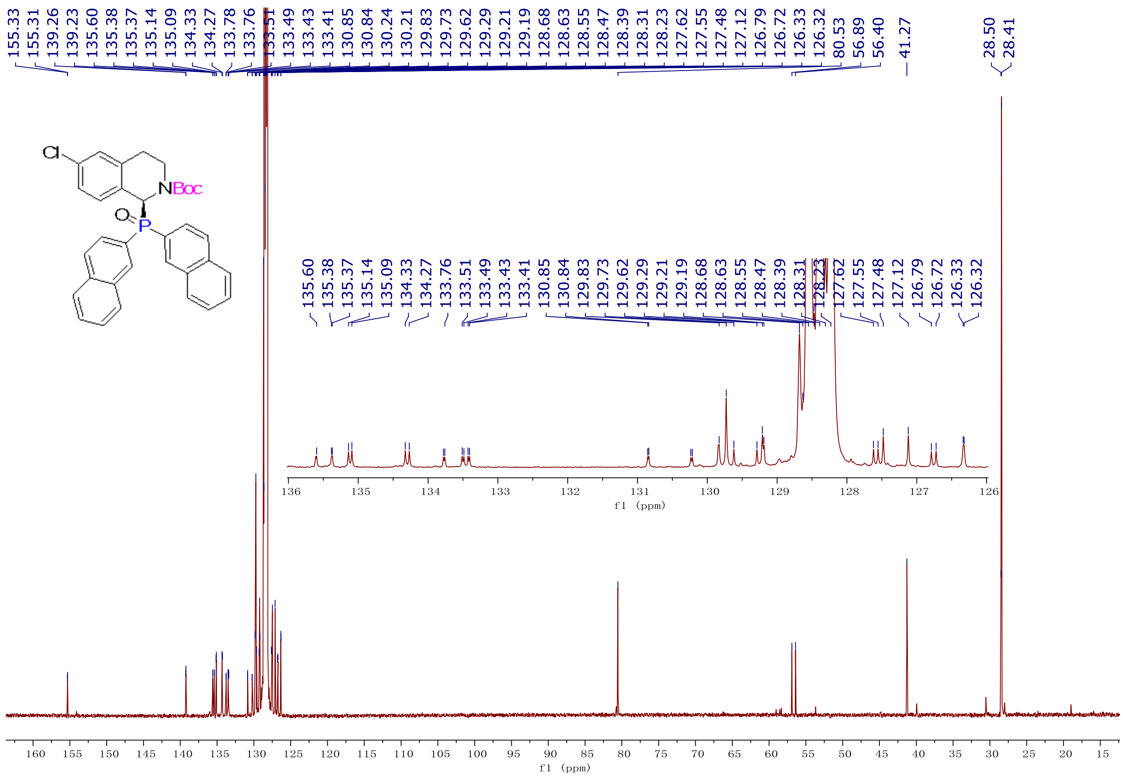

^{13}C NMR of compound **4iha**

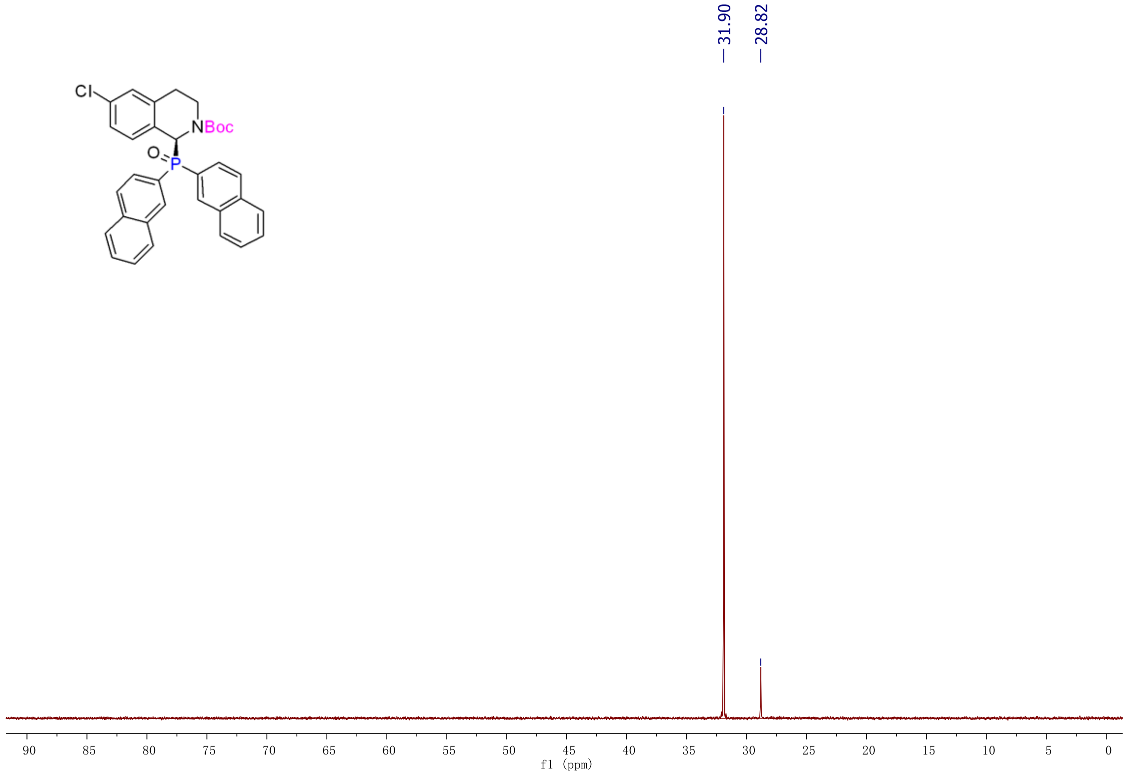

^{31}P NMR of compound **4iha**

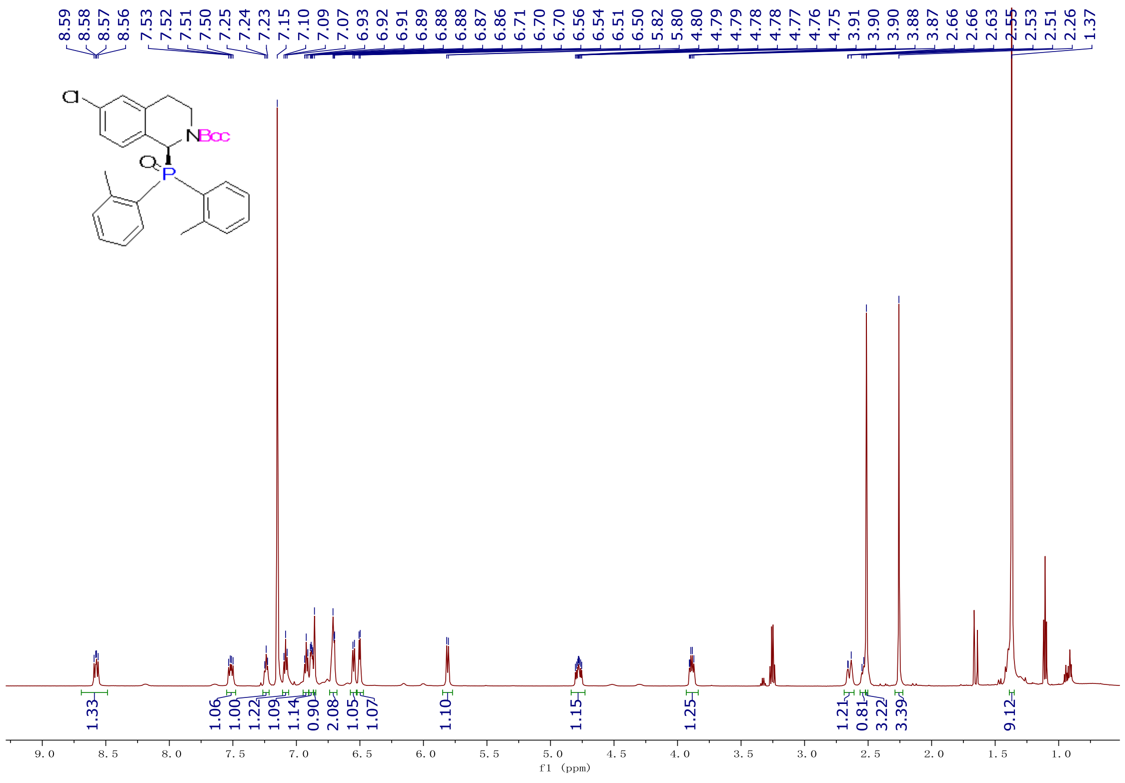

^{1}H NMR of compound **4iia**

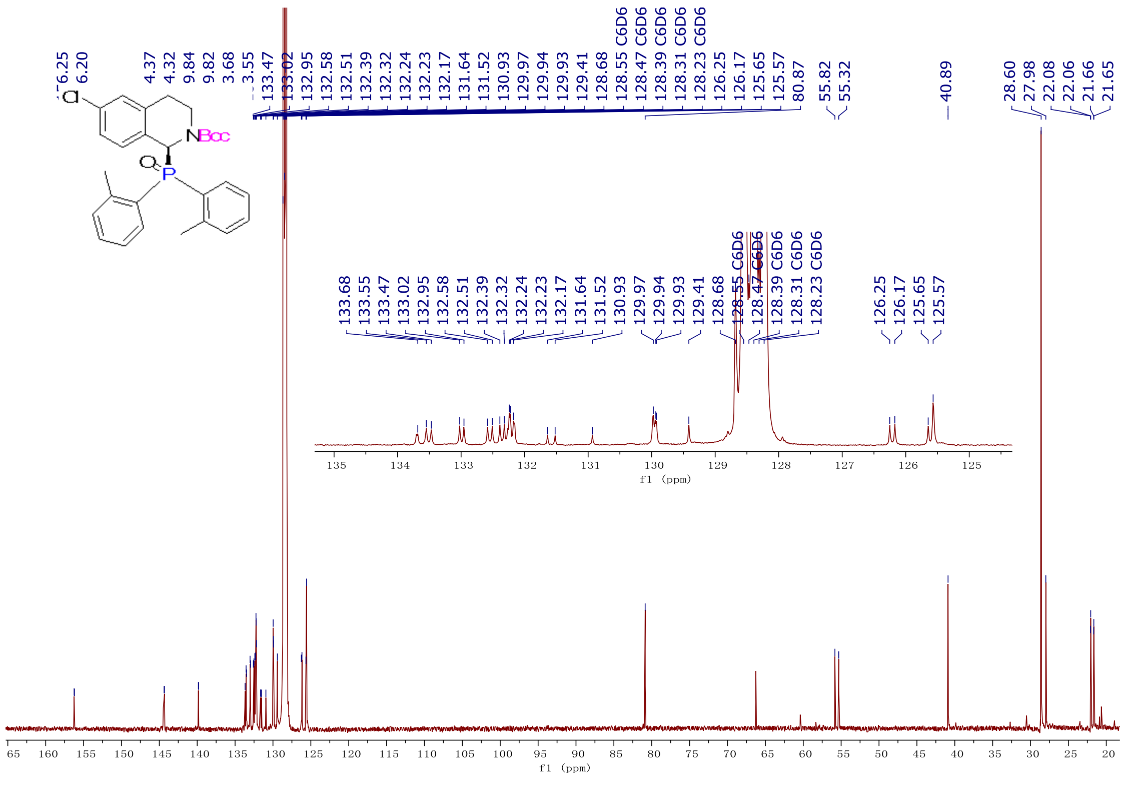

^{13}C NMR of compound **4iia**

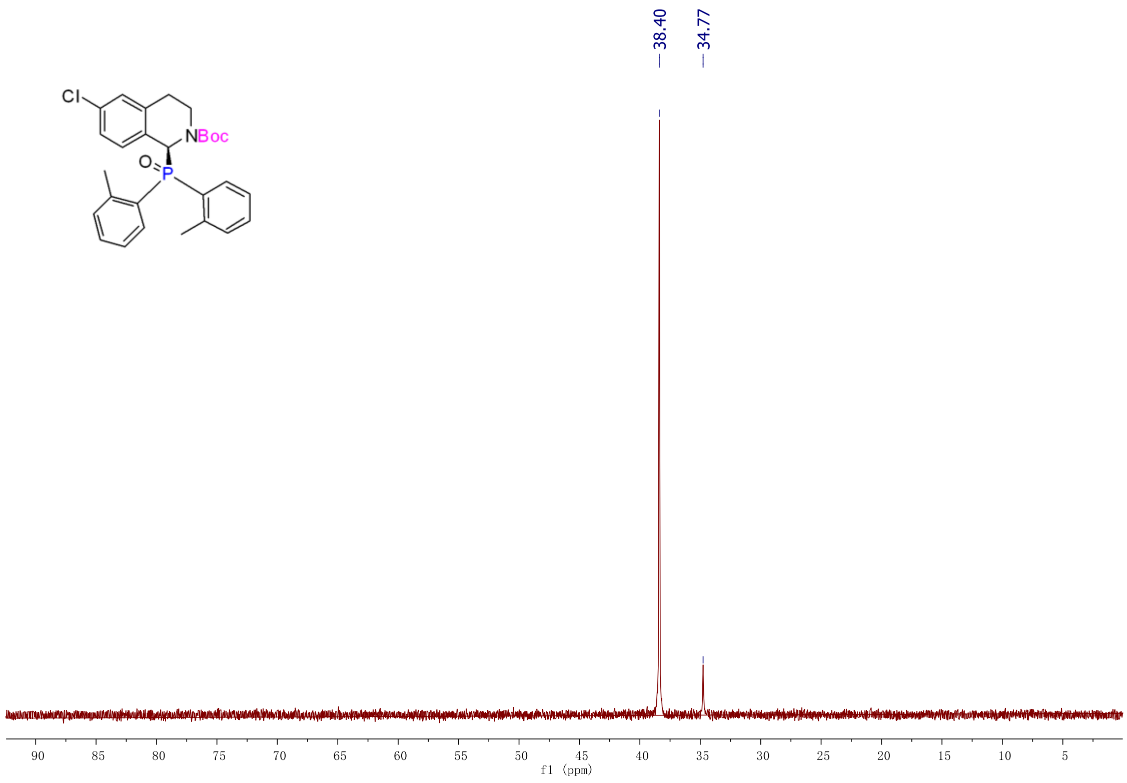

^{31}P NMR of compound **4iia**

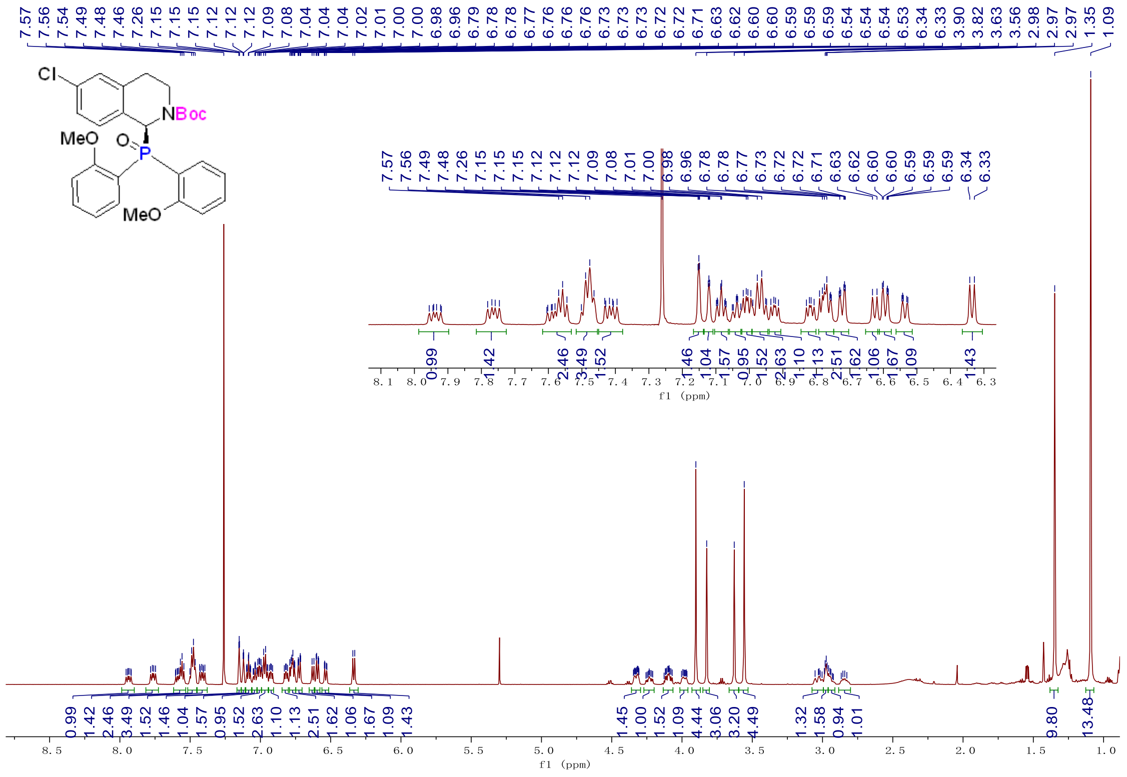

^{1}H NMR of compound **4ija**

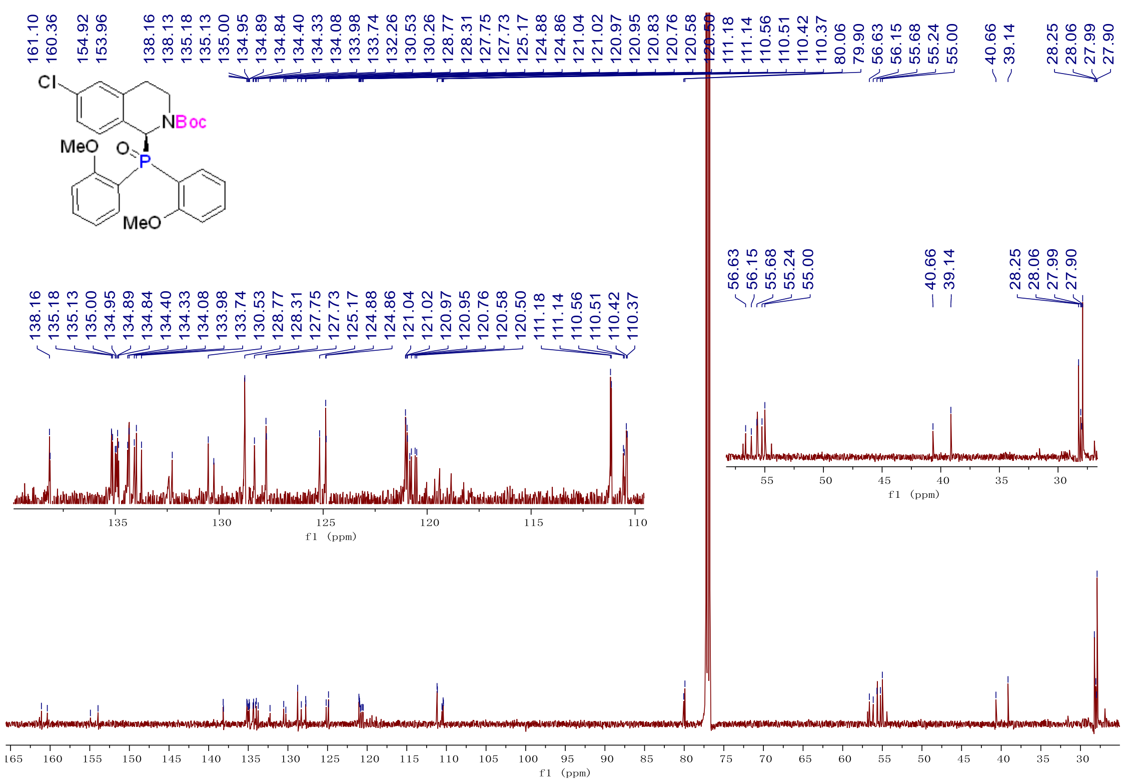

^{13}C NMR of compound **4ija**

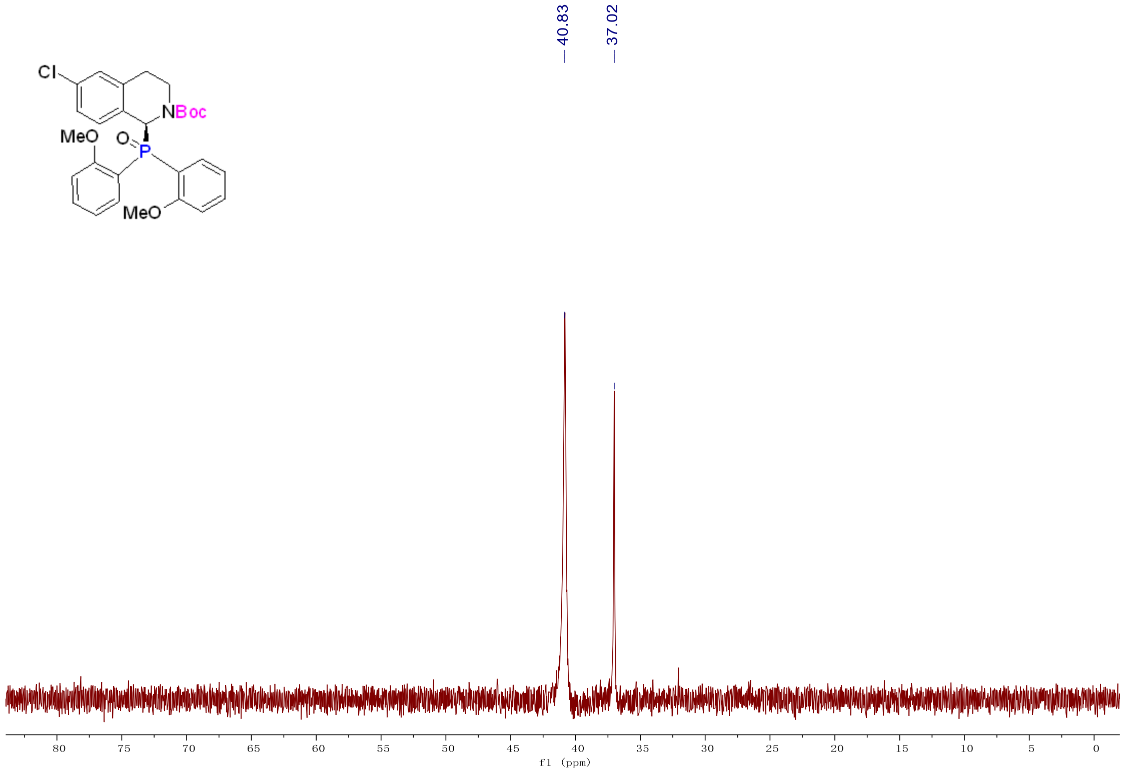

^{31}P NMR of compound **4ija**

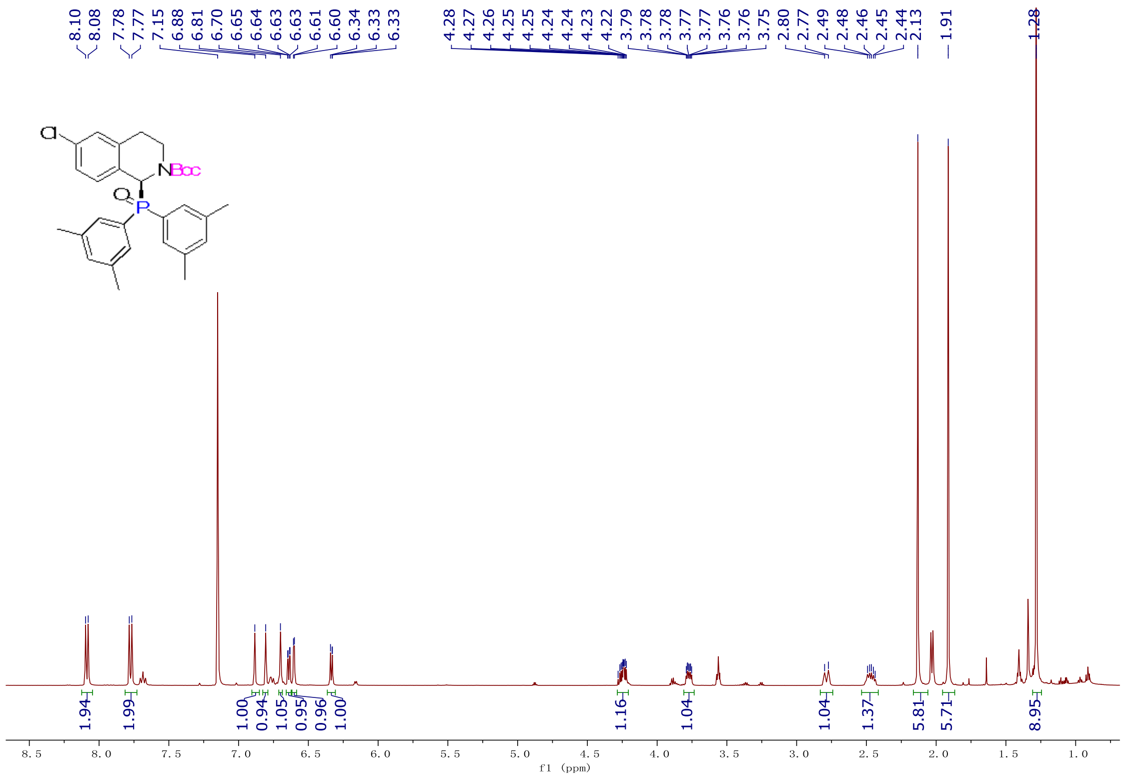

^{1}H NMR of compound **4ika**

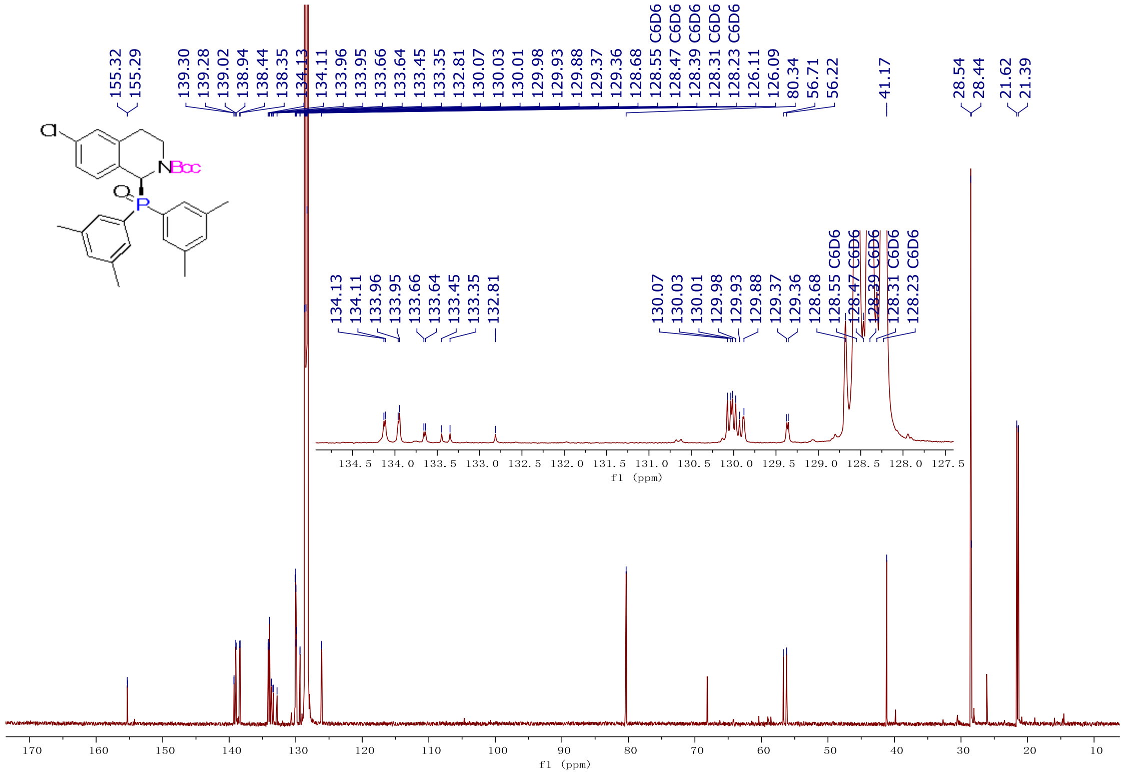

^{13}C NMR of compound **4ika**

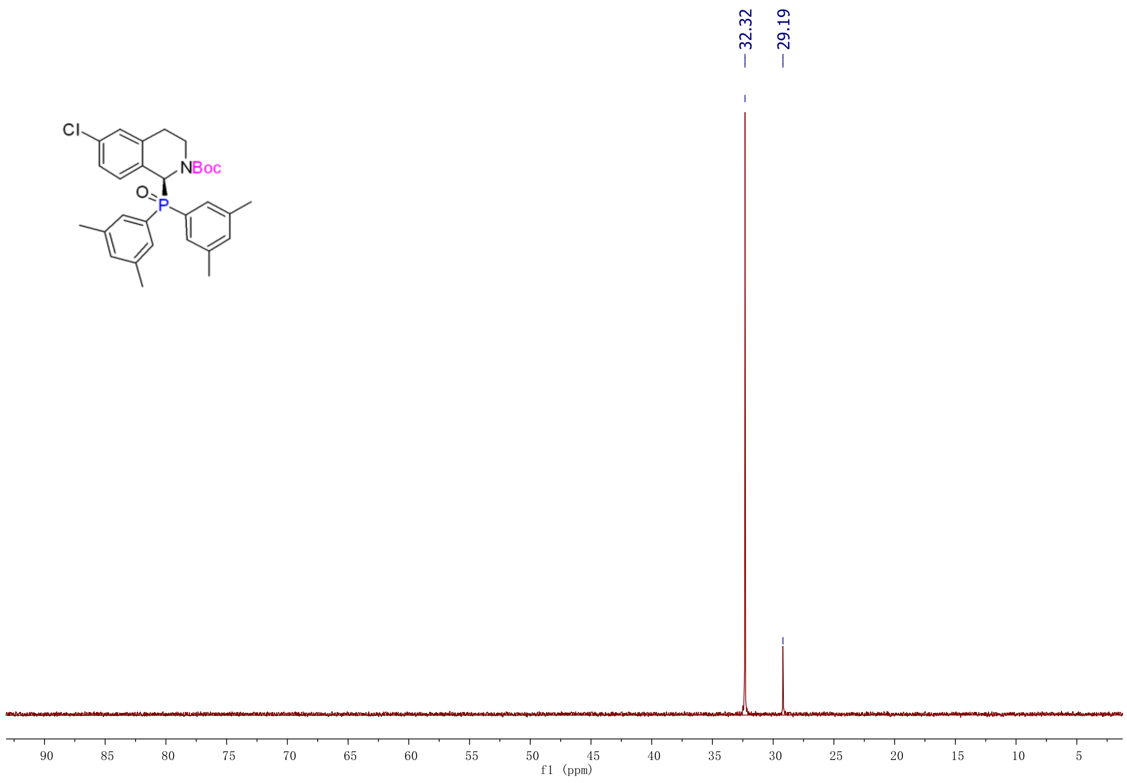

^{31}P NMR of compound **4ika**

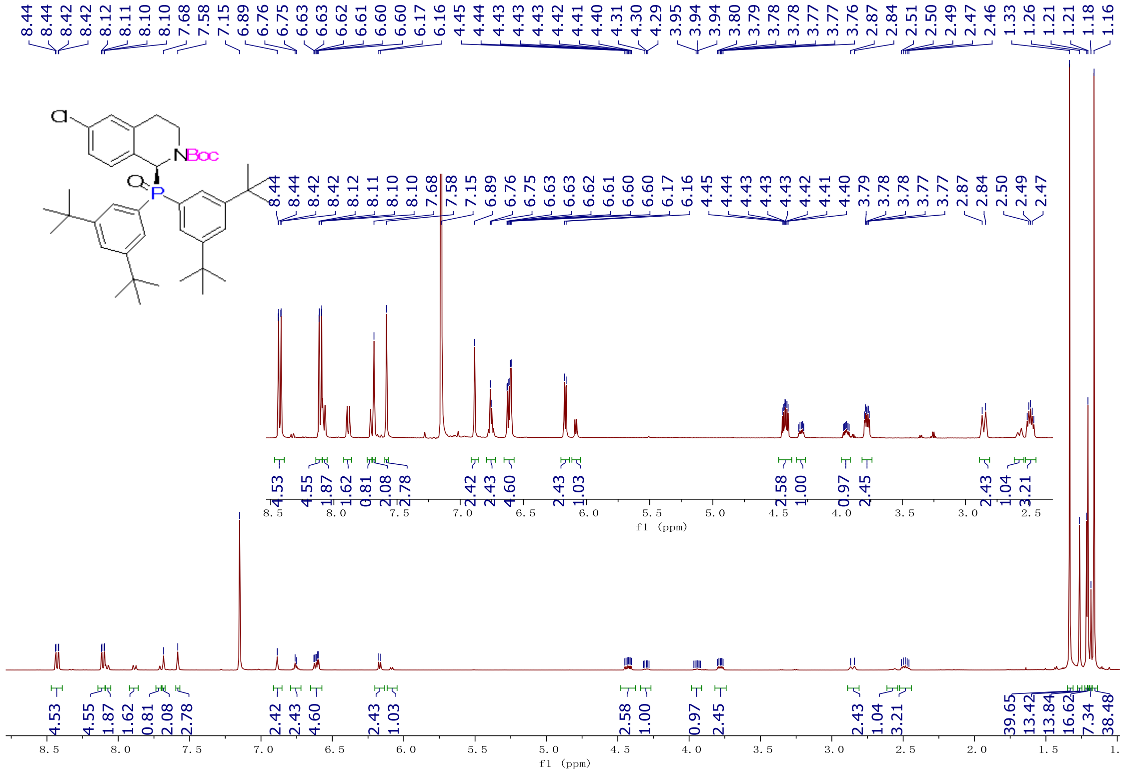

^{1}H NMR of compound **4ika**

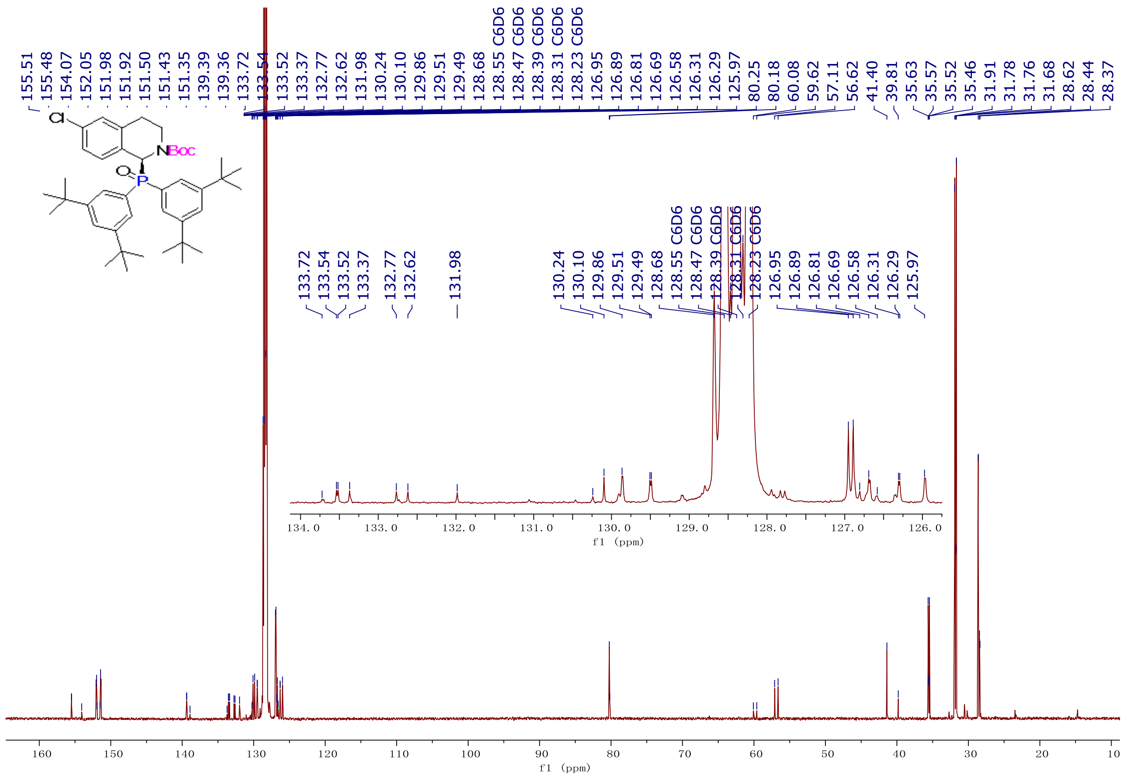

^{13}C NMR of compound **4ika**

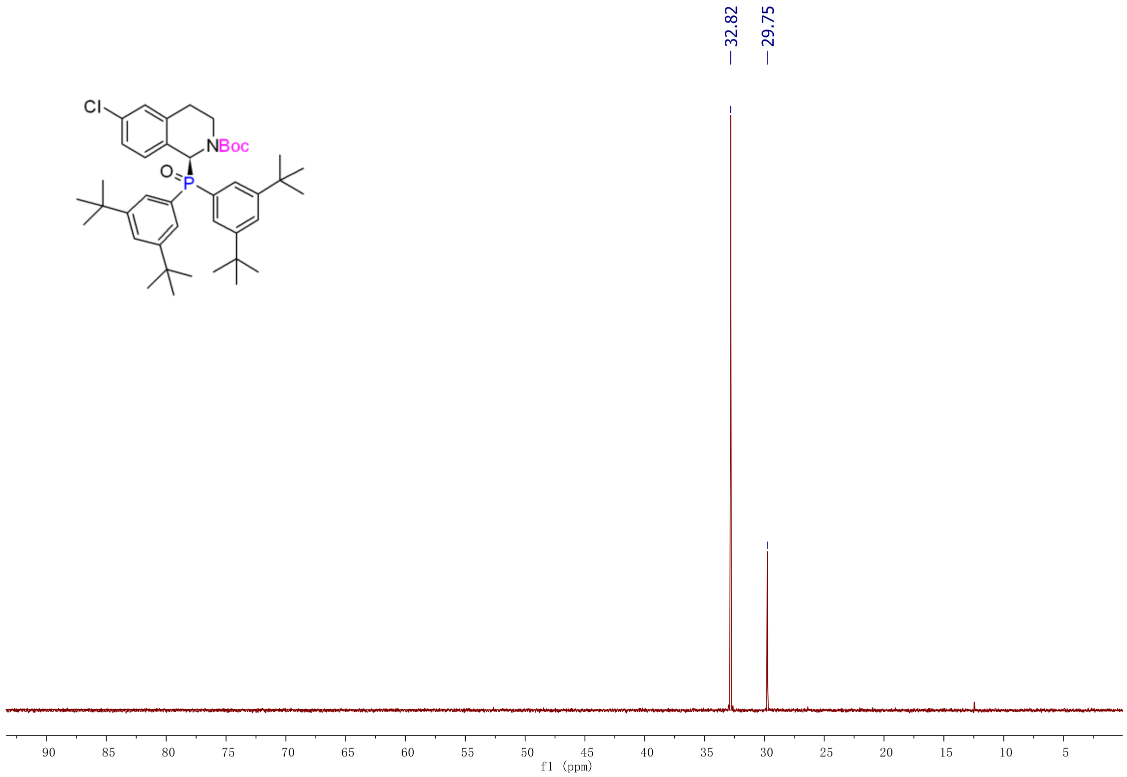

^{31}P NMR of compound **4ika**

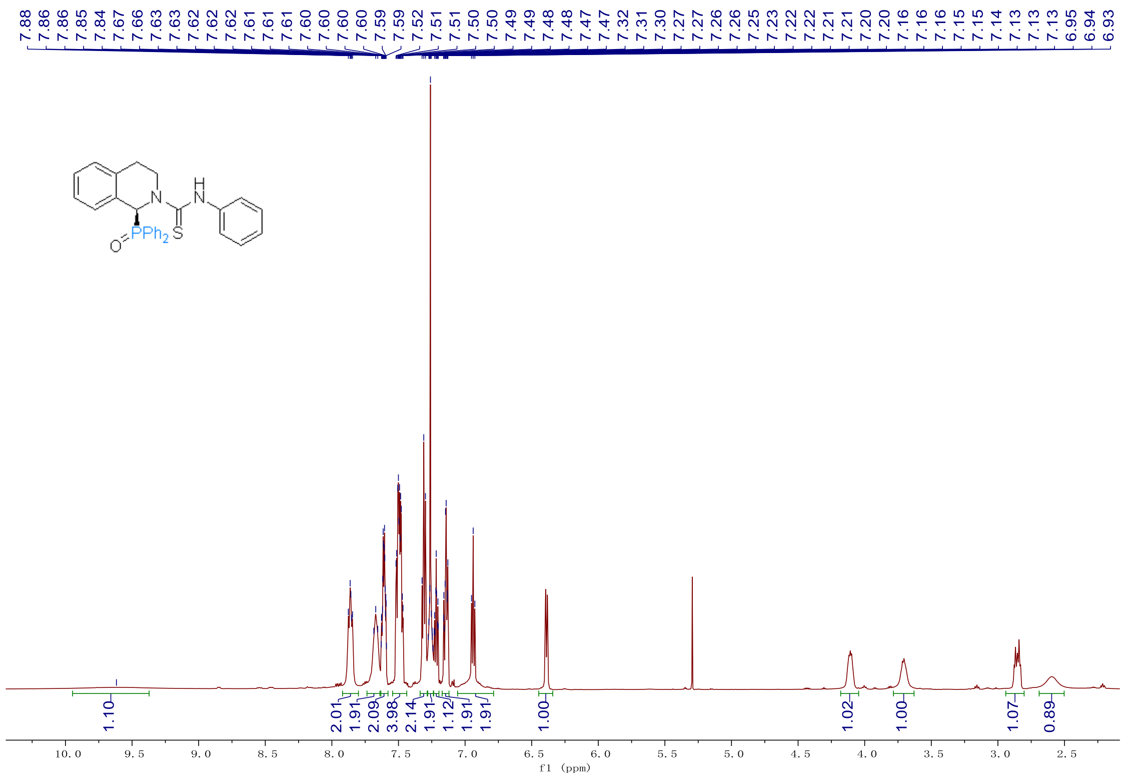

^{1}H NMR of compound **6aaa**

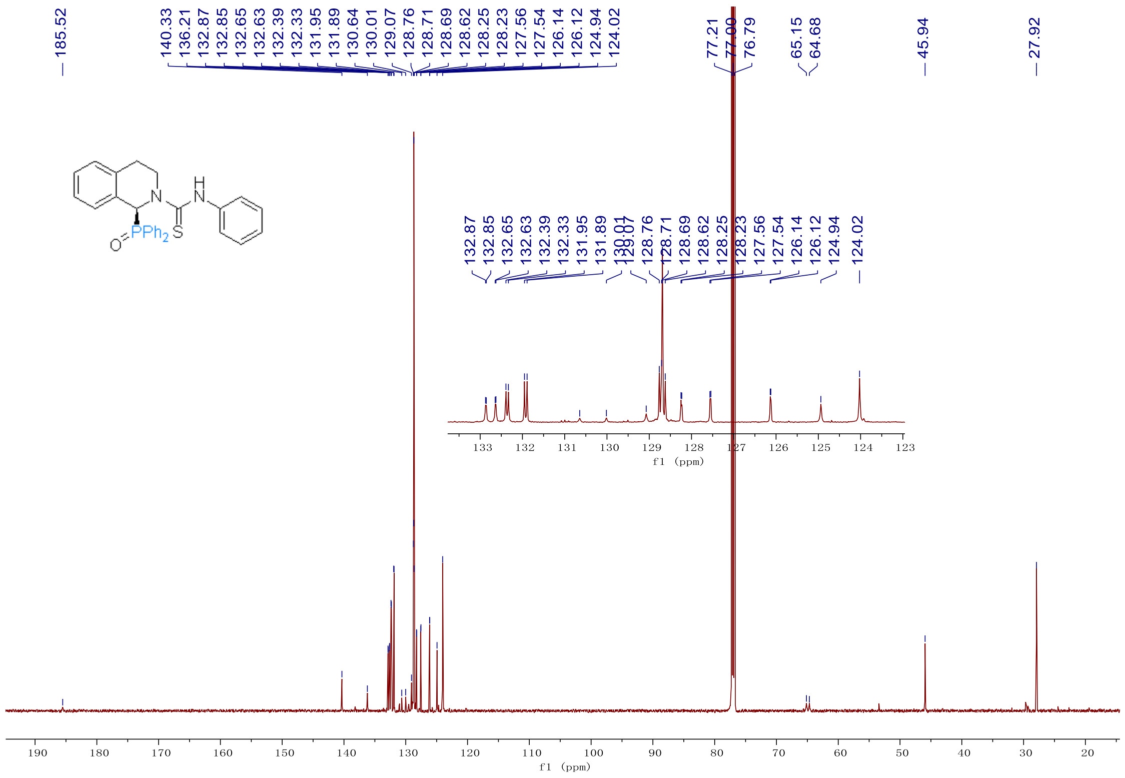

^{13}C NMR of compound **6aaa**

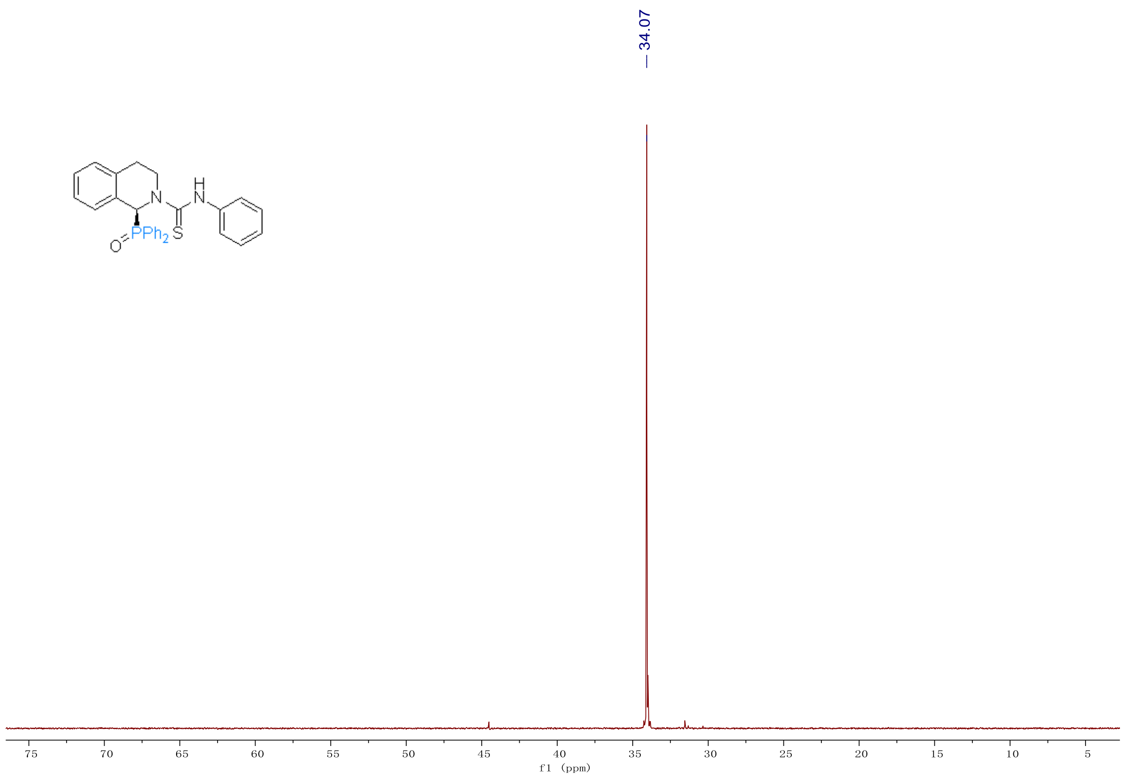

^{31}P NMR of compound **6aaa**

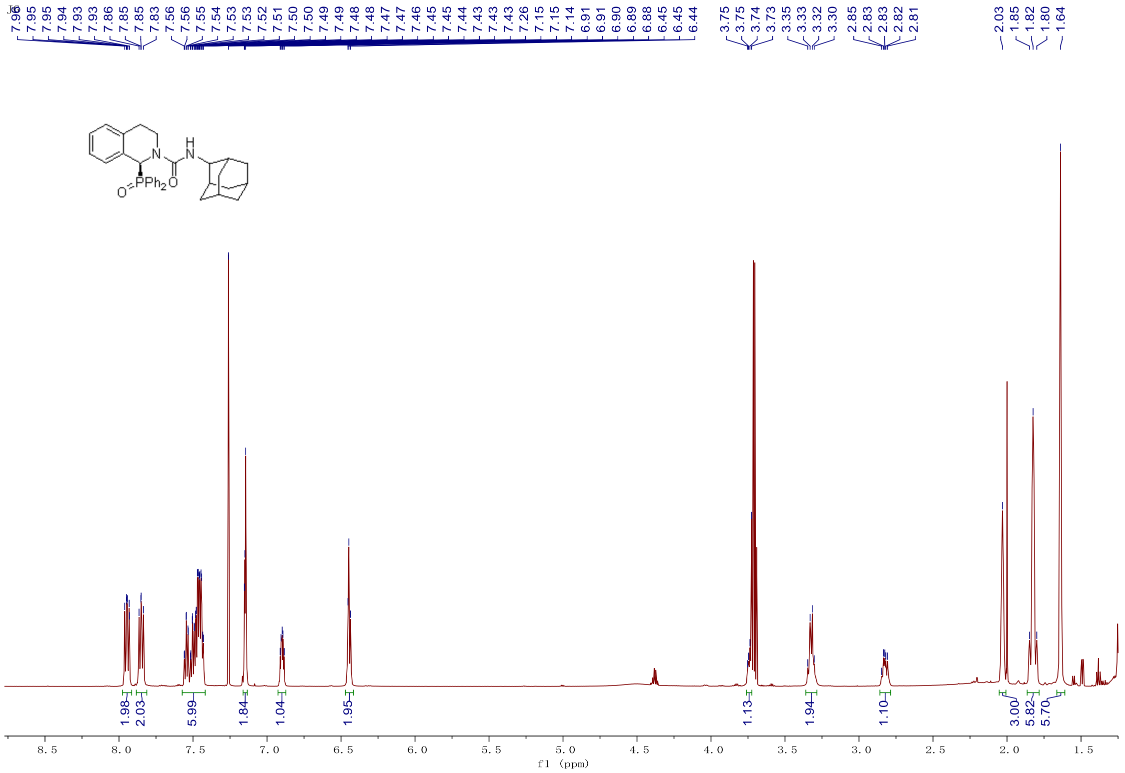

^{1}H NMR of compound **6aab**

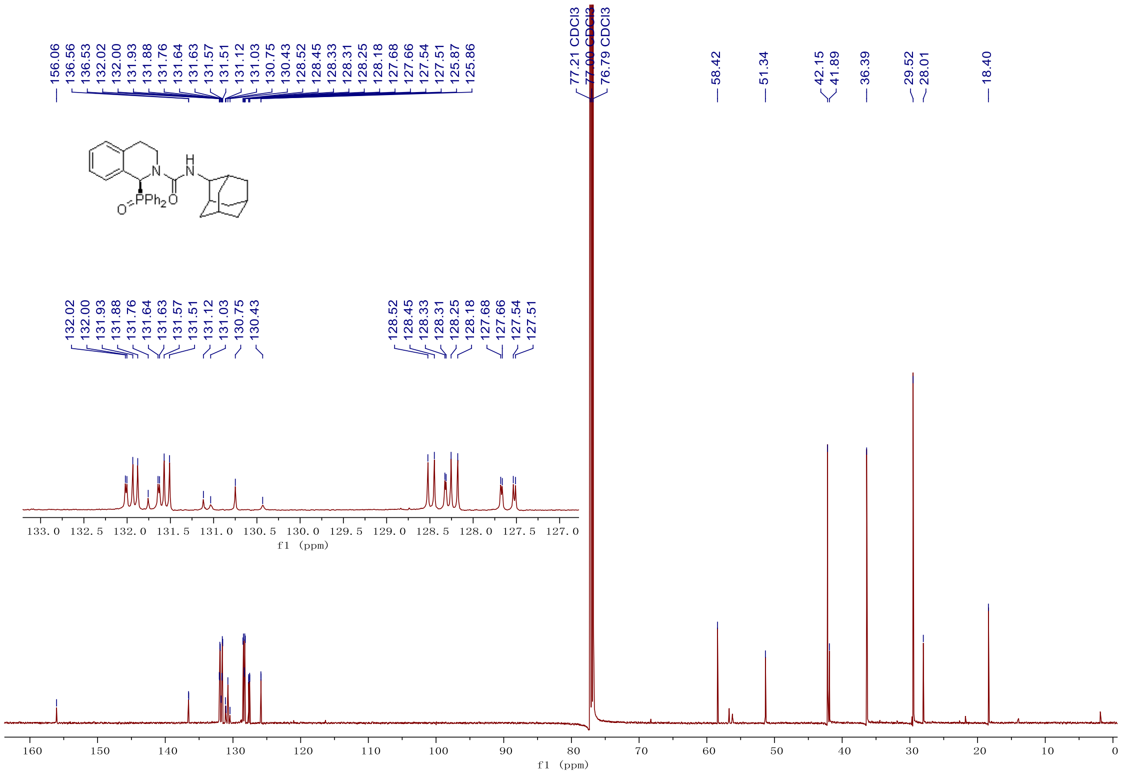

^{13}C NMR of compound **6aab**

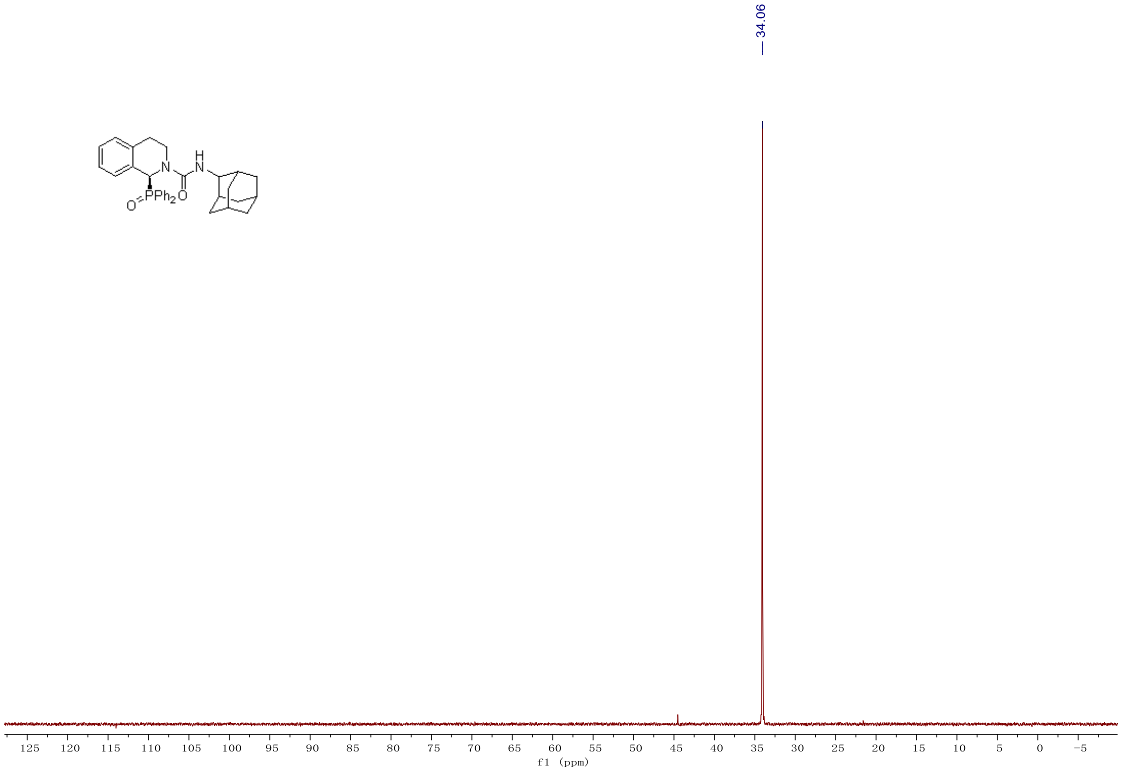

^{31}P NMR of compound **6aab**

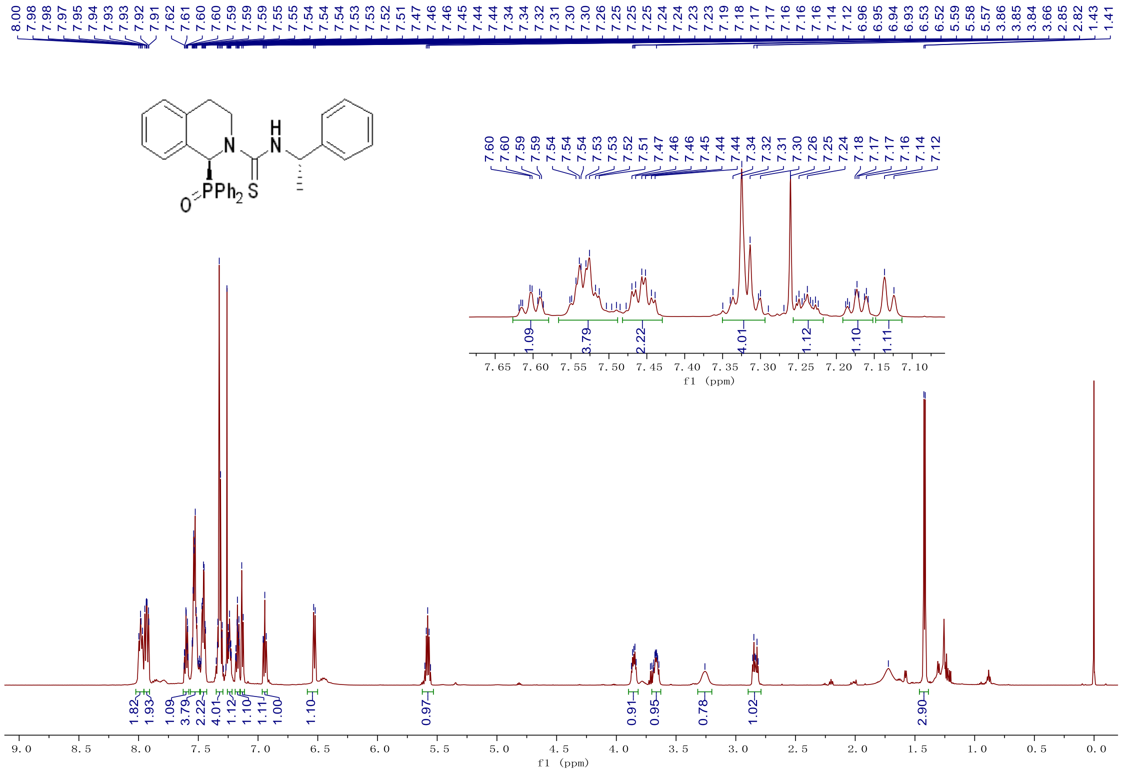

^{1}H NMR of compound **6aac**

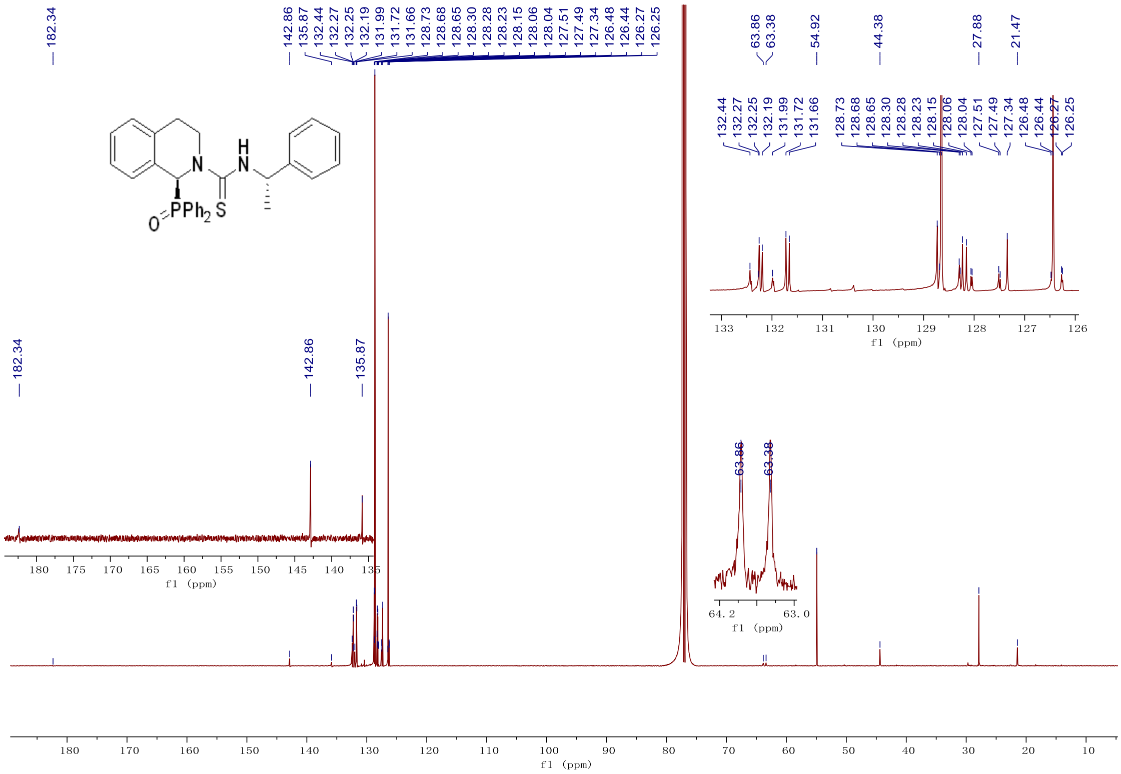

^{13}C NMR of compound **6aac**

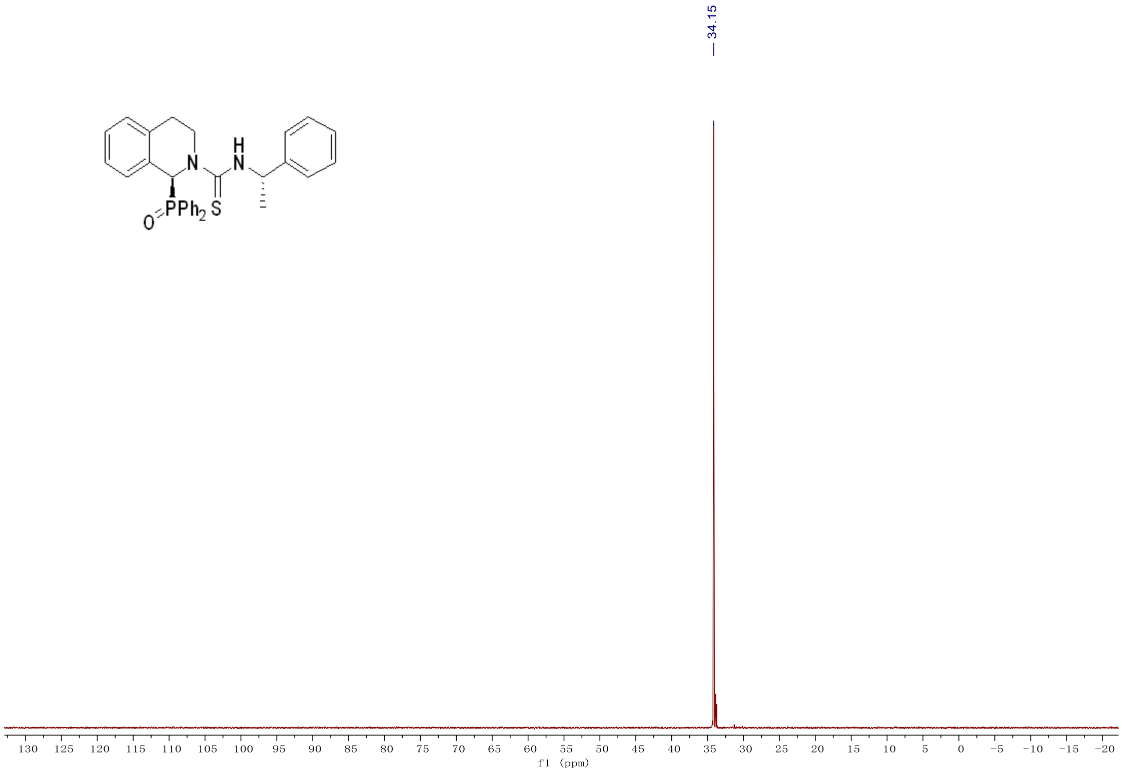

^{31}P NMR of compound **6aac**

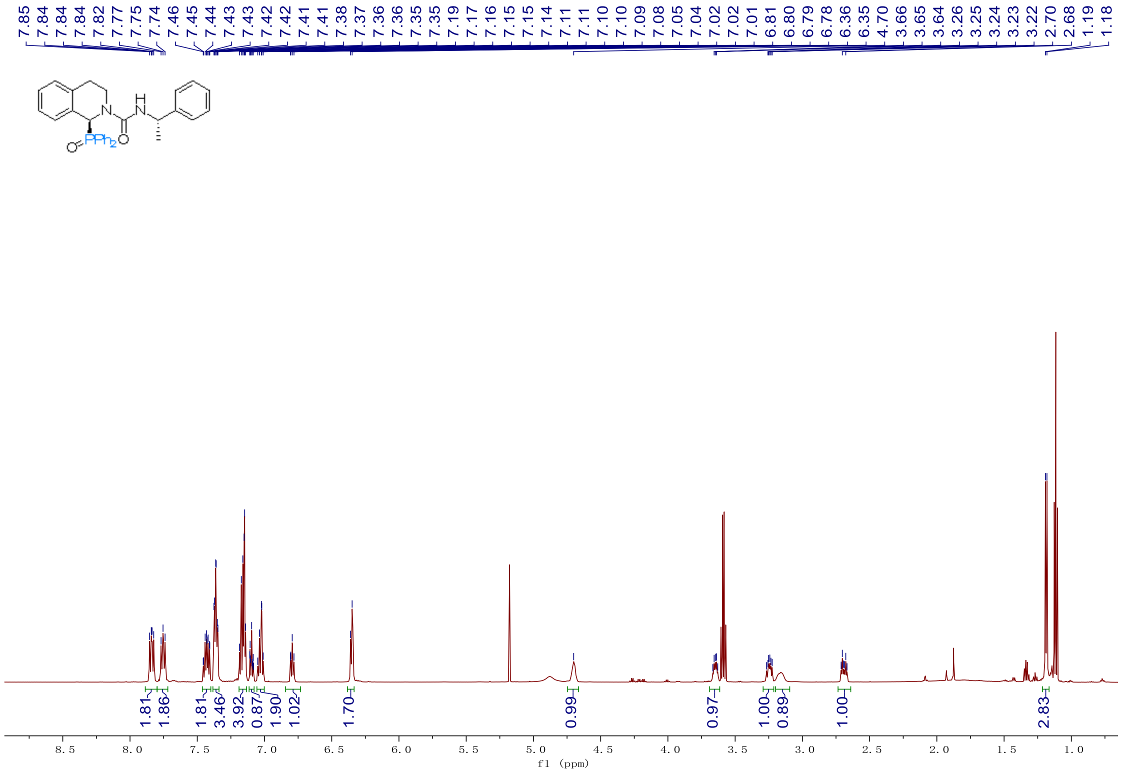

^{1}H NMR of compound **6aad**

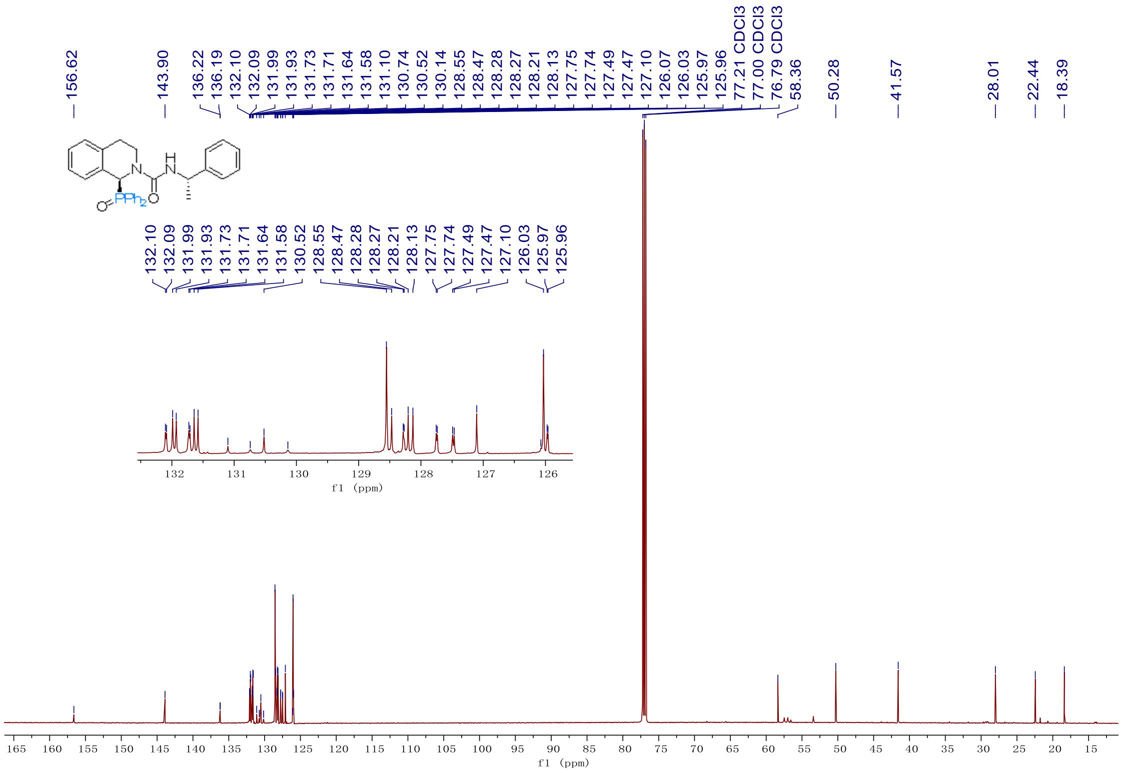

^{13}C NMR of compound **6aad**

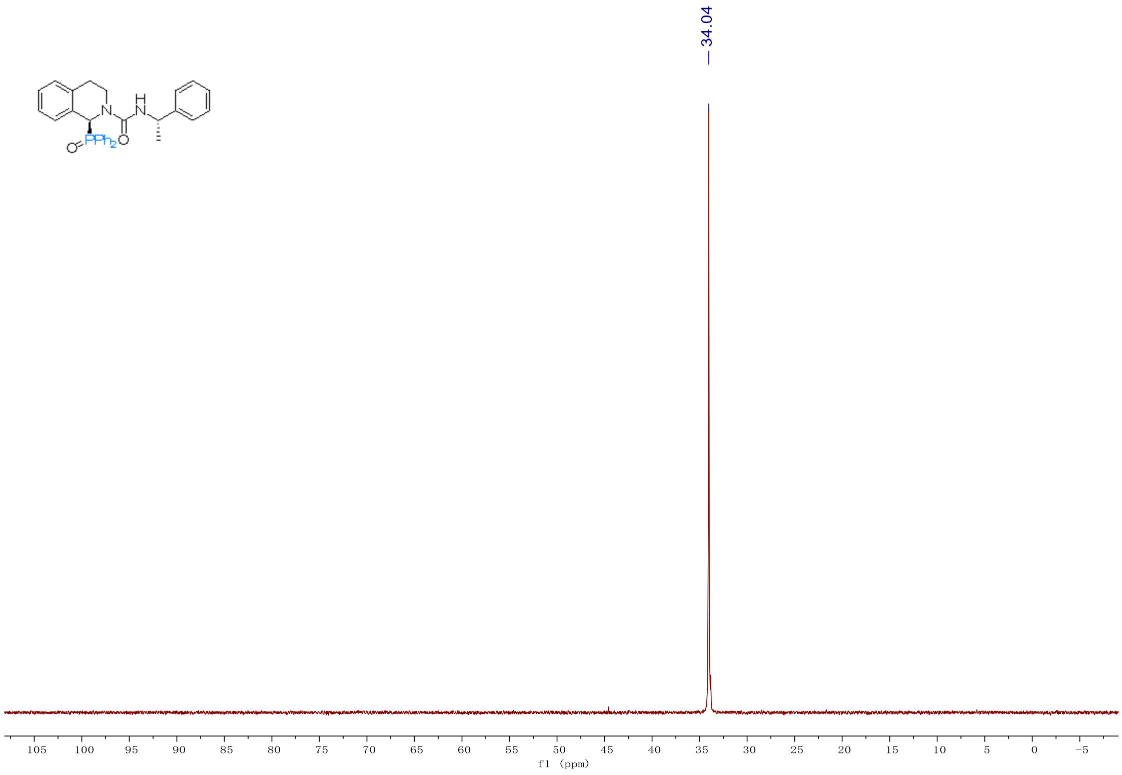

^{31}P NMR of compound **6aad**

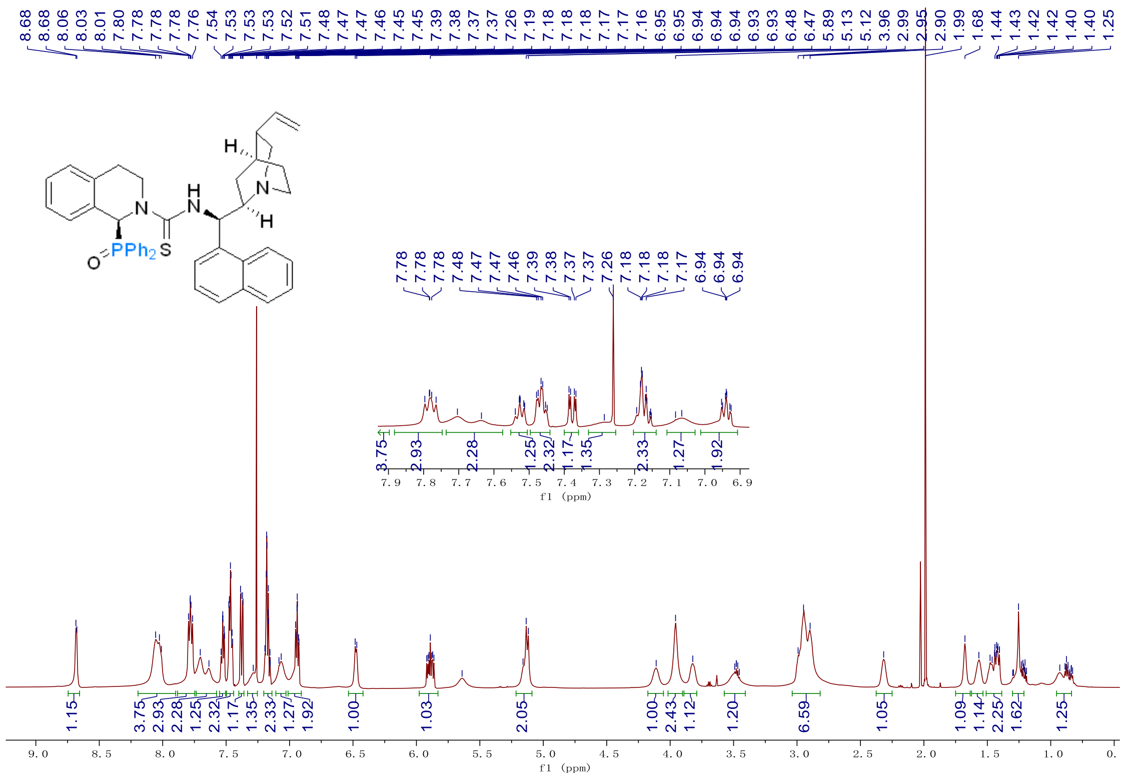

^{1}H NMR of compound **6aae**

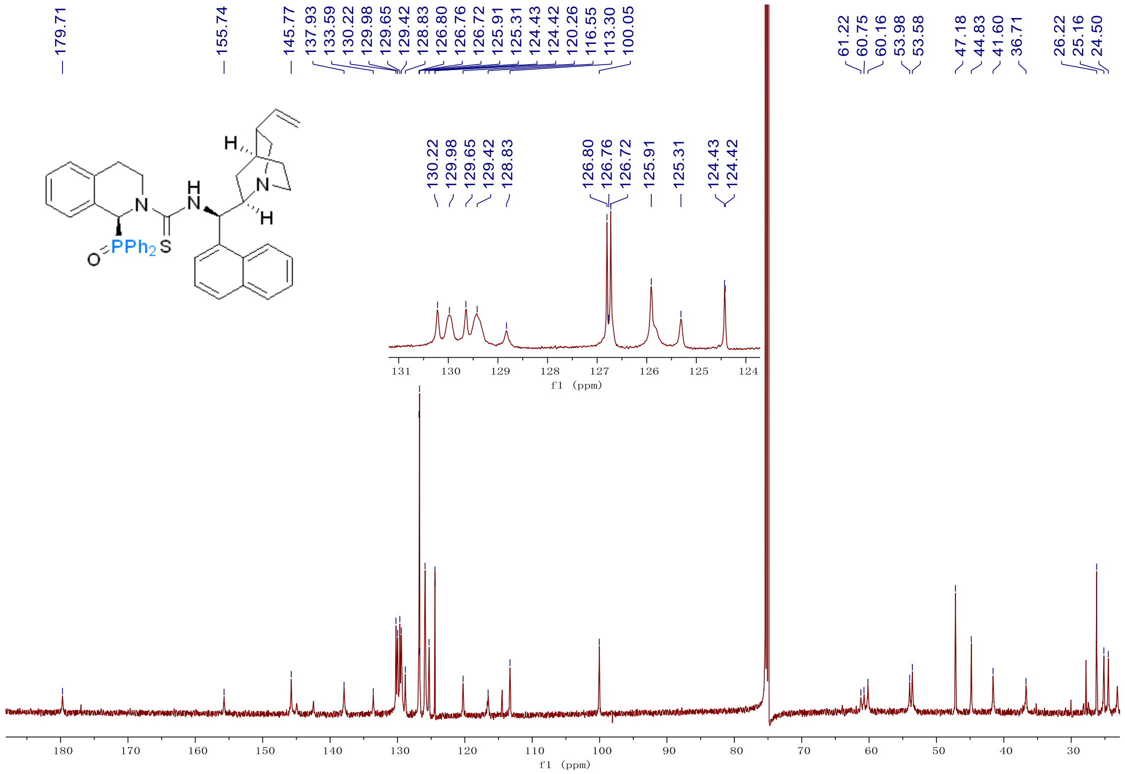

^{13}C NMR of compound **6aad**

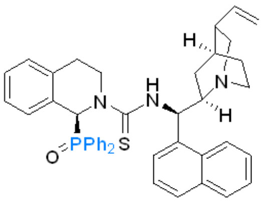

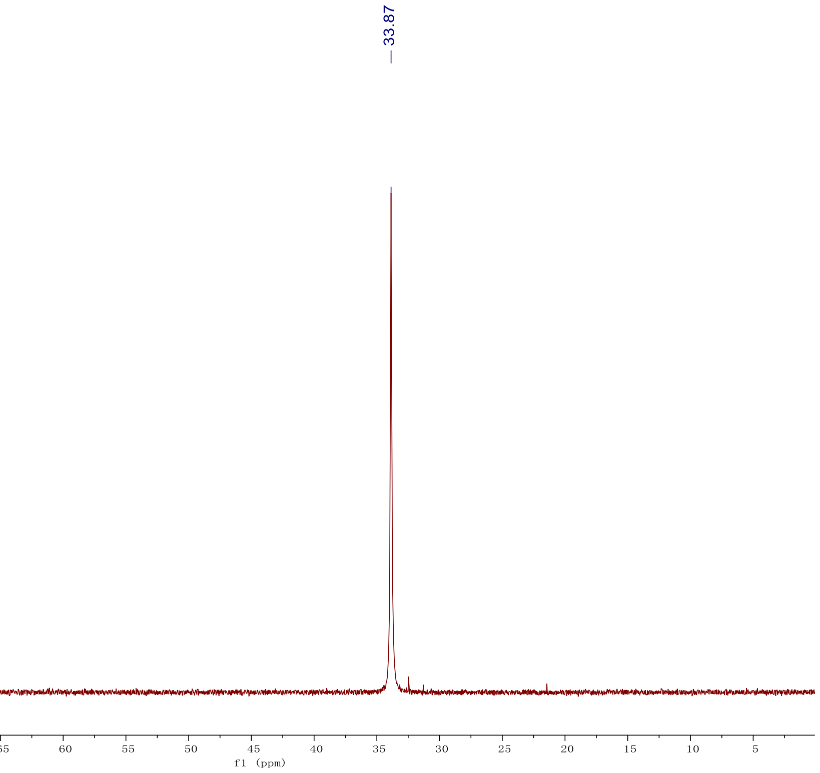

^{31}P NMR of compound **6aad**
