# Supplementary material for: Chiral phosphoric acid-catalyzed enantioselective phosphinylation of 3,4-dihydroisoquinolines with diarylphosphine oxides
Source: Commun Chem. 2023 Feb 9;6:26. doi: 10.1038/s42004-023-00826-4 (PMC9911717; doi:10.1038/s42004-023-00826-4)
Supplement: Supplementary file 5 — Supplementary data 2 [file 42004_2023_826_MOESM5_ESM.pdf]

Supplementary Data 2.

HPLC chromatogram

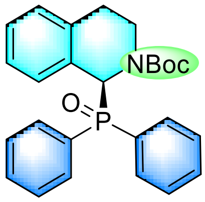

4aaa
99% yield, 91% ee

Chiralpak OD-RH column, MeCN/H_2O= 60/40, flow rate 1.0 mL/min

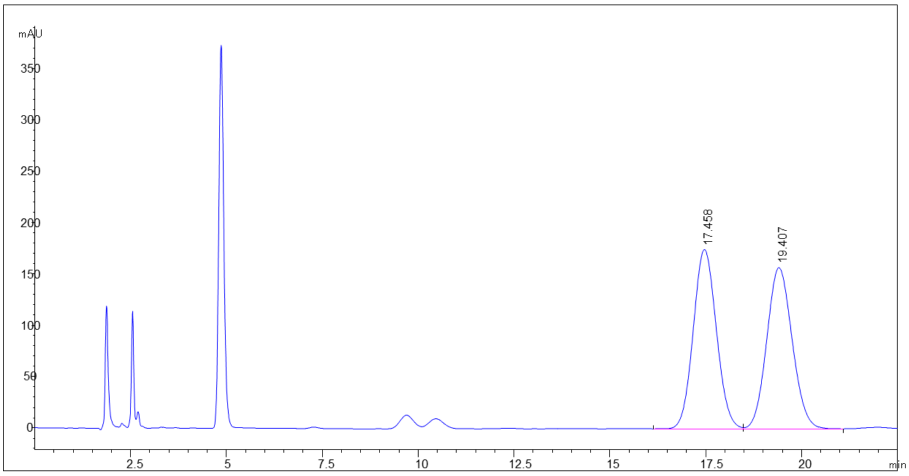

Peak	Retention time	Area	% Area
1	17.458	7399.4	50.228
2	19.407	7332.2	49.772

HPLC for racemic compound **4aaa**

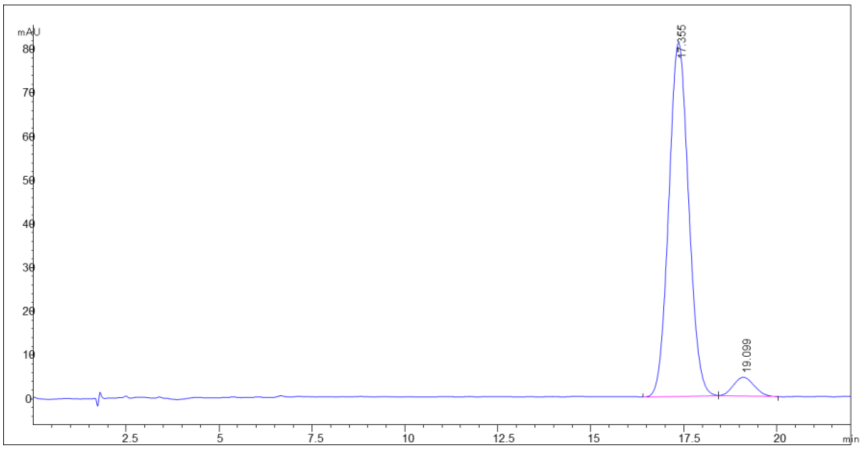

Peak	Retention time	Area	% Area
1	17.355	3011.1	95.35
2	19.099	146.8	4.65

HPLC for pure enantioenriched compound **4aaa**

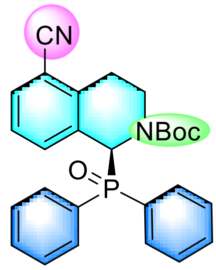

4baa
86% yield, 92% ee

Chiralpak OJ-RH column, MeCN/H_2O= 65/35, flow rate 1.0 mL/min

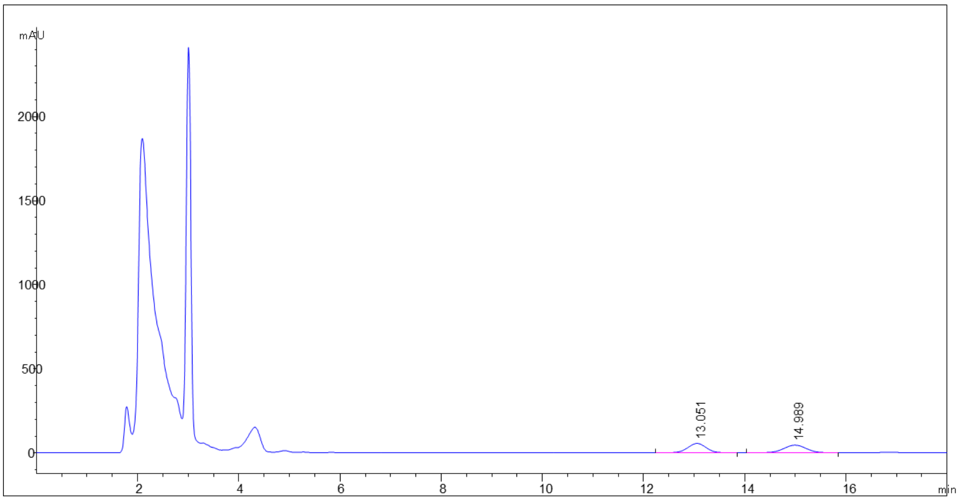

Peak	Retention time	Area	% Area
1	13.050	1408.4	49.832
2	14.989	1417.9	50.168

HPLC for racemic compound **4baa**

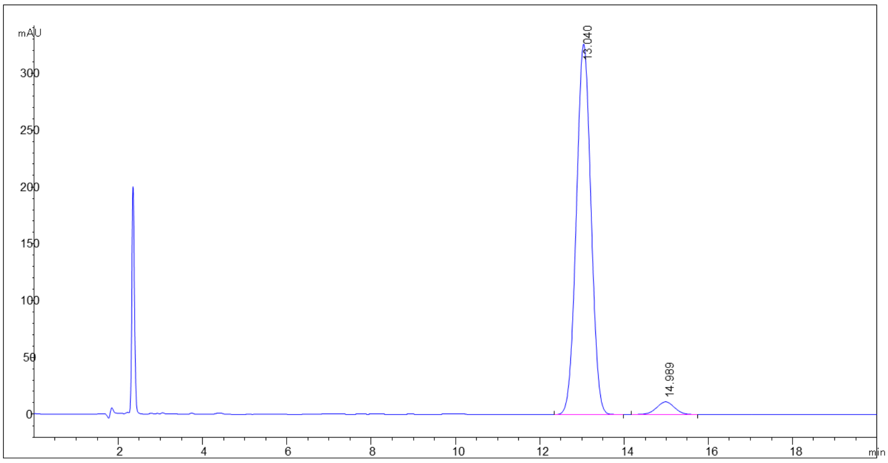

Peak	Retention time	Area	% Area
1	13.040	7888.8	95.915
2	14.989	336	4.085

HPLC for pure enantioenriched compound **4baa**

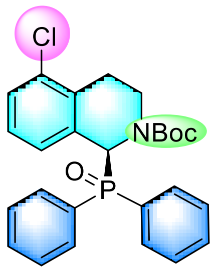

4caa
83% yield, 95% ee

Chiralpak OJ-RH column, MeCN/H_2O= 55/45, flow rate 1.0 mL/min

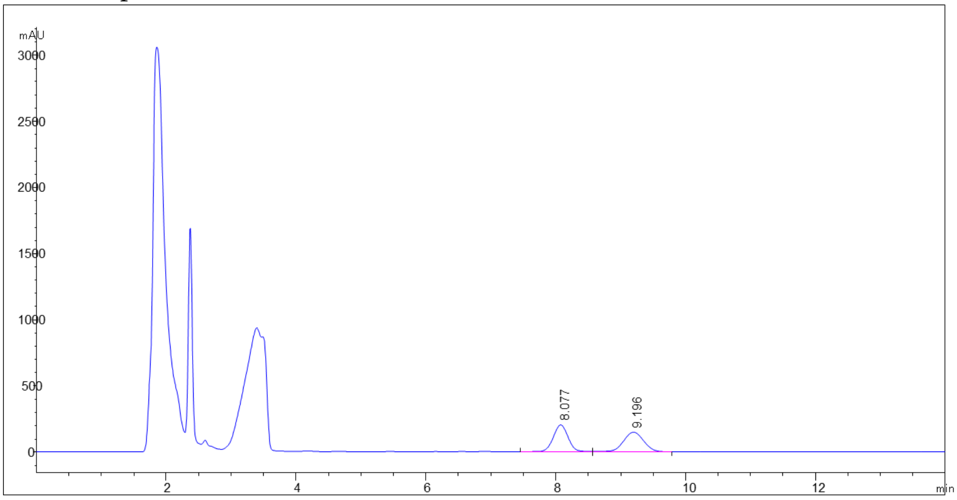

Peak	Retention time	Area	% Area
1	8.077	3245.6	50.260
2	9.196	3212	49.740

HPLC for racemic compound **4caa**

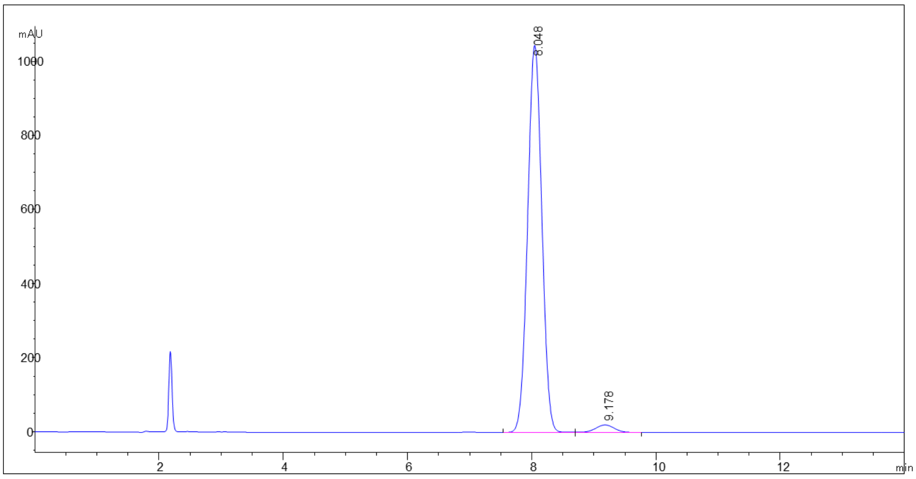

Peak	Retention time	Area	% Area
1	8.048	16570.2	97.556
2	9.178	415.1	2.444

HPLC for pure enantioenriched compound **4caa**

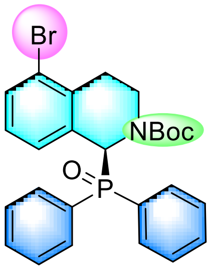

4daa
91% yield, 97% ee

Chiralpak OJ-RH column, MeCN/H_2O= 65/35, flow rate 1.0 mL/min

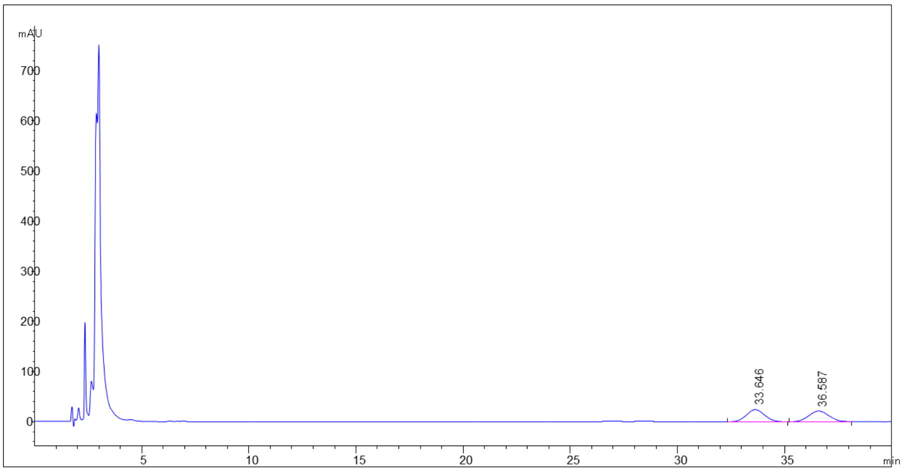

Peak	Retention time	Area	% Area
1	33.646	1445.3	49.975
2	36.587	1446.7	50.025

HPLC for racemic compound **4daa**

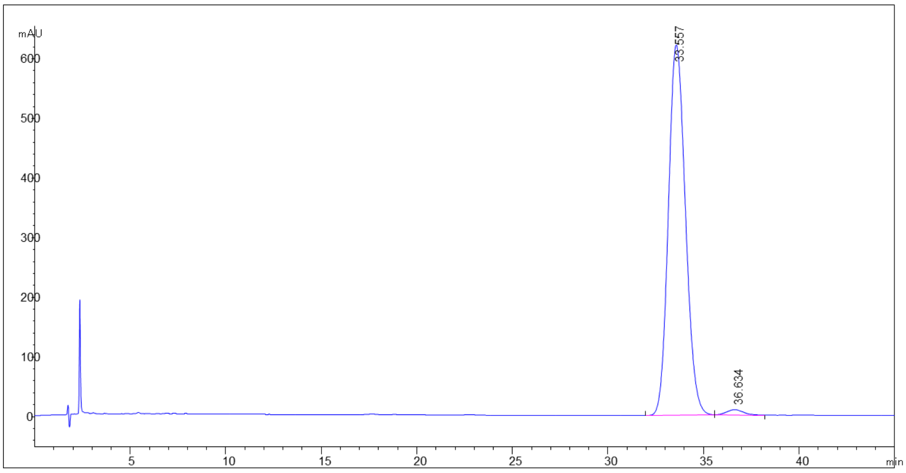

Peak	Retention time	Area	% Area
1	33.557	38390.7	98.566
2	36.634	558.6	1.434

HPLC for pure enantioenriched compound **4daa**

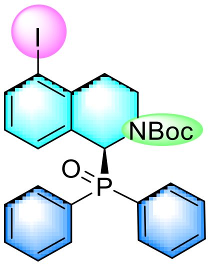

4eaa
83% yield, 95% ee

Chiralpak AS-RH column, MeCN/H_2O= 50/50, flow rate 1.0 mL/min

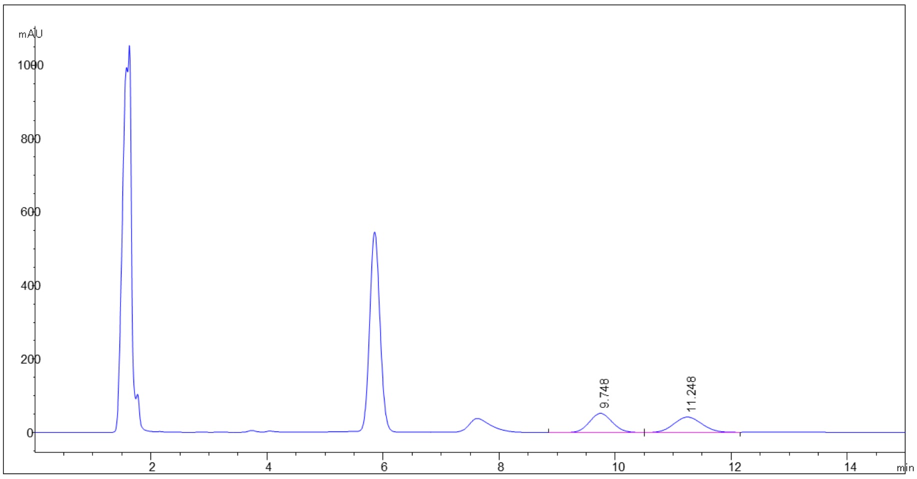

Peak	Retention time	Area	% Area
1	9.748	1465.3	50.459
2	11.248	1438.7	49.541

HPLC for racemic compound **4eaa**

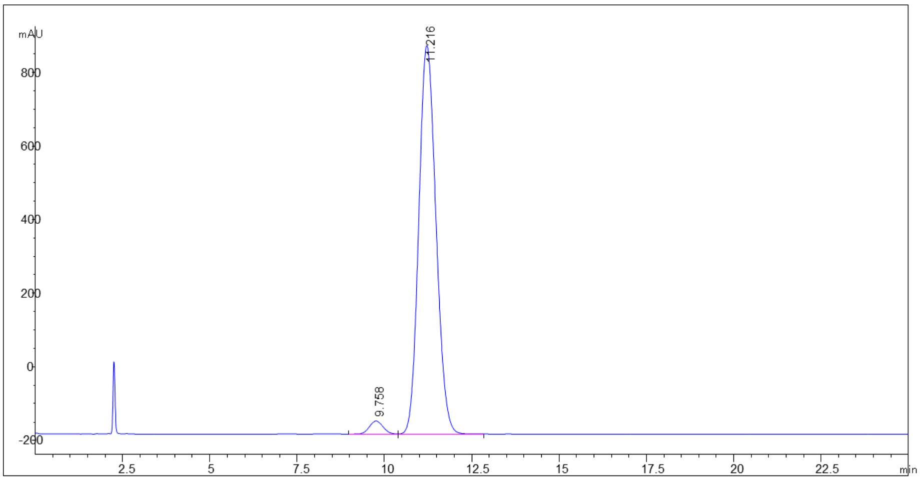

Peak	Retention time	Area	% Area
1	9.758	999.2	2.683
2	11.216	36243.5	97.317

HPLC for pure enantioenriched compound **4eaa**

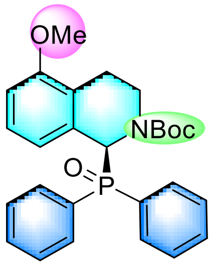

4faa
92% yield, 89% ee

Chiralpak AS-RH column, MeCN/H_2O= 50/50, flow rate 1.0 mL/min

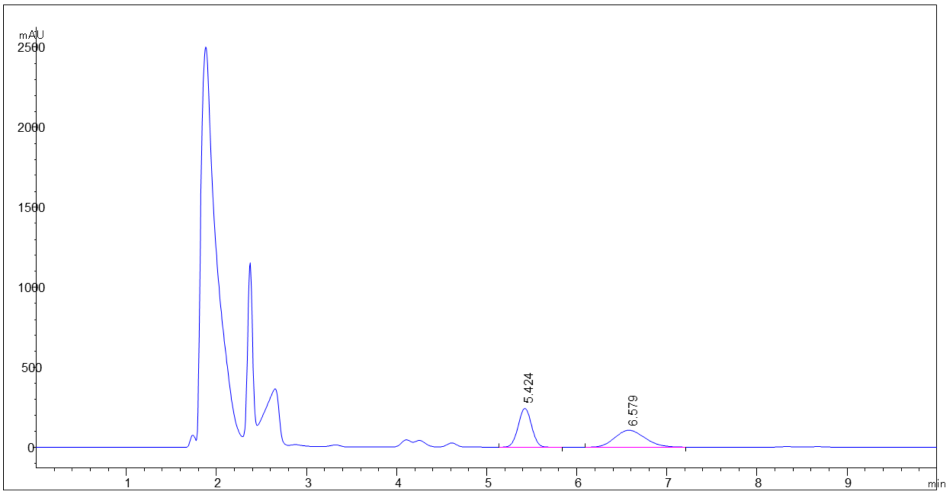

Peak	Retention time	Area	% Area
1	5.424	2543.6	50.294
2	6.579	2513.9	49.706

HPLC for racemic compound **4faa**

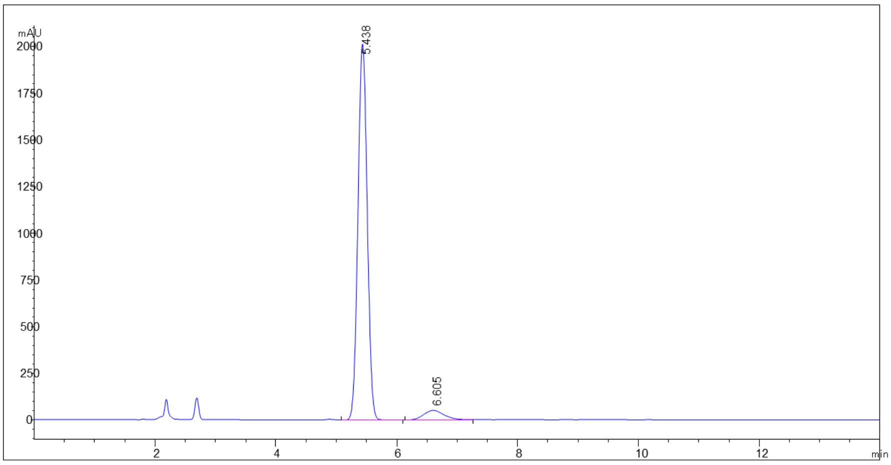

Peak	Retention time	Area	% Area
1	5.438	21480.3	94.655
2	6.605	1213.1	5.345

HPLC for pure enantioenriched compound **4faa**

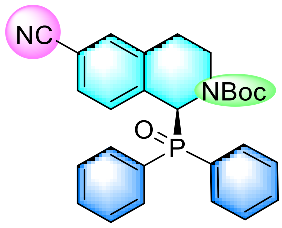

4gaa

90% yield, 89% ee

Chiralpak OD-RH column, MeCN/H_2O= 68/32, flow rate 1.0 mL/min

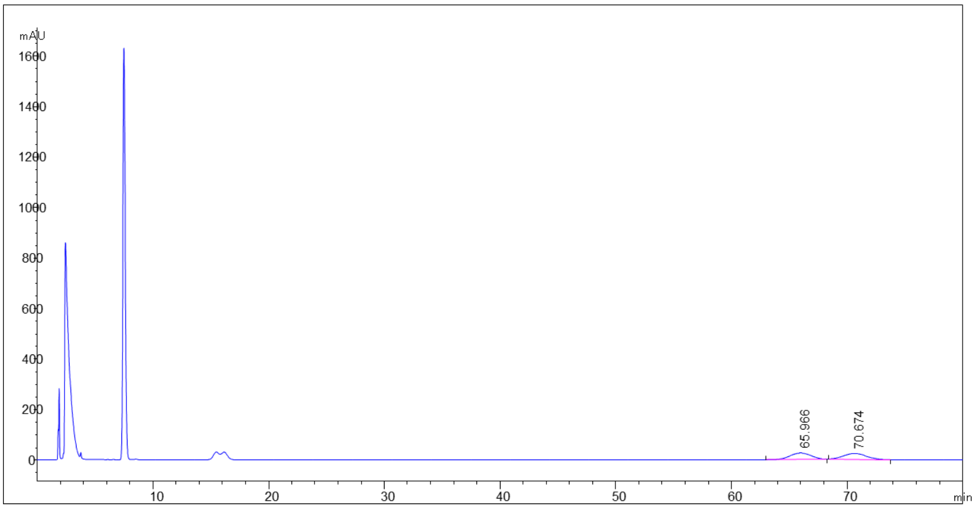

Peak	Retention time	Area	% Area
1	65.966	3356.2	50.324
2	70.674	3313	49.676

HPLC for racemic compound **4gaa**

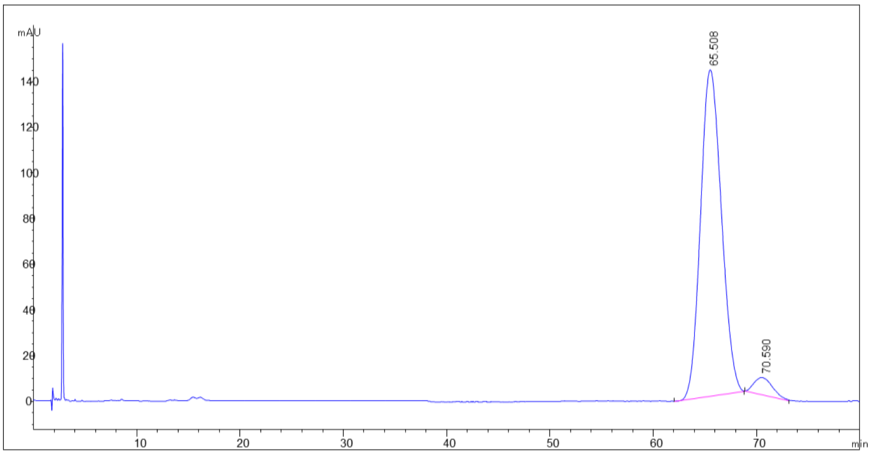

Peak	Retention time	Area	% Area
1	65.508	20089	95.498
2	70.590	947.1	4.502

HPLC for pure enantioenriched compound **4gaa**

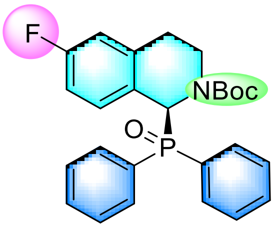

4haa

93% yield, 88% ee

Chiralpak OJ-RH column, MeCN/H_2O= 50/50, flow rate 1.0 mL/min

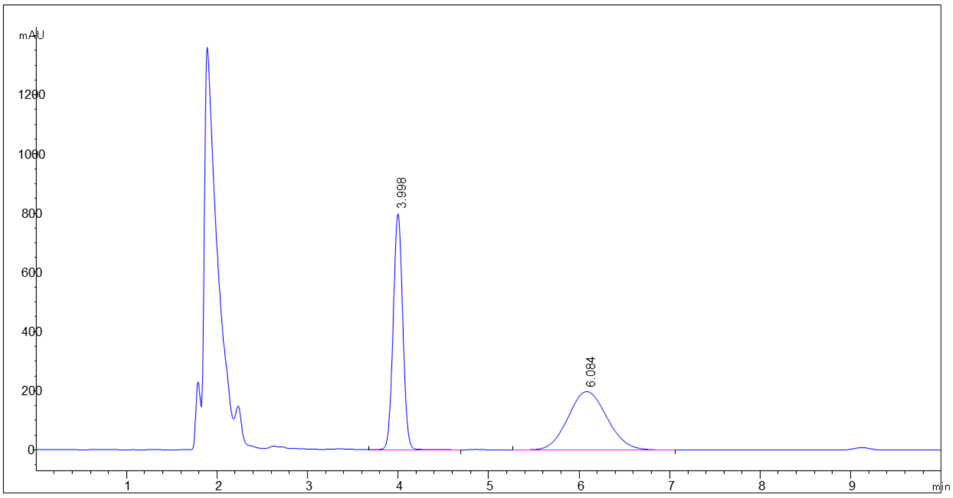

Peak	Retention time	Area	% Area
1	3.998	5871.5	49.727
2	6.084	5936.1	50.273

HPLC for racemic compound **4haa**

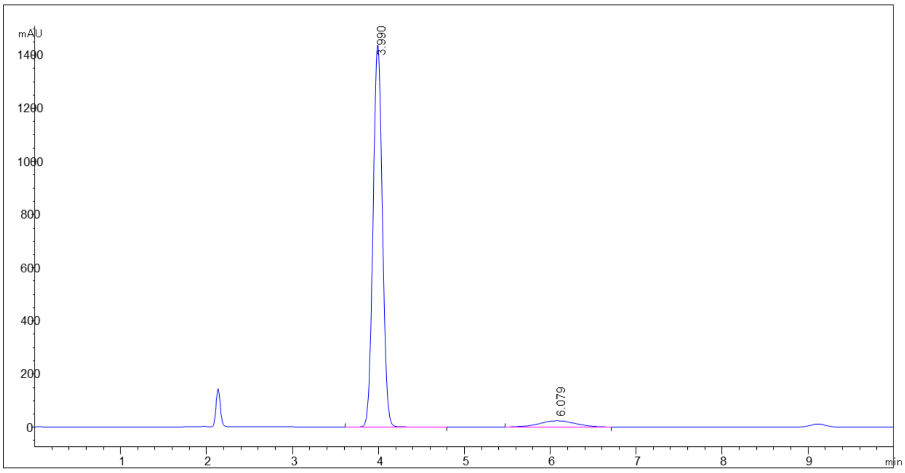

Peak	Retention time	Area	% Area
1	3.990	10861	93.942
2	6.079	700.4	6.058

HPLC for pure enantioenriched compound **4haa**

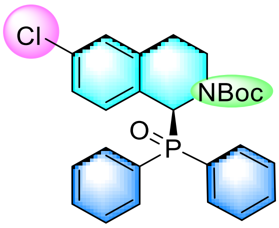

4iaa
91% yield, 93% ee

Chiralpak OJ-RH column, MeCN/H_2O= 50/50, flow rate 1.0 mL/min

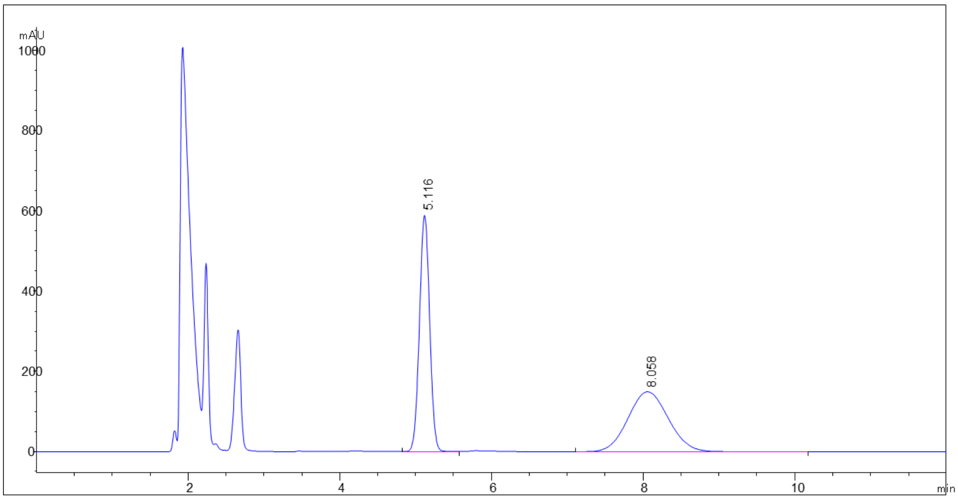

Peak	Retention time	Area	% Area
1	5.116	5525.7	49.819
2	8.058	5565.8	50.181

HPLC for racemic compound **4iaa**

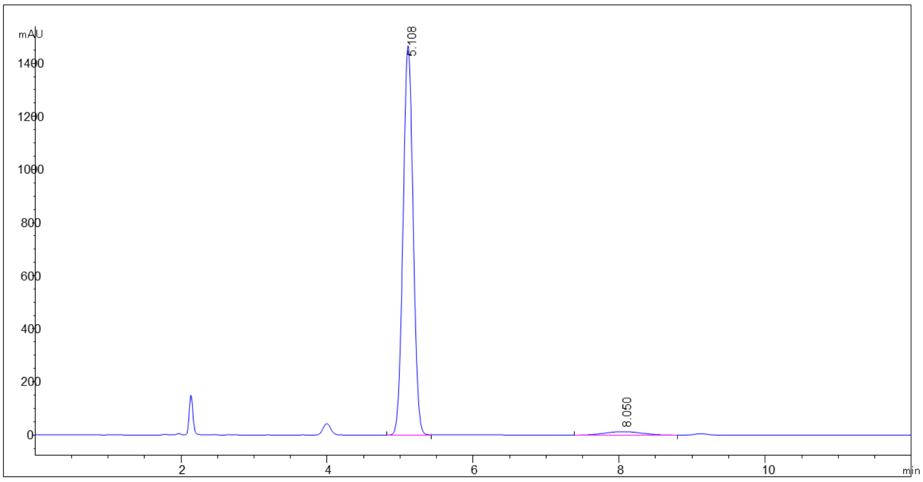

Peak	Retention time	Area	% Area
1	5.108	14023.4	96.391
2	8.050	525.1	3.609

HPLC for pure enantioenriched compound **4iaa**

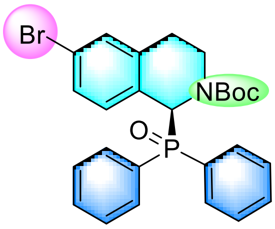

4jaa
93% yield, 93% ee

Chiralpak OJ-RH column, MeCN/H_2O= 55/45, flow rate 1.0 mL/min

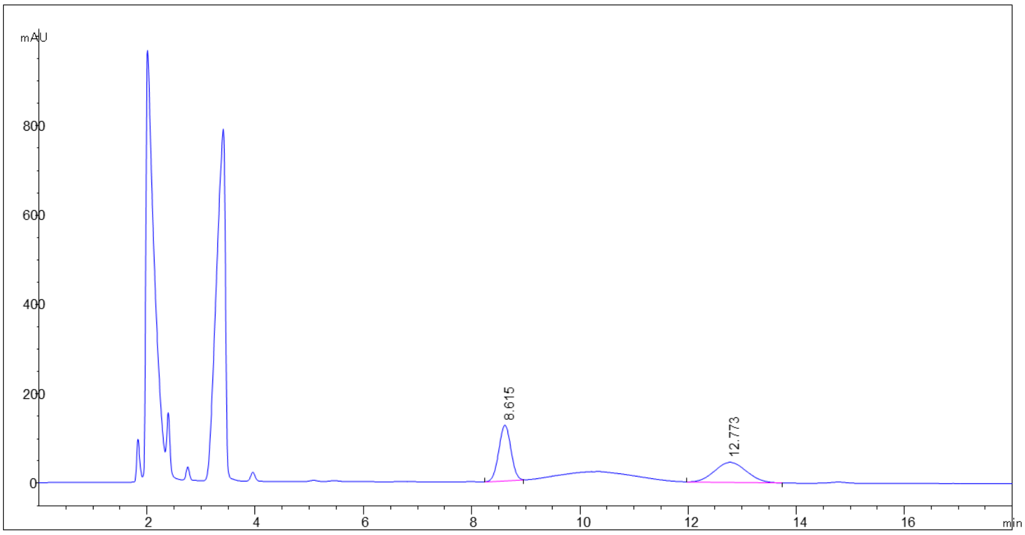

Peak	Retention time	Area	% Area
1	8.615	1962.8	51.130
2	12.773	1876	48.870

HPLC for racemic compound **4jaa**

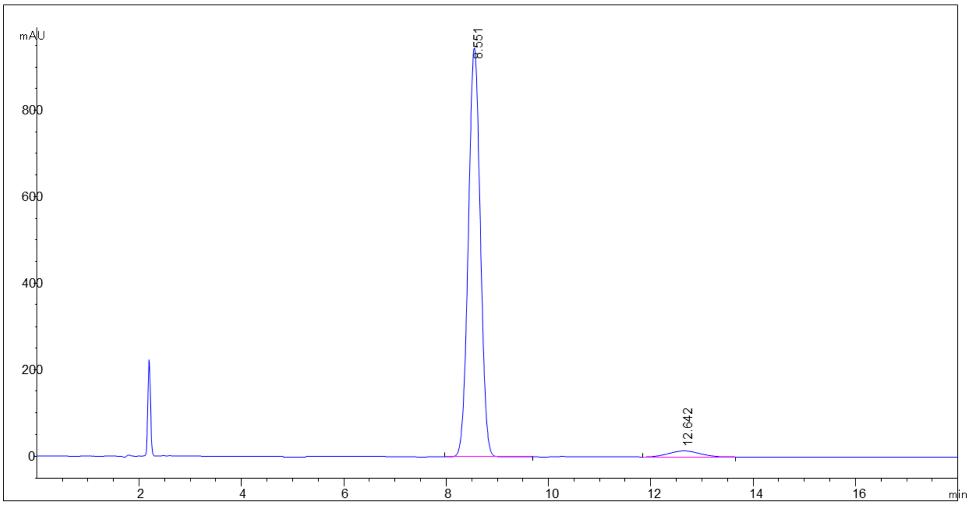

Peak	Retention time	Area	% Area
1	8.551	15352.6	96.254
2	12.642	594.2	3.746

HPLC for pure enantioenriched compound **4jaa**

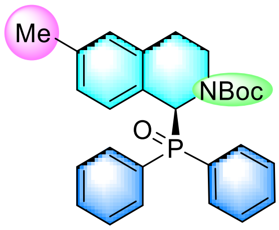

4kaa
99% yield, 79% ee

Chiralpak OJ-RH column, MeCN/H_2O= 50/50, flow rate 1.0 mL/min

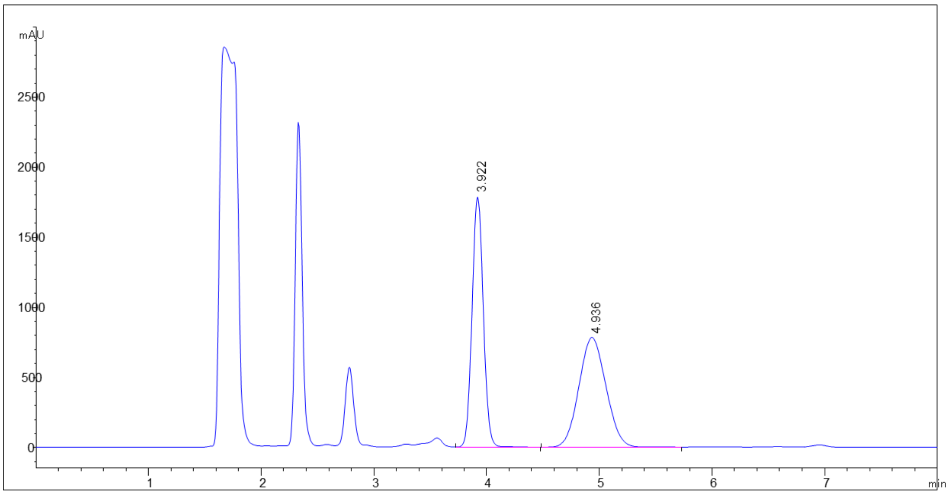

Peak	Retention time	Area	% Area
1	3.922	12405.7	48.708
2	4.936	13063.5	51.292

HPLC for racemic compound **4kaa**

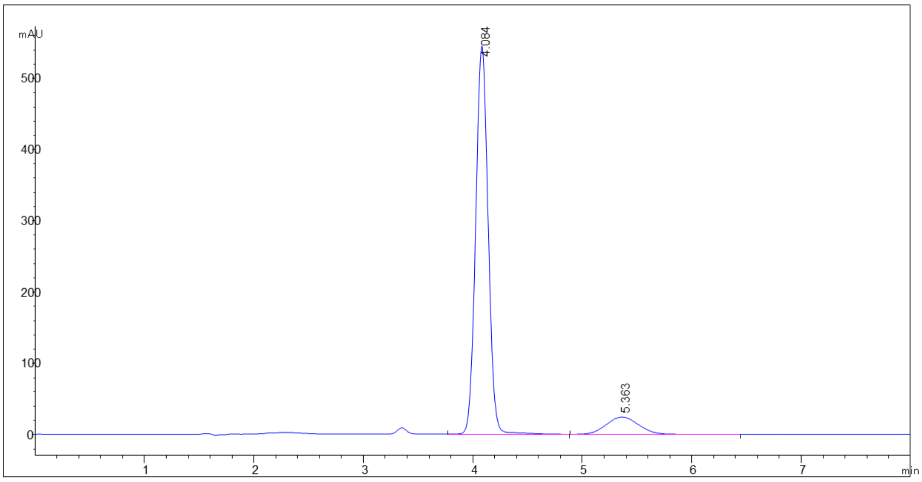

Peak	Retention time	Area	% Area
1	4.084	4302.5	89.534
2	5.363	502.9	10.466

HPLC for pure enantioenriched compound **4kaa**

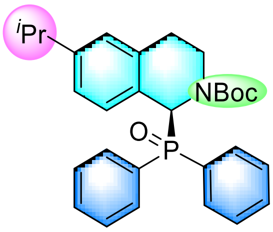

4laa
86% yield, 42% ee

Chiralpak OJ-RH column, MeCN/H_2O= 55/45, flow rate 1.0 mL/min

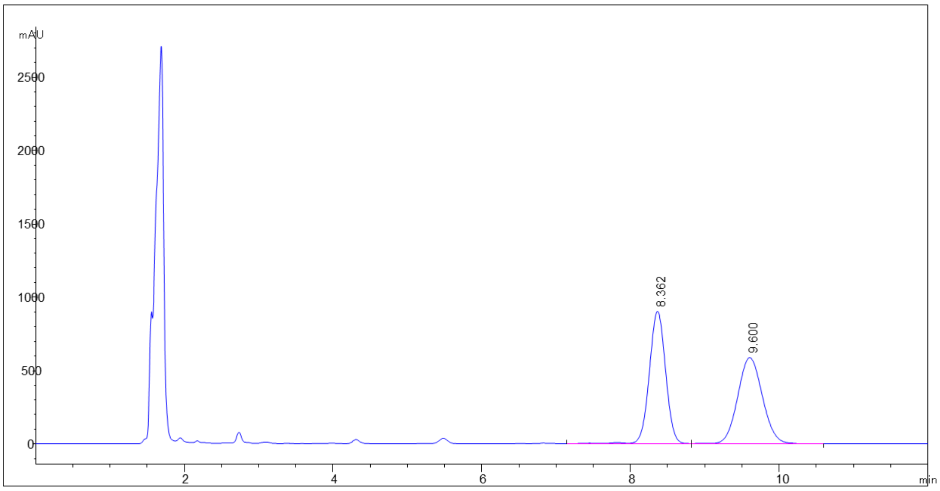

Peak	Retention time	Area	% Area
1	8.362	13706.4	50.382
2	9.600	13498.8	49.618

HPLC for racemic compound **4laa**

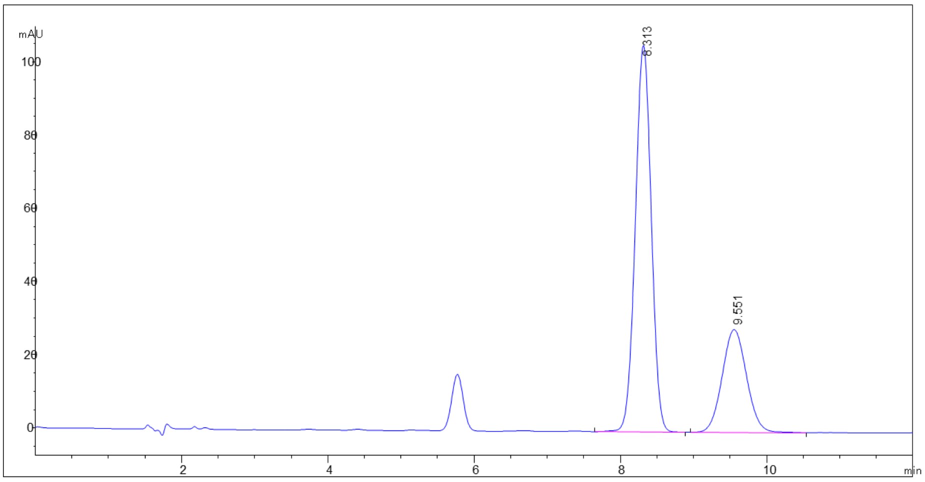

Peak	Retention time	Area	% Area
1	8.313	1975.5	71.086
2	9.551	649.8	28.914

HPLC for pure enantioenriched compound **4laa**

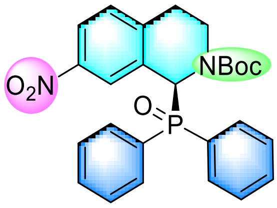

4maa
52% yield, 89% ee

Chiralpak OJ-RH column, MeCN/H_2O= 70/30, flow rate 1.0 mL/min

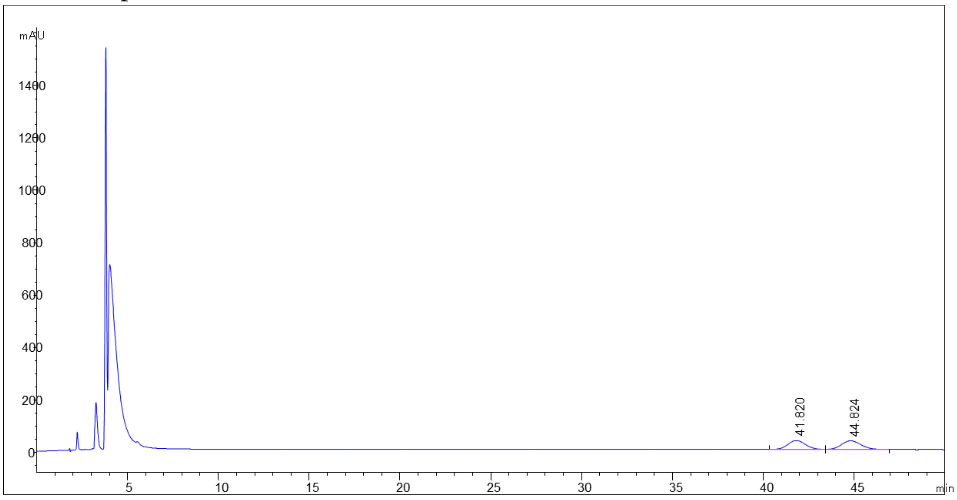

Peak	Retention time	Area	% Area
1	41.820	2387.3	50.096
2	44.824	2378.2	49.904

HPLC for racemic compound **4maa**

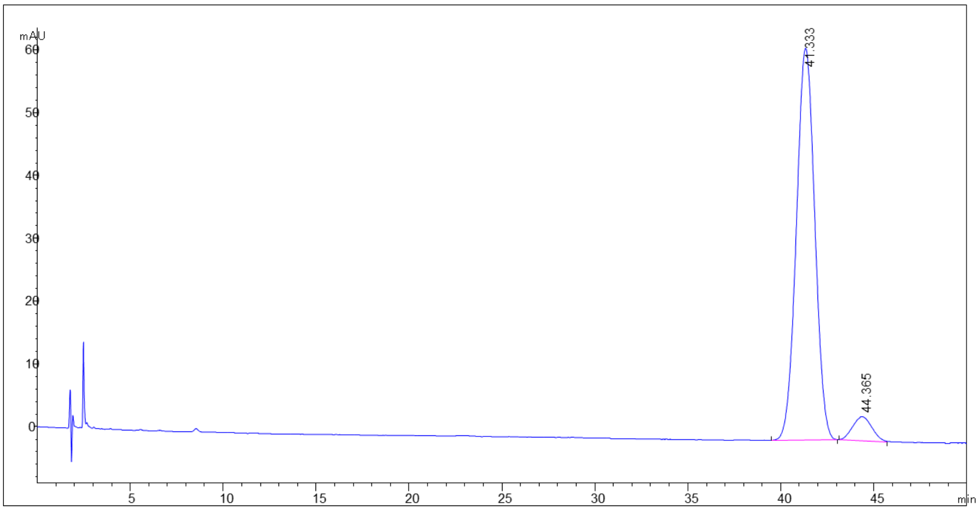

Peak	Retention time	Area	% Area
1	41.333	5262.7	94.503
2	44.365	361.2	5.497

HPLC for pure enantioenriched compound **4maa**

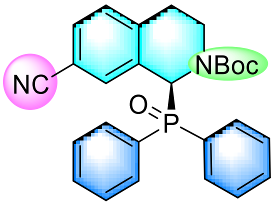

4naa

86% yield, 90% ee

Chiralpak OJ-RH column, MeCN/H_2O= 72/28, flow rate 1.0 mL/min

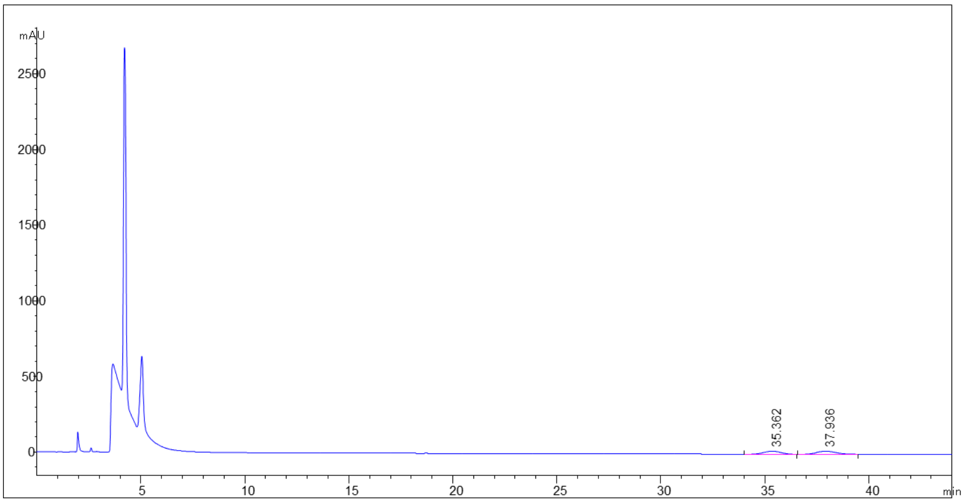

Peak	Retention time	Area	% Area
1	35.262	1305.4	49.409
2	37.936	1336.6	50.091

HPLC for racemic compound **4naa**

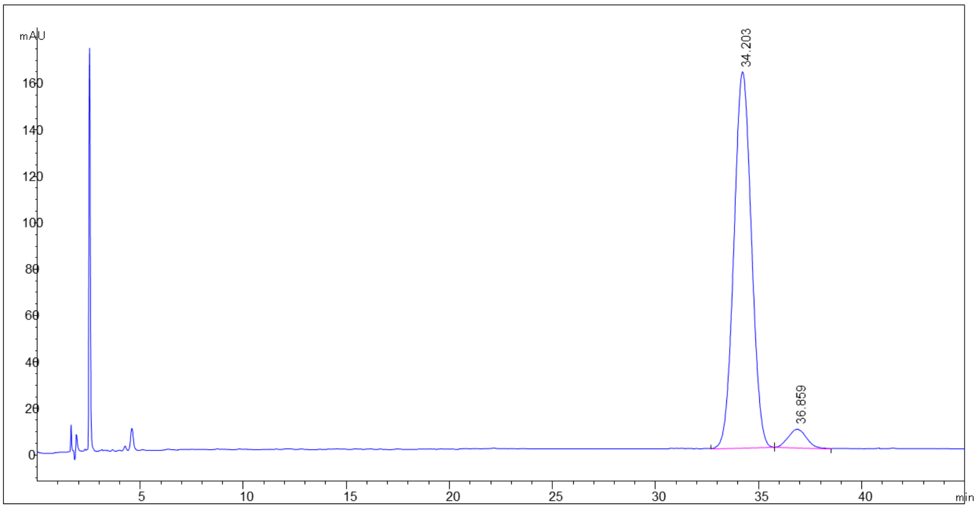

Peak	Retention time	Area	% Area
1	34.203	9554.1	95.044
2	36.859	498.2	4.956

HPLC for pure enantioenriched compound **4naa**

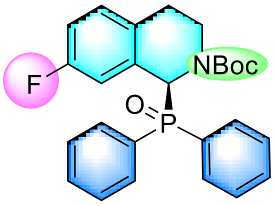

4oaa

92% yield, 94% ee

Chiralpak OJ-RH column, MeCN/H_2O= 60/40, flow rate 1.0 mL/min

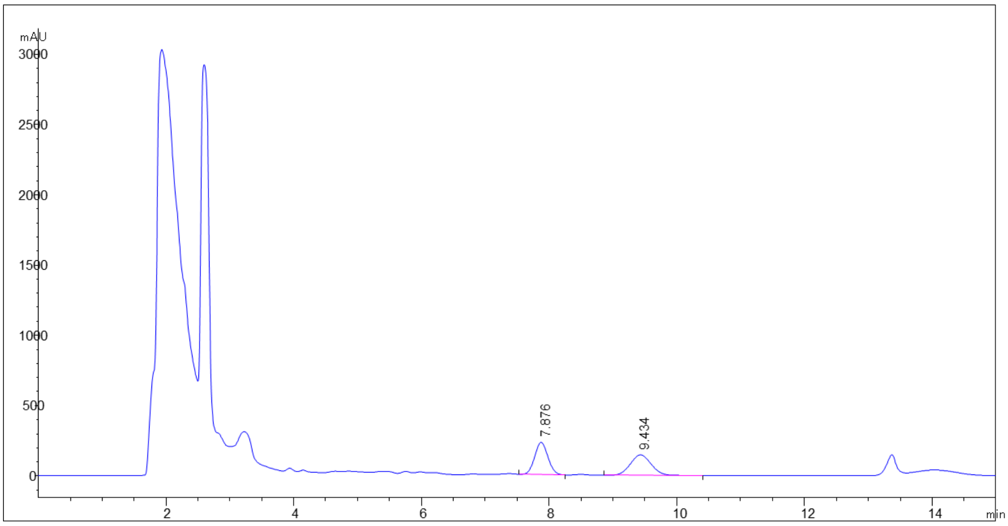

Peak	Retention time	Area	% Area
1	7.876	3337.6	50.083
2	9.434	3326.6	49.917

HPLC for racemic compound **4oaa**

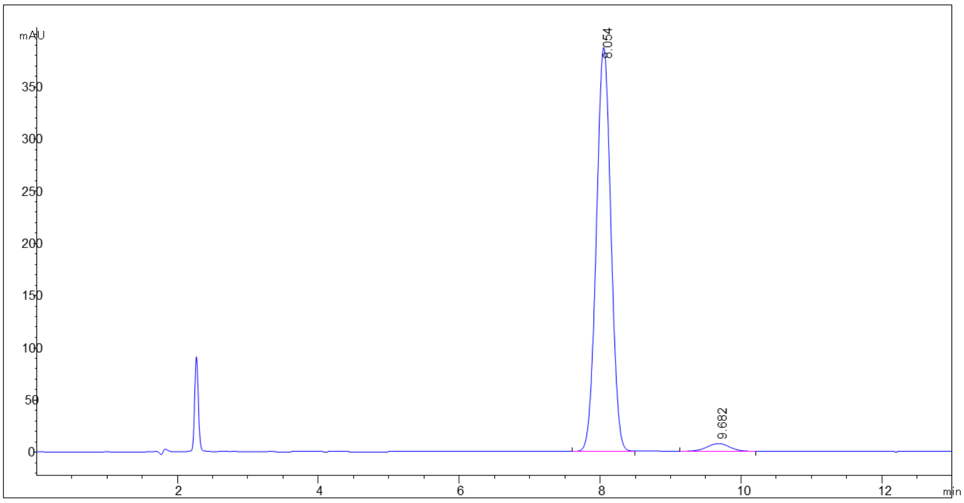

Peak	Retention time	Area	% Area
1	8.054	5551.9	97.088
2	9.682	166.5	2.912

HPLC for pure enantioenriched compound **4oaa**

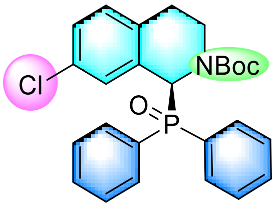

4paa
76% yield, 95% ee

Chiralpak OJ-RH column, MeCN/H_2O= 65/35, flow rate 1.0 mL/min

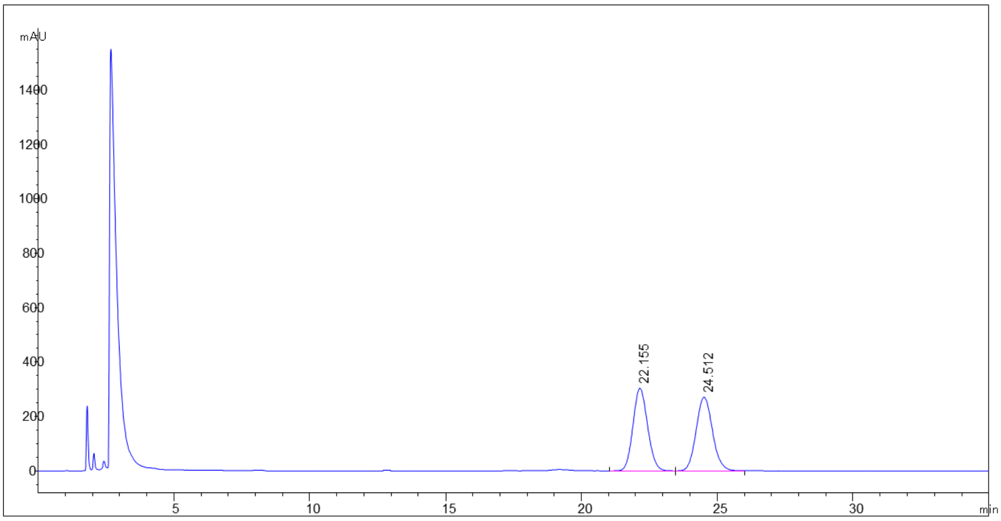

Peak	Retention time	Area	% Area
1	22.155	303.7	50.072
2	24.512	271.1	49.928

HPLC for racemic compound **4paa**

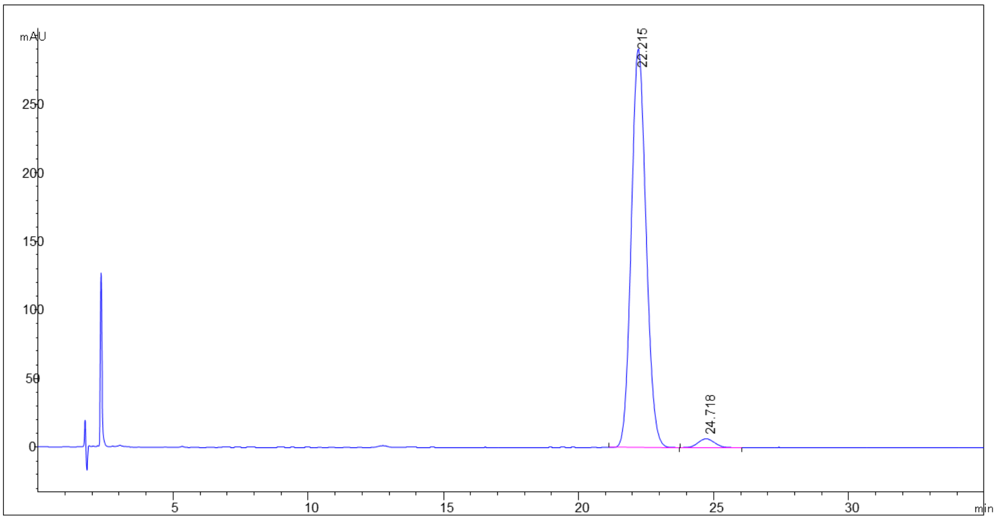

Peak	Retention time	Area	% Area
1	22.215	11017.5	97.543
2	24.718	277.6	2.457

HPLC for pure enantioenriched compound **4paa**

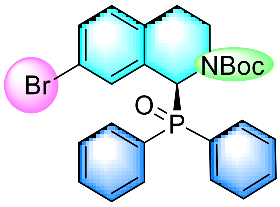

4qaa
56%yield, 76% ee

Chiralpak OD-RH-3um column, MeCN/H_2O= 65/35, flow rate 0.6 mL/min

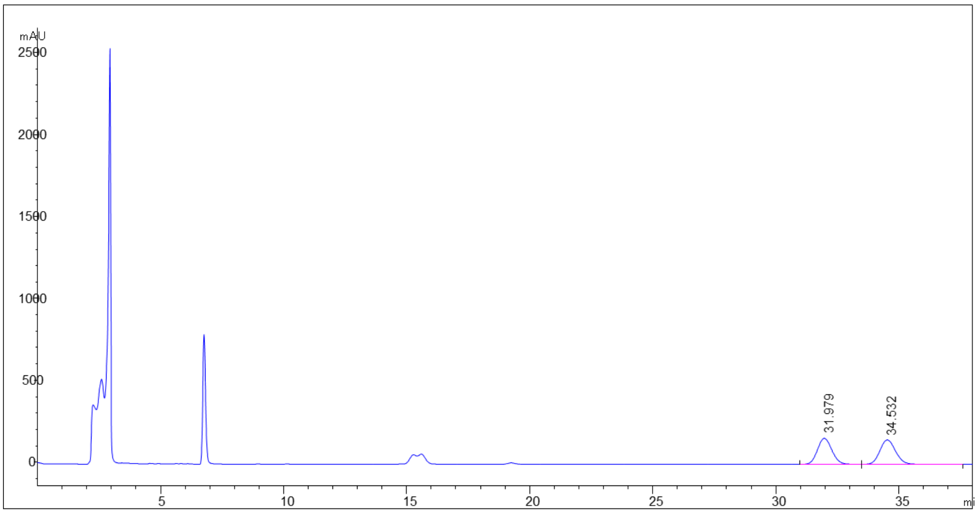

Peak	Retention time	Area	% Area
1	31.979	6644	49.917
2	34.532	6666.1	50.083

HPLC for racemic compound **4qaa**

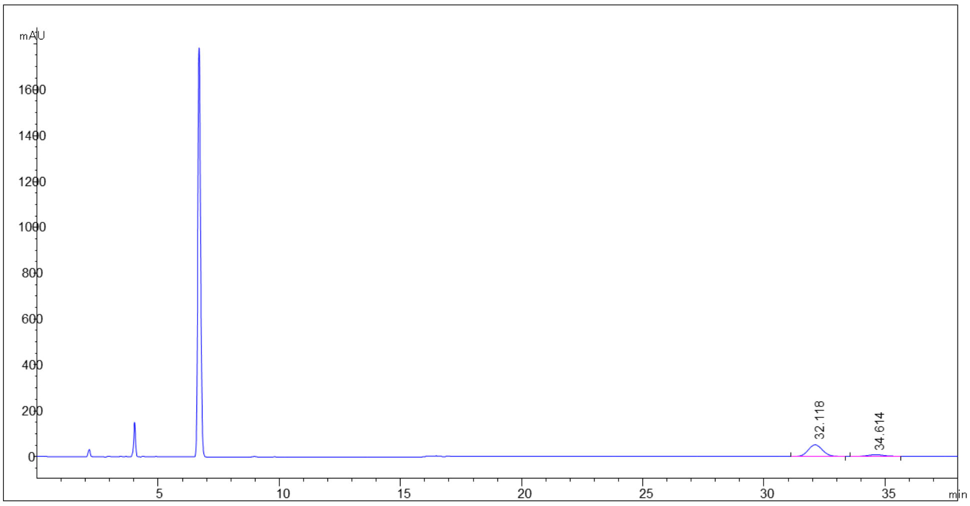

Peak	Retention time	Area	% Area
1	32.118	2160	87.846
2	34.614	298.8	12.154

HPLC for pure enantioenriched compound **4qaa**

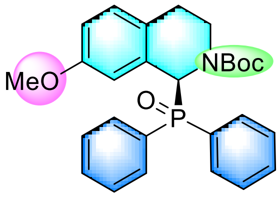

4raa

82% yield, 95% ee

Chiralpak OJ-RH column, MeCN/H_2O= 65/35, flow rate 1.0 mL/min

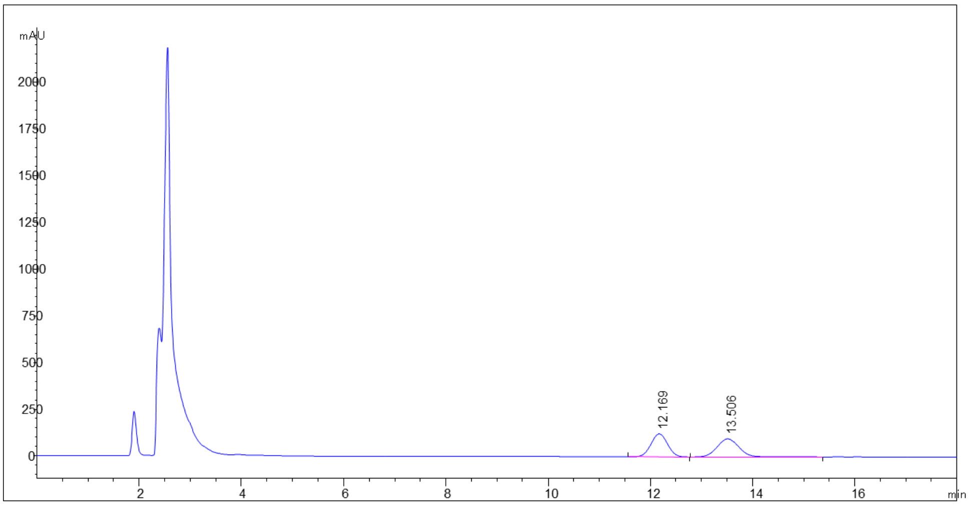

Peak	Retention time	Area	% Area
1	12.169	2814	49.263
2	13.506	2898.2	50.737

HPLC for racemic compound **4raa**

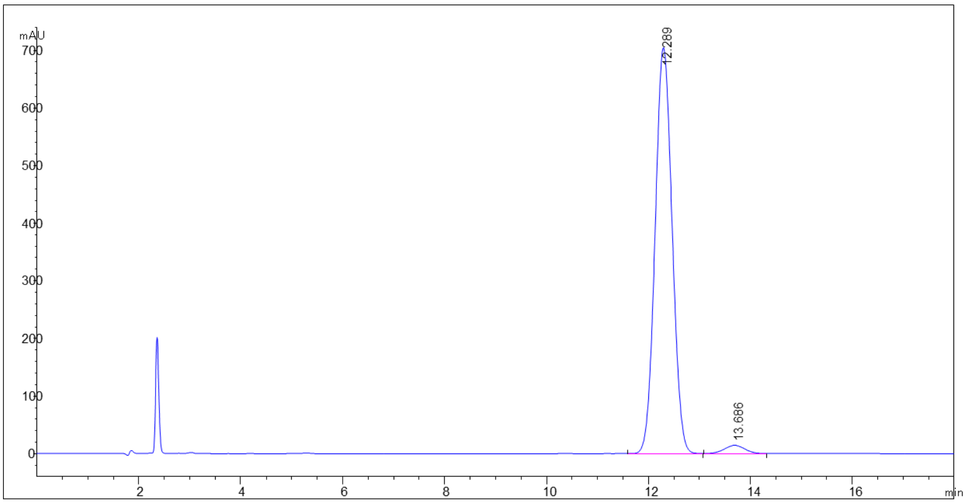

Peak	Retention time	Area	% Area
1	12.289	16291.4	97.682
2	13.686	386.7	2.318

HPLC for pure enantioenriched compound **4raa**

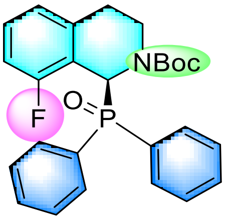

4saa

53% yield, 3% ee

Chiralpak AS-RH column, MeCN/H_2O= 50/50, flow rate 1.0 mL/min

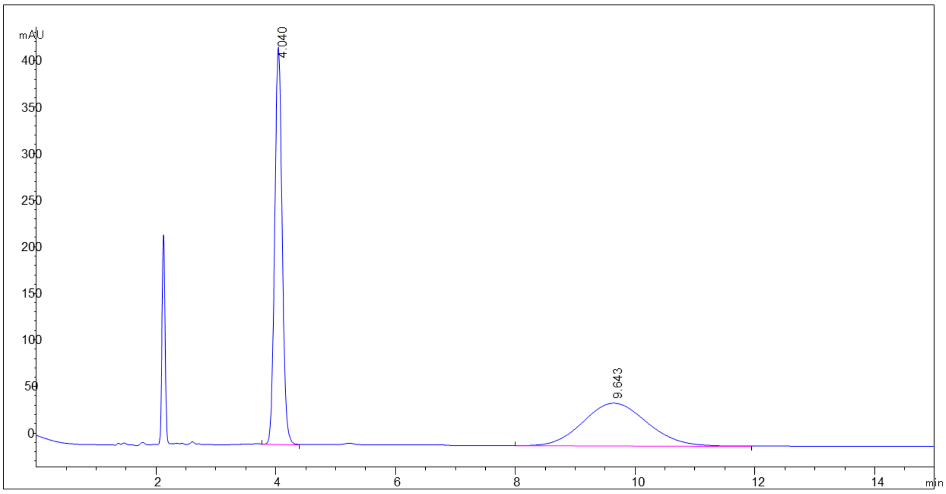

Peak	Retention time	Area	% Area
1	4.040	7044.6	49.645
2	9.643	7145.5	50.355

HPLC for racemic compound **4saa**

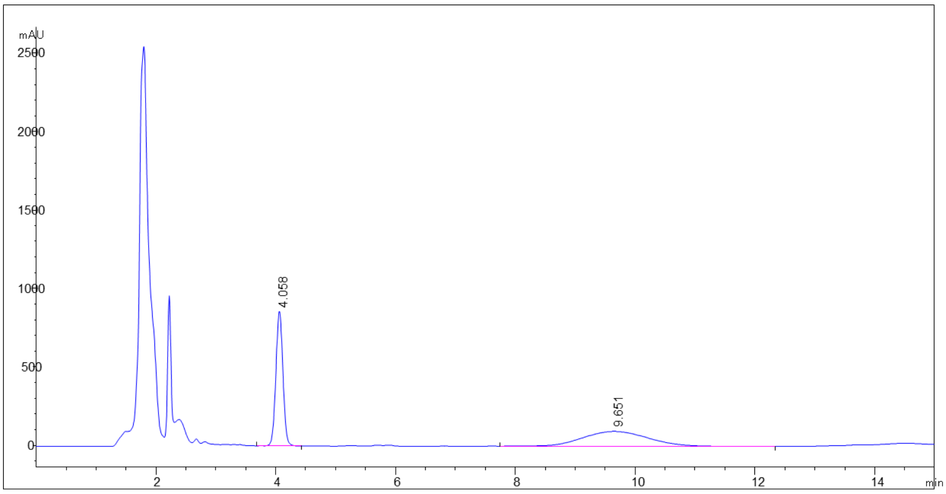

Peak	Retention time	Area	% Area
1	4.058	3679	51.674
2	9.651	3440.6	48.326

HPLC for pure enantioenriched compound **4saa**

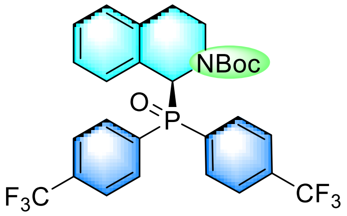

4aba, 92% yield, 79% *ee*

Chiralpak OD-RH column, MeCN/H_2O= 45/55, flow rate 1.0 mL/min

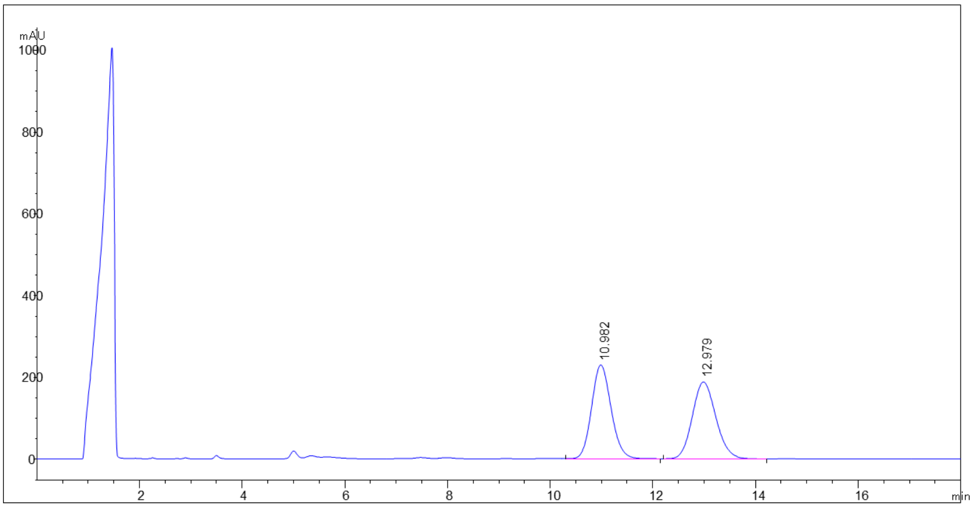

Peak	Retention time	Area	% Area
1	10.982	6085.4	50.166
2	12.979	6045.3	49.834

HPLC for racemic compound **4aba**

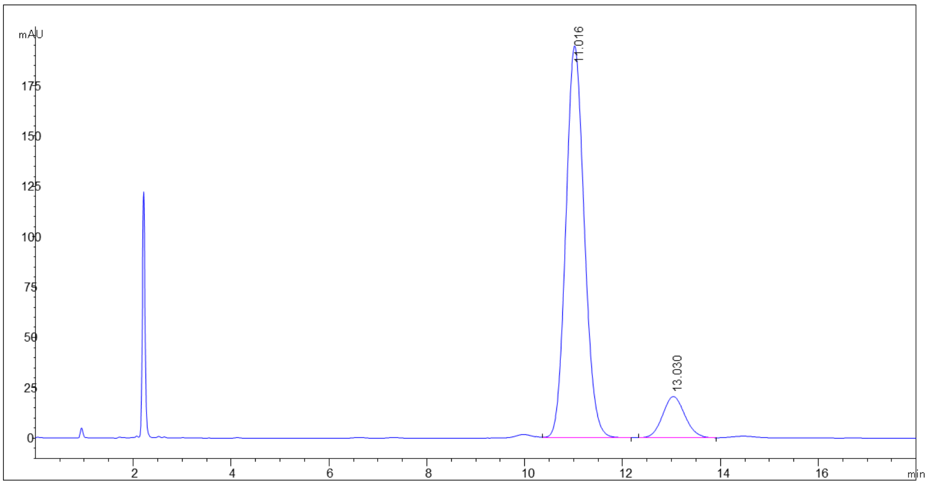

Peak	Retention time	Area	% Area
1	11.016	5530.4	89.587
2	13.030	642.8	10.413

HPLC for pure enantioenriched compound **4aba**

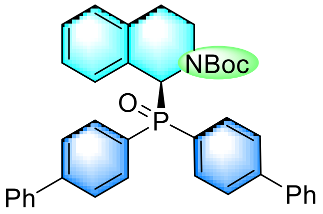

4aca, 88% yield, 82% ee

Chiralpak OD-RH column, MeCN/H_2O= 35/65, flow rate 1.0 mL/min

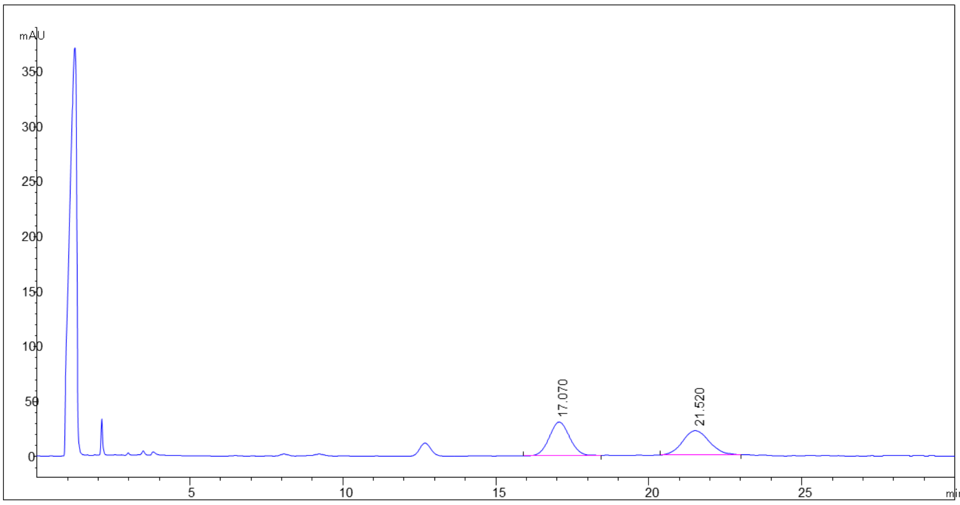

Peak	Retention time	Area	% Area
1	17.070	1419.9	50.945
2	21.520	1367.2	49.055

HPLC for racemic compound **4aca**

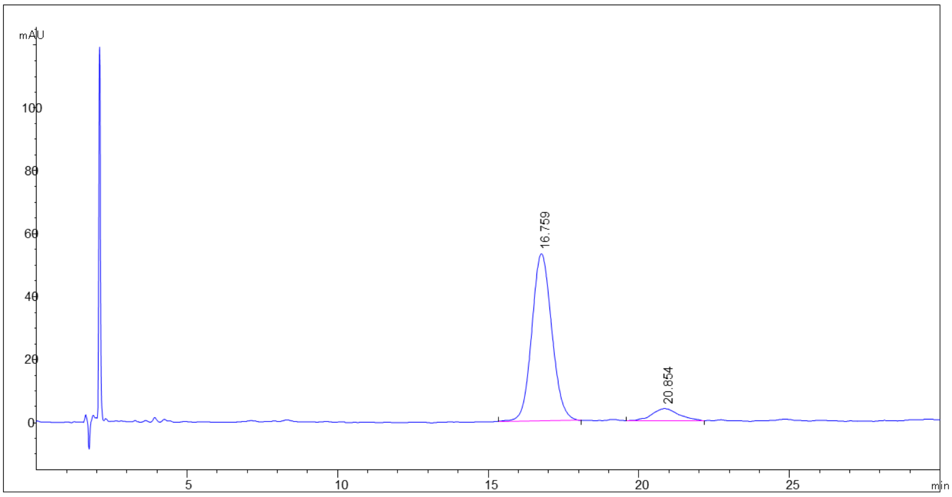

Peak	Retention time	Area	% Area
1	16.759	2451.4	90.986
2	20.854	242.9	9.014

HPLC for pure enantioenriched compound **4aca**

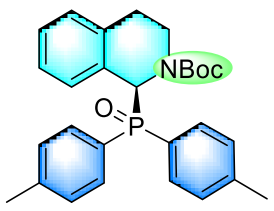

4ada, 85% yield, 88% ee

Chiralpak OD-RH column, MeCN/H_2O= 55/45, flow rate 1.0 mL/min

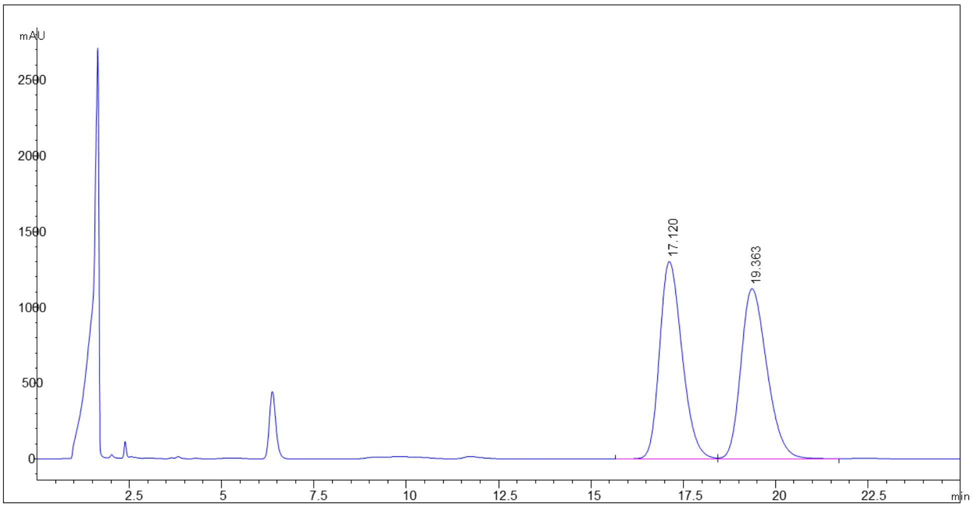

Peak	Retention time	Area	% Area
1	17.120	55243.3	50.063
2	19.363	55104.2	49.937

HPLC for racemic compound **4ada**

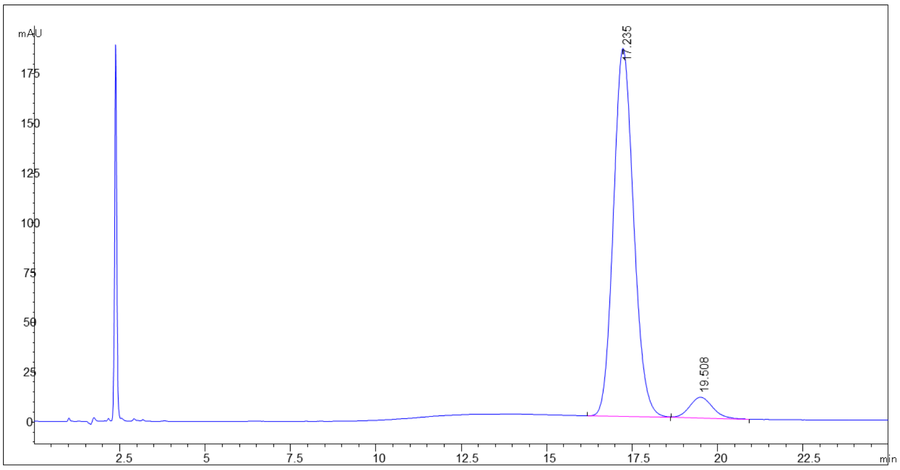

Peak	Retention time	Area	% Area
1	17.235	7610.8	94.179
2	19.508	470.4	5.821

HPLC for pure enantioenriched compound **4ada**

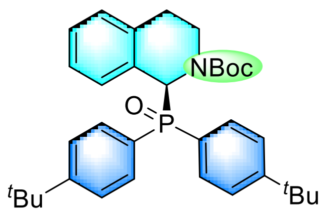

4**aea**, 88% yield, 91% ee

Chiralpak OD-RH column, MeCN/H_2O= 45/55, flow rate 1.0 mL/min

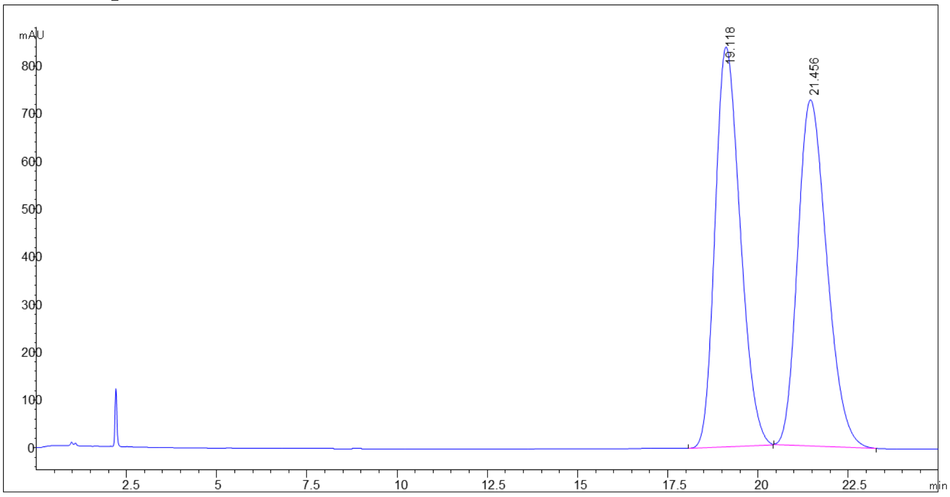

Peak	Retention time	Area	% Area
1	19.118	40659.4	50.054
2	21.456	40571.7	49.946

HPLC for racemic compound **4aea**

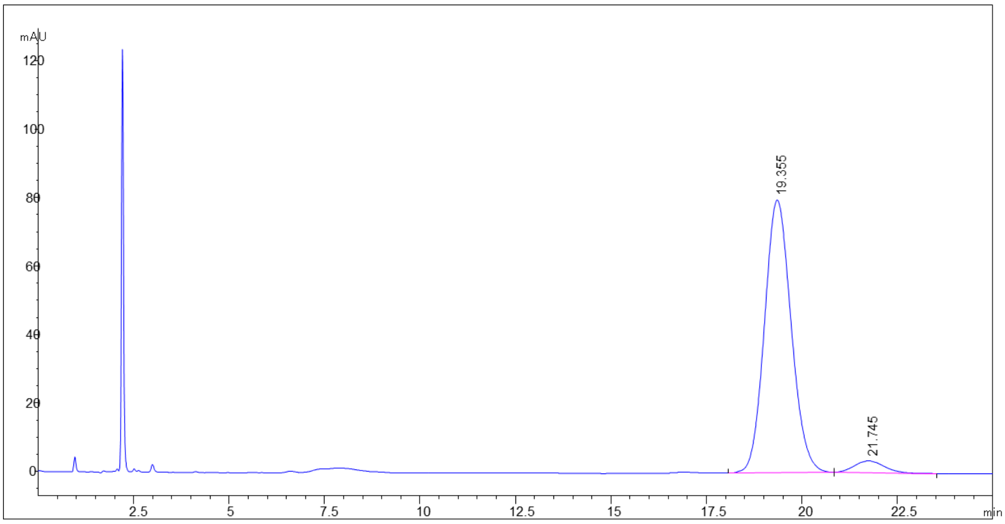

Peak	Retention time	Area	% Area
1	19.355	3861.5	95.220
2	21.745	193.9	4.780

HPLC for pure enantioenriched compound **4aea**

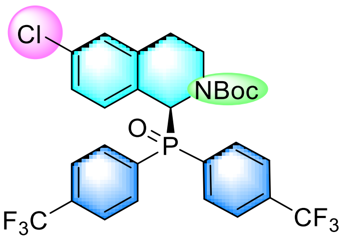

4iba, 98% yield, 86% *ee*

Chiralpak OJ-RH column, MeCN/H_2O= 45/55, flow rate 1.0 mL/min

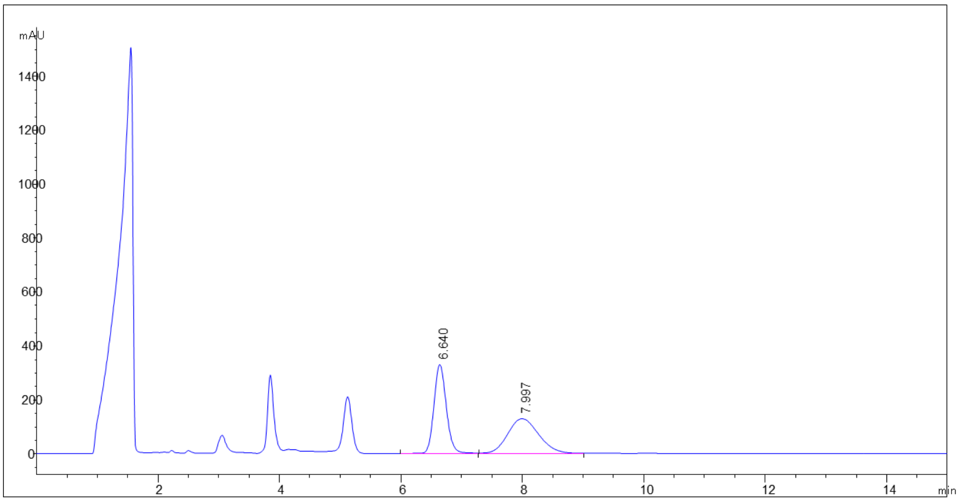

Peak	Retention time	Area	% Area
1	6.640	4644.6	50.507
2	7.997	4551.2	49.493

HPLC for racemic compound **4iba**

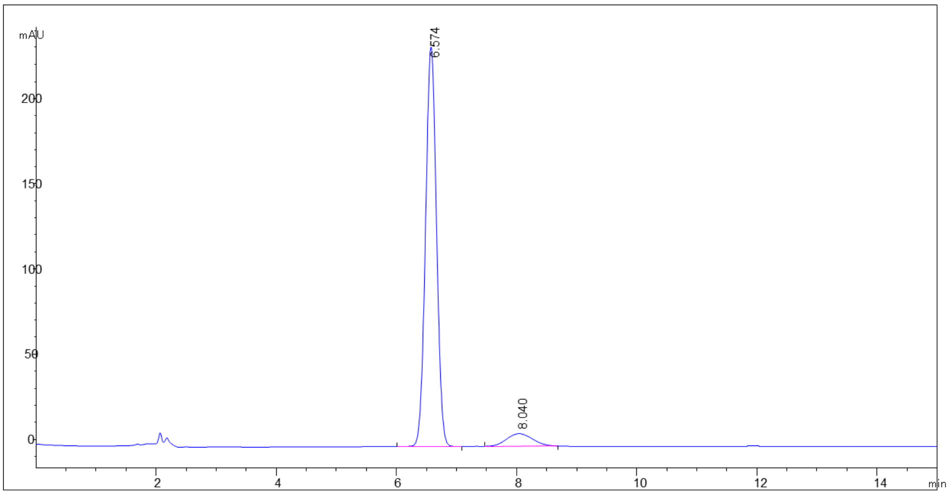

Peak	Retention time	Area	% Area
1	6.574	2976.4	92.955
2	8.040	225.6	7.045

HPLC for pure enantioenriched compound **4iba**

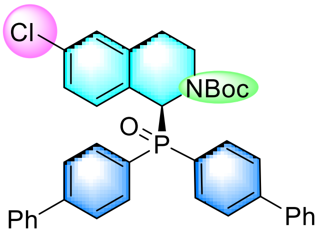

4ica, 93% yield, 93% *ee*

Chiralpak OD-RH column, MeCN/H_2O= 25/75, flow rate 1.0 mL/min

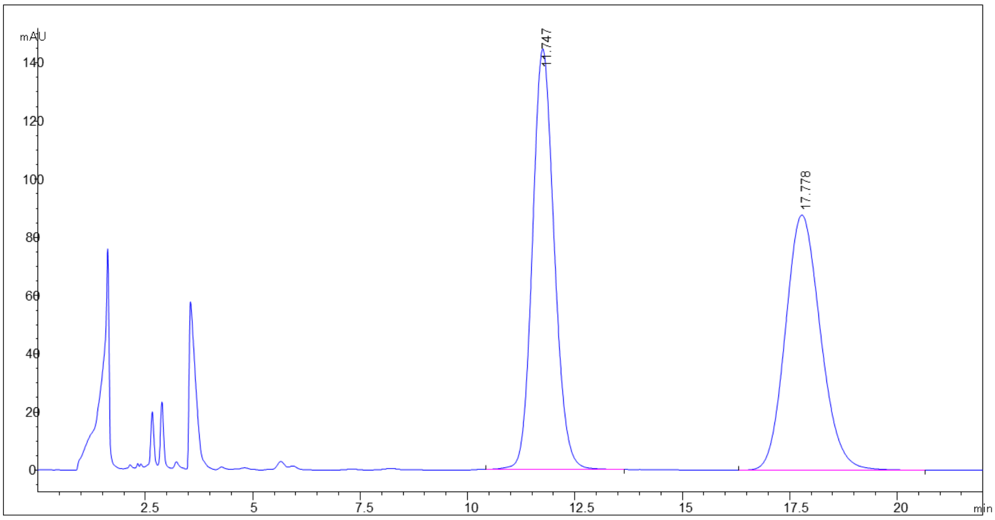

Peak	Retention time	Area	% Area
1	11.747	5080	50.372
2	17.778	5005	49.628

HPLC for racemic compound **4ica**

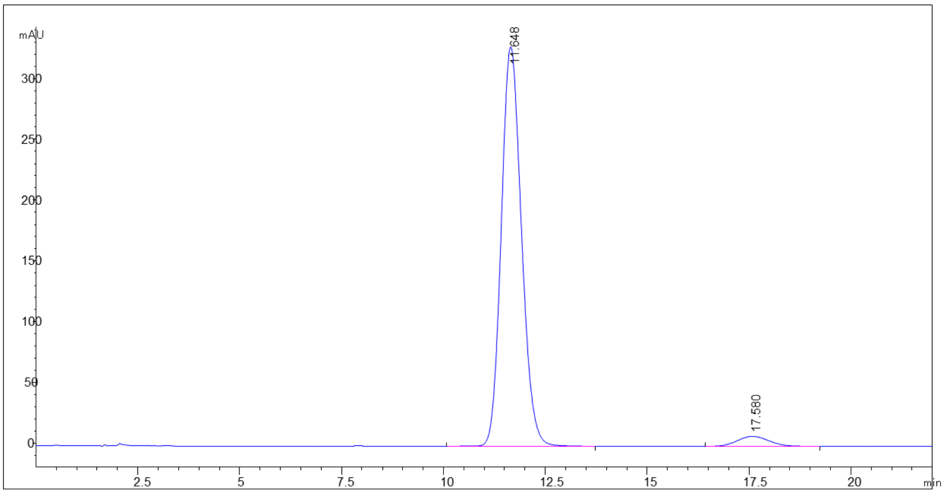

Peak	Retention time	Area	% Area
1	11.648	11304.6	96.290
2	17.580	435.6	3.710

HPLC for pure enantioenriched compound **4ica**

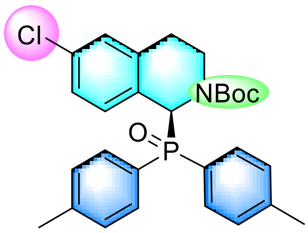

4ida, 83% yield, 90% *ee*

Chiralpak OD-RH column, MeCN/H2O= 40/60, flow rate 1.0 mL/min

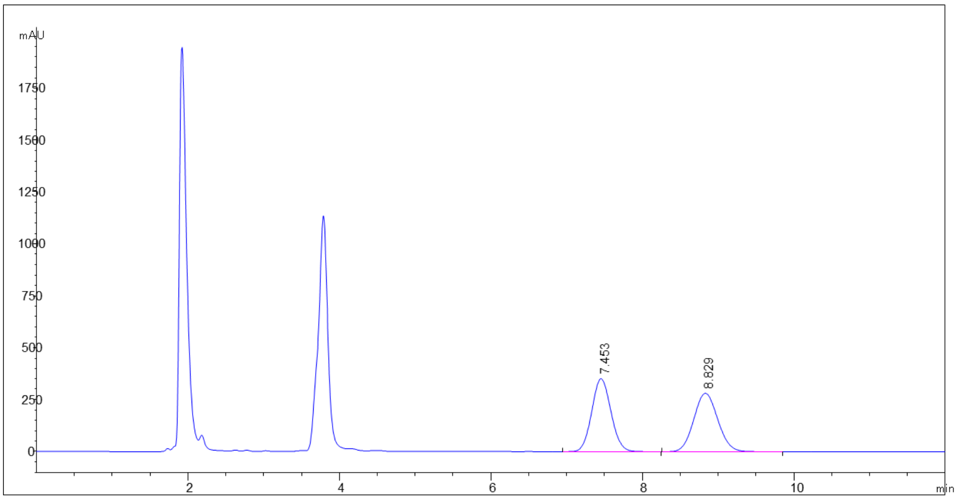

Peak	Retention time	Area	% Area
1	7.453	6211.8	49.987
2	8.829	6215	50.013

HPLC for racemic compound **4ida**

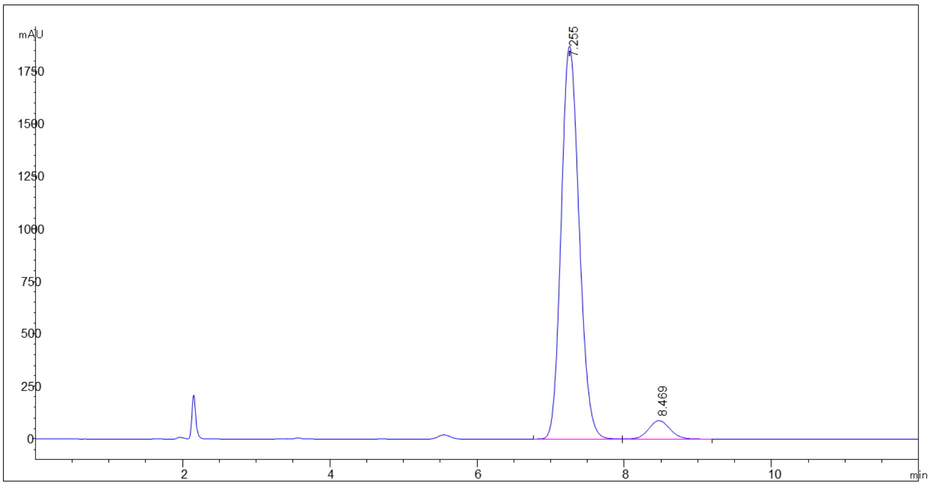

Peak	Retention time	Area	% Area
1	7.255	31719.9	94.781
2	8.469	1746.6	5.219

HPLC for pure enantioenriched compound **4ida**

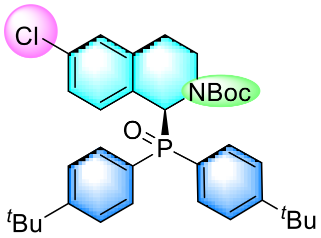

4iea, 85% yield, 95% *ee*

Chiralpak OJ-RH column, MeCN/H_2O= 45/55, flow rate 1.0 mL/min

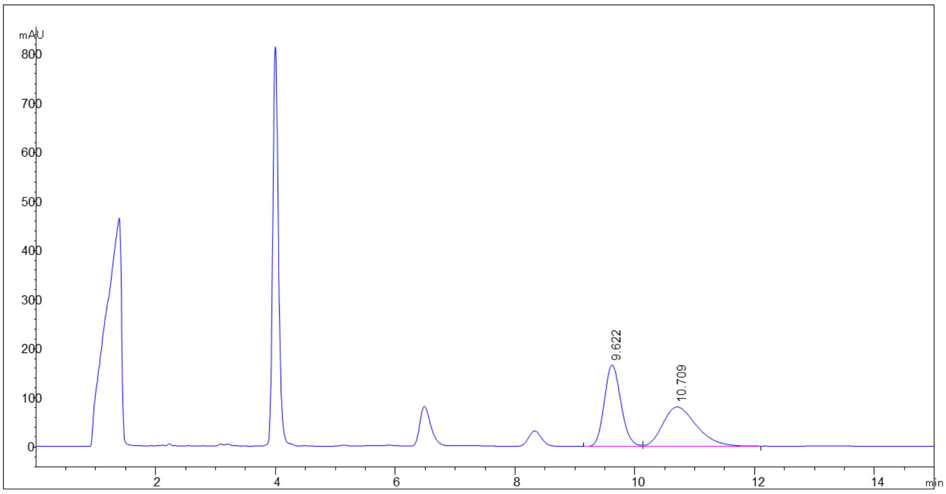

Peak	Retention time	Area	% Area
1	9.622	3193.2	50.211
2	10.709	3166.3	49.789

HPLC for racemic compound **4iea**

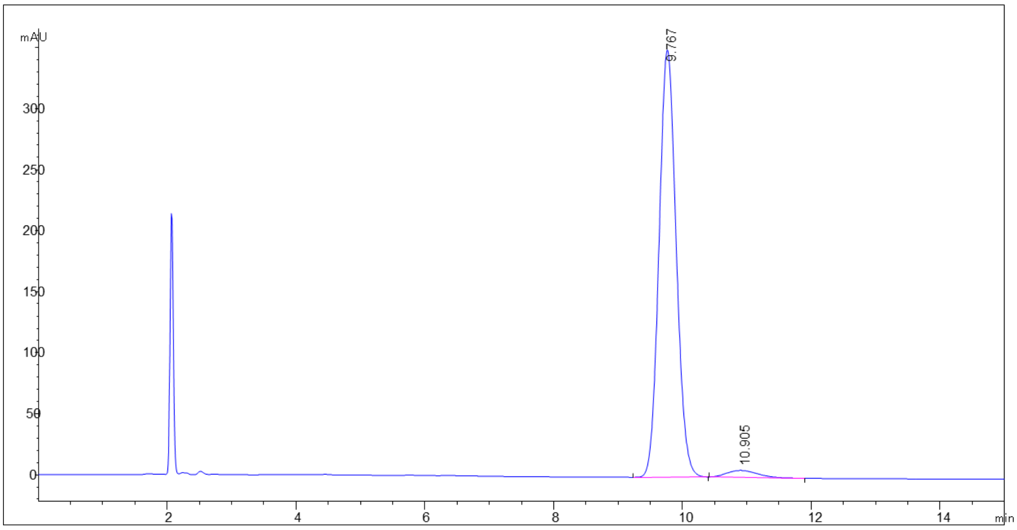

Peak	Retention time	Area	% Area
1	9.767	6472.1	97.398
2	10.905	173.9	2.602

HPLC for pure enantioenriched compound **4iea**

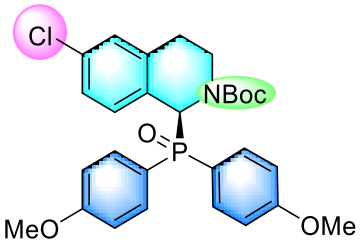

4ifa, 80% yield, 83% *ee*

Chiralpak AS-RH column, MeCN/H_2O= 55/45, flow rate 1.0 mL/min

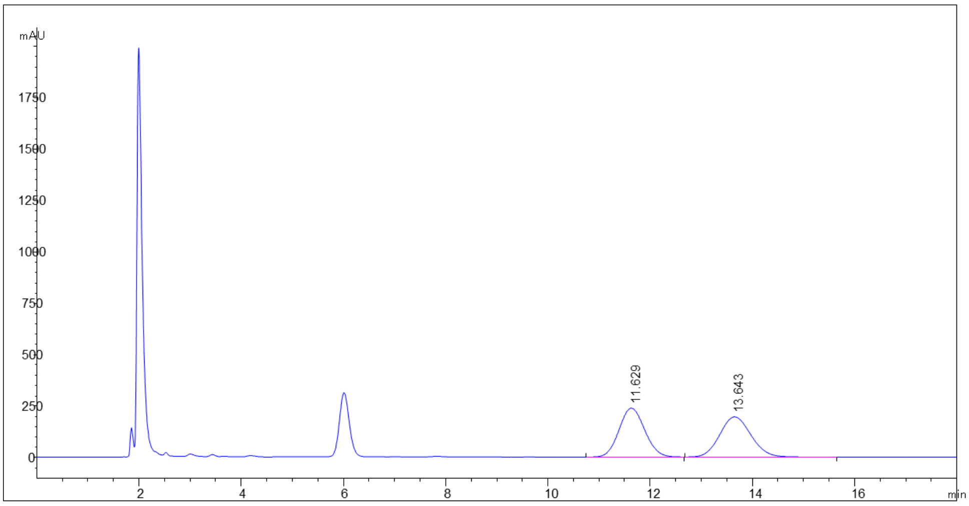

Peak	Retention time	Area	% Area
1	11.629	8360.8	49.931
2	13.643	8363.8	50.069

HPLC for racemic compound **4ifa**

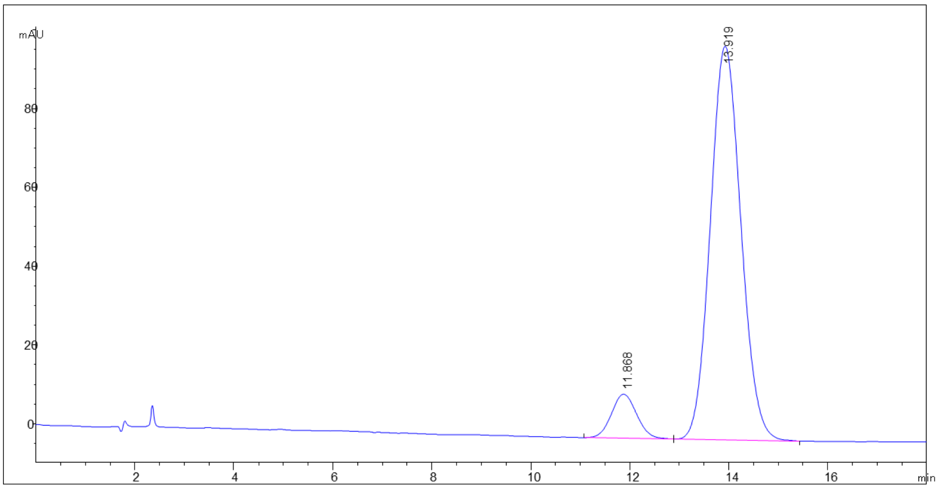

Peak	Retention time	Area	% Area
1	11.686	397	8.467
2	13.919	4292.2	91.533

HPLC for pure enantioenriched compound **4ifa**

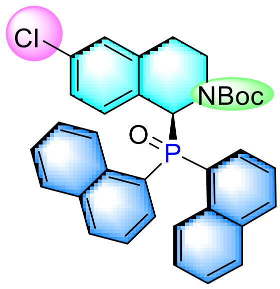

4iga, 80% yield, 91% *ee*

Chiralpak OJ-RH column, MeCN/H_2O= 50/50, flow rate 1.0 mL/min

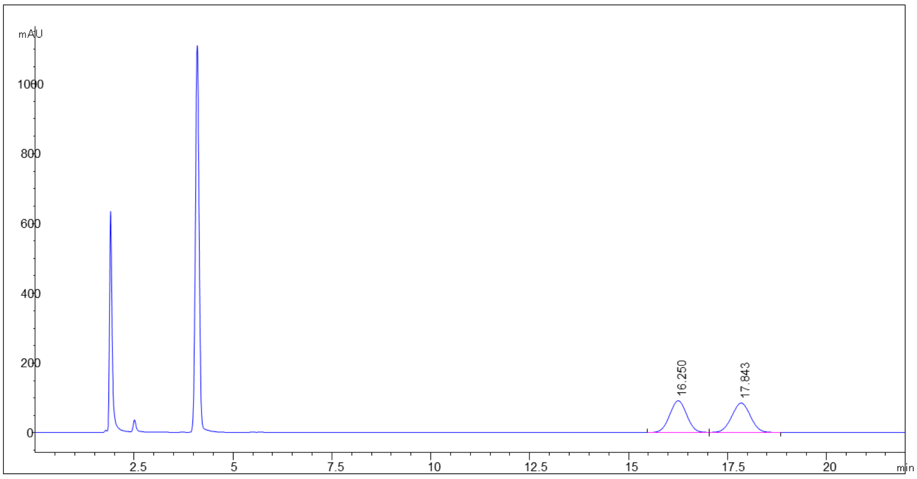

Peak	Retention time	Area	% Area
1	16.250	2754.7	50.006
2	17.751	2754	40.994

HPLC for racemic compound **4iga**

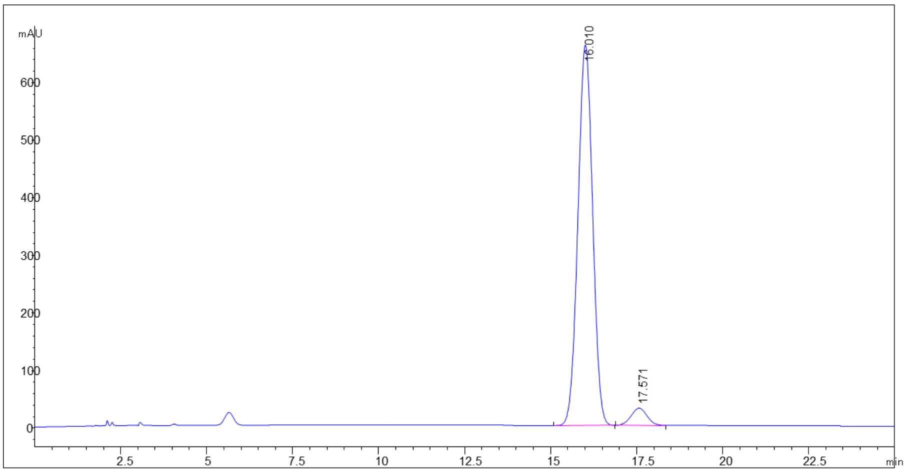

Peak	Retention time	Area	% Area
1	16.010	19623.5	95.343
2	17.778	958.4	4.657

HPLC for pure enantioenriched compound **4iea**

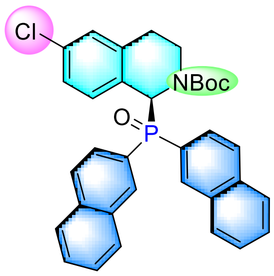

4iha, 98% yield, 96% *ee*

Chiralpak OJ-RH column, MeCN/H_2O= 45/55, flow rate 1.0 mL/min

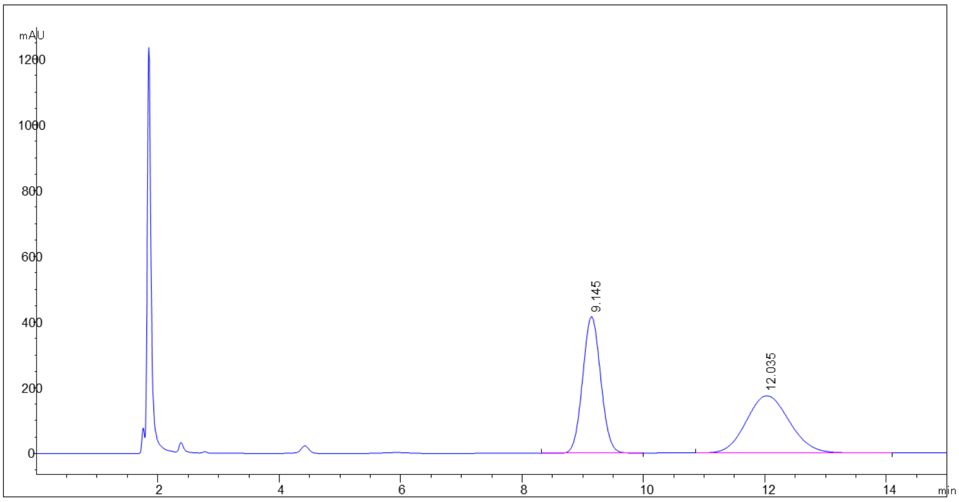

Peak	Retention time	Area	% Area
1	9.145	8614.4	49.892
2	12.035	8651.9	50.108

HPLC for racemic compound **4iha**

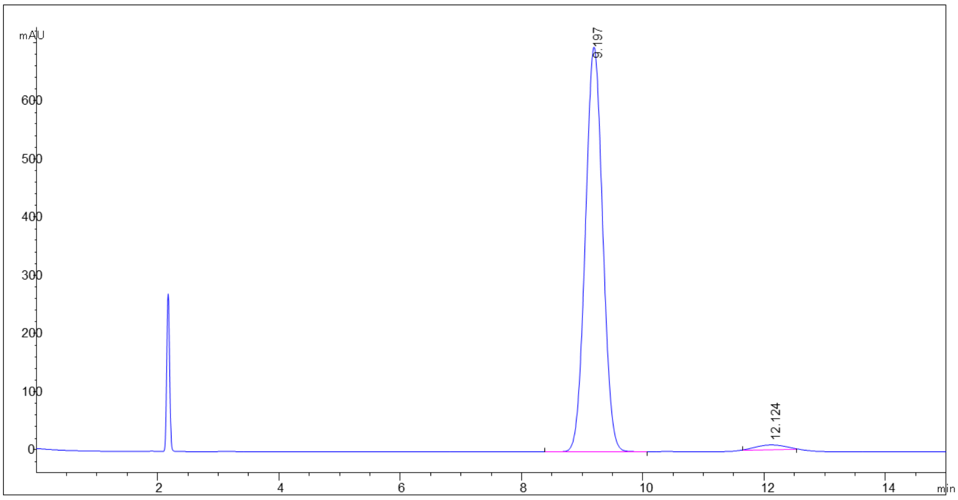

Peak	Retention time	Area	% Area
1	9.197	13814.8	98.193
2	12.124	254.3	1.807

HPLC for pure enantioenriched compound **4iha**

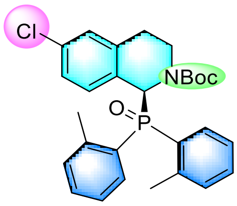

4iia, 88% yield, 90% *ee*

Chiralpak OD-RH-3um, CH_3CN/H_2O = 50/50 v/v, flow rate 0.6 mL/min

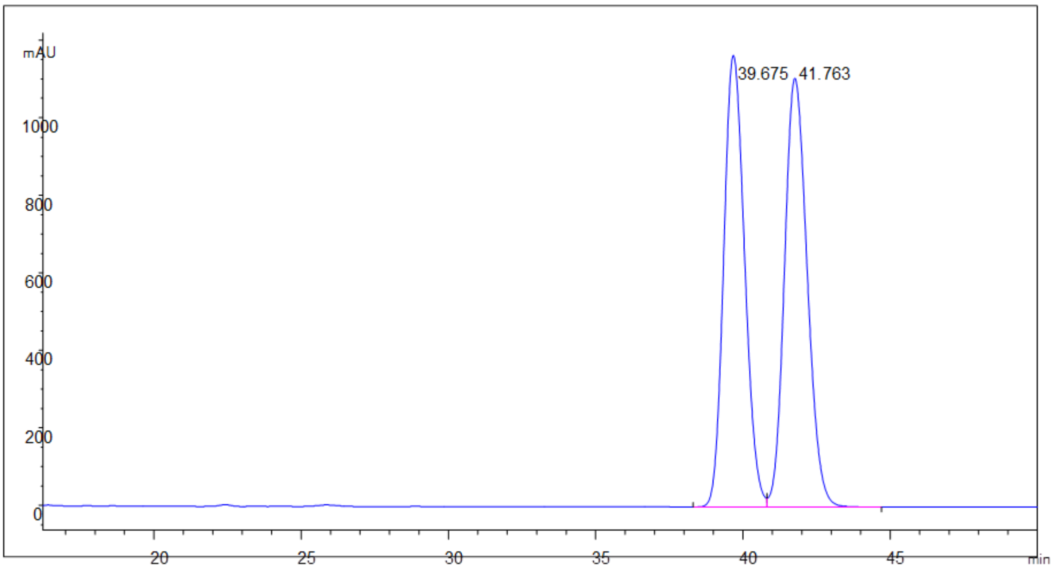

Peak	Retention time	Area	% Area
1	39.675	57449	49.197
2	41.763	59324	50.803

HPLC for racemic compound **4iia**

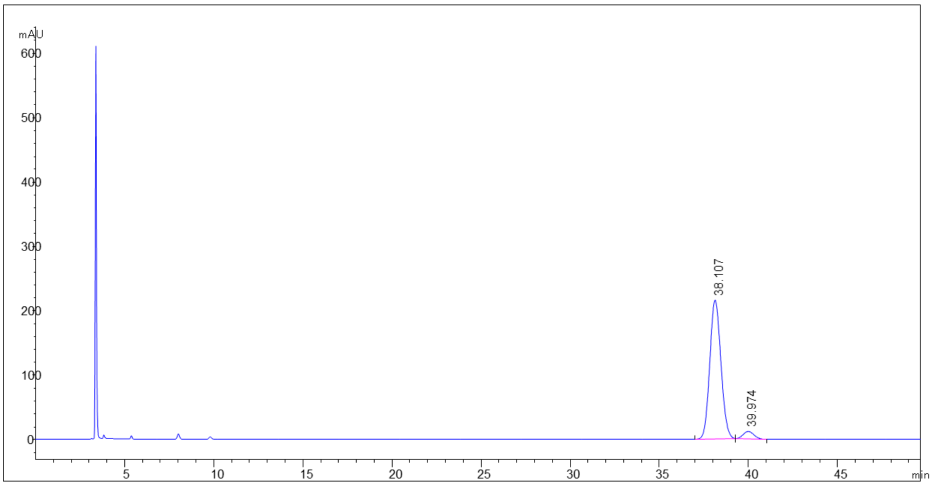

Peak	Retention time	Area	% Area
1	38.107	9399.9	95.084
2	39.974	486	4.916

HPLC for pure enantioenriched compound **4iia**

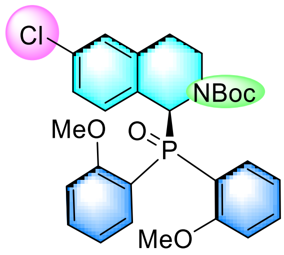

4ija, 78% yield, 86% ee

Chiralpak OX-RH column, MeCN/H_2O= 35/65, flow rate 1.0 mL/min

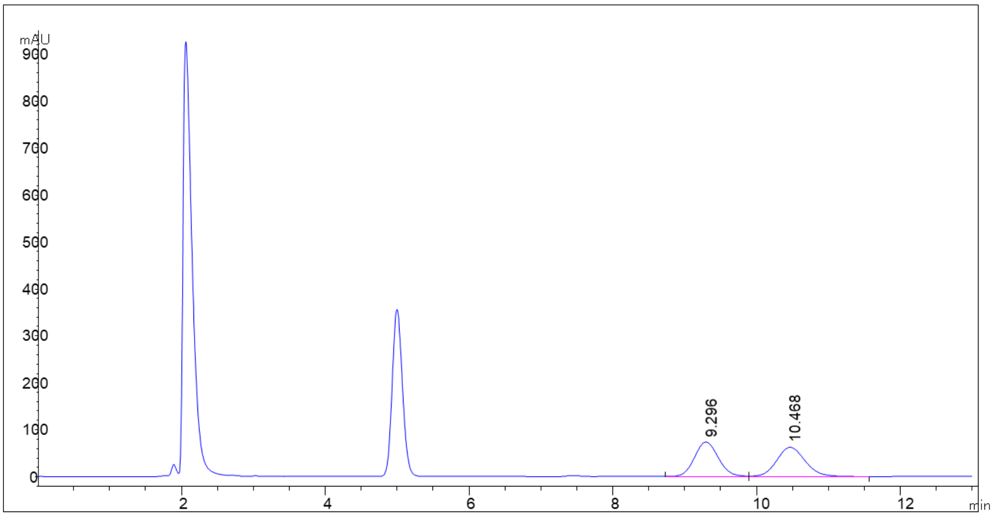

Peak	Ret. Time	Area	Area %
1	9.296	1675.4	49.145
2	10.468	1733.7	50.855

HPLC for racemic compound **4ija**

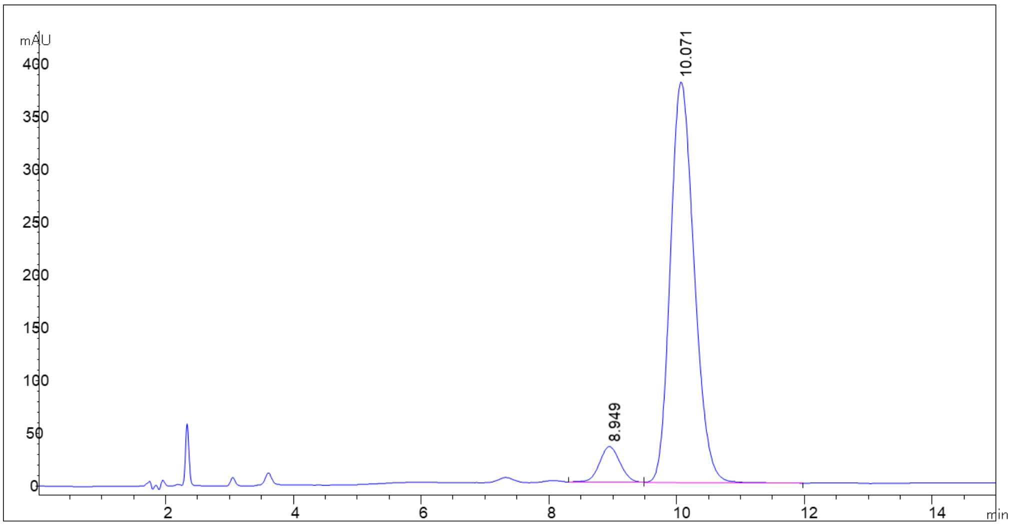

Peak	Ret. Time	Area	Area %
1	8.949	755	7.122
2	10.071	9846.8	92.878

HPLC for pure enantioenriched compound **4ija**

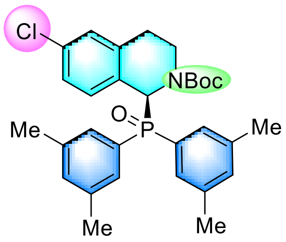

3ika, 85% yield, 94% *ee*

Chiralpak OX-RH column, MeCN/H_2O= 35/65, flow rate 1.0 mL/min

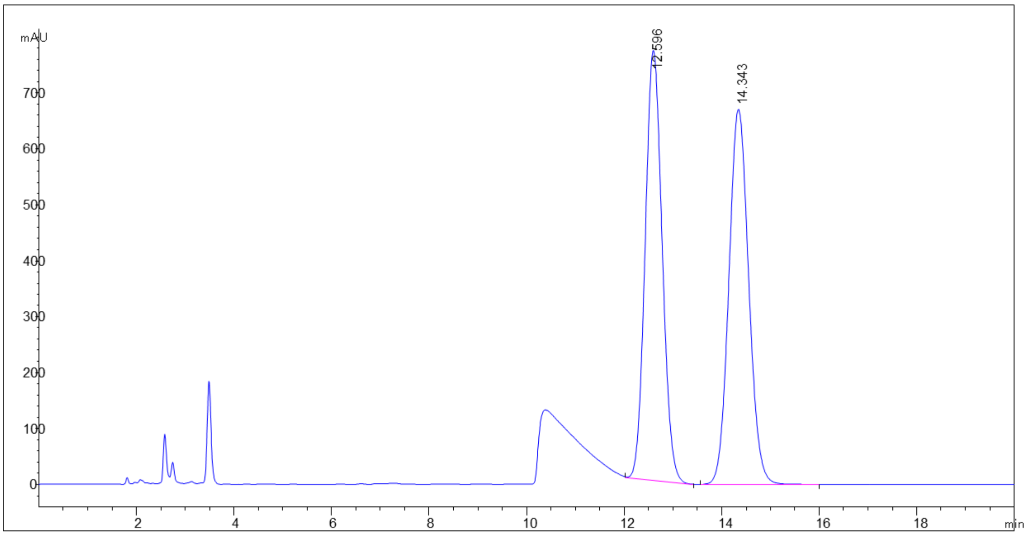

Peak	Retention time	Area	% Area
1	12.596	18604.8	49.531
2	14.343	18957.4	50.469

HPLC for racemic compound **4ika**

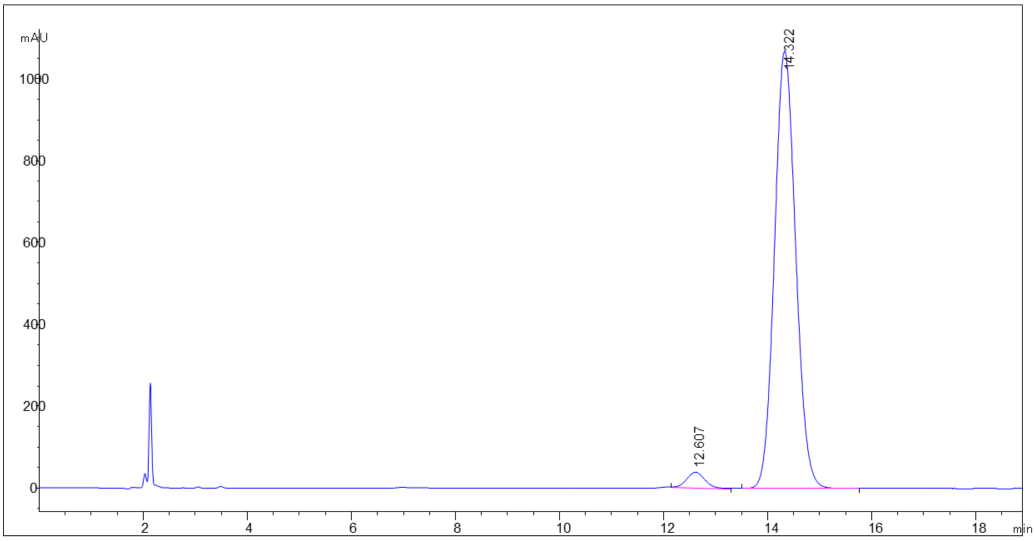

Peak	Retention time	Area	% Area
1	12.607	904.2	2.892
2	14.322	30362	97.108

HPLC for pure enantioenriched compound **4ika**

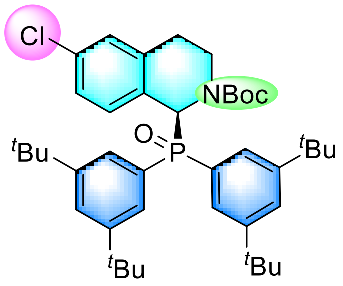

4ila, 60% yield, 96% *ee*

Chiralpak OX-RH column, MeCN/H_2O= 20/80, flow rate 1.0 mL/min

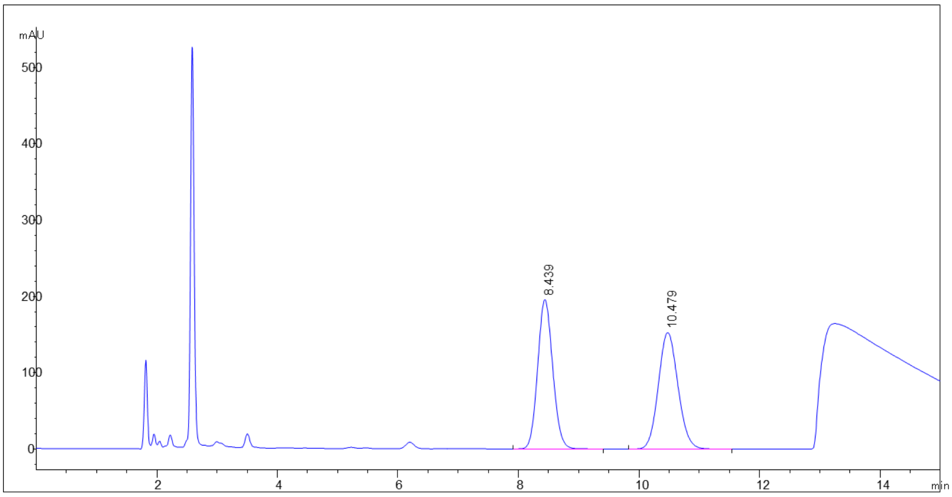

Peak	Retention time	Area	% Area
1	8.439	3396.8	49.637
2	10.479	3446.6	50.363

HPLC for racemic compound **4ila**

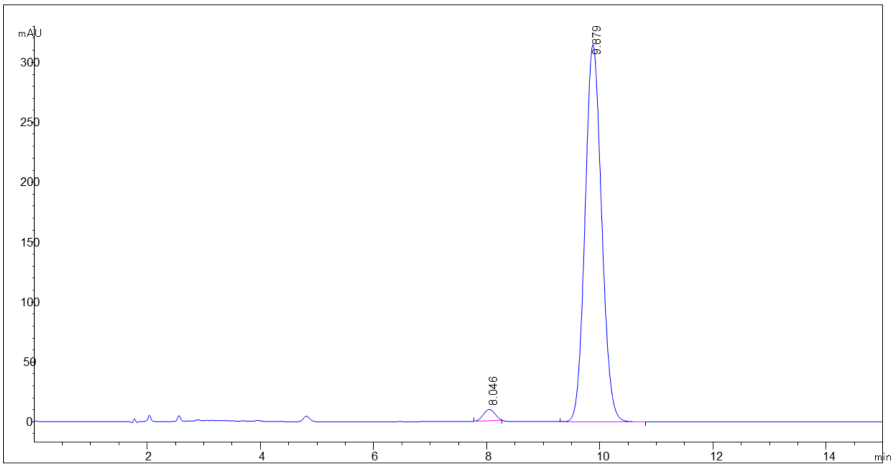

Peak	Retention time	Area	% Area
1	8.046	130	1.988
2	9.879	6046.8	98.012

HPLC for pure enantioenriched compound **4ila**

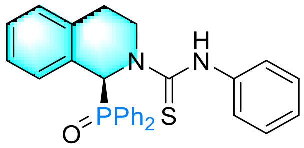

6aaa, 85% yield, 98% ee

Chiralpak OD-RH, MeCN/H_2O= 40/60, flow rate 1.0 mL/min

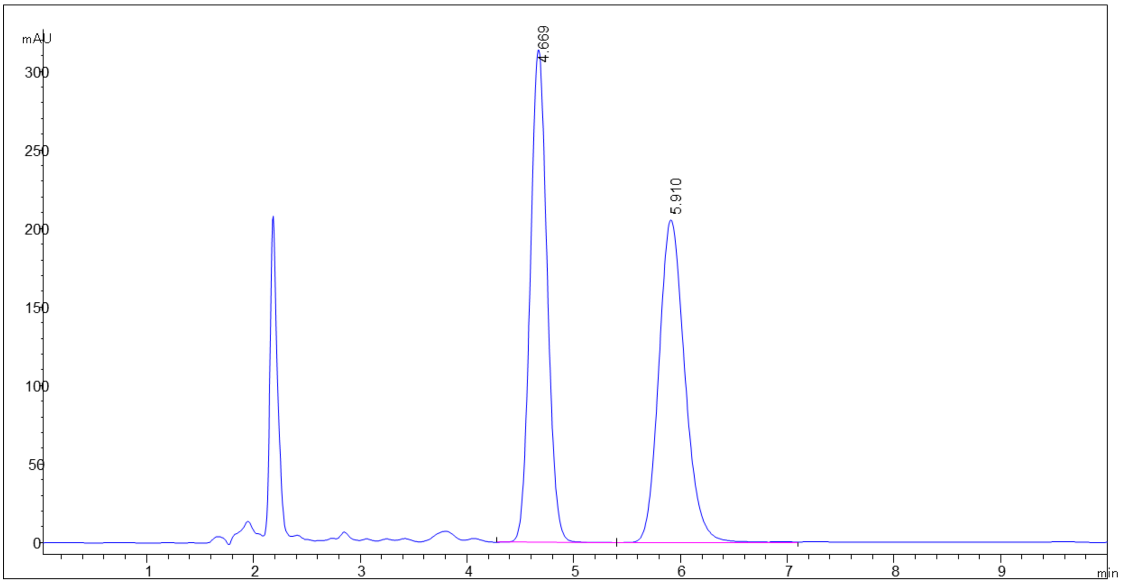

Peak	Ret. Time	Area	Area %
1	4.669	3455.3	49.845
2	5.91	3476.9	50.155

HPLC for racemic compound **6aab**

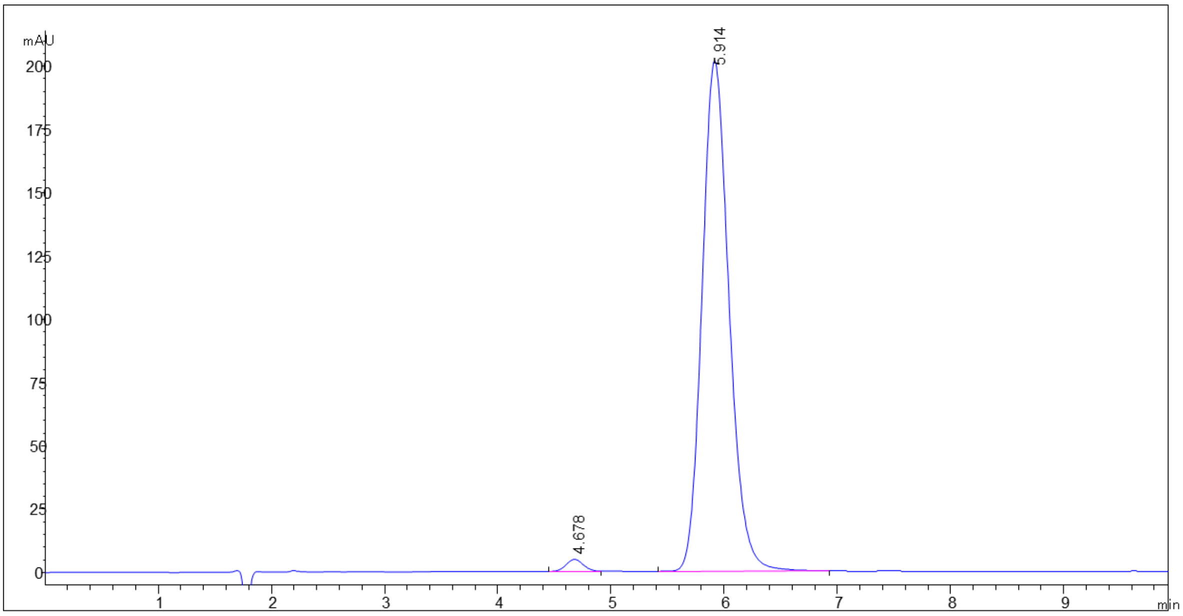

Peak	Ret. Time	Area	Area %
1	4.678	51.2	1.513
2	5.914	3334.7	98.487

HPLC for pure enantioenriched compound **6aab**

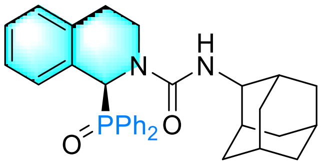

6aab, 88% yield, 99% ee

Chiralpak OD-RH, MeCN/H_2O= 50/50, flow rate 1.0 mL/min

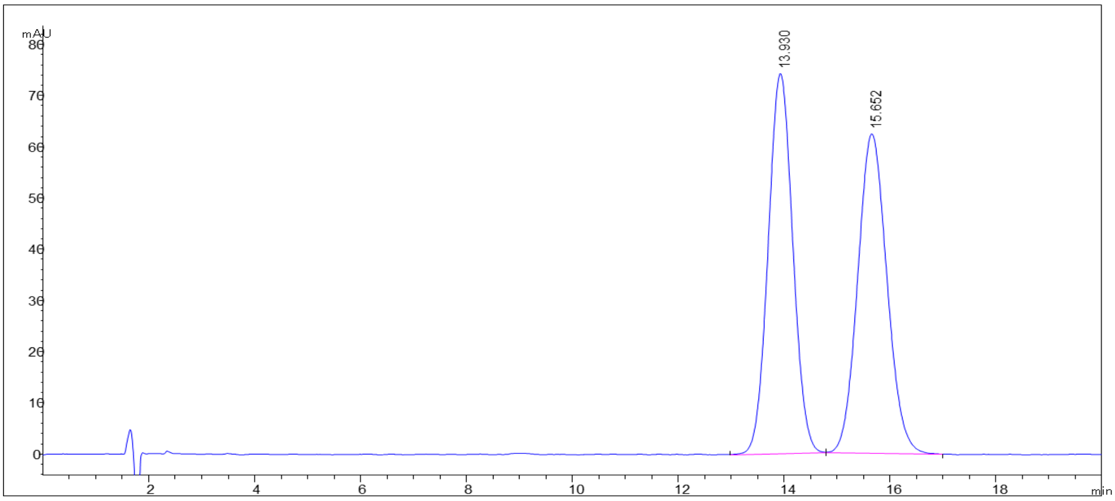

Peak	Ret. Time	Area	Area %
1	13.93	2384.4	50.015
2	15.652	2383	49.985

HPLC for racemic compound **6aab**

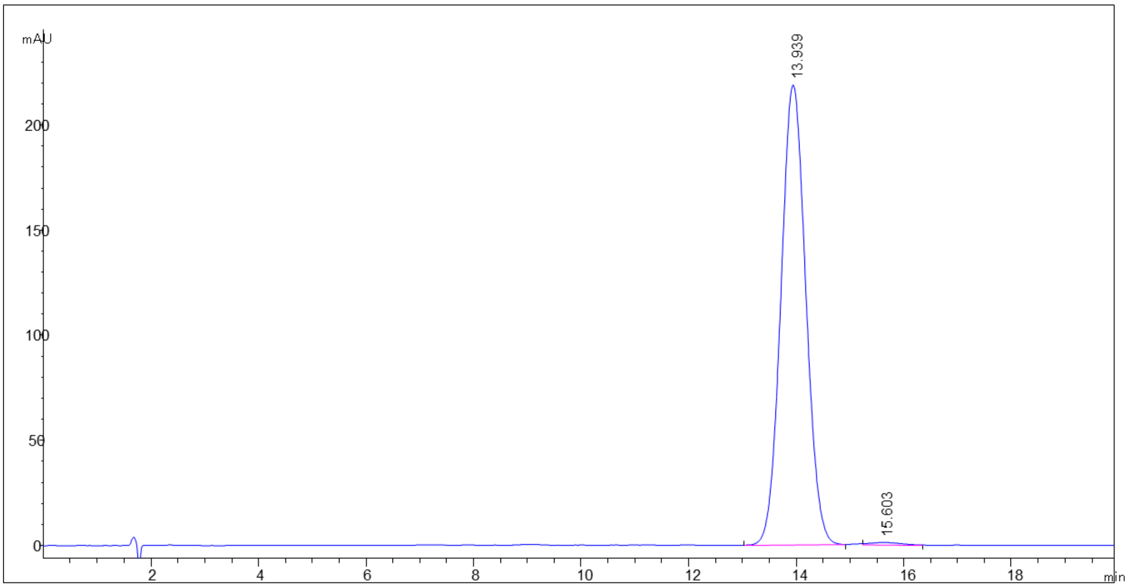

Peak	Ret. Time	Area	Area %
1	13.939	7031.2	99.414
2	15.603	41.4	0.586

HPLC for pure enantioenriched compound **6aab**

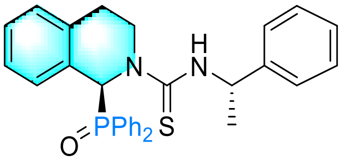

6aac, 93% yield, 98% ee

Chiralpak OJ-RH, MeCN/H_2O= 50/50, flow rate 1.0 mL/min

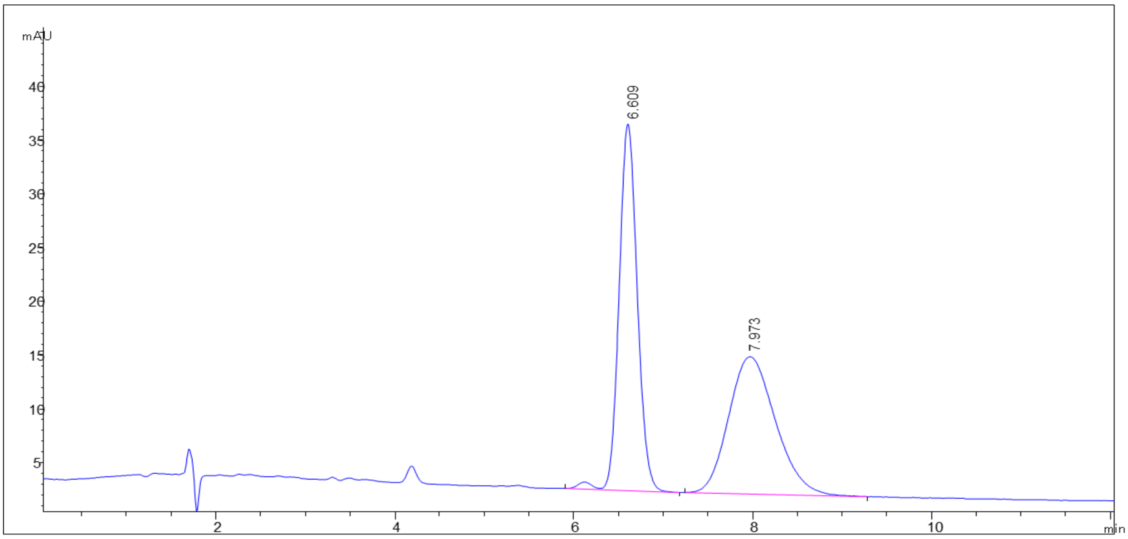

Peak	Ret. Time	Area	Area %
1	6.609	485.7	51.072
2	7.973	465.4	48.928

HPLC for racemic compound **6aac**

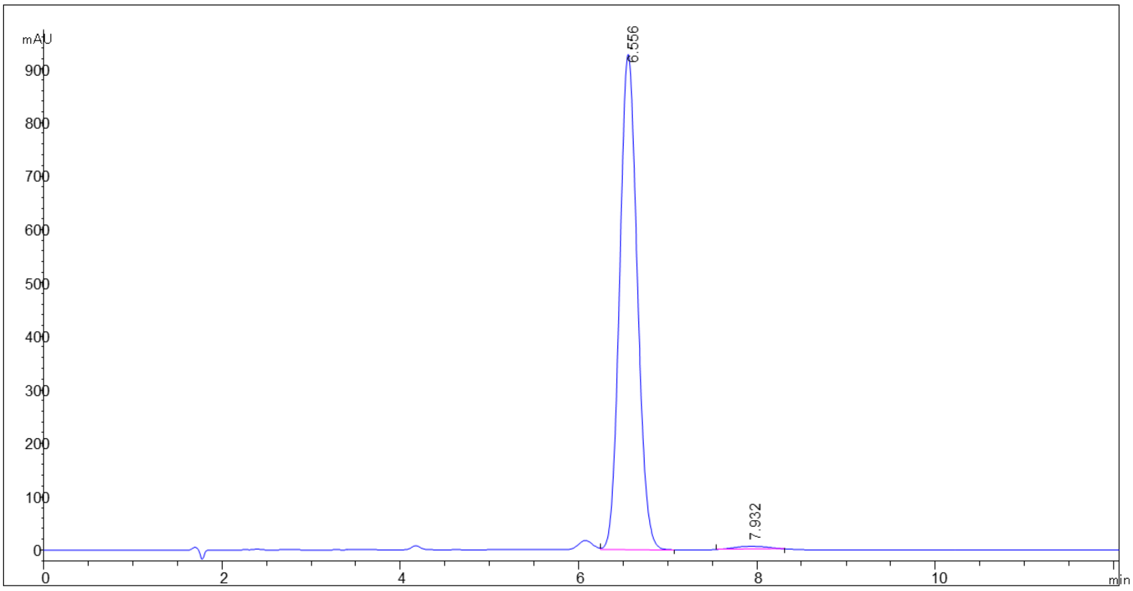

Peak	Ret. Time	Area	Area %
1	6.556	12715.8	98.761
2	7.932	159.6	1.239

HPLC for pure enantioenriched compound **6aac**

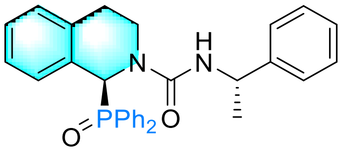

6aad, 96% yield, 98% ee

Chiralpak AS-RH, MeCN/H_2O= 50/50, flow rate 1.0 mL/min

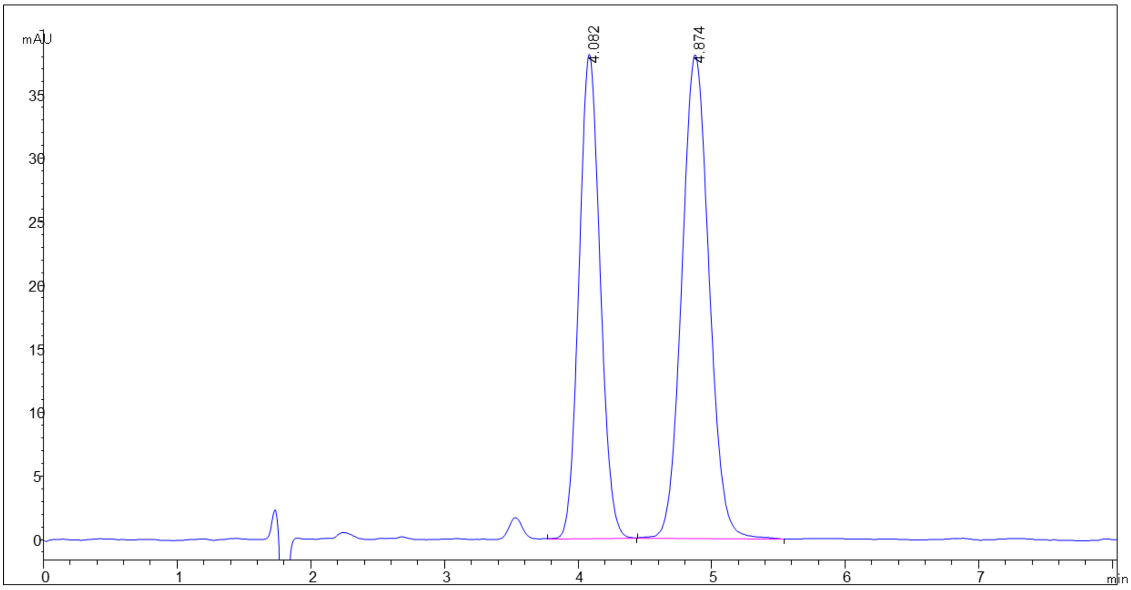

Peak	Ret. Time	Area	Area %
1	4.082	420	43.91
2	4.874	536.5	56.09

HPLC for racemic compound **6aad**

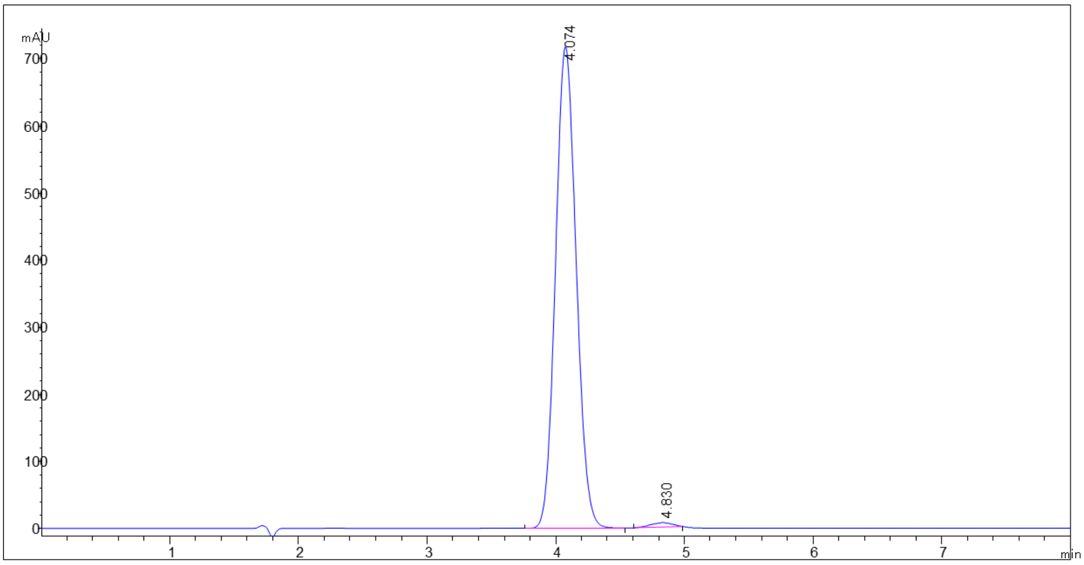

Peak	Ret. Time	Area	Area %
1	4.074	7962.4	99.028
2	4.83	78.2	0.972

HPLC for pure enantioenriched compound **6aad**

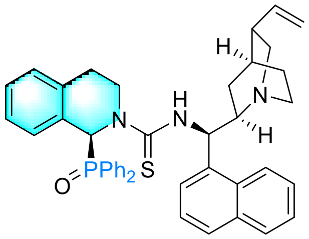

6aae, 86% yield, 99% ee

C8 column, MeCN/H_2O= 60/40, flow rate 1.0 mL/min

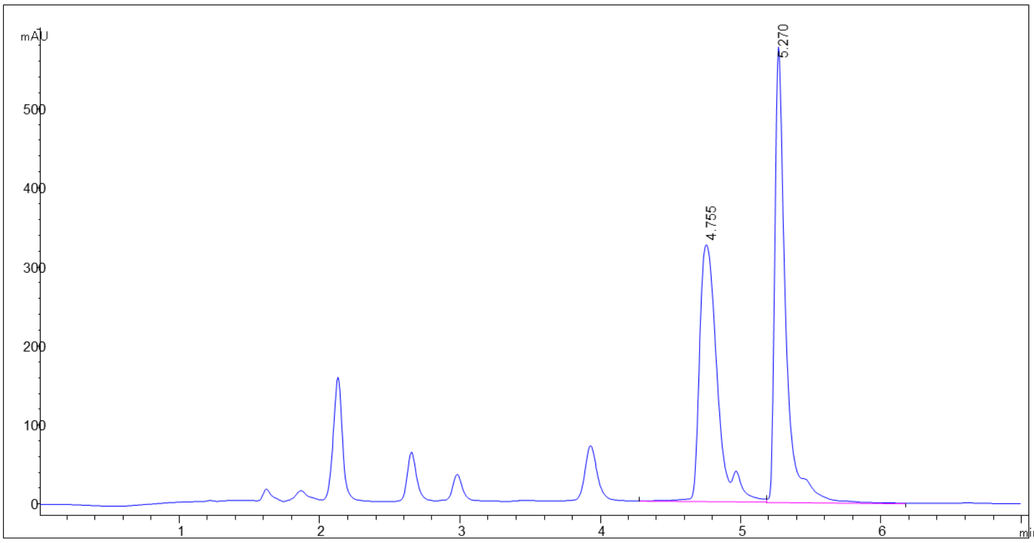

Peak	Ret. Time	Area	Area %
1	4.755	2899.4	48.33
2	5.27	3099.8	51.67

HPLC for racemic compound **6aae**

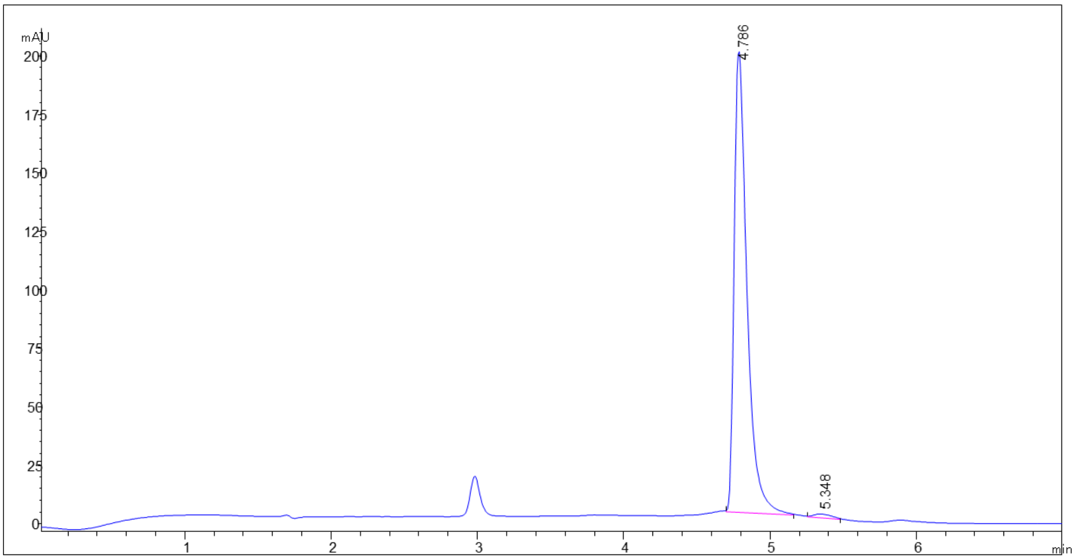

Peak	Ret. Time	Area	Area %
1	4.786	1245.1	99.347
2	5.348	8.2	0.653

HPLC for pure enantioenriched compound **6aae**
